# Bioinformatics investigation of adaptive immune‐related genes in peri‐implantitis and periodontitis: Characteristics and diagnostic values

**DOI:** 10.1002/iid3.1272

**Published:** 2024-05-23

**Authors:** Jingju Yin, Youran Fang, Yunyang Liao, Zhe Chen, Shaofeng Liu, Hanghang Zhu, Kun Song, Bin Shi

**Affiliations:** ^1^ Department of Oral and Maxillofacial Surgery The First Affiliated Hospital of Fujian Medical University Fuzhou China; ^2^ Oral Medicine Center, National Regional Medical Center, Binhai Campus of the First Affiliated Hospital Fujian Medical University Fuzhou China; ^3^ School of Stomatology Fujian Medical University Fuzhou China; ^4^ Fujian Key Laboratory of Oral Diseases, School and Hospital of Stomatology Fujian Medical University Fuzhou China

**Keywords:** adaptive immunity, immune infiltration, peri‐implantitis, periodontitis, RNA‐Seq

## Abstract

**Background:**

Peri‐implantitis and periodontitis have similar immunological bioprocesses and inflammatory phenotypes. In the inflammatory process, the adaptive immune cells can drive the development of disease. This research investigated the differences and diagnostic significance of peri‐implantitis and periodontitis in adaptive immune responses.

**Methods:**

We acquired four GEO datasets of gene expressions in surrounding tissues in healthy person, healthy implant, periodontitis, and peri‐implantitis patients. The structural characteristics and enrichment analyses of differential expression genes were examined. The adaptive immune landscapes in peri‐implantitis and periodontitis were then evaluated using single sample gene set enrichment analysis. The STRING database and Cytoscape were used to identify adaptive hub genes, and the ROC curve was used to verify them. Finally, qRT‐PCR method was used to verify the expression level of Hub gene in activated T cells on the titanium‐containing or titanium‐free culture plates.

**Results:**

At the transcriptome level, the data of healthy implant, peri‐implantitis and periodontitis were highly dissimilar. The peri‐implantitis and periodontitis both exhibited adaptive immune response. Except for the activated CD4^+^T cells, there was no significant difference in other adaptive immune cells between peri‐implantitis and periodontitis. In addition, correlation analysis showed that *CD53*, *CYBB*, and *PLEK* were significantly positively linked with activated CD4^+^T cells in the immune microenvironment of peri‐implantitis, making them effective biomarkers to differentiate it from periodontitis.

**Conclusions:**

Peri‐implantitis has a uniquely immunogenomic landscape that differs from periodontitis. This study provides new insights and ideas into the activated CD4^+^T cells and hub genes that underpin the immunological bioprocess of peri‐implantitis.

## Graphical abstract

The pathogenesis and etiology of peri‐implantitis are unknown. The periodontal bacteria induce peri‐implantitis, but the host's immune response determines the course and severity of peri‐implantitis in the future. Given the similarities and differences in the immunological processes of peri‐implantitis and periodontitis, we used bioinformatics tools to analyze and compare the differences and characteristics of transcriptome data among samples of peri‐implantitis, periodontitis, healthy implants, and healthy people, from the GEO database. Our purpose is to construct a unique landscape of adaptive immune cells, hub genes, and immunological factors linked to peri‐implantitis, therefore providing new ideas for the immunological mechanism of peri‐implantitis. Further, We believe that our study makes a significant contribution to the literature because these characteristics may be valuable as biomarkers for evaluating and treating peri‐implantitis.

## 1 INTRODUCTION

Peri‐implantitis (PI), an inflammatory disease characterized by soft tissue infection and bone resorption, severely affects implant longevity and is the main cause of implant failure.^1^, ^2^ According to statistics, about 30% of implant patients suffered from PI.^3^ However, PI has no effective diagnosis or therapy since its pathophysiology is not fully understood.^4^ Thus, it is necessary to further consider the pathogenic mechanism of PI to provide new ideas for effective diagnosis and treatment.

Similarly, periodontitis (P) is a biofilm‐induced chronic inflammatory disease. The tooth‐associated biofilm or dental plaque is essential but not sufficient to induce periodontitis, and the inflammatory response induced by the resistance of the host to these microbes is the key to the destruction of the periodontium.^5^ PI and periodontitis (P) have many similarities in etiology, pathogenesis, diagnosis and treatment.^6^, ^7^ Thus, in the past, studies on the molecular mechanisms of peri‐implantitis and periodontitis had sought to reveal some important differences in histopathology and immunology.^8^, ^9^ Both diseases are opportunistic infections, but histopathology showed that PI has higher inflammatory regions.^8^, ^9^ Even though the types of immune cells in both were similar, some studies had shown that the degree of immune infiltration was different, with PI having more plasma cells and lymphocytes.^2^ When bone resorption is caused by peri‐implant infection, T and B cells are highly enriched in the inflammatory region, and the gene expression levels of transcription factors *RORγt* (Th17) and *FoxP3*(Treg) were significantly higher than in healthy tissues,^10^ indicating that T cell subsets might dominate disease's development in the late stage of PI. We speculate that the the adaptive immune response in PI may differ from periodontitis, which aids in the development of effective‐targeted treatment strategies.

In recent years, bioinformatics analysis of transcriptomes had revealed the molecular processes of complicated disease. Previous studies on the pathogenesis differences between PI and P mainly focused on differentially expressed lncRNAs and mRNAs,^11^, ^12^, ^13^ and only one study based on weighted gene co‐expression network analysis of public databases preliminarily showed that PI‐related gene networks were rich in adaptive immune responses.^14^

Due to the importance of adaptive immune response and the lack of studies on it in PI, this study used bioinformatics to compare the adaptive immune responses of PI, P, and healthy implant (HI) to obtain the transcriptional genes with significant differences and immune cells that chemotaxis in PI. Subsequently, to uncover hub genes and immunological factors regulating this immunobiological process, a protein‐protein interaction network of genes associated to key adaptive immune cells was constructed. We created a unique landscape of adaptive immune cells, hub genes, and immunological factors connected to peri‐implantitis, providing new ideas for the immune mechanism of PI.

## 2 MATERIALS AND METHODS

### 2.1 Data set selection and preprocessing

The GEO (https://www.ncbi.nlm.nih.gov/geo/) database provided gene expression data for HI, PI, P, and healthy person (HP) in the surrounding tissue. HP served as a control group. For HI and PI, healthy or inflammatory peri‐implant soft and hard tissues were taken, as well as healthy or inflammatory gingivae for HP and P. This research used four datasets: GSE106090, GSE178351, GSE33774, and GSE57631. The datasets were annotated and normalized, and when many probes were mapped to the same gene symbol, gene expression was calculated using the average value. GSE106090, GSE57631, and GSE178351 will be combined as training datasets, with GSE33774 as validation. To eliminate the batch effect, the sva R package's CombAt method was utilized.

### 2.2 Screening and analysis of differential expression genes (DEGs)

We performed principal component analysis (PCA) on the transcriptome data of HP, HI, PI, and P, first using stats to z‐score the expression profile and then using the prcomp function to reduce dimensionality. The limma R package screened DEGs between HI/PI/P and HP. |logFC | >1 and an adjusted *p* < .05 indicated significant differences. The Venn diagram was created using the R package ggplot2.

### 2.3 Gene ontology (GO) and the kyoto encyclopedia of genes and genomes (KEGG) pathway enrichment analysis

Using clusterProfiler R package for GO and KEGG (www.kegg.jp/kegg/kegg1.html) analysis of DEGs. GO analysis includes biological processes, molecular functions and cellular components. KEGG analysis is an important way to identify significant pathways of gene enrichment.

### 2.4 Evaluation of adaptive immune cell infiltration

We downloaded the GSEA (v4.2.3) and pre‐designed the gene sets of interest, namely the HI, PI, and P DEGs sets. The list of DEGs between HI/PI/P and HP was inputted into the software 1000 times. When *p* < .05 and *FDR* < 0.25, the function was significantly enriched. The 14 different types of adaptive immune cell infiltrations were evaluated using the expression levels of immune cell‐specific marker genes. The gsva R package was used to perform single sample gene set enrichment analysis (ssGSEA), and the pheatmap R software visualized the findings. The TISIDB database (http://cis.hku.hk/TISIDB/index.php) collected marker genes with 400 genotypes. The Pearson algorithm was utilized to assess the variations in adaptive immune cell infiltrations and correlations among groups, and the results were shown using ggplot2 R package.

### 2.5 Constructing a protein‐protein interaction (PPI) network and identifying adaptive immunity‐related hub genes

The screened DEGs overlapped with 400 adaptive immune‐related genes (IRGs). The PPI network of IRGs was based on the STRING (http://string-db.org) database. The required minimum interaction score was set at moderate confidence (0.4). The top 10 hub genes were screened using the MCC, MNC, Degree, and EPC algorithms of the CytoHubba plugin in Cytoscape. Finally, the overlapping hub genes of the 4 algorithms were screened out.

### 2.6 Construction of activated CD4^+^T cell‐related interaction network by hub genes and immune factors

TISIDB database (http://cis.hku.hk/TISIDB/) provided immune factors. There were 24 immunosuppressive factors, 46 immunostimulatory factors, 41 chemokines, 21 major histocompatibility complex (MHC) and 18 receptors. To identify hub genes and immune factors related to activated CD4^+^T cell, the Pearson algorithm was utilized. Then, using the string database (http://string-db.org), the PPI network of hub genes and immune factors related to activated CD4^+^T cell was constructed. The Cytosscape (v3.9.1) was used for visualization.

### 2.7 Correlation analysis and validation of diagnostic markers and adaptive immune cells

The connection between hub genes and 14 adaptive immune cells were estimated using Pearson analysis and shown using the ggplot2 R package. Receiver operating characteristic (ROC) analysis was utilized to evaluate the diagnostic value of hub genes for separating PI and P. The pROC R package performed the ROC analysis.

### 2.8 Extraction, activation and culture of lymphocytes

The Experimental Animal Ethics Review Committee of Fujian Medical University has given their clearance for this work (project approval number: IACUC FJMU 2022‐0440). SPF‐level male Sprague‐Dawley rats (6–8 weeks, 200–220 g) were provided by the SLAC Experimental Animal Co., Ltd., Shanghai, China. The rats were housed in dedicated cages under controlled conditions (temperature: 22°C ± 2°C; relative humidity: 50% ± 6%) with the light/dark cycle (on 07:00 AM, off 19:00 PM). The rats were conventionally euthanized by the intraperitoneal injection of an overdose of pentobarbital sodium (100 mg/kg). After 2–3 min, the criteria for sacrifice were that rats did not have spontaneous breath and blink reflexes. Then, spleen tissues were removed and segregated aseptically. We placed them on 70 μm‐filter and ground while adding 4°C‐DHANKs solution (Solarbio Life Sciences). The ground‐up spleen cell suspension was gathered. With the help of a lymphocyte separation solution, the spleen cell suspension was divided into four layers. A T25 cell culture bottle (Sorfa) was used to cultivate the lymphocytes from the second layer. A 12‐well plate was coated with the final concentration of Monoclonal Antibody CD3 (final concentration 1 ug/mL) (ThermoFisher Scientific) and put in the refrigerator at 4°C for 24 h. At a concentration of 4 × 10^6^ cells/mL, lymphocytes were injected into 12‐well plates with or without pure titanium sheet (Northwest Institute of Non‐Ferrous Metals, Xi'an). To activate the T cell population, monoclonal antibody CD28 (ThermoFisher Scientific), at a final concentration of 5 μg/mL, was added. After 3 days of cultivation, cells were harvested.

### 2.9 Quantitative real‐time polymerase chain reaction

Total RNA was extracted by means of RNAiso Plus (Takara), and reverse transcription was performed with the PrimeScript™RT reagent Kit (Takara). Quantitative real‐time polymerase chain reaction (RT‐qPCR) was performed on a LightCycler 480 (Roche) with TB Green Premix Ex Taq (Takara). The data were subjected to the comparative cycle threshold method (ΔΔCt) and normalized to a housekeeping gene, *Gapdh*. The following primers were used: *Gapdh*, Forward 5ʹ‐ACGGCAAGTTCAACGGCACAG‐3ʹ and Reverse 5ʹ‐ GAAGACGCCAGTAGACTCCACGAC ‐3ʹ (NM_017008.4); *Cybb*, Forward 5ʹ‐GTTTGCCGGAAACCCTCCTA‐3ʹ and Reverse 5ʹ‐GTGCGTGACCACCTTAGTGA‐3ʹ (NM_023965.2); *Plek*, Forward 5ʹ‐TGTGTGGTGACTTCCGTAGAGAG‐3ʹ and Reverse 5ʹ‐ATGAACTTCGTCTGCTGTGATGATC‐3ʹ (NM_001025750.1); *CD53*, Forward 5ʹ‐TTCTGCTGCTGCTTATTCTCCTTG‐3ʹ and Reverse 5ʹ‐TGGTGCTGTTGTCAGAGTGATAAT‐3ʹ (NM_012523.2).

### 2.10 Statistical analysis

SPSS (v23.0), R software (v4.1.3), and Cytoscape software (v3.9.1) handled and analyzed the data. The independent *t* test or Mann‐whitney U‐test compared continuous variables between the two groups. The one‐way anova or Kruskal‐wallis test compared continuous variables among the three groups. Statistical significance was defined as *p* < .05.

## 3 RESULTS

### 3.1 Datasets preprocessing

**Figure 1:**
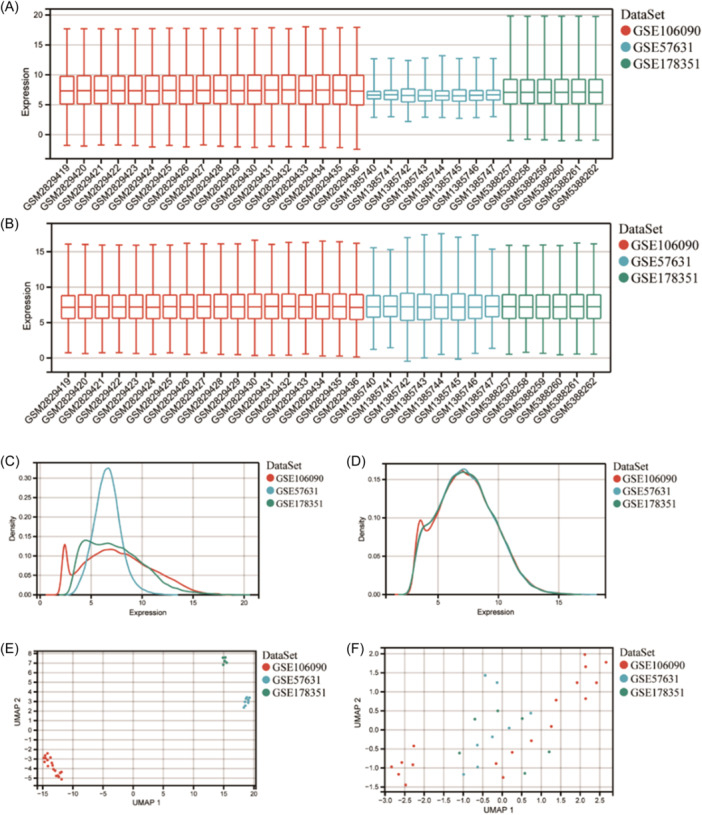
GSE106090, GSE57631 and GSE178351 datasets merged and the distribution differences before and after removing batch effects. (A, B) Boxplots; (C, D) Density distribution; (E, F) Umap distribution.

The GSE106090, GSE178351, and GSE57631 were combined into a training set. After removing batch effects, data distributions converged, showing that batch‐to‐batch variability had been eliminated (Figure 1).

### 3.2 PCA and DEGs screening

**Figure 2:**
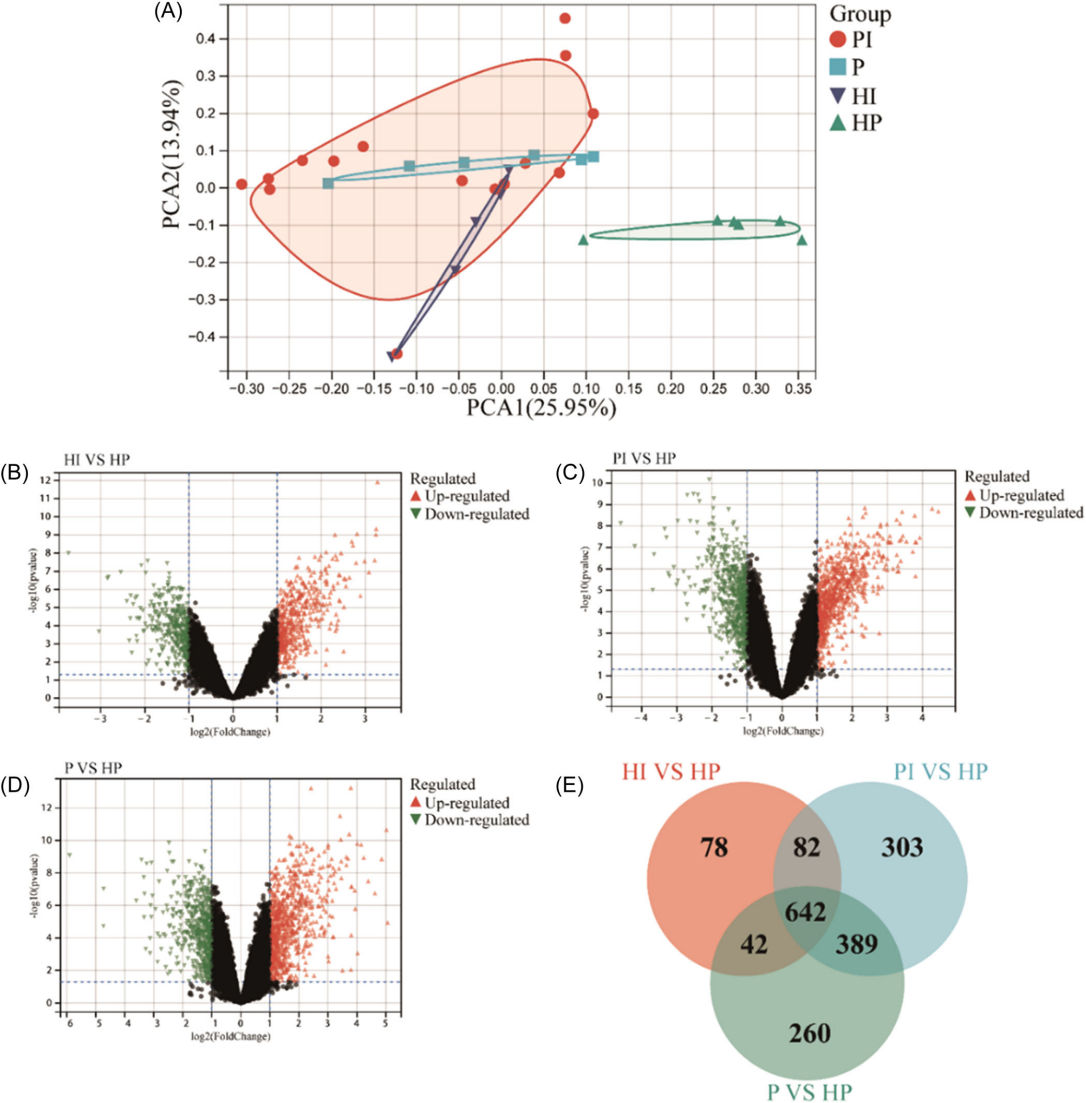
Comprehensive analysis of differentially expressed genes (DEGs) in sample tissues of healthy implant/peri‐implantitis/periodontitis versus healthy people. (A) Principal component analysis; (B–D) Volcano map in healthy implant/peri‐implantitis/periodontitis versus healthy people. Red dots represent significantly upregulated genes, green dots represent significantly downregulated genes, and black dots represent nonsignificantly DEGs; (E) Venn diagram.

First, through the PCA, we found the PI, P, and HI samples were significantly separated from the HP group, indicating that the DEGs in the HI/PI/P groups were truly significantly different from HP (Figure 2A). The range of PI and P samples, on the other hand, basically overlapped and PI partially overlapped with HI, showing that the enriched genes of PI and P were substantially comparable, followed by HI.

Figure 2B–D demonstrated that when HI/PI/P were compared to HP, there were 470, 759, 756 upregulated and 374, 657, 577 downregulated DEGs, respectively. Furthermore, the Venn diagram revealed that 642 DEGs coexisted in the three oral states, with HI, PI, and P having 78, 303, and 260 unique DEGs, respectively (Figure 2E), demonstrating that peri‐implant and periodontal tissue genotypes were both distinct and similar in the three oral states.

### 3.3 Functional enrichment analysis of DEGs

**Figure 3:**
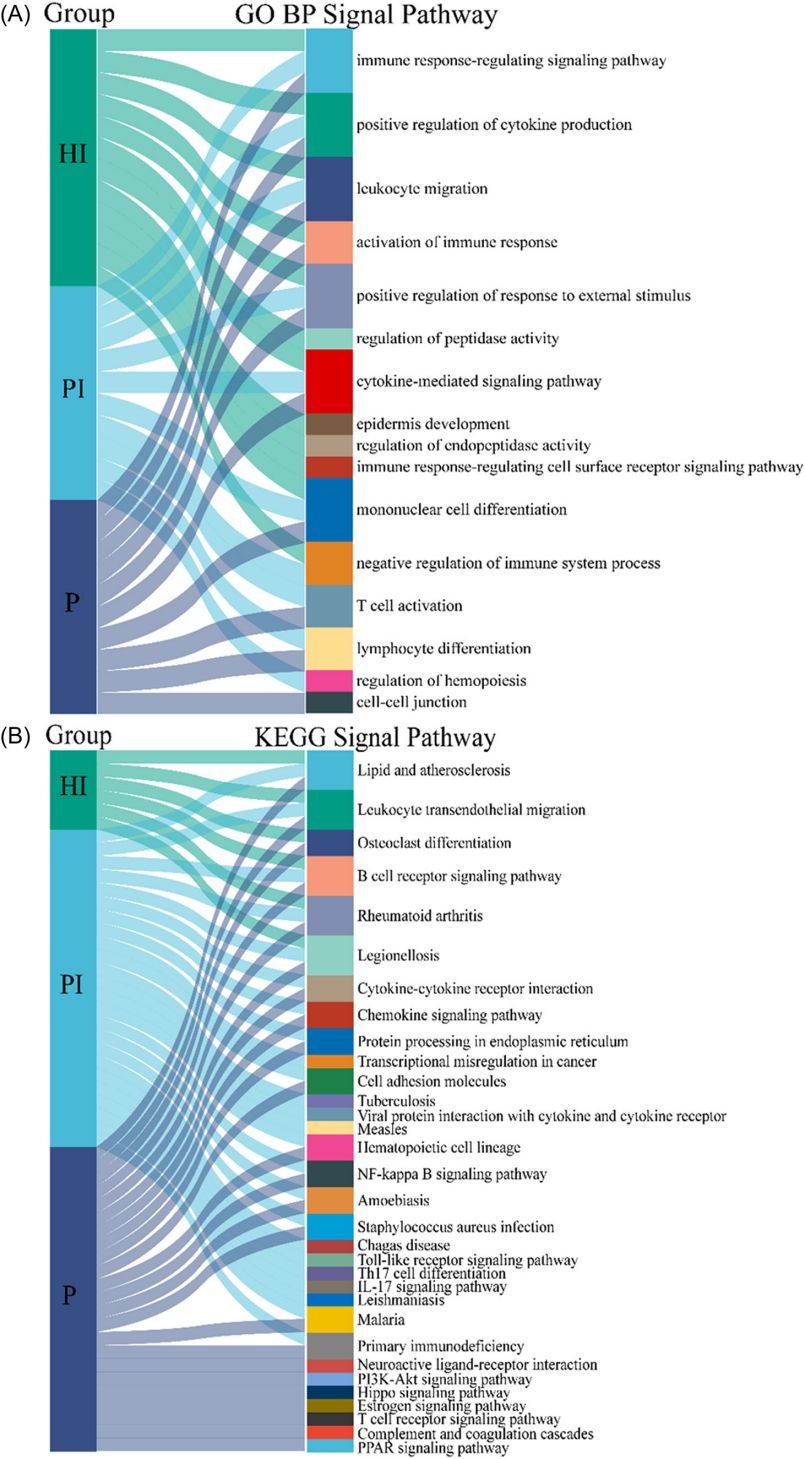
Enrichment analyses were used to determine the key pathways of the DEGs. (A) The Gene Ontology (GO) enrichments and (B) the Kyoto Encyclopedia of Genes and Genomes (KEGG) pathways in healthy implant/peri‐implantitis/periodontitis versus healthy people.

We used GO and KEGG enrichment analysis on the DEGs to investigate the probable associated molecular processes of HI, PI, and P. The top 10 GO analysis findings with the greatest count in each group were shown in Figure 3A. They were mostly engaged in immunological and inflammatory responses. Interestingly, both PI and P DEGs were concentrated in innate and adaptive immunity processes such as mononuclear cell differentiation, T cell activation, and lymphocyte differentiation. Supplementary Tables [Link], [Link], and 5 showed the details of the GO enrichment study.

According to KEGG enrichment analysis (Figure 3B), DEGs enrichment pathways of PI and P were very similar, mainly concentrated in bacterial infection, cell signal transduction, cell adhesion and migration, NF‐kappa B signaling pathway, and the DEGs‐specific enrichment pathways of PI were mainly Th17 cell differentiation, IL‐17 signaling pathway, Toll‐like receptor signaling pathway. DEGs‐specific enrichment pathways of P involved in T cell receptor signaling pathway, etc. Above all, GO enrichments and KEGG data showed that, DEGs of PI and P were mostly enriched in adaptive immunity‐mediated inflammatory activation, particularly in T cell activation and differentiation pathways, which might be important in the pathogenesis of PI. Supplementary Tables [Link], [Link], and 6 showed the details of the KEGG enrichment study.

### 3.4 Adaptive immune cell infiltration

**Figure 4:**
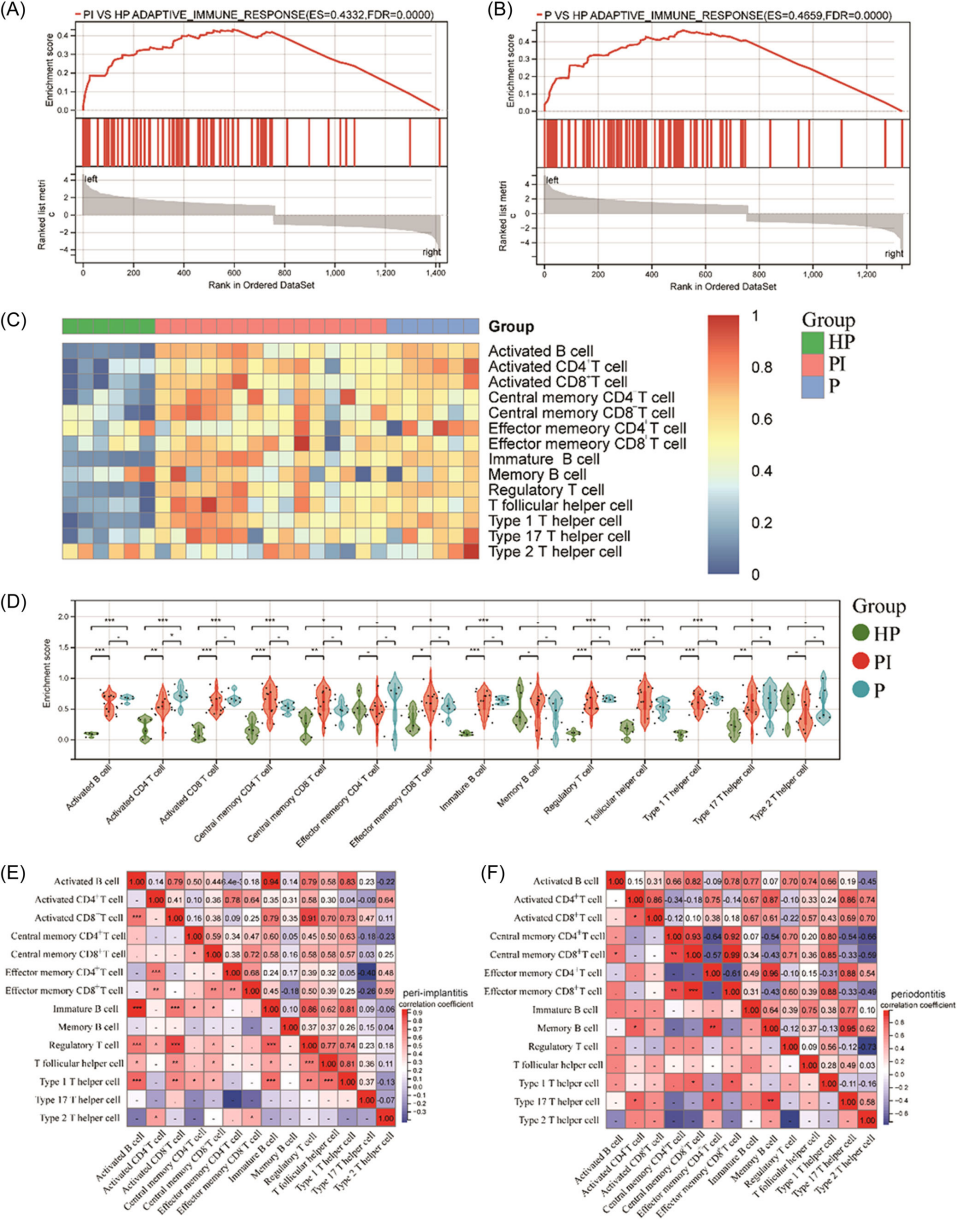
Comprehensive analysis of adaptive immune cells in peri‐implantitis/periodontitis versus healthy people. (A, B) GSEA enrichment analysis; (C) Heatmap of the landscape of adaptive immune cells infiltration; (D) Violin diagram of the comparision in adaptive immune cells. *: *p* < .05, **: *p* < .01, ***: *p* < .001, ‐: No significant difference; (E–F) Correlation heatmap of adaptive immune cells in peri‐implantitis and periodontitis. The colors of the squares represent the strength of the correlation; red represents a positive correlation, blue represents a negative correlation. The darker the color, the stronger the correlation.

To further investigate adaptive immune responses in PI and P. On both sets of DEGs, we ran GSEA. The results indicated that PI and P's *FDA* = 0, were considerably smaller than 0.25, showing that these disease states had a high degree of adaptive immune response enrichment (Figure 4A,B). The ssGSEA technique was then utilized to examine the adaptive immune cell infiltration landscapes of PI and P. The heatmap results revealed that the majority of the adaptive immune cell infiltration in HP was low abundance, and PI/P was highly similar in immune cell distributions with high abundance (Figure 4C). In PI, only activated CD4^+^T cells revealed a change, with reduced infiltration compared to P (Figure 4D).

According to a Pearson analysis, activated CD4^+^T cells in PI were substantially positively linked with Treg, Th2, effector memory CD4^+^ and CD8^+^T cell (Figure 4E). Activated CD4^+^ T cells in P were strongly positively connected with activated CD8^+^ T cells, memory B cells, and type 17 T helper cells (Figure 4F). This suggests that activated CD4^+^T cell were the major body, and other immune cells interact with it in various degrees in forward directions, generating distinct immunocyte signatures in PI.

### 3.5 PPI network analysis and identification of hub genes

**Figure 5:**
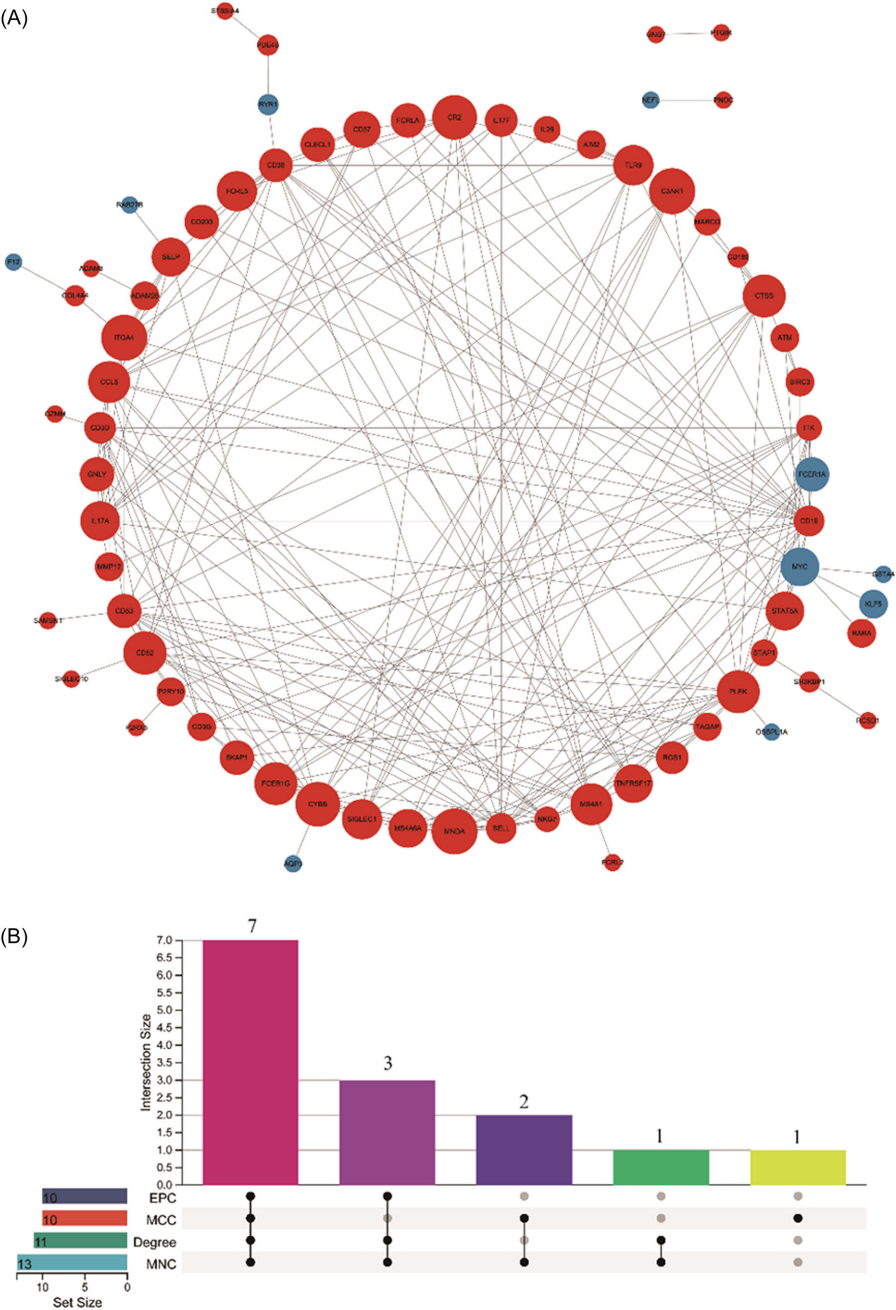
The protein‐protein interaction (PPI) network of adaptive immune‐related genes (IRGs) in peri‐implantitis. (A) PPI network for 84 IRGs, each round node represents a gene; (B) Upset Venn diagram of the top 10 hub genes. Each black dot represents the different algorithm. The figure represents the number of hub genes overlapped by different algorithms.

We found that PI DEGs were enriched for adaptive immune response activities and showed unique immunocyte signatures compared to P. We intersected the screened DEGs with 14 adaptive immune cell‐related marker genes to generate 84 hub genes (Supplementary Table 7), 67 of which were upregulated and 17 downregulated. We then created a PPI network with 84 IRGs to identify IRGs involved in adaptive immunity in PI (Figure 5A). The cytoHubba plugin's MCC, MNC, Degree, and EPC algorithms screened the top 10 hub genes (Figure 5B), and selected 7 overlapping hub genes: *CD19*, *CD38*, *SELL*, *IL17A*, *CD53*, *PLEK* and *CYBB* (Table 1).

**Table 1:** The distribution of top 10 hub genes in peri‐implantitis with four algorithms.

Catalog	Rank methods in cytoHubba							
	MCC		MNC		Degree		EPC	
	Gene	Score	Gene	Score	Gene	Score	Gene	Score
Gene top 10	***CD19***	765	***CD19***	23	***CD19***	24	***CD19***	24.984
	***CD38***	631	***SELL***	19	***SELL***	19	***SELL***	24.345
	***SELL***	626	***CD38***	16	***CD38***	17	***CD38***	23.26
	***IL17A***	482	***PLEK***	16	***PLEK***	17	***CD53***	23.132
	***CD53***	371	***CD53***	15	***CD53***	16	***PLEK***	22.411
	*CR2*	366	*CCL5*	15	*CCL5*	15	*CCL5*	21.896
	***PLEK***	353	***IL17A***	14	***IL17A***	14	*CD52*	21.853
	*FCER1G*	308	*CR2*	12	*MS4A1*	14	***IL17A***	21.774
	***CYBB***	303	*FCER1G*	12	***CYBB***	13	***CYBB***	21.719
	*TLR9*	298	***CYBB***	12	*CD52*	13	*CD3D*	21.432
			*MS4A1*	12	*CD3D*	13		
			*CD52*	12				
			*CD3D*	12				

*Note*: The bolded genes represent the overlapping hub genes that were ranked in the top 10 by 4 different approaches in cytoHubba.

Abbreviations: EPC, edge percolated component; Degree, degree of connection between nodes; MCC, maximum clique centrality; MNC, maximum neighborhood component.

### 3.6 Construction of activated CD4^+^T cell‐related interaction network between hub genes and immune factors

**Figure 6:**
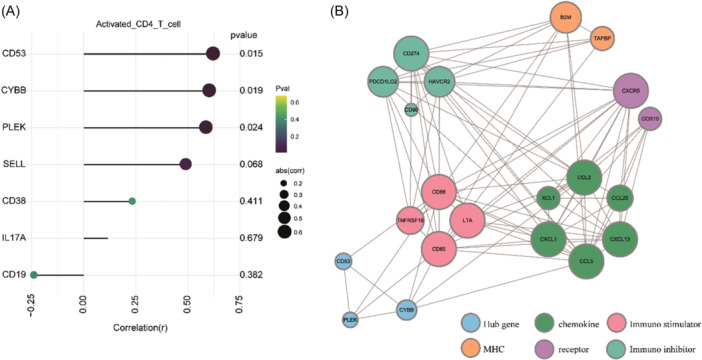
Network of hub genes and immune factors associated with activated CD4^+^T cells. (A) Correlation analysis of 7 hub genes with activated CD4^+^T cells in peri‐implantitis. (B) Interaction network diagram of 18 immune factors and 3 hub genes.

Only activated CD4^+^T cells showed significant PI changes in adaptive immune cell infiltration, so we used Pearson algorithm to analyze the link between activated CD4^+^T cells and hub genes, immune factors. The results showed that *CD53*, *CYBB* and *PLEK* in hub genes were significantly positively correlated with activated CD4^+^T cells in PI, while *SELL*, *CD38*, *IL17A* and *CD19* did not exhibit a significant correlation (Figure 6A, Supplementary Table 8).

A total of 20 immune factors, including 6 chemokines, 4 immunostimulatory factors, 6 immunosuppressive factors, 2 receptors and 2 MHC, were associated with activated CD4^+^T cells (Supplementary Table 9). After removing the 2 immunosuppressive factors that did not interact, we constructed a network of immune factors associated with activated CD4^+^T cells (Figure 6B). We found that the hub genes *CD53* and immune activator CD86 interact directly, as well as *PLEK* and *CYBB* with costimulatory molecules CD86/CD80. And only *CYBB* interacted with the chemokines CCL2 and CCL5.

### 3.7 Diagnostic values of CD53, CYBB and PLEK in PI

**Figure 7:**
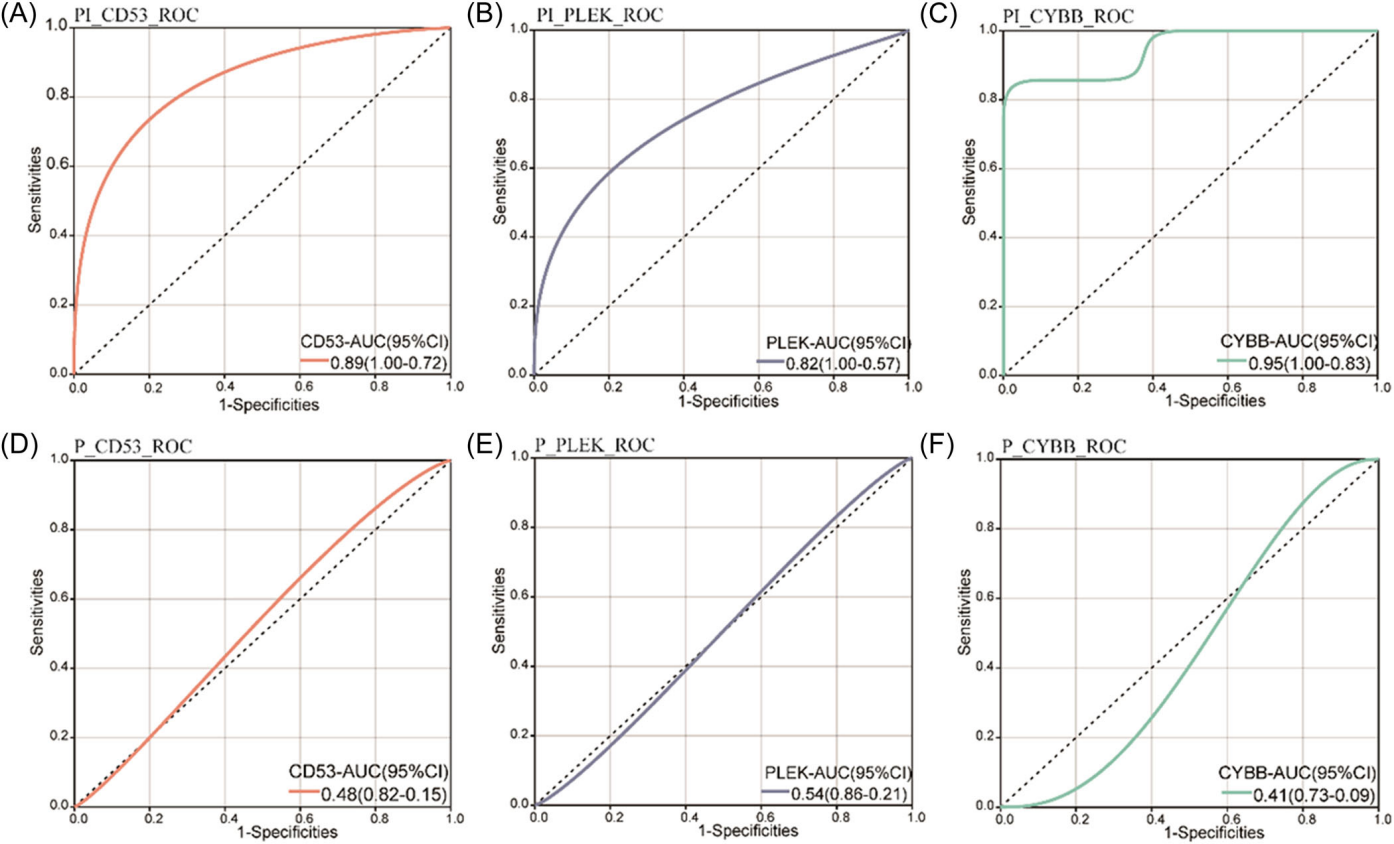
Diagnostic values of *CD53*, *PLEK* and *CYBB* in peri‐implantitis and periodontitis. (A, D) Diagnostic value of *CD53*, (B, E) *PLEK* and (C, F) *CYBB* in peri‐implantitis and periodontitis, respectively.

We used the GSE33774 data set to obtain *CD53*, *CYBB* and *PLEK* expression levels and construct the ROC curve to validate their diagnostic values in PI. With HP as the control, the area under the curve values of these genes in PI were all more than 0.80, whereas in P were less than 0.7 (Figure 7). As a result, this data set confirmed that *CD53*, *CYBB* and *PLEK* had high diagnostic performance in PI, might regulate the illness process, and that they were used as potential diagnostic markers for the disease.

### 3.8 Experimental verification of CD53, CYBB and PLEK

**Figure 8:**
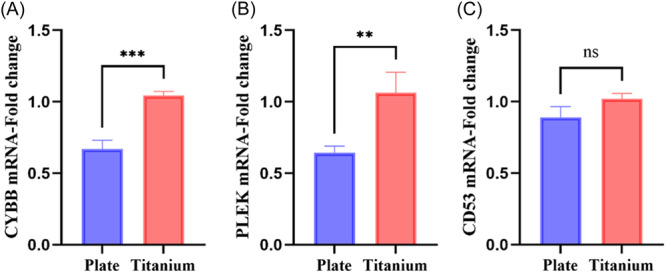
Activated T cells were cultured on the surface of culture plate and pure titanium plate. Comparison of gene expression levels of (A) CYBB, (B) PLEK, and (C) CD53. *: *p* < .05, **: *p* < .01, ***: *p* < .001, ns: No significant difference.

In the case of inflammation, lymphocytes are activated, and we activate T cells and cultivate them in an environment with or without titanium tablets to roughly replicate peri‐implantitis and periodontitis. We discovered that the expression of *CYBB* and *PLEK* was considerably higher in cells cultivated on a pure Ti plate than on the culture plate without Ti (*p* < .05) (Figure 8A,B). Although there was no significant difference in *CD53* between the two groups, the trend was consistent with the results of bioinformatics analysis (*p* > .05) (Figure 8C). This suggests that these three genes may play an important role in peri‐implantitis.

## 4 DISCUSSION

Although periodontal bacteria are the main cause of PI, the immune response of the host determines the future process and severity of the disease.^15^ When periodontal ligament and sharpey's fibers are lost, there is a more extensive inflammatory infiltration and a higher immune response in the tissue around the implant. This makes getting trustworthy clinical samples harder, which makes more difficult to consider the pathophysiological mechanism of PI. Fortunately, as high‐throughput sequencing technology has advanced, RNA‐Seq can now perform genome‐wide transcriptome analysis of any disease or species to offer a complete transcriptional gene profile.^16^ This method definitely has a benefit in increasing comprehension of the underlying mechanisms of complex diseases. In this study, based on the bioinformatics analysis of public data sets, we simultaneously revealed the similarities and differences of immunological microenvironment in PI, P and HI at the transcriptional spectrum level for the first time.

Usually, once the implant is inserted into the bone tissue as a foreign body, the tissue damage caused by it will lead to the release of damage‐associated molecular patterns and the activation of the host immune response, resulting in chemotaxis, or the movement and activation of immune cells.^17^ Therefore, inflammation seems to be a symptom of the healing process of the mucosa around the implant. A previous study showed inflammatory cells (macrophages, polymorphonuclear leukocytes and lymphocytes) in specimens at different stages of healing following implant insertion. In addition, the study showed that, although inflammatory cells reduced steadily as healing time increased, they did not entirely vanish.^18^ Unlike in HI, adaptive immune cells were more prevalent in P and PI, and were frequently directly involved in pathological changes in patients' tissues, especially T and B lymphocytes.^19^, ^20^ In this study, we obtained similar results. Through the enrichment analysis of their DEGs functions, the immune system function of HI was mainly focused in the innate immune response, while PI and P not only enriched the innate immune response but also greatly enriched the adaptive immunological processes. Especially, PI had been related to T cell activation and Th17 cell differentiation. In addition, our results showed a significant degree of similarity between PI and P in adaptive immune cell types,^21^, ^22^ which might explain why the two diseases had similar inflammatory manifestations.

We still discovered a significant difference in activated CD4^+^T cell between the two diseases in adaptive immune cells. It has been proposed that the mechanisms of PI and P subsets differentiation after activated CD4^+^T cells may be different. Usually, CD4^+^T cells are activated to differentiate into cell subsets with different functions under the action of various immunologic cytokines and environment. These cell subsets then regulated adaptive immune response and the processes of inflammatory by releasing different immunologic cytokines.^23^, ^24^ Some previous studies had shown that recognized CD4^+^T cell subsets such as Th1, Th2, Treg, and Th17 played an important role in PI and P, and one study found that Th1/Th2 cells infiltrate similarly in the gingival tissue of these two diseases.^25^ Other subsets have not been reported. In this study, we also found the same trend. Figure 4D showed that the infiltration of Th1, Th2, Treg, Th17, and Tfh in the tissues surrounding the peri‐implantitis is similar to that of periodontitis. Therefore, we speculate that the differences in immunological infiltration of activated CD4^+^T cells in peri‐implantitis and periodontitis may be attributed to other activated CD4^+^T subsets that were not labeled from the TISIDB database, such as Th9 and Th22.^26^, ^27^

T cell activation was accompanied by changes in transcriptional and proteomic mechanisms that promoted the growth, proliferation, and differentiation of T cells.^28^ Therefore, to comprehend the critical molecules related to activated CD4^+^T cell in PI. We then constructed the PPI network through the IRGs in PI and identified the key genes in the network. Through correlation analysis, *CD53*, *CYBB* and *PLEK* were highly linked with activated CD4^+^T cell. *CD53*, also known as *OX44* or *TSPAN25*, is a Tetraspan superfamily member that expresses in the immunological cavity. Previous studies had shown that it might affect the adhesion and migration of immune cells, as well as signal transmission through the interaction of a variety of membrane proteins and intracellular signal molecules, influencing T and B cell activation.^29^, ^30^, ^31^ *CYBB* is a family of inflammation‐related genes, and gp91phox (*NOX2* or *CYBB*) is the complex's major redox center, responsible for electron transferase activity.^32^ According to research, the gp91phox is involved in the formation of cytochrome b‐245. NADPH oxidase requires cytochrome b‐245 as a component protein. Cytokines with NADPH oxidase activity have been found to regulate the homeostasis of activated T cells in a dependent way.^33^ Pleckstrin, which is encoded by the *PLEK* gene, is mostly found in the cytoplasm, although it may be phosphorylated and localized to intracellular binding sites.^34^ It had been discovered in lymphocytes, monocytes, and granulocytes, and it primarily influenced cytoskeletal remodeling and cell movement capability.^34^, ^35^, ^36^, ^37^ More studies had linked it to T cell activation and TCR diversity.^38^ Although there are few relevant literatures, available data showed that *CD53*, *CYBB* and *PLEK* were related to T cells activation and proliferation, which might provide new avenues and potential therapeutic targets for future research on the pathophysiology of PI.

Immune factors are important regulatory proteins in the progression of inflammatory diseases. This study also delineated the network of target genes and immunological factors linked with activated CD4^+^T cells. In the network, hub genes *CD53*, *CYBB* and *PLEK* interacted with immune stimulatory factors CD86 and CD80, while *CYBB* interacted with chemokines CCL2 and CCL5. Studies had confirmed that CD80 and CD86 were both potent and similar co‐stimulators of T lymphocytes. And their role in the immune response might be mainly determined by their differential expression on APC.^39^ On the other hand, chemokines CCL2 and CCL5 could recruit monocytes and T cells to promote the inflammatory process and also played a role in the proliferation and differentiation of T cells.^40^, ^41^, ^42^ Further corroborating our above results,*CD53*, *CYBB* and *PLEK* were associated with the activation and proliferation of CD4^+^T cells, thereby regulating the immunological process of PI.

This research was based on the data of transcriptional gene expression from surrounding tissues with HI, PI, P, and HP. Secondly, this was the first research to explore the differences in adaptive immunity between PI and P, helping in understanding the common pathophysiological process of the two diseases and providing new ideas for exploring the pathogenic mechanism and treatment strategy of PI. However, our research also had some limitations. Because of the disease characteristics of PI and the difficulties in acquiring samples, a large sample multicenter cohort study should be conducted in the future to verify the results. Secondly, this work was mainly based on bioinformatics analysis. Although we used different datasets and qRT‐PCR to validate the diagnostic value, further animals and cells experiments were needed to verify our findings.

## 5 CONCLUSIONS

In conclusion, the results of this study reveal that PI and P have high similarity in adaptive immune response. The activation of CD4^+^T cells can help to distinguish PI from P. The hub genes CD53, CYBB and PLEK are related to the proliferation and activation of CD4^+^T cells and may serve as biomarkers of PI.

## AUTHOR CONTRIBUTIONS

**Jingju Yin**: Data curation; methodology; software; validation; visualization; writing—original draft; writing—review and editing. **Youran Fang**: Data curation; validation. **Yunyang Liao**: Software. **Zhe Chen**: Validation. **Shaofeng Liu**: Validation. **Hanghang Zhu**: Visualization. **Kun Song**: Visualization. **Bin Shi**: Conceptualization; funding acquisition; methodology; writing—review and editing.

## CONFLICT OF INTEREST STATEMENT

The authors declare no competing interests.

## ETHICS STATEMENT

The Experimental Animal Ethics Review Committee of Fujian Medical University has given their clearance for this work (project approval number: IACUC FJMU 2022‐0440). All methods were carried out in accordance with the relevant guidelines and regulations. This study was carried out in compliance with the ARRIVE guidelines. GEO belongs to public databases. Users can download relevant data for free for research and publish relevant articles. Therefore, there are no moral problems and other conflicts of interest in the bioinformatics analysis part based on GEO data sets.

## Supporting information

Supporting information.

Supporting information.

Supporting information.

Supporting information.

Supporting information.

Supporting information.

Supporting information.

Supporting information.

Supporting information.

## Data Availability

The datasets used and/or analysed during the current study are available from the corresponding author on reasonable request.
