# Supplementary material for: Bioinformatics investigation of adaptive immune‐related genes in peri‐implantitis and periodontitis: Characteristics and diagnostic values
Source: Immun Inflamm Dis. 2024 May 23;12(5):e1272. doi: 10.1002/iid3.1272 (PMC11112631; doi:10.1002/iid3.1272)
Supplement: Supplementary file 1 — Supporting information. [file IID3-12-e1272-s004.docx]

**Supplementary Table 1. GO enrichment analysis of** **differential expression genes in HI vs HP groups**

| **ONTOLOGY** | **ID** | **Description** | **GeneRatio** | **BgRatio** | **pvalue** | **p.adjust** | **qvalue** | **geneID** | **Count** |
| --- | --- | --- | --- | --- | --- | --- | --- | --- | --- |
| BP | GO:0002764 | immune response-regulating signaling pathway | 58/761 | 468/18723 | 3.33E-14 | 1.57E-10 | 1.39E-10 | IGLL1/CD19/KLHL6/TLR9/CD79A/BTLA/BANK1/PLCG2/STAP1/CR2/CYBA/LILRB2/TLR10/FFAR2/IRAK2/HSPA1B/IRF4/PAK3/PLEKHA1/BTN1A1/TREM2/VAV1/NMI/LILRB1/IL20RB/TLR2/S100A14/LAT2/FCN1/CTLA4/PIK3CD/LGR4/LAX1/ITK/CD38/SKAP1/RIOK3/MYO1G/WNK1/FGR/CTSS/PAWR/NFKBIL1/FCRL3/CR1/KCNN4/HSPA1A/THEMIS2/RAB11FIP2/TLR3/CD226/MAP3K1/PRNP/CLEC4E/MS4A1/LTF/MNDA/PAK2 | 58 |
| BP | GO:0001819 | positive regulation of cytokine production | 44/761 | 467/18723 | 2.07E-07 | 9.82E-05 | 8.65E-05 | TLR9/POU2AF1/IL17F/PLCG2/IL26/IL21/CYBA/LILRA5/LILRB2/FFAR2/SLAMF1/LTB/HSPA1B/IRF4/FERMT1/PANX2/TREM2/HMHB1/LILRB1/CLECL1/IL20RB/TLR2/NLRC4/SEMA7A/FCN1/PIK3CD/CD200/ITK/RIOK3/RARA/CD86/FGR/XBP1/HSPA1A/AIRE/SLC7A5/PAEP/PLA2G3/TLR3/CD226/CYBB/IL17A/AGT/MNDA | 44 |
| BP | GO:0050900 | leukocyte migration | 41/761 | 369/18723 | 5.60E-09 | 6.63E-06 | 5.84E-06 | CHST2/DPEP1/SCG2/C10orf99/STAP1/RHOH/CXCR4/CXADR/FFAR2/SLAMF1/PTN/BMP5/TREM2/ITGA4/GPR183/VAV1/P2RX4/TNFSF11/SELL/RET/CXCL3/S100A14/PIK3CD/MMP9/CD200/MYO1G/WNK1/CD9/CXCL6/PF4V1/CCL13/GPR18/CXCR1/RAC1/TREM1/CXCL2/AIRE/PECAM1/CCL18/IL17A/CXCL1 | 41 |
| BP | GO:0002253 | activation of immune response | 41/761 | 375/18723 | 8.94E-09 | 8.47E-06 | 7.46E-06 | IGLL1/CD19/KLHL6/CD79A/BANK1/PLCG2/STAP1/CR2/KRT1/ZBP1/FFAR2/PAK3/PLEKHA1/BTN1A1/TREM2/VAV1/SUSD4/NLRC4/LAT2/FCN1/CTLA4/PIK3CD/FCN2/LAX1/ITK/CD38/SKAP1/MYO1G/WNK1/FGR/PAWR/FCRL3/CR1/C1QA/KCNN4/THEMIS2/CD226/PRNP/MS4A1/MNDA/PAK2 | 41 |
| BP | GO:0032103 | positive regulation of response to external stimulus | 41/761 | 427/18723 | 3.28E-07 | 0.000129771 | 0.000114247 | TLR9/SCG2/IL17F/CD180/PLCG2/STAP1/CXCR4/IL21/CYBA/LILRA5/TLR10/ZBP1/FFAR2/SLAMF1/PTN/PAK3/TREM2/VAV1/P2RX4/TNFSF11/TIAM1/NMI/TLR2/NLRC4/PGC/S100A14/FCN1/RIOK3/KLK7/TGM2/CD1D/WNK1/VAMP8/RAC1/PLA2G3/TLR3/CD226/IL17A/AGT/MNDA/PAK2 | 41 |
| BP | GO:0052547 | regulation of peptidase activity | 41/761 | 461/18723 | 2.37E-06 | 0.000454218 | 0.000399884 | DPEP1/CCK/PPARG/CRYAB/CLDN3/SMR3B/CST7/MAPT/TP63/SERPINB5/NLRC4/SOX2/PERP/P2RX1/A2ML1/KLF4/MYC/MMP9/WFDC2/RENBP/HERPUD1/SERPINB11/ITIH2/COL4A3/SPINK7/SERPINB13/TFPI2/DNAJB6/RARRES1/CR1/ITIH3/DHCR24/PRNP/SLPI/CIDEB/LTF/SERPINA11/ANXA2/SERPINI2/AGT/PAK2 | 41 |
| BP | GO:0019221 | cytokine-mediated signaling pathway | 41/761 | 472/18723 | 4.26E-06 | 0.000696229 | 0.000612945 | TNFRSF17/EDA2R/IL17F/MPL/STAP1/KRT8/CXCR4/PPARG/LILRA5/LILRB2/ZBP1/FLT3/IRAK2/HSPA1B/TREM2/LILRA6/IFI27/TNFSF11/NMI/LILRB1/IL20RB/CXCL3/STAT5A/LILRA1/KRT18/MX1/WNK1/CXCL6/PF4V1/LILRB3/CCL13/HSPA1A/LILRB5/CXCR1/CXCL2/CCL18/IL7R/IL22RA2/IL17A/PLP2/CXCL1 | 41 |
| BP | GO:0008544 | epidermis development | 40/761 | 324/18723 | 3.94E-10 | 9.32E-07 | 8.21E-07 | KRT76/SOX21/EDA2R/ACVR1B/LIPK/KRTDAP/DSP/FOXE1/OVOL3/FERMT1/BNC1/JAG1/TFDP1/IRF6/TP63/EXPH5/ERCC3/FGFR2/KRT16/KLF4/LGR4/CRABP2/GRHL3/ESRP1/HES5/AQP3/KLK7/CALML5/FZD6/SOSTDC1/KRT5/KRT14/FLG2/SCEL/EMP1/KRT27/SPRR1A/DCT/SPRR1B/CYP27B1 | 40 |
| BP | GO:0052548 | regulation of endopeptidase activity | 40/761 | 432/18723 | 1.16E-06 | 0.000273814 | 0.00024106 | DPEP1/CCK/PPARG/CRYAB/SMR3B/CST7/MAPT/TP63/SERPINB5/NLRC4/SOX2/PERP/P2RX1/A2ML1/KLF4/MYC/MMP9/WFDC2/RENBP/HERPUD1/SERPINB11/ITIH2/COL4A3/SPINK7/SERPINB13/TFPI2/DNAJB6/RARRES1/CR1/ITIH3/DHCR24/PRNP/SLPI/CIDEB/LTF/SERPINA11/ANXA2/SERPINI2/AGT/PAK2 | 40 |
| BP | GO:0002768 | immune response-regulating cell surface receptor signaling pathway | 38/761 | 315/18723 | 2.10E-09 | 3.31E-06 | 2.91E-06 | IGLL1/CD19/KLHL6/CD79A/BTLA/BANK1/PLCG2/STAP1/CR2/LILRB2/FFAR2/PAK3/PLEKHA1/BTN1A1/VAV1/LILRB1/LAT2/FCN1/CTLA4/PIK3CD/LAX1/ITK/CD38/SKAP1/MYO1G/WNK1/FGR/PAWR/FCRL3/CR1/KCNN4/THEMIS2/CD226/MAP3K1/PRNP/MS4A1/MNDA/PAK2 | 38 |
| BP | GO:1903131 | mononuclear cell differentiation | 38/761 | 426/18723 | 5.17E-06 | 0.0007894 | 0.000694971 | CD19/TLR9/IKZF3/PCK1/CD79A/POU2AF1/PLCG2/CR2/RHOH/PPARG/IL21/LILRB2/FLT3/SLAMF1/IRF4/TREM2/ITGA4/GPR183/VAV1/LILRB1/PSMB11/CTLA4/MYC/PIK3CD/ITK/RARA/CD1D/CD86/FCRL3/CR1/GPR18/XBP1/AIRE/EOMES/ITGB8/IL7R/MS4A1/KLF6 | 38 |
| BP | GO:0002683 | negative regulation of immune system process | 37/761 | 434/18723 | 1.88E-05 | 0.001992645 | 0.001754282 | BANK1/STAP1/CR2/PPARG/LILRB2/SLAMF1/TARBP2/BMP5/GPNMB/TREM2/SUSD4/CST7/NMI/LILRB1/IL20RB/CTLA4/MYC/CD200/LAX1/RIOK3/RARA/SAMSN1/CD86/FGR/PAWR/FCRL3/CR1/LILRB3/GPR18/DLG1/TLR3/IL7R/PRNP/LTF/SOX11/PLK2/MNDA | 37 |
| BP | GO:0022407 | regulation of cell-cell adhesion | 36/761 | 448/18723 | 8.32E-05 | 0.005512937 | 0.004853473 | CHST2/PCK1/RHOH/IL21/LILRB2/SLAMF1/AKNA/JAG1/GPNMB/ITGA4/VAV1/TNFSF11/EPCAM/LILRB1/CLECL1/IL20RB/SOX2/CTLA4/KLF4/IGFBP2/LAX1/CDH1/SKAP1/RARA/CD1D/WNK1/CD9/CD86/PAWR/CR1/XBP1/DLG1/PODXL/IL7R/PRNP/HSPH1 | 36 |
| BP | GO:0070661 | leukocyte proliferation | 35/761 | 318/18723 | 9.33E-08 | 5.52E-05 | 4.86E-05 | MZB1/CD19/TLR9/IKZF3/CD79A/CD180/MPL/CR2/IL21/NPR3/OCSTAMP/LILRB2/FLT3/SLAMF1/GPNMB/TREM2/GPR183/TNFSF11/LILRB1/CLECL1/IL20RB/CTLA4/IGFBP2/CD38/CD1D/CD86/PAWR/FCRL3/CR1/DLG1/IL7R/PRNP/MS4A1/SOX11/MNDA | 35 |
| BP | GO:0050878 | regulation of body fluid levels | 35/761 | 379/18723 | 5.69E-06 | 0.000817419 | 0.000719638 | MPL/PLCG2/NPR3/CYBA/F5/KRT1/ENPP4/P2RY1/CYP4F12/VAV1/GJA1/TP63/PLEK/KRT16/P2RX1/STAT5A/GRHL3/EMP2/METAP1/AQP3/FZD6/WNK1/CD9/TFPI2/PF4V1/XBP1/KCNN4/VAMP8/FLG2/GP5/MLLT6/RAP2B/ANXA2/CLDN1/NEUROG1 | 35 |
| BP | GO:0042110 | T cell activation | 35/761 | 487/18723 | 0.00083301 | 0.029441311 | 0.025919504 | PCK1/RHOH/IL21/CXADR/LILRB2/SLAMF7/SLAMF1/IRF4/GPNMB/GPR183/VAV1/TNFSF11/LILRB1/CLECL1/IL20RB/PSMB11/CTLA4/PIK3CD/IGFBP2/LAX1/ITK/RARA/CD1D/TREML2/CD86/PAWR/CR1/GPR18/XBP1/DLG1/AIRE/EOMES/IL7R/PRNP/HSPH1 | 35 |
| BP | GO:0002429 | immune response-activating cell surface receptor signaling pathway | 34/761 | 291/18723 | 3.30E-08 | 2.23E-05 | 1.97E-05 | IGLL1/CD19/KLHL6/CD79A/BANK1/PLCG2/STAP1/CR2/FFAR2/PAK3/PLEKHA1/BTN1A1/VAV1/LAT2/FCN1/CTLA4/PIK3CD/LAX1/ITK/CD38/SKAP1/MYO1G/WNK1/FGR/PAWR/FCRL3/CR1/KCNN4/THEMIS2/CD226/PRNP/MS4A1/MNDA/PAK2 | 34 |
| BP | GO:0002757 | immune response-activating signal transduction | 34/761 | 291/18723 | 3.30E-08 | 2.23E-05 | 1.97E-05 | IGLL1/CD19/KLHL6/CD79A/BANK1/PLCG2/STAP1/CR2/FFAR2/PAK3/PLEKHA1/BTN1A1/VAV1/LAT2/FCN1/CTLA4/PIK3CD/LAX1/ITK/CD38/SKAP1/MYO1G/WNK1/FGR/PAWR/FCRL3/CR1/KCNN4/THEMIS2/CD226/PRNP/MS4A1/MNDA/PAK2 | 34 |
| BP | GO:0002697 | regulation of immune effector process | 34/761 | 339/18723 | 1.25E-06 | 0.000281741 | 0.000248039 | MZB1/TLR9/PCK1/IL17F/PLCG2/STAP1/CR2/IL21/FFAR2/SLAMF1/IRF4/TREM2/VAV1/SUSD4/LILRB1/IL20RB/SEMA7A/PGC/RARA/KLK7/CD1D/CXCL6/CD86/FGR/FCRL3/CR1/XBP1/VAMP8/SLC7A5/PLA2G3/TLR3/CD226/IL7R/IL17A | 34 |
| BP | GO:0002237 | response to molecule of bacterial origin | 34/761 | 363/18723 | 5.70E-06 | 0.000817419 | 0.000719638 | TLR9/IL24/PCK1/CD180/PLCG2/STAP1/PTGES/ANKRD1/LILRB2/NUGGC/IRAK2/MPO/TREM2/GJA1/FGFR2/LILRB1/TLR2/CXCL3/S100A14/GJB6/RARA/CXCL6/CD86/NFKBIL1/PF4V1/PTGIR/XBP1/CXCL2/SLPI/LTF/CXCL1/CLDN1/CYP27B1/CAMP | 34 |
| BP | GO:0032496 | response to lipopolysaccharide | 33/761 | 343/18723 | 4.41E-06 | 0.000696559 | 0.000613235 | IL24/PCK1/CD180/PLCG2/STAP1/PTGES/ANKRD1/LILRB2/NUGGC/IRAK2/MPO/TREM2/GJA1/FGFR2/LILRB1/TLR2/CXCL3/S100A14/GJB6/RARA/CXCL6/CD86/NFKBIL1/PF4V1/PTGIR/XBP1/CXCL2/SLPI/LTF/CXCL1/CLDN1/CYP27B1/CAMP | 33 |
| BP | GO:0030098 | lymphocyte differentiation | 33/761 | 374/18723 | 2.66E-05 | 0.002571532 | 0.002263922 | CD19/TLR9/IKZF3/PCK1/CD79A/POU2AF1/PLCG2/CR2/RHOH/IL21/LILRB2/FLT3/SLAMF1/IRF4/ITGA4/GPR183/VAV1/PSMB11/CTLA4/PIK3CD/ITK/RARA/CD1D/CD86/FCRL3/CR1/GPR18/XBP1/AIRE/EOMES/IL7R/MS4A1/KLF6 | 33 |
| BP | GO:0051346 | negative regulation of hydrolase activity | 33/761 | 379/18723 | 3.47E-05 | 0.003013415 | 0.002652947 | DPEP1/CRYAB/APOC1/SMR3B/CST7/SERPINB5/A2ML1/KLF4/MMP9/WFDC2/RENBP/HERPUD1/SERPINB11/ITIH2/WNK1/COL4A3/SPINK7/SERPINB13/TFPI2/DNAJB6/RARRES1/CR1/ITIH3/DHCR24/PRNP/SLPI/FZD10/LTF/SERPINA11/ANXA2/SERPINI2/AGT/PAK2 | 33 |
| BP | GO:0050727 | regulation of inflammatory response | 33/761 | 386/18723 | 4.98E-05 | 0.003685818 | 0.003244916 | TLR9/PLCG2/STAP1/MMP3/PPARG/PTGES/IL21/LILRA5/TLR10/KRT1/ZBP1/FFAR2/AKNA/TREM2/CST7/TNFSF11/NMI/IL20RB/TLR2/SEMA7A/NR1D2/KLF4/MMP9/CD200/TGM2/FGR/ACE2/VAMP8/PLA2G3/TLR3/IL22RA2/PLK2/AGT | 33 |
| BP | GO:0050867 | positive regulation of cell activation | 33/761 | 420/18723 | 0.000245515 | 0.013362376 | 0.011763952 | IGLL1/TLR9/PCK1/MPL/STAP1/RHOH/IL21/LILRA5/LILRB2/SPACA3/SLAMF1/CLEC4D/TREM2/GPR183/VAV1/TNFSF11/LILRB1/CLECL1/PLEK/IGFBP2/CD38/RARA/CD1D/CD86/FGR/FCRL3/CR1/XBP1/VAMP8/PLA2G3/CD226/IL7R/HSPH1 | 33 |
| BP | GO:0002443 | leukocyte mediated immunity | 33/761 | 440/18723 | 0.000561421 | 0.022725551 | 0.020007092 | IGLL1/CD19/PLCG2/STAP1/CR2/IL21/SLAMF7/PRF1/SLAMF1/JAG1/TREM2/VAV1/SUSD4/LILRB1/IL20RB/GZMB/LAT2/PIK3CD/EMP2/MYO1G/CD1D/CXCL6/FGR/CR1/C1QA/TUBB4B/VAMP8/TREM1/AIRE/PLA2G3/TLR3/CD226/IL7R | 33 |
| BP | GO:0003012 | muscle system process | 33/761 | 452/18723 | 0.000890132 | 0.030111911 | 0.026509886 | JSRP1/NPY2R/CXCR4/RCSD1/CYBA/DSP/KCNA5/MYL1/CRYAB/P2RY1/JUP/ACTA1/GJA1/P2RX4/TIAM1/PPP1R13L/P2RX1/DSC2/KLF4/GATM/MYL3/PROK2/UTS2/CD38/ADRB2/MB/STAC2/ACE2/DLG1/MYBPC2/TNNT1/AGT/NEUROG1 | 33 |
| BP | GO:0042113 | B cell activation | 32/761 | 334/18723 | 6.70E-06 | 0.000906611 | 0.000798161 | MZB1/IGLL1/CD19/TLR9/IKZF3/CD79A/POU2AF1/BANK1/CD180/PLCG2/CR2/IL21/FLT3/ITGA4/GPR183/FCRL1/LAT2/CTLA4/PIK3CD/LAX1/CD38/SAMSN1/CD86/PAWR/FCRL3/CR1/XBP1/THEMIS2/IL7R/MS4A1/KLF6/MNDA | 32 |
| BP | GO:0007159 | leukocyte cell-cell adhesion | 32/761 | 371/18723 | 5.45E-05 | 0.003967776 | 0.003493146 | CHST2/PCK1/RHOH/IL21/LILRB2/SLAMF1/GPNMB/ITGA4/VAV1/TNFSF11/SELL/LILRB1/CLECL1/IL20RB/CLEC4M/CTLA4/KLF4/IGFBP2/LAX1/SKAP1/RARA/CD1D/WNK1/CD86/PAWR/CR1/XBP1/DLG1/PECAM1/IL7R/PRNP/HSPH1 | 32 |
| BP | GO:0045785 | positive regulation of cell adhesion | 32/761 | 437/18723 | 0.001007567 | 0.032937571 | 0.028997537 | CHST2/PCK1/NPY2R/RHOH/IL21/LILRB2/SLAMF1/PTN/FERMT1/JUP/ITGA4/VAV1/TNFSF11/LILRB1/CLECL1/SOX2/RET/IGFBP2/EMP2/SKAP1/RARA/TGM2/CD1D/CD86/CR1/XBP1/PODXL/S100A10/RAC1/IQGAP1/IL7R/HSPH1 | 32 |
| BP | GO:0050673 | epithelial cell proliferation | 32/761 | 437/18723 | 0.001007567 | 0.032937571 | 0.028997537 | SCG2/TBX1/EAF2/PPARG/IL26/CYBA/PTN/BMP5/FERMT1/FGFBP1/ITGA4/IRF6/GJA1/TP63/TNFSF11/SERPINB5/EHF/FGFR2/SOX2/MYC/PIK3CD/STAT5A/LGR4/HES5/SLURP1/PROK2/COL4A3/XBP1/DLG1/SOX11/CLDN1/TNMD | 32 |
| BP | GO:0031349 | positive regulation of defense response | 31/761 | 278/18723 | 3.78E-07 | 0.000137777 | 0.000121296 | CCK/TLR9/PLCG2/STAP1/IL21/CYBA/LILRA5/TLR10/ZBP1/FFAR2/PAK3/TREM2/VAV1/GJA1/TNFSF11/NMI/TLR2/NLRC4/PGC/FCN1/RIOK3/KLK7/TGM2/CD1D/VAMP8/PLA2G3/TLR3/CD226/AGT/MNDA/PAK2 | 31 |
| BP | GO:0046651 | lymphocyte proliferation | 31/761 | 288/18723 | 8.19E-07 | 0.000242434 | 0.000213434 | MZB1/CD19/TLR9/IKZF3/CD79A/CD180/MPL/CR2/IL21/LILRB2/FLT3/SLAMF1/GPNMB/GPR183/LILRB1/CLECL1/IL20RB/CTLA4/IGFBP2/CD38/CD1D/CD86/PAWR/FCRL3/CR1/DLG1/IL7R/PRNP/MS4A1/SOX11/MNDA | 31 |
| BP | GO:0032943 | mononuclear cell proliferation | 31/761 | 291/18723 | 1.02E-06 | 0.000273814 | 0.00024106 | MZB1/CD19/TLR9/IKZF3/CD79A/CD180/MPL/CR2/IL21/LILRB2/FLT3/SLAMF1/GPNMB/GPR183/LILRB1/CLECL1/IL20RB/CTLA4/IGFBP2/CD38/CD1D/CD86/PAWR/FCRL3/CR1/DLG1/IL7R/PRNP/MS4A1/SOX11/MNDA | 31 |
| BP | GO:0006959 | humoral immune response | 31/761 | 317/18723 | 6.15E-06 | 0.000856228 | 0.000753805 | IGLL1/POU2AF1/IL17F/CR2/KRT1/RNASE6/TREM2/GPR183/SUSD4/CXCL3/PGC/FCN1/FCN2/JCHAIN/WFDC2/KLK7/CXCL6/PF4V1/CR1/CCL13/C1QA/TREM1/CXCL2/AIRE/SLPI/MS4A1/GNLY/LTF/IL17A/CXCL1/CAMP | 31 |
| BP | GO:0045861 | negative regulation of proteolysis | 31/761 | 351/18723 | 4.55E-05 | 0.003419817 | 0.003010734 | DPEP1/CRYAB/SMR3B/CST7/SERPINB5/CHAC1/A2ML1/KLF4/MMP9/WFDC2/RENBP/HERPUD1/SERPINB11/ITIH2/COL4A3/SPINK7/SERPINB13/TFPI2/DNAJB6/RARRES1/CR1/ITIH3/DHCR24/PRNP/SLPI/LTF/SERPINA11/ANXA2/SERPINI2/AGT/PAK2 | 31 |
| BP | GO:1903706 | regulation of hemopoiesis | 31/761 | 367/18723 | 0.00010413 | 0.006404665 | 0.005638531 | TLR9/IKZF3/PCK1/ACVR1B/MPL/RHOH/OCSTAMP/LILRB2/HSPA1B/PTN/IRF4/JAG1/TREM2/HCLS1/TNFSF11/LILRB1/CTLA4/MYC/RARA/MEIS1/CD86/FCRL3/CR1/LILRB3/XBP1/HSPA1A/PLA2G3/TLR3/IL7R/LTF/IL17A | 31 |
| BP | GO:0002696 | positive regulation of leukocyte activation | 31/761 | 409/18723 | 0.000685479 | 0.02576531 | 0.022683231 | IGLL1/TLR9/PCK1/MPL/STAP1/RHOH/IL21/LILRB2/SPACA3/SLAMF1/CLEC4D/TREM2/GPR183/VAV1/TNFSF11/LILRB1/CLECL1/IGFBP2/CD38/RARA/CD1D/CD86/FGR/FCRL3/CR1/XBP1/VAMP8/PLA2G3/CD226/IL7R/HSPH1 | 31 |
| BP | GO:0010951 | negative regulation of endopeptidase activity | 30/761 | 252/18723 | 1.40E-07 | 7.37E-05 | 6.49E-05 | DPEP1/CRYAB/SMR3B/CST7/SERPINB5/A2ML1/KLF4/MMP9/WFDC2/RENBP/HERPUD1/SERPINB11/ITIH2/COL4A3/SPINK7/SERPINB13/TFPI2/DNAJB6/RARRES1/CR1/ITIH3/DHCR24/PRNP/SLPI/LTF/SERPINA11/ANXA2/SERPINI2/AGT/PAK2 | 30 |
| BP | GO:0010466 | negative regulation of peptidase activity | 30/761 | 262/18723 | 3.29E-07 | 0.000129771 | 0.000114247 | DPEP1/CRYAB/SMR3B/CST7/SERPINB5/A2ML1/KLF4/MMP9/WFDC2/RENBP/HERPUD1/SERPINB11/ITIH2/COL4A3/SPINK7/SERPINB13/TFPI2/DNAJB6/RARRES1/CR1/ITIH3/DHCR24/PRNP/SLPI/LTF/SERPINA11/ANXA2/SERPINI2/AGT/PAK2 | 30 |
| BP | GO:0002831 | regulation of response to biotic stimulus | 30/761 | 327/18723 | 2.96E-05 | 0.002750678 | 0.002421638 | IL17F/CD180/PLCG2/PPARG/IL21/CYBA/ZBP1/FFAR2/TARBP2/PAK3/TREM2/VAV1/SUSD4/NMI/LILRB1/NLRC4/PGC/FCN1/RIOK3/KLK7/CD1D/CXCL6/FGR/NFKBIL1/CR1/CD226/LTF/IL17A/MNDA/PAK2 | 30 |
| BP | GO:0042742 | defense response to bacterium | 30/761 | 350/18723 | 0.00010322 | 0.006404665 | 0.005638531 | IGLL1/TLR9/C10orf99/IL17F/CYBA/RNASE6/PRB3/GSDMC/MPO/TREM2/GBP6/TLR2/NLRC4/PGC/S100A14/FCN2/JCHAIN/WFDC2/KLK7/CXCL6/FGR/TREM1/TLR3/IL7R/GBP2/SLPI/GNLY/LTF/IL17A/CAMP | 30 |
| BP | GO:0042060 | wound healing | 30/761 | 422/18723 | 0.002240244 | 0.054961361 | 0.048386813 | IL24/MPL/PLCG2/CXCR4/F5/KRT1/ENPP4/DSP/P2RY1/CLDN3/FERMT1/VAV1/KANK1/GJA1/FGFR2/PLEK/P2RX1/GRHL3/AJUBA/METAP1/FZD6/CD9/TFPI2/PF4V1/OCLN/XBP1/GP5/RAP2B/ANXA2/CLDN1 | 30 |
| BP | GO:0043588 | skin development | 29/761 | 263/18723 | 1.13E-06 | 0.000273814 | 0.00024106 | KRT76/SOX21/ACVR1B/LIPK/KRT1/DSP/LTB/FOXE1/FERMT1/JUP/JAG1/IRF6/TP63/EXPH5/FGFR2/KRT16/LGR4/GRHL3/AQP3/FZD6/SOSTDC1/DHCR24/FLG2/SCEL/KRT27/SPRR1A/SPRR1B/CLDN1/CYP27B1 | 29 |
| BP | GO:1903037 | regulation of leukocyte cell-cell adhesion | 29/761 | 336/18723 | 0.000118723 | 0.007208604 | 0.006346302 | CHST2/PCK1/RHOH/IL21/LILRB2/SLAMF1/GPNMB/ITGA4/VAV1/TNFSF11/LILRB1/CLECL1/IL20RB/CTLA4/KLF4/IGFBP2/LAX1/SKAP1/RARA/CD1D/WNK1/CD86/PAWR/CR1/XBP1/DLG1/IL7R/PRNP/HSPH1 | 29 |
| BP | GO:0060326 | cell chemotaxis | 28/761 | 310/18723 | 7.11E-05 | 0.004850813 | 0.004270552 | DPEP1/SCG2/C10orf99/STAP1/CXCR4/CXADR/FFAR2/SLAMF1/PTN/GPR183/VAV1/P2RX4/TNFSF11/TIAM1/CXCL3/S100A14/PIK3CD/WNK1/CXCL6/PF4V1/CCL13/GPR18/CXCR1/RAC1/TREM1/CXCL2/CCL18/CXCL1 | 28 |
| BP | GO:0006936 | muscle contraction | 28/761 | 347/18723 | 0.000464429 | 0.020022567 | 0.017627443 | JSRP1/NPY2R/CXCR4/RCSD1/DSP/KCNA5/MYL1/CRYAB/JUP/ACTA1/GJA1/P2RX4/PPP1R13L/P2RX1/DSC2/MYL3/PROK2/UTS2/CD38/ADRB2/MB/STAC2/ACE2/DLG1/MYBPC2/TNNT1/AGT/NEUROG1 | 28 |
| BP | GO:0030099 | myeloid cell differentiation | 28/761 | 381/18723 | 0.001922672 | 0.051737357 | 0.045548469 | ACVR1B/MPL/ZNF385A/PPARG/OCSTAMP/HSPA1B/IRF4/JAG1/TREM2/HCLS1/GPR183/TNFSF11/LILRB1/TLR2/MYC/PIK3CD/MMP9/RARA/MEIS1/LILRB3/MB/HSPA1A/PLA2G3/ITGB8/TLR3/LTF/IL17A/ANXA2 | 28 |
| BP | GO:0097529 | myeloid leukocyte migration | 26/761 | 220/18723 | 1.09E-06 | 0.000273814 | 0.00024106 | DPEP1/SCG2/STAP1/RHOH/CXADR/SLAMF1/TREM2/VAV1/P2RX4/TNFSF11/CXCL3/S100A14/PIK3CD/CD200/CD9/CXCL6/PF4V1/CCL13/CXCR1/RAC1/TREM1/CXCL2/PECAM1/CCL18/IL17A/CXCL1 | 26 |
| BP | GO:0030595 | leukocyte chemotaxis | 26/761 | 230/18723 | 2.52E-06 | 0.000459926 | 0.000404909 | DPEP1/SCG2/C10orf99/STAP1/CXCR4/CXADR/FFAR2/SLAMF1/PTN/GPR183/VAV1/TNFSF11/CXCL3/S100A14/PIK3CD/WNK1/CXCL6/PF4V1/CCL13/GPR18/CXCR1/RAC1/TREM1/CXCL2/CCL18/CXCL1 | 26 |
| BP | GO:0002699 | positive regulation of immune effector process | 26/761 | 235/18723 | 3.77E-06 | 0.000636881 | 0.000560696 | MZB1/TLR9/PCK1/IL17F/PLCG2/STAP1/IL21/FFAR2/SLAMF1/TREM2/VAV1/LILRB1/SEMA7A/PGC/RARA/KLK7/CD1D/CD86/FGR/CR1/XBP1/VAMP8/SLC7A5/PLA2G3/CD226/IL17A | 26 |
| BP | GO:0043270 | positive regulation of ion transport | 26/761 | 275/18723 | 6.00E-05 | 0.004305914 | 0.003790835 | CD19/CCK/NPY2R/PLCG2/PTGES/LILRA5/RAB3B/P2RY1/TREM2/HSPA2/P2RX4/TNFSF11/P2RX1/WNK1/ADRB2/SLC6A4/CTSS/STAC2/ACE2/DLG1/KCNN4/ATP1B3/VAMP8/PLA2G3/MLLT6/AGT | 26 |
| BP | GO:0051047 | positive regulation of secretion | 26/761 | 310/18723 | 0.00040843 | 0.01824833 | 0.016065442 | CCK/NPY2R/PTGES/ANKRD1/CYBA/P2RY1/TREM2/GJA1/EXPH5/VSNL1/P2RX4/TNFSF11/TLR2/CD38/SLC6A4/FGR/PARD6A/XBP1/KCNN4/VAMP8/RAC1/PLA2G3/TM7SF3/SOX11/RETN/AGT | 26 |
| BP | GO:0060249 | anatomical structure homeostasis | 26/761 | 314/18723 | 0.000496228 | 0.020983363 | 0.018473306 | TLR9/MC4R/CXADR/KRT1/P2RY1/CLDN3/GJA1/P2RX4/TNFSF11/IL20RB/P2RX1/VSIG1/JCHAIN/GJB6/CD38/ADRB2/OCLN/RAC1/RDH12/PECAM1/SLC2A1/LTF/ALDH1A1/IL17A/TULP1/CLDN1 | 26 |
| BP | GO:0050851 | antigen receptor-mediated signaling pathway | 25/761 | 240/18723 | 1.65E-05 | 0.001836775 | 0.001617058 | IGLL1/CD19/KLHL6/CD79A/BANK1/PLCG2/STAP1/PLEKHA1/BTN1A1/LAT2/CTLA4/PIK3CD/LAX1/ITK/CD38/SKAP1/WNK1/PAWR/FCRL3/KCNN4/THEMIS2/CD226/PRNP/MS4A1/MNDA | 25 |
| BP | GO:0070663 | regulation of leukocyte proliferation | 25/761 | 245/18723 | 2.34E-05 | 0.002312931 | 0.002036256 | MZB1/TLR9/IKZF3/MPL/IL21/OCSTAMP/LILRB2/SLAMF1/GPNMB/GPR183/LILRB1/CLECL1/IL20RB/CTLA4/IGFBP2/CD38/CD1D/CD86/PAWR/FCRL3/CR1/DLG1/PRNP/SOX11/MNDA | 25 |
| BP | GO:0050863 | regulation of T cell activation | 25/761 | 329/18723 | 0.002101661 | 0.053636743 | 0.047220648 | PCK1/RHOH/IL21/LILRB2/SLAMF1/IRF4/GPNMB/VAV1/TNFSF11/LILRB1/CLECL1/IL20RB/CTLA4/IGFBP2/LAX1/RARA/CD1D/CD86/PAWR/CR1/XBP1/DLG1/IL7R/PRNP/HSPH1 | 25 |
| BP | GO:0009612 | response to mechanical stimulus | 24/761 | 216/18723 | 8.19E-06 | 0.001021119 | 0.000898971 | CXCR4/ANKRD1/CYBA/KCNA5/PTN/P2RY1/JUP/ACTA1/MPO/GJA1/IGFBP2/SMPD2/SLC38A2/PKDREJ/STRA6/RAC1/TLR3/MMP7/MAP3K1/SLC2A1/SCEL/RETN/AGT/NEUROG1 | 24 |
| BP | GO:0050670 | regulation of lymphocyte proliferation | 24/761 | 225/18723 | 1.63E-05 | 0.001836775 | 0.001617058 | MZB1/TLR9/IKZF3/MPL/IL21/LILRB2/SLAMF1/GPNMB/GPR183/LILRB1/CLECL1/IL20RB/CTLA4/IGFBP2/CD38/CD1D/CD86/PAWR/FCRL3/CR1/DLG1/PRNP/SOX11/MNDA | 24 |
| BP | GO:0032944 | regulation of mononuclear cell proliferation | 24/761 | 227/18723 | 1.89E-05 | 0.001992645 | 0.001754282 | MZB1/TLR9/IKZF3/MPL/IL21/LILRB2/SLAMF1/GPNMB/GPR183/LILRB1/CLECL1/IL20RB/CTLA4/IGFBP2/CD38/CD1D/CD86/PAWR/FCRL3/CR1/DLG1/PRNP/SOX11/MNDA | 24 |
| BP | GO:0001894 | tissue homeostasis | 24/761 | 268/18723 | 0.000259323 | 0.013799497 | 0.012148784 | TLR9/MC4R/CXADR/KRT1/CLDN3/GJA1/P2RX4/TNFSF11/IL20RB/VSIG1/JCHAIN/GJB6/CD38/ADRB2/OCLN/RAC1/RDH12/PECAM1/SLC2A1/LTF/ALDH1A1/IL17A/TULP1/CLDN1 | 24 |
| BP | GO:1902105 | regulation of leukocyte differentiation | 24/761 | 279/18723 | 0.000467437 | 0.020022567 | 0.017627443 | TLR9/IKZF3/PCK1/RHOH/OCSTAMP/LILRB2/IRF4/TREM2/HCLS1/TNFSF11/LILRB1/CTLA4/MYC/RARA/CD86/FCRL3/CR1/LILRB3/XBP1/PLA2G3/TLR3/IL7R/LTF/IL17A | 24 |
| BP | GO:1903532 | positive regulation of secretion by cell | 24/761 | 282/18723 | 0.000545025 | 0.022642428 | 0.019933912 | CCK/NPY2R/PTGES/ANKRD1/P2RY1/TREM2/GJA1/EXPH5/VSNL1/P2RX4/TNFSF11/TLR2/CD38/SLC6A4/FGR/PARD6A/KCNN4/VAMP8/RAC1/PLA2G3/TM7SF3/SOX11/RETN/AGT | 24 |
| BP | GO:0006909 | phagocytosis | 24/761 | 308/18723 | 0.001837142 | 0.051516459 | 0.045353995 | IGLL1/PLCG2/STAP1/RHOH/CYBA/SPACA3/SLAMF1/NCF4/TREM2/VAV1/TLR2/FCN1/FCN2/RARA/TXNDC5/MYO1G/TGM2/FGR/RAB11FIP2/RAC1/XKR9/PECAM1/TULP1/MARCO | 24 |
| BP | GO:0002221 | pattern recognition receptor signaling pathway | 23/761 | 172/18723 | 5.18E-07 | 0.000175073 | 0.000154131 | TLR9/PLCG2/CYBA/TLR10/FFAR2/IRAK2/HSPA1B/IRF4/TREM2/NMI/TLR2/S100A14/FCN1/LGR4/RIOK3/CTSS/NFKBIL1/FCRL3/HSPA1A/RAB11FIP2/TLR3/CLEC4E/LTF | 23 |
| BP | GO:0045216 | cell-cell junction organization | 23/761 | 200/18723 | 7.11E-06 | 0.000935869 | 0.000823919 | CXADR/POF1B/DSP/PKP1/CLDN3/JUP/MPP7/MARVELD2/GJA1/PERP/GJB6/CDH1/CLDN10/CD9/PARD6A/OCLN/ACE2/DLG1/PECAM1/IL17A/CLDN1/AGT/PAK2 | 23 |
| BP | GO:0002274 | myeloid leukocyte activation | 23/761 | 223/18723 | 4.16E-05 | 0.003226076 | 0.002840169 | PLCG2/STAP1/RHOH/SPACA3/SLAMF1/IRF4/CLEC4D/TREM2/CST7/MAPT/NMI/TLR2/LAT2/PIK3CD/CD200/CXCL6/FGR/C1QA/VAMP8/PLA2G3/ITGB8/TLR3/CD226 | 23 |
| BP | GO:0071219 | cellular response to molecule of bacterial origin | 22/761 | 221/18723 | 0.000102651 | 0.006404665 | 0.005638531 | IL24/CD180/PLCG2/STAP1/ANKRD1/LILRB2/NUGGC/IRAK2/TREM2/LILRB1/TLR2/CXCL3/RARA/CXCL6/CD86/NFKBIL1/PF4V1/XBP1/CXCL2/LTF/CXCL1/CAMP | 22 |
| BP | GO:0071216 | cellular response to biotic stimulus | 22/761 | 246/18723 | 0.000469279 | 0.020022567 | 0.017627443 | IL24/CD180/PLCG2/STAP1/ANKRD1/LILRB2/NUGGC/IRAK2/TREM2/LILRB1/TLR2/CXCL3/RARA/CXCL6/CD86/NFKBIL1/PF4V1/XBP1/CXCL2/LTF/CXCL1/CAMP | 22 |
| BP | GO:0002366 | leukocyte activation involved in immune response | 22/761 | 275/18723 | 0.002014332 | 0.052416906 | 0.046146729 | CD19/PCK1/POU2AF1/CD180/PLCG2/IL21/SLAMF1/IRF4/TREM2/GPR183/NMI/LILRB1/LAT2/PIK3CD/RARA/CD86/FGR/CR1/XBP1/VAMP8/EOMES/PLA2G3 | 22 |
| BP | GO:0071222 | cellular response to lipopolysaccharide | 21/761 | 209/18723 | 0.000128337 | 0.007597539 | 0.006688711 | IL24/CD180/PLCG2/STAP1/ANKRD1/LILRB2/NUGGC/IRAK2/LILRB1/TLR2/CXCL3/RARA/CXCL6/CD86/NFKBIL1/PF4V1/XBP1/CXCL2/LTF/CXCL1/CAMP | 21 |
| BP | GO:2000116 | regulation of cysteine-type endopeptidase activity | 21/761 | 235/18723 | 0.000631685 | 0.024467316 | 0.021540505 | DPEP1/CCK/PPARG/CRYAB/CST7/MAPT/TP63/NLRC4/SOX2/PERP/P2RX1/KLF4/MYC/MMP9/HERPUD1/COL4A3/DNAJB6/DHCR24/CIDEB/LTF/PAK2 | 21 |
| BP | GO:0051091 | positive regulation of DNA-binding transcription factor activity | 21/761 | 260/18723 | 0.00224513 | 0.054961361 | 0.048386813 | TLR9/EDA2R/SMARCB1/PLCG2/PPARG/IRAK2/HSPA1B/JUP/TFDP1/CTH/HCLS1/TNFSF11/MID2/TLR2/NLRC4/CD200/HSPA1A/TLR3/LTF/AGT/NEUROG1 | 21 |
| BP | GO:0097530 | granulocyte migration | 20/761 | 148/18723 | 2.40E-06 | 0.000454218 | 0.000399884 | DPEP1/SCG2/RHOH/CXADR/SLAMF1/VAV1/CXCL3/S100A14/PIK3CD/CXCL6/PF4V1/CCL13/CXCR1/RAC1/TREM1/CXCL2/PECAM1/CCL18/IL17A/CXCL1 | 20 |
| BP | GO:0002833 | positive regulation of response to biotic stimulus | 20/761 | 168/18723 | 1.67E-05 | 0.001836775 | 0.001617058 | IL17F/CD180/PLCG2/IL21/CYBA/ZBP1/FFAR2/PAK3/VAV1/NMI/NLRC4/PGC/FCN1/RIOK3/KLK7/CD1D/CD226/IL17A/MNDA/PAK2 | 20 |
| BP | GO:0002573 | myeloid leukocyte differentiation | 20/761 | 208/18723 | 0.000330318 | 0.016516456 | 0.014540737 | PPARG/OCSTAMP/IRF4/TREM2/HCLS1/GPR183/TNFSF11/LILRB1/TLR2/MYC/PIK3CD/MMP9/RARA/LILRB3/PLA2G3/ITGB8/TLR3/LTF/IL17A/ANXA2 | 20 |
| BP | GO:0050866 | negative regulation of cell activation | 20/761 | 210/18723 | 0.000374262 | 0.01808679 | 0.015923226 | BANK1/LILRB2/GPNMB/TREM2/CST7/LILRB1/IL20RB/CTLA4/CD200/LAX1/SAMSN1/CD9/CD86/FGR/PAWR/CR1/DLG1/PRNP/SOX11/MNDA | 20 |
| BP | GO:0045088 | regulation of innate immune response | 20/761 | 218/18723 | 0.000604984 | 0.02387672 | 0.021020557 | PLCG2/PPARG/IL21/ZBP1/FFAR2/PAK3/TREM2/VAV1/SUSD4/NMI/LILRB1/NLRC4/FCN1/RIOK3/CD1D/FGR/CR1/CD226/MNDA/PAK2 | 20 |
| BP | GO:1903039 | positive regulation of leukocyte cell-cell adhesion | 20/761 | 239/18723 | 0.001867748 | 0.051737357 | 0.045548469 | CHST2/PCK1/RHOH/IL21/LILRB2/SLAMF1/ITGA4/VAV1/TNFSF11/LILRB1/CLECL1/IGFBP2/SKAP1/RARA/CD1D/CD86/CR1/XBP1/IL7R/HSPH1 | 20 |
| BP | GO:0002700 | regulation of production of molecular mediator of immune response | 19/761 | 164/18723 | 3.94E-05 | 0.00316395 | 0.002785475 | MZB1/TLR9/IL17F/PLCG2/IL21/FFAR2/SLAMF1/LILRB1/SEMA7A/PGC/KLK7/CD86/FCRL3/CR1/XBP1/SLC7A5/TLR3/CD226/IL17A | 19 |
| BP | GO:0022408 | negative regulation of cell-cell adhesion | 19/761 | 196/18723 | 0.00041639 | 0.01843014 | 0.016225503 | LILRB2/AKNA/JAG1/GPNMB/EPCAM/LILRB1/IL20RB/CTLA4/KLF4/LAX1/CDH1/WNK1/CD9/CD86/PAWR/CR1/DLG1/PODXL/PRNP | 19 |
| BP | GO:0002064 | epithelial cell development | 19/761 | 220/18723 | 0.001672761 | 0.048306062 | 0.04252763 | BFSP2/CXCR4/RAB25/POF1B/CLDN3/BMP5/JAG1/MARVELD2/TP63/EXPH5/KLF5/ARHGEF26/VSIG1/RARA/PODXL/IQGAP1/PECAM1/CLDN1/TNMD | 19 |
| BP | GO:0008037 | cell recognition | 19/761 | 225/18723 | 0.002163209 | 0.054494454 | 0.047975759 | IGLL1/CXCR4/SPACA3/PRF1/TREM2/CLGN/CLEC4M/FCN1/FCN2/ROBO2/HSPA1L/CD9/IGSF9/YWHAZ/DLG1/PAEP/CD226/PECAM1/TULP1 | 19 |
| BP | GO:0002703 | regulation of leukocyte mediated immunity | 19/761 | 226/18723 | 0.002274583 | 0.054961361 | 0.048386813 | PLCG2/STAP1/CR2/IL21/SLAMF1/TREM2/VAV1/SUSD4/LILRB1/IL20RB/CD1D/CXCL6/FGR/CR1/VAMP8/PLA2G3/TLR3/CD226/IL7R | 19 |
| BP | GO:0007043 | cell-cell junction assembly | 18/761 | 146/18723 | 2.71E-05 | 0.002571532 | 0.002263922 | POF1B/PKP1/CLDN3/JUP/MPP7/MARVELD2/GJA1/GJB6/CLDN10/CD9/OCLN/ACE2/DLG1/PECAM1/IL17A/CLDN1/AGT/PAK2 | 18 |
| BP | GO:0071706 | tumor necrosis factor superfamily cytokine production | 18/761 | 186/18723 | 0.00059633 | 0.023732947 | 0.020893982 | TLR9/IL17F/PLCG2/CYBA/LILRA5/SLAMF1/GPNMB/TREM2/LILRB1/TLR2/RARA/CD86/NFKBIL1/GPR18/TLR3/CYBB/LTF/IL17A | 18 |
| BP | GO:1903555 | regulation of tumor necrosis factor superfamily cytokine production | 18/761 | 186/18723 | 0.00059633 | 0.023732947 | 0.020893982 | TLR9/IL17F/PLCG2/CYBA/LILRA5/SLAMF1/GPNMB/TREM2/LILRB1/TLR2/RARA/CD86/NFKBIL1/GPR18/TLR3/CYBB/LTF/IL17A | 18 |
| BP | GO:0002695 | negative regulation of leukocyte activation | 18/761 | 187/18723 | 0.000635448 | 0.024467316 | 0.021540505 | BANK1/LILRB2/GPNMB/CST7/LILRB1/IL20RB/CTLA4/CD200/LAX1/SAMSN1/CD86/FGR/PAWR/CR1/DLG1/PRNP/SOX11/MNDA | 18 |
| BP | GO:0001906 | cell killing | 18/761 | 188/18723 | 0.000676752 | 0.025640772 | 0.02257359 | STAP1/IL21/SLAMF7/PRF1/VAV1/LILRB1/GZMB/EMP2/CD1D/CXCL6/CCL13/TUBB4B/TREM1/CD226/IL7R/GNLY/LTF/CAMP | 18 |
| BP | GO:0071674 | mononuclear cell migration | 18/761 | 196/18723 | 0.001098486 | 0.034682861 | 0.030534053 | C10orf99/CXCR4/SLAMF1/BMP5/ITGA4/GPR183/TNFSF11/RET/S100A14/PIK3CD/CD200/MYO1G/WNK1/CCL13/CXCR1/AIRE/PECAM1/CCL18 | 18 |
| BP | GO:0002224 | toll-like receptor signaling pathway | 17/761 | 121/18723 | 7.97E-06 | 0.001019525 | 0.000897568 | TLR9/PLCG2/CYBA/TLR10/IRAK2/IRF4/TREM2/NMI/TLR2/S100A14/LGR4/CTSS/NFKBIL1/FCRL3/RAB11FIP2/TLR3/LTF | 17 |
| BP | GO:0019730 | antimicrobial humoral response | 17/761 | 122/18723 | 8.91E-06 | 0.001082064 | 0.000952627 | IL17F/RNASE6/CXCL3/PGC/JCHAIN/WFDC2/KLK7/CXCL6/PF4V1/CCL13/CXCL2/SLPI/GNLY/LTF/IL17A/CXCL1/CAMP | 17 |
| BP | GO:0071621 | granulocyte chemotaxis | 17/761 | 125/18723 | 1.24E-05 | 0.001465675 | 0.001290349 | DPEP1/SCG2/CXADR/SLAMF1/VAV1/CXCL3/S100A14/PIK3CD/CXCL6/PF4V1/CCL13/CXCR1/RAC1/TREM1/CXCL2/CCL18/CXCL1 | 17 |
| BP | GO:0050729 | positive regulation of inflammatory response | 17/761 | 142/18723 | 6.56E-05 | 0.004634271 | 0.004079914 | TLR9/PLCG2/STAP1/IL21/LILRA5/TLR10/ZBP1/FFAR2/TREM2/TNFSF11/NMI/TLR2/TGM2/VAMP8/PLA2G3/TLR3/AGT | 17 |
| BP | GO:0006941 | striated muscle contraction | 17/761 | 179/18723 | 0.001032079 | 0.032937571 | 0.028997537 | JSRP1/CXCR4/RCSD1/DSP/KCNA5/MYL1/JUP/GJA1/P2RX4/PPP1R13L/DSC2/MYL3/MB/STAC2/ACE2/DLG1/TNNT1 | 17 |
| BP | GO:0042100 | B cell proliferation | 16/761 | 99/18723 | 2.29E-06 | 0.000454218 | 0.000399884 | MZB1/CD19/TLR9/IKZF3/CD79A/CD180/CR2/IL21/GPR183/CTLA4/CD38/PAWR/FCRL3/IL7R/MS4A1/MNDA | 16 |
| BP | GO:0002702 | positive regulation of production of molecular mediator of immune response | 16/761 | 117/18723 | 2.07E-05 | 0.002127135 | 0.001872684 | MZB1/TLR9/IL17F/PLCG2/IL21/FFAR2/SLAMF1/LILRB1/SEMA7A/PGC/KLK7/CD86/XBP1/SLC7A5/CD226/IL17A | 16 |
| BP | GO:1990266 | neutrophil migration | 16/761 | 122/18723 | 3.50E-05 | 0.003013415 | 0.002652947 | DPEP1/RHOH/CXADR/VAV1/CXCL3/PIK3CD/CXCL6/PF4V1/CCL13/CXCR1/RAC1/TREM1/CXCL2/PECAM1/CCL18/CXCL1 | 16 |
| BP | GO:0050853 | B cell receptor signaling pathway | 16/761 | 131/18723 | 8.38E-05 | 0.005512937 | 0.004853473 | IGLL1/CD19/KLHL6/CD79A/BANK1/PLCG2/STAP1/PLEKHA1/LAT2/CTLA4/PIK3CD/ITK/CD38/FCRL3/MS4A1/MNDA | 16 |
| BP | GO:0006986 | response to unfolded protein | 16/761 | 137/18723 | 0.000142998 | 0.008279292 | 0.007288913 | HSPA1B/BHLHA15/HSPA2/CTH/EDEM1/CHAC1/HSPA13/DNAJB1/ERLEC1/HERPUD1/HSPA4L/HSPA1L/XBP1/HSPA1A/TM7SF3/HSPH1 | 16 |
| BP | GO:0030183 | B cell differentiation | 16/761 | 141/18723 | 0.000200291 | 0.01142864 | 0.010061532 | CD19/TLR9/IKZF3/CD79A/POU2AF1/PLCG2/CR2/IL21/FLT3/ITGA4/GPR183/FCRL3/CR1/XBP1/MS4A1/KLF6 | 16 |
| BP | GO:0051250 | negative regulation of lymphocyte activation | 16/761 | 157/18723 | 0.000673463 | 0.025640772 | 0.02257359 | BANK1/LILRB2/GPNMB/LILRB1/IL20RB/CTLA4/LAX1/SAMSN1/CD86/FGR/PAWR/CR1/DLG1/PRNP/SOX11/MNDA | 16 |
| BP | GO:0035966 | response to topologically incorrect protein | 16/761 | 159/18723 | 0.000773053 | 0.02774261 | 0.024424004 | HSPA1B/BHLHA15/HSPA2/CTH/EDEM1/CHAC1/HSPA13/DNAJB1/ERLEC1/HERPUD1/HSPA4L/HSPA1L/XBP1/HSPA1A/TM7SF3/HSPH1 | 16 |
| BP | GO:0035148 | tube formation | 15/761 | 148/18723 | 0.001036254 | 0.032937571 | 0.028997537 | BMP5/LRP2/HOXA1/FGFR2/RET/PIK3CD/GRHL3/HES5/TULP3/RARA/TGM2/FZD6/YWHAZ/PODXL/SOX11 | 15 |
| BP | GO:0061844 | antimicrobial humoral immune response mediated by antimicrobial peptide | 14/761 | 79/18723 | 3.19E-06 | 0.000559482 | 0.000492556 | IL17F/RNASE6/CXCL3/PGC/KLK7/CXCL6/PF4V1/CCL13/CXCL2/GNLY/LTF/IL17A/CXCL1/CAMP | 14 |
| BP | GO:0030593 | neutrophil chemotaxis | 14/761 | 103/18723 | 7.17E-05 | 0.004850813 | 0.004270552 | DPEP1/CXADR/VAV1/CXCL3/PIK3CD/CXCL6/PF4V1/CCL13/CXCR1/RAC1/TREM1/CXCL2/CCL18/CXCL1 | 14 |
| BP | GO:0001909 | leukocyte mediated cytotoxicity | 14/761 | 124/18723 | 0.000517514 | 0.021689808 | 0.019095246 | STAP1/IL21/SLAMF7/PRF1/VAV1/LILRB1/GZMB/EMP2/CD1D/CXCL6/TUBB4B/TREM1/CD226/IL7R | 14 |
| BP | GO:0045089 | positive regulation of innate immune response | 14/761 | 131/18723 | 0.000897537 | 0.03014707 | 0.02654084 | PLCG2/IL21/ZBP1/FFAR2/PAK3/VAV1/NMI/NLRC4/FCN1/RIOK3/CD1D/CD226/MNDA/PAK2 | 14 |
| BP | GO:0072073 | kidney epithelium development | 14/761 | 136/18723 | 0.001294309 | 0.039547394 | 0.034816685 | JAG1/EPCAM/FGFR2/RET/MYC/LGR4/HES5/RARA/ROBO2/DLG1/PODXL/IQGAP1/PECAM1/AGT | 14 |
| BP | GO:0046718 | viral entry into host cell | 14/761 | 144/18723 | 0.002228427 | 0.054961361 | 0.048386813 | CR2/CXCR4/CXADR/SLAMF1/HSPA1B/MID2/CLEC4M/FCN1/CD86/CR1/ACE2/HSPA1A/VAMP8/CLDN1 | 14 |
| BP | GO:0034113 | heterotypic cell-cell adhesion | 13/761 | 61/18723 | 8.05E-07 | 0.000242434 | 0.000213434 | ITGAX/CXADR/LILRB2/DSP/JUP/ITGA4/PERP/DSC2/KLF4/CD200/SKAP1/CD1D/WNK1 | 13 |
| BP | GO:0062207 | regulation of pattern recognition receptor signaling pathway | 13/761 | 105/18723 | 0.000331306 | 0.016516456 | 0.014540737 | TLR9/CYBA/HSPA1B/IRF4/TREM2/TLR2/LGR4/RIOK3/NFKBIL1/FCRL3/HSPA1A/TLR3/LTF | 13 |
| BP | GO:0032526 | response to retinoic acid | 13/761 | 107/18723 | 0.000399085 | 0.01824833 | 0.016065442 | PCK1/TBX1/PTGES/GJA1/FGFR2/RET/KLF4/IGFBP2/AQP3/CD38/RARA/SLC6A4/FZD10 | 13 |
| BP | GO:0042303 | molting cycle | 13/761 | 107/18723 | 0.000399085 | 0.01824833 | 0.016065442 | SOX21/ACVR1B/FOXE1/FERMT1/TP63/PPP1R13L/FGFR2/KRT16/LGR4/FZD6/SOSTDC1/KRT14/KRT27 | 13 |
| BP | GO:0042633 | hair cycle | 13/761 | 107/18723 | 0.000399085 | 0.01824833 | 0.016065442 | SOX21/ACVR1B/FOXE1/FERMT1/TP63/PPP1R13L/FGFR2/KRT16/LGR4/FZD6/SOSTDC1/KRT14/KRT27 | 13 |
| BP | GO:0070830 | bicellular tight junction assembly | 12/761 | 70/18723 | 2.27E-05 | 0.002286482 | 0.00201297 | POF1B/CLDN3/MPP7/MARVELD2/GJA1/CLDN10/OCLN/DLG1/PECAM1/IL17A/CLDN1/PAK2 | 12 |
| BP | GO:0120192 | tight junction assembly | 12/761 | 74/18723 | 4.03E-05 | 0.003184094 | 0.002803208 | POF1B/CLDN3/MPP7/MARVELD2/GJA1/CLDN10/OCLN/DLG1/PECAM1/IL17A/CLDN1/PAK2 | 12 |
| BP | GO:0043297 | apical junction assembly | 12/761 | 78/18723 | 6.88E-05 | 0.004793261 | 0.004219885 | POF1B/CLDN3/MPP7/MARVELD2/GJA1/CLDN10/OCLN/DLG1/PECAM1/IL17A/CLDN1/PAK2 | 12 |
| BP | GO:0120193 | tight junction organization | 12/761 | 80/18723 | 8.86E-05 | 0.005750922 | 0.005062989 | POF1B/CLDN3/MPP7/MARVELD2/GJA1/CLDN10/OCLN/DLG1/PECAM1/IL17A/CLDN1/PAK2 | 12 |
| BP | GO:0050672 | negative regulation of lymphocyte proliferation | 12/761 | 83/18723 | 0.000127507 | 0.007597539 | 0.006688711 | LILRB2/GPNMB/LILRB1/IL20RB/CTLA4/CD86/PAWR/CR1/DLG1/PRNP/SOX11/MNDA | 12 |
| BP | GO:0032945 | negative regulation of mononuclear cell proliferation | 12/761 | 84/18723 | 0.000143349 | 0.008279292 | 0.007288913 | LILRB2/GPNMB/LILRB1/IL20RB/CTLA4/CD86/PAWR/CR1/DLG1/PRNP/SOX11/MNDA | 12 |
| BP | GO:0070098 | chemokine-mediated signaling pathway | 12/761 | 88/18723 | 0.000224692 | 0.012668357 | 0.011152952 | MPL/CXCR4/TREM2/CXCL3/WNK1/CXCL6/PF4V1/CCL13/CXCR1/CXCL2/CCL18/CXCL1 | 12 |
| BP | GO:0070664 | negative regulation of leukocyte proliferation | 12/761 | 90/18723 | 0.000278301 | 0.014644819 | 0.012892987 | LILRB2/GPNMB/LILRB1/IL20RB/CTLA4/CD86/PAWR/CR1/DLG1/PRNP/SOX11/MNDA | 12 |
| BP | GO:1990868 | response to chemokine | 12/761 | 97/18723 | 0.000559321 | 0.022725551 | 0.020007092 | MPL/CXCR4/TREM2/CXCL3/WNK1/CXCL6/PF4V1/CCL13/CXCR1/CXCL2/CCL18/CXCL1 | 12 |
| BP | GO:1990869 | cellular response to chemokine | 12/761 | 97/18723 | 0.000559321 | 0.022725551 | 0.020007092 | MPL/CXCR4/TREM2/CXCL3/WNK1/CXCL6/PF4V1/CCL13/CXCR1/CXCL2/CCL18/CXCL1 | 12 |
| BP | GO:0042116 | macrophage activation | 12/761 | 106/18723 | 0.001239527 | 0.038368637 | 0.033778932 | PLCG2/STAP1/SPACA3/TREM2/CST7/MAPT/NMI/TLR2/CD200/C1QA/PLA2G3/TLR3 | 12 |
| BP | GO:0032609 | interferon-gamma production | 12/761 | 112/18723 | 0.001995475 | 0.052349442 | 0.046087335 | TLR9/IL21/SLAMF1/HMHB1/LILRB1/IL20RB/RARA/CR1/SLC7A5/TLR3/CD226/PRNP | 12 |
| BP | GO:0032649 | regulation of interferon-gamma production | 12/761 | 112/18723 | 0.001995475 | 0.052349442 | 0.046087335 | TLR9/IL21/SLAMF1/HMHB1/LILRB1/IL20RB/RARA/CR1/SLC7A5/TLR3/CD226/PRNP | 12 |
| BP | GO:0050829 | defense response to Gram-negative bacterium | 11/761 | 88/18723 | 0.000860444 | 0.02974497 | 0.026186839 | TLR9/IL17F/RNASE6/PRB3/TREM2/FCN2/CXCL6/TREM1/LTF/IL17A/CAMP | 11 |
| BP | GO:0032755 | positive regulation of interleukin-6 production | 11/761 | 93/18723 | 0.001364936 | 0.041438043 | 0.036481172 | TLR9/POU2AF1/IL17F/PLCG2/CYBA/LILRA5/LILRB2/TLR2/XBP1/TLR3/IL17A | 11 |
| BP | GO:0034620 | cellular response to unfolded protein | 11/761 | 96/18723 | 0.001769553 | 0.050791542 | 0.044715794 | HSPA1B/BHLHA15/HSPA2/CTH/HSPA13/ERLEC1/HERPUD1/HSPA1L/XBP1/HSPA1A/TM7SF3 | 11 |
| BP | GO:0032602 | chemokine production | 11/761 | 99/18723 | 0.002267026 | 0.054961361 | 0.048386813 | TLR9/IL17F/FFAR2/TREM2/TLR2/KLF4/CXCL6/TREM1/AIRE/TLR3/IL17A | 11 |
| BP | GO:0045104 | intermediate filament cytoskeleton organization | 10/761 | 51/18723 | 3.27E-05 | 0.002974008 | 0.002618253 | KRT3/BFSP2/DSP/PKP1/NEFM/NEFL/KRT16/KRT18/DNAJB6/KRT14 | 10 |
| BP | GO:0043277 | apoptotic cell clearance | 10/761 | 52/18723 | 3.90E-05 | 0.00316395 | 0.002785475 | RHOH/TREM2/FCN1/FCN2/RARA/TXNDC5/TGM2/RAC1/XKR9/MARCO | 10 |
| BP | GO:0045103 | intermediate filament-based process | 10/761 | 52/18723 | 3.90E-05 | 0.00316395 | 0.002785475 | KRT3/BFSP2/DSP/PKP1/NEFM/NEFL/KRT16/KRT18/DNAJB6/KRT14 | 10 |
| BP | GO:0030888 | regulation of B cell proliferation | 10/761 | 64/18723 | 0.000240495 | 0.013362376 | 0.011763952 | MZB1/TLR9/IKZF3/IL21/GPR183/CTLA4/CD38/PAWR/FCRL3/MNDA | 10 |
| BP | GO:0042130 | negative regulation of T cell proliferation | 10/761 | 67/18723 | 0.000352903 | 0.017409858 | 0.015327269 | LILRB2/GPNMB/LILRB1/IL20RB/CTLA4/CD86/PAWR/CR1/DLG1/PRNP | 10 |
| BP | GO:0034121 | regulation of toll-like receptor signaling pathway | 10/761 | 75/18723 | 0.000881436 | 0.03003222 | 0.026439728 | TLR9/CYBA/IRF4/TREM2/TLR2/LGR4/NFKBIL1/FCRL3/TLR3/LTF | 10 |
| BP | GO:0001942 | hair follicle development | 10/761 | 81/18723 | 0.001606313 | 0.047340192 | 0.041677298 | SOX21/ACVR1B/FOXE1/FERMT1/TP63/FGFR2/LGR4/FZD6/SOSTDC1/KRT27 | 10 |
| BP | GO:0071260 | cellular response to mechanical stimulus | 10/761 | 81/18723 | 0.001606313 | 0.047340192 | 0.041677298 | ANKRD1/CYBA/GJA1/SLC38A2/RAC1/TLR3/MMP7/MAP3K1/SLC2A1/AGT | 10 |
| BP | GO:0022404 | molting cycle process | 10/761 | 84/18723 | 0.002117836 | 0.053636743 | 0.047220648 | SOX21/ACVR1B/FOXE1/FERMT1/TP63/FGFR2/LGR4/FZD6/SOSTDC1/KRT27 | 10 |
| BP | GO:0022405 | hair cycle process | 10/761 | 84/18723 | 0.002117836 | 0.053636743 | 0.047220648 | SOX21/ACVR1B/FOXE1/FERMT1/TP63/FGFR2/LGR4/FZD6/SOSTDC1/KRT27 | 10 |
| BP | GO:0032370 | positive regulation of lipid transport | 10/761 | 84/18723 | 0.002117836 | 0.053636743 | 0.047220648 | DENND5B/DBI/PPARG/PTGES/TREM2/P2RX4/TNFSF11/PLA2G3/RETN/ANXA2 | 10 |
| BP | GO:0098773 | skin epidermis development | 10/761 | 85/18723 | 0.002314852 | 0.055650461 | 0.048993482 | SOX21/ACVR1B/FOXE1/FERMT1/TP63/FGFR2/LGR4/FZD6/SOSTDC1/KRT27 | 10 |
| BP | GO:0032615 | interleukin-12 production | 9/761 | 62/18723 | 0.000849125 | 0.029569521 | 0.026032378 | TLR9/PLCG2/LILRA5/SLAMF1/LTB/LILRB1/TLR2/TLR3/IL17A | 9 |
| BP | GO:0032655 | regulation of interleukin-12 production | 9/761 | 62/18723 | 0.000849125 | 0.029569521 | 0.026032378 | TLR9/PLCG2/LILRA5/SLAMF1/LTB/LILRB1/TLR2/TLR3/IL17A | 9 |
| BP | GO:1904888 | cranial skeletal system development | 9/761 | 68/18723 | 0.001661913 | 0.04828723 | 0.042511051 | TBX1/FOXE1/IRF6/TP63/HOXA1/FGFR2/TULP3/EIF4A3/NEUROG1 | 9 |
| BP | GO:0003208 | cardiac ventricle morphogenesis | 9/761 | 71/18723 | 0.002254935 | 0.054961361 | 0.048386813 | NPY2R/MESP1/DSP/JAG1/LRP2/PPP1R13L/FGFR2/MYL3/SOX11 | 9 |
| BP | GO:0045730 | respiratory burst | 8/761 | 37/18723 | 9.72E-05 | 0.00621945 | 0.005475472 | CYBA/NCF4/MPO/TREM2/PIK3CD/JCHAIN/RAC1/CYBB | 8 |
| BP | GO:0030574 | collagen catabolic process | 8/761 | 42/18723 | 0.000248287 | 0.013362376 | 0.011763952 | MMP3/MMP10/MMP1/MMP9/MMP27/CTSS/ADAMTS3/MMP7 | 8 |
| BP | GO:0002920 | regulation of humoral immune response | 8/761 | 45/18723 | 0.000407246 | 0.01824833 | 0.016065442 | IL17F/CR2/TREM2/SUSD4/PGC/KLK7/CR1/IL17A | 8 |
| BP | GO:0050832 | defense response to fungus | 8/761 | 49/18723 | 0.000738523 | 0.027113539 | 0.023870183 | C10orf99/PLCG2/CLEC4D/MPO/CLEC4E/GNLY/LTF/IL17A | 8 |
| BP | GO:0002218 | activation of innate immune response | 8/761 | 52/18723 | 0.001107285 | 0.034729155 | 0.030574809 | PLCG2/ZBP1/FFAR2/PAK3/NLRC4/FCN1/MNDA/PAK2 | 8 |
| BP | GO:0031113 | regulation of microtubule polymerization | 8/761 | 55/18723 | 0.001610002 | 0.047340192 | 0.041677298 | HSPA1B/MAPT/MAP2/MAPRE3/SLAIN1/OCLN/HSPA1A/RAC1 | 8 |
| BP | GO:0031529 | ruffle organization | 8/761 | 56/18723 | 0.001812698 | 0.051516459 | 0.045353995 | STAP1/SH3YL1/PLEKHA1/KANK1/ARFIP2/PLEK/ARHGEF26/RAC1 | 8 |
| BP | GO:0050891 | multicellular organismal water homeostasis | 8/761 | 57/18723 | 0.002035007 | 0.052665541 | 0.046365622 | KRT1/CYP4F12/TP63/KRT16/GRHL3/AQP3/FLG2/CLDN1 | 8 |
| BP | GO:0070841 | inclusion body assembly | 7/761 | 24/18723 | 3.36E-05 | 0.003006861 | 0.002647176 | HSPA1B/HSPA2/MAPT/DNAJB1/DNAJB6/HSPA1A/DNAJA4 | 7 |
| BP | GO:0045109 | intermediate filament organization | 7/761 | 25/18723 | 4.51E-05 | 0.003419817 | 0.003010734 | BFSP2/DSP/PKP1/NEFM/NEFL/DNAJB6/KRT14 | 7 |
| BP | GO:0002335 | mature B cell differentiation | 7/761 | 33/18723 | 0.000301894 | 0.015540984 | 0.013681952 | CD19/POU2AF1/PLCG2/IL21/GPR183/CR1/XBP1 | 7 |
| BP | GO:0048730 | epidermis morphogenesis | 7/761 | 33/18723 | 0.000301894 | 0.015540984 | 0.013681952 | FOXE1/TP63/FGFR2/KLF4/SOSTDC1/FLG2/KRT27 | 7 |
| BP | GO:0051085 | chaperone cofactor-dependent protein refolding | 7/761 | 34/18723 | 0.000366965 | 0.017916965 | 0.015773715 | HSPA1B/HSPA2/HSPA13/DNAJB1/HSPA1L/HSPA1A/HSPH1 | 7 |
| BP | GO:0051084 | 'de novo' posttranslational protein folding | 7/761 | 39/18723 | 0.000879595 | 0.03003222 | 0.026439728 | HSPA1B/HSPA2/HSPA13/DNAJB1/HSPA1L/HSPA1A/HSPH1 | 7 |
| BP | GO:0032892 | positive regulation of organic acid transport | 7/761 | 40/18723 | 0.001029308 | 0.032937571 | 0.028997537 | CCK/PTGES/P2RX4/TNFSF11/ACE2/PLA2G3/AGT | 7 |
| BP | GO:0150077 | regulation of neuroinflammatory response | 7/761 | 40/18723 | 0.001029308 | 0.032937571 | 0.028997537 | PLCG2/STAP1/MMP3/TREM2/CST7/MMP9/CD200 | 7 |
| BP | GO:0032733 | positive regulation of interleukin-10 production | 7/761 | 41/18723 | 0.001198333 | 0.037337529 | 0.032871167 | TLR9/PLCG2/LILRA5/IRF4/TREM2/IL20RB/TLR2 | 7 |
| BP | GO:0006458 | 'de novo' protein folding | 7/761 | 43/18723 | 0.001601072 | 0.047340192 | 0.041677298 | HSPA1B/HSPA2/HSPA13/DNAJB1/HSPA1L/HSPA1A/HSPH1 | 7 |
| BP | GO:1903793 | positive regulation of anion transport | 7/761 | 43/18723 | 0.001601072 | 0.047340192 | 0.041677298 | CCK/PTGES/P2RX4/TNFSF11/ACE2/PLA2G3/AGT | 7 |
| BP | GO:0062208 | positive regulation of pattern recognition receptor signaling pathway | 7/761 | 44/18723 | 0.00183832 | 0.051516459 | 0.045353995 | TLR9/CYBA/HSPA1B/TLR2/HSPA1A/TLR3/LTF | 7 |
| BP | GO:0150076 | neuroinflammatory response | 7/761 | 44/18723 | 0.00183832 | 0.051516459 | 0.045353995 | PLCG2/STAP1/MMP3/TREM2/CST7/MMP9/CD200 | 7 |
| BP | GO:0090084 | negative regulation of inclusion body assembly | 6/761 | 11/18723 | 1.71E-06 | 0.00036911 | 0.000324956 | HSPA1B/HSPA2/DNAJB1/DNAJB6/HSPA1A/DNAJA4 | 6 |
| BP | GO:0090083 | regulation of inclusion body assembly | 6/761 | 17/18723 | 3.72E-05 | 0.003148772 | 0.002772112 | HSPA1B/HSPA2/DNAJB1/DNAJB6/HSPA1A/DNAJA4 | 6 |
| BP | GO:0042026 | protein refolding | 6/761 | 23/18723 | 0.000246414 | 0.013362376 | 0.011763952 | HSPA1B/HSPA2/HSPA13/HSPA1L/HSPA1A/DNAJA4 | 6 |
| BP | GO:0002922 | positive regulation of humoral immune response | 6/761 | 24/18723 | 0.000317335 | 0.016160198 | 0.014227095 | IL17F/TREM2/PGC/KLK7/CR1/IL17A | 6 |
| BP | GO:0032753 | positive regulation of interleukin-4 production | 6/761 | 25/18723 | 0.000403303 | 0.01824833 | 0.016065442 | IRF4/CLECL1/IL20RB/RARA/CD86/SLC7A5 | 6 |
| BP | GO:0061436 | establishment of skin barrier | 6/761 | 25/18723 | 0.000403303 | 0.01824833 | 0.016065442 | KRT1/TP63/KRT16/GRHL3/FLG2/CLDN1 | 6 |
| BP | GO:0062009 | secondary palate development | 6/761 | 25/18723 | 0.000403303 | 0.01824833 | 0.016065442 | TBX1/FOXE1/COL11A2/DLG1/ITGB8/SOX11 | 6 |
| BP | GO:0033561 | regulation of water loss via skin | 6/761 | 27/18723 | 0.000628941 | 0.024467316 | 0.021540505 | KRT1/TP63/KRT16/GRHL3/FLG2/CLDN1 | 6 |
| BP | GO:0002313 | mature B cell differentiation involved in immune response | 6/761 | 28/18723 | 0.000773232 | 0.02774261 | 0.024424004 | POU2AF1/PLCG2/IL21/GPR183/CR1/XBP1 | 6 |
| BP | GO:0006972 | hyperosmotic response | 6/761 | 28/18723 | 0.000773232 | 0.02774261 | 0.024424004 | RCSD1/SST/YBX3/TLR3/SLC2A1/CLDN1 | 6 |
| BP | GO:0003382 | epithelial cell morphogenesis | 6/761 | 33/18723 | 0.001912718 | 0.051737357 | 0.045548469 | RAB25/POF1B/CLDN3/ARHGEF26/VSIG1/TNMD | 6 |
| BP | GO:0031116 | positive regulation of microtubule polymerization | 6/761 | 33/18723 | 0.001912718 | 0.051737357 | 0.045548469 | HSPA1B/MAPT/SLAIN1/OCLN/HSPA1A/RAC1 | 6 |
| BP | GO:0032633 | interleukin-4 production | 6/761 | 33/18723 | 0.001912718 | 0.051737357 | 0.045548469 | IRF4/CLECL1/IL20RB/RARA/CD86/SLC7A5 | 6 |
| BP | GO:0032673 | regulation of interleukin-4 production | 6/761 | 33/18723 | 0.001912718 | 0.051737357 | 0.045548469 | IRF4/CLECL1/IL20RB/RARA/CD86/SLC7A5 | 6 |
| BP | GO:0035025 | positive regulation of Rho protein signal transduction | 6/761 | 33/18723 | 0.001912718 | 0.051737357 | 0.045548469 | P2RY10/GPR65/LPAR6/GPR18/RAC1/NET1 | 6 |
| BP | GO:0050869 | negative regulation of B cell activation | 6/761 | 34/18723 | 0.002243951 | 0.054961361 | 0.048386813 | BANK1/CTLA4/SAMSN1/PAWR/CR1/MNDA | 6 |
| BP | GO:0098751 | bone cell development | 6/761 | 34/18723 | 0.002243951 | 0.054961361 | 0.048386813 | ZNF385A/TNFSF11/LILRB1/MEIS1/LTF/ANXA2 | 6 |
| BP | GO:1903975 | regulation of glial cell migration | 5/761 | 19/18723 | 0.000791129 | 0.02817132 | 0.024801431 | STAP1/TREM2/GPR183/P2RX4/TIAM1 | 5 |
| BP | GO:0070269 | pyroptosis | 5/761 | 21/18723 | 0.001294152 | 0.039547394 | 0.034816685 | ZBP1/GSDMC/TREM2/NLRC4/GZMB | 5 |
| BP | GO:0002220 | innate immune response activating cell surface receptor signaling pathway | 5/761 | 22/18723 | 0.001619322 | 0.047340192 | 0.041677298 | PLCG2/FFAR2/PAK3/FCN1/PAK2 | 5 |
| BP | GO:0002758 | innate immune response-activating signal transduction | 5/761 | 23/18723 | 0.002000686 | 0.052349442 | 0.046087335 | PLCG2/FFAR2/PAK3/FCN1/PAK2 | 5 |
| BP | GO:2000252 | negative regulation of feeding behavior | 4/761 | 10/18723 | 0.000467269 | 0.020022567 | 0.017627443 | CCK/NPY2R/MC4R/RETN | 4 |
| BP | GO:0002759 | regulation of antimicrobial humoral response | 4/761 | 11/18723 | 0.00071072 | 0.026296632 | 0.023150996 | IL17F/PGC/KLK7/IL17A | 4 |
| BP | GO:0032308 | positive regulation of prostaglandin secretion | 4/761 | 11/18723 | 0.00071072 | 0.026296632 | 0.023150996 | PTGES/P2RX4/TNFSF11/PLA2G3 | 4 |
| BP | GO:0032306 | regulation of prostaglandin secretion | 4/761 | 12/18723 | 0.001031923 | 0.032937571 | 0.028997537 | PTGES/P2RX4/TNFSF11/PLA2G3 | 4 |
| BP | GO:0098911 | regulation of ventricular cardiac muscle cell action potential | 4/761 | 12/18723 | 0.001031923 | 0.032937571 | 0.028997537 | DSP/JUP/DSC2/DLG1 | 4 |
| BP | GO:0046348 | amino sugar catabolic process | 4/761 | 14/18723 | 0.001955479 | 0.052028929 | 0.045805163 | RENBP/NAGK/CHI3L2/ALDH1A1 | 4 |
| BP | GO:0055057 | neuroblast division | 4/761 | 14/18723 | 0.001955479 | 0.052028929 | 0.045805163 | AKNA/FGFR2/RAB10/DCT | 4 |
| CC | GO:0070820 | tertiary granule | 24/797 | 164/19550 | 5.56E-08 | 1.53E-05 | 1.38E-05 | ITGAX/CYBA/LILRB2/SIGLEC5/ENPP4/DSP/PKP1/ARL8A/CLEC4D/CD53/PLD1/MMP9/CTSS/CR1/VAMP8/RAC1/CYBB/YPEL5/FLG2/LTF/RAP2B/CXCL1/MGAM/CAMP | 24 |
| CC | GO:0005911 | cell-cell junction | 47/797 | 494/19550 | 6.62E-08 | 1.53E-05 | 1.38E-05 | KRT8/CXADR/DSC3/POF1B/SHROOM1/DSP/KCNA5/PKP1/CLDN3/JUP/JAG1/PANX2/VAV1/TRPC4/CDC42BPA/MPP7/MARVELD2/GJA1/CD53/DSG3/TIAM1/EPCAM/PERP/DSC2/GJB6/AJUBA/CDH1/CLDN10/TRIM29/AQP3/ITK/KRT18/SKAP1/FAT2/PARD6A/OCLN/DLG1/PODXL/RAB10/YBX3/IQGAP1/PECAM1/SLC2A1/RAP2B/ANXA2/CLDN1/PAK2 | 47 |
| CC | GO:0045121 | membrane raft | 36/797 | 335/19550 | 1.22E-07 | 1.53E-05 | 1.38E-05 | CD19/CD79A/KCNA3/ARID3A/CXADR/KCNA5/TREM2/TRPC4/MAPT/GJA1/MALL/TLR2/RET/P2RX1/ARID3C/LAT2/MAL2/CDH1/EMP2/SMPD2/SKAP1/SLC6A4/CR1/ACE2/DLG1/PODXL/ATP1B3/S100A10/IQGAP1/CD226/PECAM1/SLC2A1/PRNP/MS4A1/RAP2B/ANXA2 | 36 |
| CC | GO:0098857 | membrane microdomain | 36/797 | 335/19550 | 1.22E-07 | 1.53E-05 | 1.38E-05 | CD19/CD79A/KCNA3/ARID3A/CXADR/KCNA5/TREM2/TRPC4/MAPT/GJA1/MALL/TLR2/RET/P2RX1/ARID3C/LAT2/MAL2/CDH1/EMP2/SMPD2/SKAP1/SLC6A4/CR1/ACE2/DLG1/PODXL/ATP1B3/S100A10/IQGAP1/CD226/PECAM1/SLC2A1/PRNP/MS4A1/RAP2B/ANXA2 | 36 |
| CC | GO:0001533 | cornified envelope | 12/797 | 45/19550 | 1.61E-07 | 1.62E-05 | 1.45E-05 | DSC3/KRT1/DSP/RPTN/PKP1/JUP/DSG3/DSC2/FLG2/SCEL/SPRR1A/SPRR1B | 12 |
| CC | GO:0016324 | apical plasma membrane | 35/797 | 367/19550 | 3.00E-06 | 0.000251341 | 0.00022557 | DPEP1/TLR9/CYBA/SLC7A8/SHROOM1/KCNA5/P2RY1/AQP6/CYP4F12/JAG1/LRP2/MARVELD2/GJA1/EPCAM/ACY3/PLD1/IGFBP2/MAL2/GJB6/EMP2/SLC22A12/FZD6/CD9/ADRB2/KIAA1614/PARD6A/OCLN/ACE2/DLG1/PODXL/ATP1B3/SLC7A5/SLC2A1/MGAM/CLDN1 | 35 |
| CC | GO:0030057 | desmosome | 8/797 | 25/19550 | 4.30E-06 | 0.000308027 | 0.000276443 | DSC3/POF1B/DSP/PKP1/JUP/DSG3/PERP/DSC2 | 8 |
| CC | GO:0101002 | ficolin-1-rich granule | 22/797 | 185/19550 | 6.87E-06 | 0.000431276 | 0.000387055 | ITGAX/LILRB2/SIGLEC5/KRT1/ENPP4/DSP/PKP1/ARL8A/HSPA1B/CLEC4D/JUP/FCN1/MMP9/CALML5/CTSS/CR1/PGM2/HSPA1A/RAC1/YPEL5/MGAM/MNDA | 22 |
| CC | GO:0101003 | ficolin-1-rich granule membrane | 11/797 | 61/19550 | 3.13E-05 | 0.001586622 | 0.001423934 | ITGAX/LILRB2/SIGLEC5/ENPP4/DSP/PKP1/ARL8A/CLEC4D/CR1/RAC1/MGAM | 11 |
| CC | GO:0030667 | secretory granule membrane | 29/797 | 311/19550 | 3.16E-05 | 0.001586622 | 0.001423934 | ITGAX/CYBA/LILRB2/SIGLEC5/SPACA3/ENPP4/DSP/PKP1/ARL8A/CLEC4D/DEGS1/CD53/SELL/TLR2/P2RX1/PLD1/CD38/CD9/CR1/LILRB3/RAB10/CXCR1/VAMP8/RAC1/IQGAP1/PECAM1/CYBB/RAP2B/MGAM | 29 |
| CC | GO:0045177 | apical part of cell | 35/797 | 435/19550 | 0.000108483 | 0.004950771 | 0.004443132 | DPEP1/TLR9/CYBA/SLC7A8/SHROOM1/KCNA5/P2RY1/AQP6/CYP4F12/JAG1/LRP2/MARVELD2/GJA1/EPCAM/ACY3/PLD1/IGFBP2/MAL2/GJB6/EMP2/SLC22A12/FZD6/CD9/ADRB2/KIAA1614/PARD6A/OCLN/ACE2/DLG1/PODXL/ATP1B3/SLC7A5/SLC2A1/MGAM/CLDN1 | 35 |
| CC | GO:0016327 | apicolateral plasma membrane | 6/797 | 21/19550 | 0.000144447 | 0.006042699 | 0.005423097 | KRT8/CXADR/CLDN3/JUP/FZD6/OCLN | 6 |
| CC | GO:0070821 | tertiary granule membrane | 11/797 | 73/19550 | 0.000170623 | 0.00658869 | 0.005913104 | ITGAX/CYBA/LILRB2/SIGLEC5/CLEC4D/CD53/PLD1/VAMP8/CYBB/RAP2B/MGAM | 11 |
| CC | GO:0043296 | apical junction complex | 16/797 | 145/19550 | 0.000286749 | 0.010281983 | 0.009227697 | CXADR/POF1B/SHROOM1/CLDN3/JUP/MPP7/MARVELD2/EPCAM/CDH1/CLDN10/PARD6A/OCLN/DLG1/YBX3/RAP2B/CLDN1 | 16 |
| CC | GO:0016323 | basolateral plasma membrane | 21/797 | 231/19550 | 0.000526274 | 0.017612642 | 0.015806691 | TLR9/CXADR/SLC7A8/DSP/P2RY1/TRPC4/MARVELD2/MAP7/EPCAM/DLG2/VSIG1/TSHR/AQP3/CD38/CD1D/DLG1/ATP1B3/SLC7A5/SLC2A1/ANXA2/CLDN1 | 21 |
| CC | GO:0016328 | lateral plasma membrane | 9/797 | 61/19550 | 0.000769594 | 0.023472639 | 0.02106582 | CLDN3/JUP/MPP7/EPCAM/CDH1/OCLN/DLG1/NSG1/CLDN1 | 9 |
| CC | GO:0070160 | tight junction | 14/797 | 129/19550 | 0.00079489 | 0.023472639 | 0.02106582 | CXADR/POF1B/CLDN3/MPP7/MARVELD2/GJA1/EPCAM/CLDN10/PARD6A/OCLN/DLG1/YBX3/RAP2B/CLDN1 | 14 |
| CC | GO:0009925 | basal plasma membrane | 22/797 | 257/19550 | 0.000881336 | 0.024579477 | 0.022059165 | TLR9/CXADR/SLC7A8/DSP/P2RY1/TRPC4/MARVELD2/MAP7/EPCAM/DLG2/VSIG1/TSHR/AQP3/CD38/CD1D/DLG1/ATP1B3/SLC7A5/CLCA2/SLC2A1/ANXA2/CLDN1 | 22 |
| CC | GO:0072562 | blood microparticle | 15/797 | 146/19550 | 0.000930706 | 0.02459022 | 0.022068807 | KRT1/HSPA1B/ACTA1/HSPA2/OAZ3/FCN2/JCHAIN/ITIH2/HSPA1L/YWHAZ/HSPA1A/TMPRSS13/SLC2A1/BCHE/AGT | 15 |
| CC | GO:0045178 | basal part of cell | 23/797 | 276/19550 | 0.00098056 | 0.024612049 | 0.022088398 | TLR9/CXADR/SLC7A8/DSP/P2RY1/TRPC4/MARVELD2/MAP7/EPCAM/DLG2/VSIG1/TSHR/AQP3/CD38/CD1D/KRT14/DLG1/ATP1B3/SLC7A5/CLCA2/SLC2A1/ANXA2/CLDN1 | 23 |
| CC | GO:0031252 | cell leading edge | 31/797 | 422/19550 | 0.001202573 | 0.028747219 | 0.025799559 | PLCG2/CXCR4/SH3YL1/PSTPIP1/PLEKHA1/ABLIM1/FERMT1/ACTA1/CDC42BPA/KANK1/MAPT/TIAM1/SHISA9/ARFIP2/PLEK/PTPN13/ARHGEF26/AJUBA/CDH1/MYO1G/ROBO2/GDPD2/SAMSN1/FGR/PARD6A/OCLN/PODXL/RAC1/SLK/IQGAP1/ANXA2 | 31 |
| CC | GO:0005923 | bicellular tight junction | 13/797 | 122/19550 | 0.001428701 | 0.032317655 | 0.029003892 | CXADR/POF1B/CLDN3/MPP7/MARVELD2/EPCAM/CLDN10/PARD6A/OCLN/DLG1/YBX3/RAP2B/CLDN1 | 13 |
| CC | GO:0005882 | intermediate filament | 19/797 | 217/19550 | 0.001480689 | 0.032317655 | 0.029003892 | KRT3/KRT76/IFFO1/BFSP2/KRT8/KRT1/DSP/PKP1/NEFM/JUP/NEFL/KRT16/KRT6B/KRT13/KRT18/KRT75/KRT5/KRT14/KRT27 | 19 |
| CC | GO:0044304 | main axon | 9/797 | 68/19550 | 0.001698169 | 0.035520031 | 0.031877906 | ERMN/CCK/MAPT/TIAM1/DLG2/MAP2/ROBO2/DLG1/NCMAP | 9 |
| CC | GO:0042581 | specific granule | 15/797 | 160/19550 | 0.00232948 | 0.046775959 | 0.041979682 | CYBA/CLEC4D/JUP/DEGS1/CD53/P2RX1/PLD1/VAMP8/SLPI/CYBB/LTF/RETN/RAP2B/CXCL1/CAMP | 15 |
| CC | GO:0044291 | cell-cell contact zone | 9/797 | 72/19550 | 0.002539239 | 0.048379325 | 0.043418644 | DSP/KCNA5/JUP/GJA1/TIAM1/DSC2/DLG1/SLC2A1/RAP2B | 9 |
| CC | GO:0001726 | ruffle | 16/797 | 178/19550 | 0.002602075 | 0.048379325 | 0.043418644 | PLCG2/SH3YL1/PLEKHA1/FERMT1/KANK1/TIAM1/ARFIP2/PLEK/ARHGEF26/SAMSN1/FGR/PARD6A/PODXL/RAC1/IQGAP1/ANXA2 | 16 |
| MF | GO:0032396 | inhibitory MHC class I receptor activity | 7/787 | 12/18368 | 1.69E-07 | 0.000137019 | 0.00012765 | LILRA5/LILRB2/LILRA6/LILRB1/LILRA1/LILRB3/LILRB5 | 7 |
| MF | GO:0032393 | MHC class I receptor activity | 7/787 | 17/18368 | 3.44E-06 | 0.001393108 | 0.001297854 | LILRA5/LILRB2/LILRA6/LILRB1/LILRA1/LILRB3/LILRB5 | 7 |
| MF | GO:0098632 | cell-cell adhesion mediator activity | 11/787 | 49/18368 | 5.46E-06 | 0.00147227 | 0.001371603 | CXADR/DSP/JUP/EPCAM/DSC2/CD200/TRIM29/KRT18/IGSF9/RAB10/ANXA2 | 11 |
| MF | GO:0004866 | endopeptidase inhibitor activity | 22/787 | 180/18368 | 9.55E-06 | 0.00162235 | 0.001511421 | DPEP1/SMR3B/CST7/SERPINB5/A2ML1/WFDC2/RENBP/SERPINB11/ITIH2/COL4A3/SPINK7/SERPINB13/TFPI2/RARRES1/ITIH3/PRNP/SLPI/LTF/SERPINA11/ANXA2/SERPINI2/AGT | 22 |
| MF | GO:0061135 | endopeptidase regulator activity | 23/787 | 194/18368 | 1.00E-05 | 0.00162235 | 0.001511421 | DPEP1/SMR3B/CST7/SERPINB5/NLRC4/A2ML1/WFDC2/RENBP/SERPINB11/ITIH2/COL4A3/SPINK7/SERPINB13/TFPI2/RARRES1/ITIH3/PRNP/SLPI/LTF/SERPINA11/ANXA2/SERPINI2/AGT | 23 |
| MF | GO:0030414 | peptidase inhibitor activity | 22/787 | 187/18368 | 1.76E-05 | 0.002367328 | 0.00220546 | DPEP1/SMR3B/CST7/SERPINB5/A2ML1/WFDC2/RENBP/SERPINB11/ITIH2/COL4A3/SPINK7/SERPINB13/TFPI2/RARRES1/ITIH3/PRNP/SLPI/LTF/SERPINA11/ANXA2/SERPINI2/AGT | 22 |
| MF | GO:0098631 | cell adhesion mediator activity | 11/787 | 59/18368 | 3.55E-05 | 0.004101019 | 0.00382061 | CXADR/DSP/JUP/EPCAM/DSC2/CD200/TRIM29/KRT18/IGSF9/RAB10/ANXA2 | 11 |
| MF | GO:0086080 | protein binding involved in heterotypic cell-cell adhesion | 5/787 | 11/18368 | 5.31E-05 | 0.005368606 | 0.005001524 | CXADR/DSP/JUP/DSC2/CD200 | 5 |
| MF | GO:0004867 | serine-type endopeptidase inhibitor activity | 14/787 | 98/18368 | 7.23E-05 | 0.006494969 | 0.006050873 | SERPINB5/A2ML1/WFDC2/SERPINB11/ITIH2/SPINK7/SERPINB13/TFPI2/ITIH3/SLPI/SERPINA11/ANXA2/SERPINI2/AGT | 14 |
| MF | GO:0061134 | peptidase regulator activity | 23/787 | 230/18368 | 0.00014669 | 0.011867203 | 0.011055777 | DPEP1/SMR3B/CST7/SERPINB5/NLRC4/A2ML1/WFDC2/RENBP/SERPINB11/ITIH2/COL4A3/SPINK7/SERPINB13/TFPI2/RARRES1/ITIH3/PRNP/SLPI/LTF/SERPINA11/ANXA2/SERPINI2/AGT | 23 |
| MF | GO:0044183 | protein folding chaperone | 8/787 | 40/18368 | 0.000248968 | 0.018310452 | 0.017058465 | HSPA1B/HSPA2/CLGN/HSPA13/DNAJB1/HSPA1L/DNAJB6/HSPA1A | 8 |
| MF | GO:0001618 | virus receptor activity | 11/787 | 76/18368 | 0.000374895 | 0.025274166 | 0.023546032 | CR2/CXCR4/CXADR/SLAMF1/HSPA1B/CLEC4M/CD86/CR1/ACE2/HSPA1A/CLDN1 | 11 |
| MF | GO:0140272 | exogenous protein binding | 11/787 | 77/18368 | 0.000420747 | 0.026183424 | 0.024393119 | CR2/CXCR4/CXADR/SLAMF1/HSPA1B/CLEC4M/CD86/CR1/ACE2/HSPA1A/CLDN1 | 11 |
| MF | GO:0140375 | immune receptor activity | 16/787 | 144/18368 | 0.000456542 | 0.026381628 | 0.024577771 | MPL/CR2/CXCR4/LILRA5/LILRB2/FLT3/LILRA6/LILRB1/IL20RB/LILRA1/CR1/LILRB3/LILRB5/CXCR1/IL7R/IL22RA2 | 16 |
| MF | GO:0004252 | serine-type endopeptidase activity | 18/787 | 174/18368 | 0.000497205 | 0.026815902 | 0.024982351 | TLL2/MMP3/PRSS57/MMP10/TMPRSS11D/GZMB/MMP1/MMP9/HTRA4/KLK11/KLK7/CTSS/TMPRSS11A/MMP7/TMPRSS13/TMPRSS11B/LTF/SEC11C | 18 |
| MF | GO:0031489 | myosin V binding | 5/787 | 17/18368 | 0.00057359 | 0.02900214 | 0.027019104 | RAB39B/RAB25/RAB3B/RAB3C/RAB10 | 5 |
| MF | GO:0038187 | pattern recognition receptor activity | 6/787 | 26/18368 | 0.000669173 | 0.030999734 | 0.028880112 | TLR9/CLEC4D/TLR2/FCN1/CLEC4E/MARCO | 6 |
| MF | GO:0017171 | serine hydrolase activity | 19/787 | 195/18368 | 0.000738687 | 0.030999734 | 0.028880112 | TLL2/AADAC/MMP3/PRSS57/MMP10/TMPRSS11D/GZMB/MMP1/MMP9/HTRA4/KLK11/KLK7/CTSS/TMPRSS11A/MMP7/TMPRSS13/TMPRSS11B/LTF/SEC11C | 19 |
| MF | GO:0045236 | CXCR chemokine receptor binding | 5/787 | 18/18368 | 0.000766372 | 0.030999734 | 0.028880112 | CXCL3/CXCL6/PF4V1/CXCL2/CXCL1 | 5 |
| MF | GO:0098641 | cadherin binding involved in cell-cell adhesion | 5/787 | 18/18368 | 0.000766372 | 0.030999734 | 0.028880112 | EPCAM/TRIM29/KRT18/RAB10/ANXA2 | 5 |
| MF | GO:0001540 | amyloid-beta binding | 11/787 | 84/18368 | 0.000892023 | 0.034364123 | 0.032014459 | ITM2C/LILRB2/CRYAB/TREM2/TLR2/ADRB2/C1QA/PRNP/BCHE/ITM2B/MARCO | 11 |
| MF | GO:0003953 | NAD+ nucleosidase activity | 6/787 | 28/18368 | 0.001017884 | 0.036565004 | 0.034064853 | TLR9/BANK1/TLR10/TLR2/CD38/TLR3 | 6 |
| MF | GO:0008009 | chemokine activity | 8/787 | 49/18368 | 0.001039549 | 0.036565004 | 0.034064853 | C10orf99/CXCL3/CXCL6/PF4V1/CCL13/CXCL2/CCL18/CXCL1 | 8 |
| MF | GO:0017022 | myosin binding | 10/787 | 74/18368 | 0.001181777 | 0.038503915 | 0.03587119 | RAB39B/CXCR4/RAB25/SHROOM1/RAB3B/AMPD1/ACTA1/RAB3C/MYL3/RAB10 | 10 |
| MF | GO:0005125 | cytokine activity | 21/787 | 235/18368 | 0.001225363 | 0.038503915 | 0.03587119 | IL24/SCG2/C10orf99/IL17F/FAM3B/IL26/IL21/LTB/BMP5/GDF6/TNFSF11/CXCL3/CMTM2/SLURP1/CXCL6/PF4V1/CCL13/CXCL2/CCL18/IL17A/CXCL1 | 21 |
| MF | GO:0051787 | misfolded protein binding | 6/787 | 29/18368 | 0.001237456 | 0.038503915 | 0.03587119 | HSPA1B/HSPA2/EDEM1/HSPA13/HSPA1L/HSPA1A | 6 |
| MF | GO:0008236 | serine-type peptidase activity | 18/787 | 191/18368 | 0.001472949 | 0.043549339 | 0.040571631 | TLL2/MMP3/PRSS57/MMP10/TMPRSS11D/GZMB/MMP1/MMP9/HTRA4/KLK11/KLK7/CTSS/TMPRSS11A/MMP7/TMPRSS13/TMPRSS11B/LTF/SEC11C | 18 |
| MF | GO:0005200 | structural constituent of cytoskeleton | 12/787 | 103/18368 | 0.00150727 | 0.043549339 | 0.040571631 | BFSP2/DSP/NEFM/NEFL/ACTA1/KRT16/KRT6B/CCDC6/KRT5/KRT14/TUBB4B/TUBA4A | 12 |
