# Supplementary material for: Bioinformatics investigation of adaptive immune‐related genes in peri‐implantitis and periodontitis: Characteristics and diagnostic values
Source: Immun Inflamm Dis. 2024 May 23;12(5):e1272. doi: 10.1002/iid3.1272 (PMC11112631; doi:10.1002/iid3.1272)
Supplement: Supplementary file 2 — Supporting information. [file IID3-12-e1272-s002.docx]

**Supplementary Table 2. KEGG enrichment analysis of** **differential expression genes in HI vs HP groups**

| **ID** | **Description** | **GeneRatio** | **BgRatio** | **pvalue** | **p.adjust** | **qvalue** | **geneID** | **Count** | **GeneRatio** |
| --- | --- | --- | --- | --- | --- | --- | --- | --- | --- |
| hsa05417 | Lipid and atherosclerosis | 21/365 | 215/8159 | 0.000608 | 0.035982 | 0.034293 | MMP3/PPARG/CYBA/NCF4/HSPA1B/HSPA2/VAV1/TLR2/CXCL3/CYP2J2/MMP1/PIK3CD/MMP9/CALML5/HSPA1L/XBP1/HSPA1A/RAC1/CXCL2/CYBB/CXCL1 | 21 | 0.0575 |
| hsa04670 | Leukocyte transendothelial migration | 17/365 | 114/8159 | 1.10E-05 | 0.002034 | 0.001939 | PLCG2/RHOH/CXCR4/CYBA/NCF4/CLDN3/ITGA4/VAV1/PIK3CD/MMP9/CLDN10/ITK/OCLN/RAC1/PECAM1/CYBB/CLDN1 | 17 | 0.0466 |
| hsa04380 | Osteoclast differentiation | 15/365 | 128/8159 | 0.000564 | 0.035982 | 0.034293 | PLCG2/PPARG/CYBA/LILRA5/LILRB2/NCF4/TREM2/LILRA6/TNFSF11/LILRB1/PIK3CD/LILRA1/LILRB3/LILRB5/RAC1 | 15 | 0.0411 |
| hsa04662 | B cell receptor signaling pathway | 14/365 | 82/8159 | 1.37E-05 | 0.002034 | 0.001939 | CD19/CD79A/PLCG2/CR2/LILRA5/LILRB2/LILRA6/VAV1/LILRB1/PIK3CD/LILRA1/LILRB3/LILRB5/RAC1 | 14 | 0.0384 |
| hsa05323 | Rheumatoid arthritis | 12/365 | 93/8159 | 0.000844 | 0.041623 | 0.039669 | MMP3/LTB/TNFSF11/TLR2/CXCL3/MMP1/CTLA4/CXCL6/CD86/CXCL2/IL17A/CXCL1 | 12 | 0.0329 |
| hsa05134 | Legionellosis | 10/365 | 57/8159 | 0.000186 | 0.018398 | 0.017534 | HSPA1B/HSPA2/TLR2/NLRC4/CXCL3/HSPA1L/CR1/HSPA1A/CXCL2/CXCL1 | 10 | 0.0274 |
