# Supplementary material for: Bioinformatics investigation of adaptive immune‐related genes in peri‐implantitis and periodontitis: Characteristics and diagnostic values
Source: Immun Inflamm Dis. 2024 May 23;12(5):e1272. doi: 10.1002/iid3.1272 (PMC11112631; doi:10.1002/iid3.1272)
Supplement: Supplementary file 3 — Supporting information. [file IID3-12-e1272-s009.docx]

**Supplementary Table 3. GO enrichment analysis of** **differential expression genes in PI vs HP groups**

| **ONTOLOGY** | **ID** | **Description** | **GeneRatio** | **BgRatio** | **pvalue** | **p.adjust** | **qvalue** | **geneID** | **Count** |
| --- | --- | --- | --- | --- | --- | --- | --- | --- | --- |
| BP | GO:1903131 | mononuclear cell differentiation | 77/1271 | 426/18723 | 1.78E-15 | 9.69E-12 | 7.98E-12 | XBP1/RHOH/CD19/POU2AF1/CTLA4/SLAMF1/TREM2/IRF4/LILRB1/IKZF3/BATF/CD3D/CR2/GPR18/PSMB11/F2RL1/IL31RA/TMEM176B/ITGA4/CD86/TCIRG1/CD79A/CTNNBIP1/VAV1/CD80/PIK3CD/FZD5/PTK2B/ADAM8/FLT3/TMEM176A/ZMIZ1/CD3G/GPR183/DNAJB9/MYC/PREX1/PIR/PLA2G2D/IL1B/MERTK/PLCG2/NRARP/CR1/LILRB2/RORC/MS4A1/HLX/AIRE/IL7R/EOMES/BMP4/ITK/IL2RA/PCK1/TNFSF8/ADA/NCKAP1L/ATM/IL21/PRDM1/CTSL/TLR9/CLPTM1/PPARG/IL6/TOX/MT1G/RARA/CD74/FCER1G/ITGB8/PTPRC/ID2/LEPR/BATF2/JUN | 77 |
| BP | GO:0002764 | immune response-regulating signaling pathway | 75/1271 | 468/18723 | 2.43E-12 | 3.31E-09 | 2.73E-09 | CYBA/CD19/BTLA/STAP1/KLHL6/CTLA4/LAT2/MYO1G/TREM2/IRF4/LILRB1/IRAK2/THEMIS2/SKAP1/IGLL1/TLR10/CR2/F2RL1/CD226/FCN1/CTSS/CD38/CD79A/BANK1/VAV1/ARRB2/CD14/PIK3CD/RAB11FIP2/FFAR2/LY96/LPXN/TYROBP/LGR4/HSPA1B/LAX1/CD247/PLCG2/PAK3/CR1/PDE4B/LILRB2/WNK1/MNDA/MS4A1/TLR1/BIRC3/LILRA2/FPR1/LTF/PRKCB/FGR/CLEC4E/PLEKHA1/ITK/SLC15A2/CTSH/BTN3A1/ADA/NCKAP1L/TLR2/TLR9/TLR6/MAP3K1/FPR2/C3AR1/TNIP3/S100A14/TLR3/FCER1G/HSPA1A/LBP/IL20RB/PTPRC/TLR4 | 75 |
| BP | GO:0001819 | positive regulation of cytokine production | 73/1271 | 467/18723 | 1.61E-11 | 1.29E-08 | 1.06E-08 | CYBA/XBP1/IL26/POU2AF1/SLAMF1/TREM2/IRF4/LILRB1/BATF/F2RL1/NLRC4/CD226/FCN1/CD86/CD80/SLC7A5/CD14/AIM2/PIK3CD/IL17F/LILRA5/FFAR2/FZD5/ADAM8/LY96/AIF1/PLA2G3/TYROBP/HSPA1B/MAPK13/C3/CD200/IL1B/CLECL1/PLCG2/PDE4B/LILRB2/LTB/MNDA/TLR1/IL17A/AIRE/PANX2/LILRA2/SERPINB7/POU2F2/FGR/ITK/SEMA7A/CYBB/BTN3A1/AGT/PIK3CG/IL21/SAA1/TLR2/TLR9/TLR6/IL6/C3AR1/RARA/CD74/MMP12/TLR3/FCER1G/IL17D/FERMT1/HSPA1A/LBP/IL20RB/PTPRC/TLR4/PAEP | 73 |
| BP | GO:0019221 | cytokine-mediated signaling pathway | 73/1271 | 472/18723 | 2.69E-11 | 1.63E-08 | 1.35E-08 | TNFRSF17/ZBP1/STK39/STAP1/TREM2/CXCL6/LILRB1/TNFRSF25/IRAK2/CXCL1/MST1R/F2RL1/LILRA1/IL31RA/SHARPIN/CCL18/MPL/LILRB3/GHR/AIM2/KRT8/CCR3/IL17F/LILRA5/CXCL3/PTK2B/IL17RD/EDA2R/FLT3/HSPA1B/CXCR4/TNFSF11/IL1B/LILRB2/LILRA6/WNK1/IL2RG/CXCR1/BIRC3/IL17A/ACKR4/KRT18/CBL/CXCL2/STAT5A/LILRA2/IL7R/IL22RA2/IL2RA/CCL13/CXCL13/PF4V1/PRLR/CRLF1/IL20RA/PPARG/MX1/IL6/CCL5/IFI27/CD74/MMP12/FCER1G/IL1F10/HSPA1A/IL20RB/CCL11/CXCL12/PTPRC/IL37/LEPR/EDN2/TNFRSF4 | 73 |
| BP | GO:0050900 | leukocyte migration | 67/1271 | 369/18723 | 1.01E-13 | 2.74E-10 | 2.26E-10 | STK39/RHOH/STAP1/SLAMF1/MYO1G/TREM2/TRIM55/CHST2/CXCL6/DPEP1/CXCL1/GPR18/F2RL1/ITGA4/CCL18/VAV1/SELL/SCG2/PIK3CD/CXCL3/FFAR2/PTK2B/ADAM8/AIF1/MIA3/CXCR4/GPR183/TNFSF11/TREM1/PREX1/CD200/BMP5/PDE4B/WNK1/CXCR1/IL17A/SELP/CXCL2/AIRE/PLA2G7/PTN/PECAM1/SELPLG/CCL13/CXCL13/C10orf99/PF4V1/ADA/PIK3CG/NCKAP1L/SAA1/MMP9/RET/IL6/FPR2/C3AR1/CCL5/CXADR/CD74/S100A14/FCER1G/LBP/CCL11/CXCL12/CD9/F11R/EDN2 | 67 |
| BP | GO:0042110 | T cell activation | 65/1271 | 487/18723 | 1.22E-07 | 1.73E-05 | 1.42E-05 | XBP1/RHOH/CTLA4/SLAMF1/IRF4/LILRB1/RASAL3/BATF/CD3D/SLAMF7/GPR18/PSMB11/F2RL1/CD86/TCIRG1/VAV1/CD80/PIK3CD/FZD5/ADAM8/AIF1/ZMIZ1/CD3G/GPR183/LAX1/TNFSF11/PREX1/PLA2G2D/IL1B/CLECL1/NRARP/CR1/LILRB2/RORC/HLX/AIRE/IL7R/EOMES/BMP4/ITK/IL2RA/PCK1/TNFSF8/BTN3A1/ADA/PIK3CG/NCKAP1L/HES1/IL21/PRDM1/CTSL/CLPTM1/IL6/TOX/CCL5/CXADR/RARA/CD74/GPNMB/FCER1G/IL20RB/PTPRC/VSIG4/LEPR/TNFRSF4 | 65 |
| BP | GO:0030098 | lymphocyte differentiation | 63/1271 | 374/18723 | 1.65E-11 | 1.29E-08 | 1.06E-08 | XBP1/RHOH/CD19/POU2AF1/CTLA4/SLAMF1/IRF4/IKZF3/BATF/CD3D/CR2/GPR18/PSMB11/ITGA4/CD86/TCIRG1/CD79A/VAV1/CD80/PIK3CD/FZD5/PTK2B/ADAM8/FLT3/ZMIZ1/CD3G/GPR183/DNAJB9/PREX1/PLA2G2D/IL1B/MERTK/PLCG2/NRARP/CR1/LILRB2/RORC/MS4A1/HLX/AIRE/IL7R/EOMES/BMP4/ITK/IL2RA/PCK1/TNFSF8/ADA/NCKAP1L/ATM/IL21/PRDM1/CTSL/TLR9/CLPTM1/IL6/TOX/RARA/CD74/FCER1G/PTPRC/ID2/LEPR | 63 |
| BP | GO:0032103 | positive regulation of response to external stimulus | 63/1271 | 427/18723 | 4.25E-09 | 1.05E-06 | 8.69E-07 | ZBP1/STK39/CYBA/STAP1/SLAMF1/TREM2/CD180/TLR10/F2RL1/NLRC4/CD226/FCN1/VAV1/SCG2/AIM2/IL17F/LILRA5/FFAR2/PTK2B/ADAM8/LY96/AIF1/PLA2G3/TYROBP/CXCR4/TGM2/MAPK13/C3/TNFSF11/IL1B/PLCG2/PAK3/ALOX5AP/WNK1/MNDA/IL17A/PGC/LILRA2/PLA2G7/TIAM1/PTN/CXCL13/AGT/PIK3CG/NCKAP1L/IL21/TLR2/TLR9/IL6/BMP6/FPR2/C3AR1/CCL5/KLK7/CD74/MMP12/S100A14/TLR3/NPY/LBP/CXCL12/TLR4/EDN2 | 63 |
| BP | GO:0002683 | negative regulation of immune system process | 61/1271 | 434/18723 | 4.65E-08 | 8.18E-06 | 6.74E-06 | STAP1/CTLA4/SLAMF1/TREM2/TARBP2/LILRB1/CST7/CR2/GPR18/SAMSN1/IL31RA/CR1L/TMEM176B/CD86/LRFN5/LILRB3/BANK1/ARRB2/CD80/SUSD4/TAPBPL/LPXN/TMEM176A/MIA3/TYROBP/LAX1/MYC/CD200/PLA2G2D/MERTK/NRARP/BMP5/CR1/LILRB2/MNDA/HLX/TRIB1/IL7R/LTF/FGR/BMP4/IL2RA/ADA/SOX11/NCKAP1L/ATM/GPR137/PPARG/RARA/CD74/MMP12/TLR3/GPNMB/IL17D/IL20RB/CXCL12/PTPRC/VSIG4/TLR4/ID2/SERPINB4 | 61 |
| BP | GO:1903706 | regulation of hemopoiesis | 59/1271 | 367/18723 | 4.86E-10 | 2.04E-07 | 1.68E-07 | XBP1/RHOH/CTLA4/TREM2/IRF4/LILRB1/IKZF3/ACVR1B/HCLS1/TMEM176B/CD86/KLF13/MPL/CTNNBIP1/LILRB3/CD80/PTK2B/ADAM8/PITHD1/PLA2G3/TMEM176A/ZMIZ1/TYROBP/RASSF2/HSPA1B/TNFSF11/MYC/NRARP/CR1/LILRB2/OCSTAMP/MEIS1/HLX/IL17A/TRIB1/IL7R/EIF6/PTN/LTF/BMP4/IL2RA/PCK1/JAG1/ADA/NCKAP1L/PRDM1/GPR137/TLR9/CLPTM1/TOX/RARA/CD74/TLR3/IL17D/HSPA1A/PTPRC/TLR4/ID2/JUN | 59 |
| BP | GO:0022407 | regulation of cell-cell adhesion | 59/1271 | 448/18723 | 7.30E-07 | 7.23E-05 | 5.96E-05 | XBP1/RHOH/CTLA4/SLAMF1/CHST2/LILRB1/RASAL3/SKAP1/EPHB3/ITGA4/CD86/VAV1/CD80/ZDHHC2/ADAM8/AIF1/EFNA5/AKNA/MIA3/ZMIZ1/LAX1/TNFSF11/EPCAM/PLA2G2D/SOX2/IL1B/CLECL1/NRARP/CR1/LILRB2/WNK1/HLX/SELP/IL7R/PODXL/BMP4/IL2RA/PCK1/CXCL13/JAG1/ADA/NCKAP1L/HES1/IL21/CELSR2/IL6/BMP6/CCL5/KLF4/RARA/CD74/GPNMB/IL20RB/CXCL12/PTPRC/VSIG4/CD9/F11R/CDH1 | 59 |
| BP | GO:0002237 | response to molecule of bacterial origin | 57/1271 | 363/18723 | 2.35E-09 | 6.74E-07 | 5.55E-07 | NUGGC/XBP1/STAP1/TREM2/CXCL6/LILRB1/IRAK2/CD180/CXCL1/IL24/CD86/CD80/CD14/CXCL3/FZD5/LY96/KCNJ8/IL1B/PLCG2/PDE4B/LILRB2/STAR/TLR1/SELP/TRIB1/CXCL2/LILRA2/FGFR2/LTF/PCK1/CXCL13/PTGIR/PENK/PF4V1/TLR2/GJA1/TLR9/PTGES/TLR6/IL6/BMP6/CYP27B1/GGT7/CAMP/CCL5/RARA/TNIP3/S100A14/IL1F10/LBP/CEBPE/GJB6/MPO/TLR4/IL37/ANKRD1/JUN | 57 |
| BP | GO:0002253 | activation of immune response | 57/1271 | 375/18723 | 7.90E-09 | 1.66E-06 | 1.36E-06 | ZBP1/CD19/STAP1/KLHL6/CTLA4/LAT2/MYO1G/TREM2/KRT1/THEMIS2/SKAP1/IGLL1/CR2/NLRC4/CD226/CR1L/FCN1/CD38/CD79A/BANK1/VAV1/SUSD4/AIM2/PIK3CD/FCN2/FFAR2/LPXN/TYROBP/FCN3/LAX1/CD247/C3/C1QB/IL1B/PLCG2/PAK3/CR1/PDE4B/WNK1/MNDA/MS4A1/LILRA2/C1QA/FPR1/PRKCB/FGR/PLEKHA1/ITK/BTN3A1/ADA/NCKAP1L/FPR2/C3AR1/FCER1G/PTPRC/VSIG4/TLR4 | 57 |
| BP | GO:0043410 | positive regulation of MAPK cascade | 57/1271 | 480/18723 | 2.64E-05 | 0.001384 | 0.001141 | STK39/IL26/TBX1/SLAMF1/TREM2/P2RY1/EGF/MST1R/F2RL1/CCL18/GHR/BANK1/ARRB2/LILRA5/FZD5/PTK2B/ADAM8/EDA2R/FLT3/RASSF2/ADRB2/FGF19/GPR183/TNFSF11/SOX2/IL1B/FGFR4/PLCG2/MYDGF/ERN1/FGFR2/TIAM1/ARHGEF5/BMP4/CCL13/WNT16/SEMA7A/AJUBA/PIK3CG/GPR37/EFNA1/TLR9/FZD10/TLR6/RET/IL6/FPR2/MARCO/CCL5/MID1/CD74/TLR3/GPNMB/CCL11/PTPRC/TLR4/DKK1 | 57 |
| BP | GO:0050727 | regulation of inflammatory response | 56/1271 | 386/18723 | 5.58E-08 | 9.35E-06 | 7.71E-06 | ZBP1/STK39/STAP1/TREM2/F12/KRT1/MMP3/CST7/TLR10/SHARPIN/LRFN5/LILRA5/FFAR2/ADAM8/PLA2G3/AKNA/TNFAIP6/TGM2/MAPK13/C3/TNFSF11/CD200/PLA2G2D/IL1B/PLCG2/ALOX5AP/BIRC3/PLA2G7/SIGLEC10/FGR/IL22RA2/IL2RA/ACE2/SEMA7A/ADA/AGT/PIK3CG/IL21/SAA1/ABHD12/TLR2/TLR9/MMP9/NR1D2/PPARG/PTGES/IL6/FPR2/CCL5/KLF4/TLR3/LBP/IL20RB/PTPRC/TLR4/IL37 | 56 |
| BP | GO:0002443 | leukocyte mediated immunity | 55/1271 | 440/18723 | 8.39E-06 | 0.000538 | 0.000444 | CD19/STAP1/SLAMF1/LAT2/MYO1G/TREM2/ARL8B/CXCL6/LILRB1/IGLL1/BATF/SLAMF7/CR2/F2RL1/CD226/CR1L/TCIRG1/VAV1/ARRB2/SUSD4/PIK3CD/FZD5/PLA2G3/TYROBP/PRF1/C3/TREM1/C1QB/IL1B/PLCG2/CR1/GZMB/CBL/AIRE/IL7R/C1QA/FGR/CTSH/JAG1/PIK3CG/NCKAP1L/IL21/GAPT/CD1E/IL6/CD74/TLR3/FCER1G/EMP2/IL20RB/PTPRC/TLR4/GZMM/PTGDS/SERPINB4 | 55 |
| BP | GO:0060326 | cell chemotaxis | 54/1271 | 310/18723 | 1.30E-10 | 7.10E-08 | 5.86E-08 | STK39/STAP1/SLAMF1/CXCL6/DPEP1/CXCL1/GPR18/F2RL1/CCL18/VAV1/BIN2/ARRB2/SCG2/PIK3CD/CCR3/CXCL3/FFAR2/PTK2B/ADAM8/AIF1/CXCR4/GPR183/TNFSF11/TREM1/PREX1/PDE4B/WNK1/CXCR1/ACKR4/CXCL2/PLA2G7/TIAM1/PTN/CCL13/CXCL13/CORO1B/C10orf99/PF4V1/PIK3CG/NCKAP1L/SAA1/DOCK4/IL6/FPR2/C3AR1/CCL5/CXADR/CD74/S100A14/FCER1G/LBP/CCL11/CXCL12/EDN2 | 54 |
| BP | GO:0070661 | leukocyte proliferation | 54/1271 | 318/18723 | 3.46E-10 | 1.71E-07 | 1.41E-07 | NPR3/CD19/CTLA4/SLAMF1/MZB1/TREM2/LILRB1/IKZF3/RASAL3/CD180/CR2/F2RL1/CD86/TCIRG1/CD38/CD79A/MPL/CD80/AIF1/FLT3/TYROBP/GPR183/TNFSF11/PLA2G2D/IL1B/CLECL1/CR1/LILRB2/OCSTAMP/MNDA/MS4A1/IL7R/BMP4/IL2RA/TNFSF8/BTN3A1/ADA/SOX11/PIK3CG/NCKAP1L/ATM/HES1/IL21/GAPT/TLR9/IL6/CCL5/CD74/GPNMB/IL20RB/PTPRC/VSIG4/TLR4/TNFRSF4 | 54 |
| BP | GO:0008544 | epidermis development | 54/1271 | 324/18723 | 6.99E-10 | 2.72E-07 | 2.24E-07 | SOX21/DCT/KRT85/CALML5/KRT10/ACVR1B/SHARPIN/ALDH3A2/KRT2/FLG2/HES5/EDA2R/OVOL3/LGR4/SLITRK6/ERCC3/LIPK/CLIC4/KDF1/KRT27/FGFR2/EXPH5/GJB5/MCOLN3/BMP4/DSP/WNT16/JAG1/HES1/FOXE1/GRHL2/KRT76/ZBED2/MYO6/ESRP1/TCHH/CRABP2/CYP27B1/BNC1/IRF6/KLF4/KLK7/TP63/LCE3B/PPL/FERMT1/SFRP4/KRT16/SOSTDC1/LCE3D/FZD6/AQP3/LCE3E/LCE3A | 54 |
| BP | GO:0050867 | positive regulation of cell activation | 54/1271 | 420/18723 | 4.42E-06 | 0.000317 | 0.000261 | SPACA3/XBP1/RHOH/STAP1/SLAMF1/TREM2/LILRB1/RASAL3/IGLL1/CLEC4D/F2RL1/CD226/CD86/CD38/MPL/VAV1/CD80/LILRA5/ADAM8/AIF1/PLA2G3/ZMIZ1/TYROBP/PLEK/SOX15/GPR183/TNFSF11/IL1B/CLECL1/CR1/LILRB2/SH3KBP1/HLX/SELP/LILRA2/IL7R/FGR/IL2RA/PCK1/ADA/NCKAP1L/HES1/IL21/TLR9/TLR6/IL6/TOX/CCL5/RARA/CD74/LBP/PTPRC/TLR4/TNFRSF4 | 54 |
| BP | GO:0055074 | calcium ion homeostasis | 54/1271 | 460/18723 | 5.83E-05 | 0.002362 | 0.001947 | JSRP1/CYBA/CD19/P2RY1/NPY2R/P2RX1/GPR18/F2RL1/KCNA5/PROK2/P2RX5/TCIRG1/P2RY10/CD38/CCR3/PTK2B/SLC35G1/GPR65/CXCR4/TGM2/TNFSF11/HERPUD1/PLCG2/CD52/CXCR1/MS4A1/ACKR4/ANXA6/FPR1/PRKCB/RYR1/MCOLN3/CCL13/CXCL13/PTGIR/SLC24A5/AGT/PIK3CG/GPR174/SAA1/GJA1/LPAR6/NMU/SLC8A2/FPR2/CYP27B1/C3AR1/CCL5/S100A14/CCL11/CXCL12/PTPRC/JPH1/EDN2 | 54 |
| BP | GO:0032496 | response to lipopolysaccharide | 53/1271 | 343/18723 | 1.50E-08 | 2.92E-06 | 2.41E-06 | NUGGC/XBP1/STAP1/TREM2/CXCL6/LILRB1/IRAK2/CD180/CXCL1/IL24/CD86/CD80/CD14/CXCL3/LY96/KCNJ8/IL1B/PLCG2/PDE4B/LILRB2/STAR/SELP/TRIB1/CXCL2/LILRA2/FGFR2/LTF/PCK1/CXCL13/PTGIR/PENK/PF4V1/TLR2/GJA1/PTGES/IL6/BMP6/CYP27B1/GGT7/CAMP/CCL5/RARA/TNIP3/S100A14/IL1F10/LBP/CEBPE/GJB6/MPO/TLR4/IL37/ANKRD1/JUN | 53 |
| BP | GO:0033674 | positive regulation of kinase activity | 53/1271 | 467/18723 | 0.000163 | 0.004869 | 0.004013 | CD19/STAP1/TREM2/CHRNA3/MAPT/EGF/MST1R/EPHB3/CD86/GHR/CCND2/LILRA5/FZD5/PTK2B/ADAM8/EFNA5/FLT3/RASSF2/ADRB2/TNFSF11/IL1B/FGFR4/MERTK/WNK1/ERN1/MAPRE3/FGFR2/TIAM1/ARHGEF5/LTF/FGR/AJUBA/AGT/PIK3CG/PRLR/NCKAP1L/EFNA1/TLR9/FZD10/TLR6/RET/VLDLR/SLC8A2/FPR2/CCL5/CD74/EEF1A2/DYNAP/TLR3/EMP2/PTPRC/TLR4/DKK1 | 53 |
| BP | GO:0072503 | cellular divalent inorganic cation homeostasis | 53/1271 | 486/18723 | 0.000433 | 0.009588 | 0.007902 | JSRP1/CYBA/CD19/P2RY1/NPY2R/P2RX1/GPR18/F2RL1/KCNA5/PROK2/P2RX5/TCIRG1/P2RY10/CD38/CCR3/PTK2B/SLC35G1/GPR65/CXCR4/TGM2/HERPUD1/PLCG2/CD52/CXCR1/MS4A1/ACKR4/ANXA6/FPR1/PRKCB/RYR1/MCOLN3/CCL13/CXCL13/PTGIR/SLC24A5/AGT/PIK3CG/GPR174/SAA1/GJA1/LPAR6/MT1X/NMU/SLC8A2/FPR2/C3AR1/CCL5/MT1G/CCL11/CXCL12/PTPRC/JPH1/EDN2 | 53 |
| BP | GO:0050673 | epithelial cell proliferation | 52/1271 | 437/18723 | 5.57E-05 | 0.002319 | 0.001911 | CYBA/XBP1/IL26/TBX1/EGF/EAF2/DLX5/PROK2/ITGA4/KRT2/SCG2/PIK3CD/CCR3/HES5/LGR4/TNFSF11/MYC/SOX2/NRARP/BMP5/TNMD/KDF1/MYDGF/ERN1/STAT5A/FGFR2/SLURP1/PTN/BMP4/MARVELD3/WNT16/SOX11/SGPP2/HES1/TGFBR3/GJA1/PPARG/IL6/BMP6/FGFBP1/FABP7/IRF6/EHF/TP63/SERPINB5/MMP12/FERMT1/CCL11/CXCL12/SFRP2/APLN/ID2 | 52 |
| BP | GO:0002697 | regulation of immune effector process | 51/1271 | 339/18723 | 6.77E-08 | 9.97E-06 | 8.22E-06 | XBP1/STAP1/SLAMF1/MZB1/TREM2/CXCL6/IRF4/LILRB1/CR2/F2RL1/CD226/CR1L/CD86/VAV1/ARRB2/CD80/SLC7A5/SUSD4/IL17F/FFAR2/FZD5/PLA2G3/TYROBP/DNAJB9/C3/IL1B/PLCG2/CR1/HLX/IL17A/PGC/IL7R/FGR/PCK1/SEMA7A/NCKAP1L/IL21/TLR9/CD1E/IL6/RARA/KLK7/CD74/TLR3/LBP/IL20RB/PTPRC/VSIG4/TLR4/SERPINB4/TNFRSF4 | 51 |
| BP | GO:0045785 | positive regulation of cell adhesion | 51/1271 | 437/18723 | 0.000107 | 0.003591 | 0.00296 | XBP1/RHOH/SLAMF1/CHST2/LILRB1/VIT/NPY2R/RASAL3/SKAP1/ITGA4/CD86/VAV1/CD80/PTK2B/ADAM8/AIF1/ZMIZ1/TGM2/TNFSF11/PREX1/SOX2/IL1B/CLECL1/CR1/LILRB2/HLX/SELP/IL7R/PTN/PODXL/IL2RA/PCK1/CXCL13/ADA/NCKAP1L/ATM/HES1/IL21/SAA1/RET/IL6/JUP/CCL5/RARA/CD74/FERMT1/EMP2/CXCL12/PTPRC/SFRP2/F11R | 51 |
| BP | GO:0006874 | cellular calcium ion homeostasis | 51/1271 | 448/18723 | 0.000199 | 0.005647 | 0.004655 | JSRP1/CYBA/CD19/P2RY1/NPY2R/P2RX1/GPR18/F2RL1/KCNA5/PROK2/P2RX5/TCIRG1/P2RY10/CD38/CCR3/PTK2B/SLC35G1/GPR65/CXCR4/TGM2/HERPUD1/PLCG2/CD52/CXCR1/MS4A1/ACKR4/ANXA6/FPR1/PRKCB/RYR1/MCOLN3/CCL13/CXCL13/PTGIR/SLC24A5/AGT/PIK3CG/GPR174/SAA1/GJA1/LPAR6/NMU/SLC8A2/FPR2/C3AR1/CCL5/CCL11/CXCL12/PTPRC/JPH1/EDN2 | 51 |
| BP | GO:0052547 | regulation of peptidase activity | 51/1271 | 461/18723 | 0.000394 | 0.009121 | 0.007517 | CCK/DPEP1/MAPT/P2RX1/SERPINI1/CST7/DNAJB6/NLRC4/RARRES1/CRYAB/ARRB2/AIM2/SERPINA11/SIAH2/CASP10/C3/MYC/SOX2/HERPUD1/SMR3B/SERPINI2/TFAP4/CR1/SERPINB10/BIRC3/SPINK1/SERPINB7/LTF/TFPI2/CTSH/AGT/TIMP1/EFNA1/MMP9/SERPINB11/WFDC2/PPARG/SPINK7/CLDN3/DAP/KLF4/TP63/SERPINB5/SPINK9/A2ML1/DHCR24/PERP/PI3/SFRP2/SERPINB4/SERPINB13 | 51 |
| BP | GO:0007159 | leukocyte cell-cell adhesion | 50/1271 | 371/18723 | 2.61E-06 | 0.000219 | 0.00018 | XBP1/RHOH/CTLA4/SLAMF1/CHST2/LILRB1/RASAL3/SKAP1/ITGA4/CD86/VAV1/CD80/SELL/ADAM8/AIF1/MIA3/ZMIZ1/LAX1/TNFSF11/PLA2G2D/IL1B/CLECL1/NRARP/CR1/LILRB2/WNK1/HLX/SELP/IL7R/PECAM1/CLEC4M/SELPLG/BMP4/IL2RA/PCK1/ADA/NCKAP1L/HES1/IL21/IL6/CCL5/KLF4/RARA/CD74/GPNMB/IL20RB/CXCL12/PTPRC/VSIG4/F11R | 50 |
| BP | GO:0030099 | myeloid cell differentiation | 50/1271 | 381/18723 | 5.58E-06 | 0.000381 | 0.000314 | ZNF385A/TREM2/IRF4/LILRB1/BATF/ACVR1B/F2RL1/IL31RA/HCLS1/TCIRG1/KLF13/MPL/CTNNBIP1/LILRB3/PIK3CD/PTK2B/PITHD1/PLA2G3/TYROBP/RASSF2/HSPA1B/GPR183/TNFSF11/MYC/PIR/OCSTAMP/MEIS1/IL17A/TRIB1/EIF6/LTF/BMP4/JAG1/NCKAP1L/TGFBR3/TLR2/GPR137/MMP9/PPARG/MT1G/RARA/CD74/TLR3/HSPA1A/ITGB8/CEBPE/TLR4/ID2/BATF2/JUN | 50 |
| BP | GO:0052548 | regulation of endopeptidase activity | 50/1271 | 432/18723 | 0.000153 | 0.004666 | 0.003846 | CCK/DPEP1/MAPT/P2RX1/SERPINI1/CST7/DNAJB6/NLRC4/RARRES1/CRYAB/ARRB2/AIM2/SERPINA11/SIAH2/CASP10/C3/MYC/SOX2/HERPUD1/SMR3B/SERPINI2/TFAP4/CR1/SERPINB10/BIRC3/SPINK1/SERPINB7/LTF/TFPI2/CTSH/AGT/TIMP1/EFNA1/MMP9/SERPINB11/WFDC2/PPARG/SPINK7/DAP/KLF4/TP63/SERPINB5/SPINK9/A2ML1/DHCR24/PERP/PI3/SFRP2/SERPINB4/SERPINB13 | 50 |
| BP | GO:0002696 | positive regulation of leukocyte activation | 49/1271 | 409/18723 | 7.63E-05 | 0.00279 | 0.0023 | SPACA3/XBP1/RHOH/STAP1/SLAMF1/TREM2/LILRB1/RASAL3/IGLL1/CLEC4D/F2RL1/CD226/CD86/CD38/MPL/VAV1/CD80/ADAM8/AIF1/PLA2G3/ZMIZ1/TYROBP/GPR183/TNFSF11/IL1B/CLECL1/CR1/LILRB2/SH3KBP1/HLX/IL7R/FGR/IL2RA/PCK1/ADA/NCKAP1L/HES1/IL21/TLR9/TLR6/IL6/TOX/CCL5/RARA/CD74/LBP/PTPRC/TLR4/TNFRSF4 | 49 |
| BP | GO:0031667 | response to nutrient levels | 49/1271 | 474/18723 | 0.002179 | 0.030457 | 0.025102 | SLC6A4/CCK/CYBA/XBP1/UCP1/NUAK2/TYR/BCHE/ULK2/GHR/OXCT1/MN1/WIPI1/ADRB2/UCP2/DAPL1/MAP1LC3B/SLC2A1/STAR/KRT18/CBL/RRAGD/BHLHA15/PTN/MC4R/ARSB/NQO1/PCK1/SST/PENK/WNT2B/CYBB/ADA/KYNU/ADRB1/NUCB2/PPARG/CPEB4/DAP/CYP27B1/RARA/HMGCS1/NPY/MPO/SFRP2/AQP3/MMP7/SLC38A2/JUN | 49 |
| BP | GO:0002274 | myeloid leukocyte activation | 48/1271 | 223/18723 | 5.84E-13 | 1.06E-09 | 8.74E-10 | SPACA3/RHOH/STAP1/SLAMF1/LAT2/TREM2/CXCL6/IRF4/MAPT/CST7/BATF/CLEC4D/F2RL1/CD226/IL31RA/LRFN5/PIK3CD/AIF1/PLA2G3/TYROBP/PREX1/CD200/PLCG2/TLR1/CBL/LILRA2/C1QA/FGR/PIK3CG/TLR2/TLR6/NDRG1/IL6/FPR2/CD93/CCL5/MT1G/CD74/TLR3/FCER1G/LBP/ITGB8/PTPRC/VSIG4/TLR4/PTGDS/EDN2/BATF2 | 48 |
| BP | GO:0046651 | lymphocyte proliferation | 48/1271 | 288/18723 | 6.18E-09 | 1.42E-06 | 1.17E-06 | CD19/CTLA4/SLAMF1/MZB1/LILRB1/IKZF3/RASAL3/CD180/CR2/CD86/CD38/CD79A/MPL/CD80/AIF1/FLT3/TYROBP/GPR183/PLA2G2D/IL1B/CLECL1/CR1/LILRB2/MNDA/MS4A1/IL7R/BMP4/IL2RA/TNFSF8/BTN3A1/ADA/SOX11/PIK3CG/NCKAP1L/ATM/HES1/IL21/GAPT/TLR9/IL6/CCL5/CD74/GPNMB/IL20RB/PTPRC/VSIG4/TLR4/TNFRSF4 | 48 |
| BP | GO:0032943 | mononuclear cell proliferation | 48/1271 | 291/18723 | 8.71E-09 | 1.76E-06 | 1.45E-06 | CD19/CTLA4/SLAMF1/MZB1/LILRB1/IKZF3/RASAL3/CD180/CR2/CD86/CD38/CD79A/MPL/CD80/AIF1/FLT3/TYROBP/GPR183/PLA2G2D/IL1B/CLECL1/CR1/LILRB2/MNDA/MS4A1/IL7R/BMP4/IL2RA/TNFSF8/BTN3A1/ADA/SOX11/PIK3CG/NCKAP1L/ATM/HES1/IL21/GAPT/TLR9/IL6/CCL5/CD74/GPNMB/IL20RB/PTPRC/VSIG4/TLR4/TNFRSF4 | 48 |
| BP | GO:0048608 | reproductive structure development | 48/1271 | 424/18723 | 0.000344 | 0.008324 | 0.006861 | ROBO2/DNAJB6/EAF2/RHOBTB3/PRDX4/ARRB2/KRT8/PLCD3/FZD5/LGR4/SOX15/PSAPL1/C3/MERTK/BMP5/STAR/CBL/STRA6/FGFR2/PTN/EOMES/GJB5/BCL2L1/PLEKHA1/BMP4/NEUROG1/WNT2B/ADA/HES1/PRDM1/GJA1/LHX3/GRHL2/PPARG/BMP6/CYP27B1/E2F8/RARA/TP63/HMGCS1/SERPINB5/LRP2/RETN/TLR3/RSPO3/ITGB8/DHCR24/SFRP2 | 48 |
| BP | GO:0061458 | reproductive system development | 48/1271 | 427/18723 | 0.000403 | 0.009121 | 0.007517 | ROBO2/DNAJB6/EAF2/RHOBTB3/PRDX4/ARRB2/KRT8/PLCD3/FZD5/LGR4/SOX15/PSAPL1/C3/MERTK/BMP5/STAR/CBL/STRA6/FGFR2/PTN/EOMES/GJB5/BCL2L1/PLEKHA1/BMP4/NEUROG1/WNT2B/ADA/HES1/PRDM1/GJA1/LHX3/GRHL2/PPARG/BMP6/CYP27B1/E2F8/RARA/TP63/HMGCS1/SERPINB5/LRP2/RETN/TLR3/RSPO3/ITGB8/DHCR24/SFRP2 | 48 |
| BP | GO:0009410 | response to xenobiotic stimulus | 48/1271 | 462/18723 | 0.002173 | 0.030453 | 0.025099 | SLC6A4/GSTA5/CYBA/GSTA4/CYP4F12/DPEP1/AADAC/BCHE/CYP2W1/CD38/CYP3A7/OXCT1/FBP1/AIM2/PTK2B/ACSM1/CXCR4/NPPC/AK4/MYC/IL1B/GSTA1/PDE4B/GAD1/RORC/STAR/CYP3A5/AHRR/CYP2C18/CYP2J2/PTN/NQO1/SST/CYBB/ADA/NCKAP1L/SLC22A12/MYO6/SOX10/RET/HMGCS1/GSTA2/GSTA3/TLR3/SFRP2/ANKRD1/CDH1/JUN | 48 |
| BP | GO:0030595 | leukocyte chemotaxis | 47/1271 | 230/18723 | 7.23E-12 | 7.88E-09 | 6.49E-09 | STK39/STAP1/SLAMF1/CXCL6/DPEP1/CXCL1/GPR18/F2RL1/CCL18/VAV1/SCG2/PIK3CD/CXCL3/FFAR2/PTK2B/ADAM8/AIF1/CXCR4/GPR183/TNFSF11/TREM1/PREX1/PDE4B/WNK1/CXCR1/CXCL2/PLA2G7/PTN/CCL13/CXCL13/C10orf99/PF4V1/PIK3CG/NCKAP1L/SAA1/IL6/FPR2/C3AR1/CCL5/CXADR/CD74/S100A14/FCER1G/LBP/CCL11/CXCL12/EDN2 | 47 |
| BP | GO:0030217 | T cell differentiation | 47/1271 | 257/18723 | 3.90E-10 | 1.77E-07 | 1.46E-07 | XBP1/RHOH/CTLA4/IRF4/BATF/CD3D/GPR18/PSMB11/CD86/TCIRG1/VAV1/CD80/PIK3CD/FZD5/ADAM8/ZMIZ1/CD3G/GPR183/PREX1/PLA2G2D/IL1B/NRARP/CR1/LILRB2/RORC/HLX/AIRE/IL7R/EOMES/BMP4/ITK/IL2RA/PCK1/TNFSF8/ADA/NCKAP1L/IL21/PRDM1/CTSL/CLPTM1/IL6/TOX/RARA/CD74/FCER1G/PTPRC/LEPR | 47 |
| BP | GO:1902105 | regulation of leukocyte differentiation | 47/1271 | 279/18723 | 6.26E-09 | 1.42E-06 | 1.17E-06 | XBP1/RHOH/CTLA4/TREM2/IRF4/LILRB1/IKZF3/HCLS1/TMEM176B/CD86/CTNNBIP1/LILRB3/CD80/ADAM8/PLA2G3/TMEM176A/ZMIZ1/TYROBP/RASSF2/TNFSF11/MYC/NRARP/CR1/LILRB2/OCSTAMP/HLX/IL17A/TRIB1/IL7R/LTF/BMP4/IL2RA/PCK1/ADA/NCKAP1L/PRDM1/GPR137/TLR9/CLPTM1/TOX/RARA/CD74/TLR3/PTPRC/TLR4/ID2/JUN | 47 |
| BP | GO:0050878 | regulation of body fluid levels | 47/1271 | 379/18723 | 4.55E-05 | 0.002016 | 0.001662 | NPR3/STK39/CYBA/XBP1/P2RY1/CYP4F12/F12/KRT1/P2RX1/TSPAN8/F2RL1/MPL/VAV1/FLG2/ENPP4/F5/PLEK/MERTK/PLCG2/KDF1/WNK1/SELP/STAT5A/TFPI2/NEUROG1/PF4V1/ADA/PIK3CG/PRLR/SAA1/CELSR2/GJA1/COMP/NPPB/IL6/RAP2B/TP63/ALOXE3/FCER1G/EMP2/KRT16/TLR4/FZD6/CD9/APLN/AQP3/F11R | 47 |
| BP | GO:0006909 | phagocytosis | 46/1271 | 308/18723 | 3.66E-07 | 4.43E-05 | 3.65E-05 | SPACA3/CYBA/RHOH/STAP1/SLAMF1/MYO1G/TREM2/ARL8B/IGLL1/TXNDC5/MST1R/F2RL1/FCN1/VAV1/BIN2/RAB20/NCF4/CD14/FCN2/RAB11FIP2/AIF1/TYROBP/TGM2/FCN3/C3/GULP1/IL1B/MERTK/PLCG2/IL2RG/PECAM1/FGR/NCKAP1L/NCF2/TLR2/FPR2/MARCO/CD93/RARA/FCER1G/LBP/TULP1/PTPRC/CEBPE/TLR4/LEPR | 46 |
| BP | GO:0002768 | immune response-regulating cell surface receptor signaling pathway | 46/1271 | 315/18723 | 7.02E-07 | 7.11E-05 | 5.86E-05 | CD19/BTLA/STAP1/KLHL6/CTLA4/LAT2/MYO1G/LILRB1/THEMIS2/SKAP1/IGLL1/CR2/CD226/FCN1/CD38/CD79A/BANK1/VAV1/PIK3CD/FFAR2/LPXN/TYROBP/LAX1/CD247/PLCG2/PAK3/CR1/PDE4B/LILRB2/WNK1/MNDA/MS4A1/LILRA2/FPR1/PRKCB/FGR/PLEKHA1/ITK/BTN3A1/ADA/NCKAP1L/MAP3K1/FPR2/C3AR1/FCER1G/PTPRC | 46 |
| BP | GO:0042113 | B cell activation | 46/1271 | 334/18723 | 3.62E-06 | 0.000274 | 0.000226 | XBP1/CD19/POU2AF1/CTLA4/LAT2/MZB1/IKZF3/THEMIS2/IGLL1/CD180/BATF/CR2/SAMSN1/ITGA4/CD86/TCIRG1/CD38/CD79A/BANK1/PIK3CD/PTK2B/FLT3/TYROBP/GPR183/LAX1/DNAJB9/PLCG2/CR1/MNDA/SH3KBP1/MS4A1/FCRL1/IL7R/PRKCB/ADA/NCKAP1L/ATM/IL21/GAPT/TLR9/IL6/CD74/PTPRC/TLR4/ID2/TNFRSF4 | 46 |
| BP | GO:0018108 | peptidyl-tyrosine phosphorylation | 46/1271 | 375/18723 | 7.11E-05 | 0.002693 | 0.00222 | CCK/STAP1/TREM2/IL24/EGF/SAMSN1/MST1R/EPHB3/IL31RA/HCLS1/GHR/BANK1/ARRB2/CD80/HES5/LILRA5/PTK2B/EFNA5/FLT3/CNTN1/FGFR4/MERTK/PLCG2/MATK/CBL/SPINK1/FGFR2/PECAM1/FGR/IL22RA2/ITK/AGT/PRLR/HES1/IL21/CRLF1/EFNA1/RET/IL6/BMP6/CCL5/RAP2B/CD74/WEE1/PTPRC/SFRP2 | 46 |
| BP | GO:0018212 | peptidyl-tyrosine modification | 46/1271 | 378/18723 | 8.62E-05 | 0.003011 | 0.002482 | CCK/STAP1/TREM2/IL24/EGF/SAMSN1/MST1R/EPHB3/IL31RA/HCLS1/GHR/BANK1/ARRB2/CD80/HES5/LILRA5/PTK2B/EFNA5/FLT3/CNTN1/FGFR4/MERTK/PLCG2/MATK/CBL/SPINK1/FGFR2/PECAM1/FGR/IL22RA2/ITK/AGT/PRLR/HES1/IL21/CRLF1/EFNA1/RET/IL6/BMP6/CCL5/RAP2B/CD74/WEE1/PTPRC/SFRP2 | 46 |
| BP | GO:0097529 | myeloid leukocyte migration | 45/1271 | 220/18723 | 1.95E-11 | 1.33E-08 | 1.10E-08 | RHOH/STAP1/SLAMF1/TREM2/TRIM55/CXCL6/DPEP1/CXCL1/CCL18/VAV1/SCG2/PIK3CD/CXCL3/PTK2B/ADAM8/AIF1/TNFSF11/TREM1/PREX1/CD200/PDE4B/CXCR1/IL17A/CXCL2/PLA2G7/PECAM1/CCL13/CXCL13/PF4V1/PIK3CG/NCKAP1L/SAA1/IL6/FPR2/C3AR1/CCL5/CXADR/CD74/S100A14/FCER1G/LBP/CCL11/CXCL12/CD9/EDN2 | 45 |
| BP | GO:1903037 | regulation of leukocyte cell-cell adhesion | 45/1271 | 336/18723 | 9.60E-06 | 0.000594 | 0.00049 | XBP1/RHOH/CTLA4/SLAMF1/CHST2/LILRB1/RASAL3/SKAP1/ITGA4/CD86/VAV1/CD80/ADAM8/AIF1/MIA3/ZMIZ1/LAX1/TNFSF11/PLA2G2D/IL1B/CLECL1/NRARP/CR1/LILRB2/WNK1/HLX/SELP/IL7R/BMP4/IL2RA/PCK1/ADA/NCKAP1L/HES1/IL21/IL6/CCL5/KLF4/RARA/CD74/GPNMB/IL20RB/CXCL12/PTPRC/VSIG4 | 45 |
| BP | GO:0042742 | defense response to bacterium | 45/1271 | 350/18723 | 2.71E-05 | 0.001407 | 0.00116 | CYBA/TREM2/CXCL6/IGLL1/F2RL1/NLRC4/JCHAIN/FCN2/IL17F/RNASE6/TREM1/PRB3/IL17A/SELP/PGC/IL7R/LTF/FGR/CXCL13/TNFSF8/C10orf99/GNLY/TLR2/TLR9/WFDC2/TLR6/IL6/FPR2/GBP6/CAMP/KLK7/DCD/LCE3B/S100A14/TLR3/GSDMC/FCER1G/LYZ/LBP/CEBPE/MPO/GBP2/TLR4/PI3/LCE3A | 45 |
| BP | GO:0032102 | negative regulation of response to external stimulus | 45/1271 | 420/18723 | 0.001616 | 0.024883 | 0.020509 | CCK/STAP1/TREM2/TARBP2/LILRB1/F12/KRT1/ROBO2/CST7/TSPAN8/GPR18/SHARPIN/LRFN5/ARRB2/SUSD4/AIF1/TNFAIP6/CD200/CR1/KREMEN1/TRIB1/LILRA2/LTF/SIGLEC10/FGR/IL22RA2/IL2RA/CXCL13/SEMA7A/CORO1B/ADA/SAA1/GJA1/NR1D2/NUCB2/PPARG/CLDN3/FPR2/KLF4/MMP12/IL20RB/PTPRC/VSIG4/CD9/SERPINB4 | 45 |
| BP | GO:0048568 | embryonic organ development | 45/1271 | 427/18723 | 0.002234 | 0.030984 | 0.025536 | TTC39C/TBX1/USH1G/DNAJB6/HOXA1/DLX5/VANGL2/MESP1/TULP3/KRT8/PLCD3/FZD5/SOX15/PPP1R13L/SLITRK6/ERCC3/BMP5/LRIG3/HLX/STRA6/FGFR2/EOMES/GJB5/BMP4/WNT16/NEUROG1/FOXH1/ADA/SOX11/HES1/FOXE1/PRDM1/EFNA1/GRHL2/MYO6/HOXB2/E2F8/RARA/DNAAF1/HOXD10/RSPO3/EN1/GJB6/FZD6/ID2 | 45 |
| BP | GO:0048732 | gland development | 45/1271 | 436/18723 | 0.003323 | 0.040852 | 0.03367 | XBP1/TBX1/TYR/EGF/EAF2/EPHB3/MESP1/TG/TGM2/UCP2/AK4/PSAPL1/TNFSF11/SOX2/HLX/KRT18/AIRE/STAT5A/STRA6/FGFR2/PTN/BMP4/PCK1/ADA/PRLR/HES1/FOXE1/DKK3/TGFBR3/LHX3/KRT76/SOX10/IL6/E2F8/IRF6/RARA/TP63/HMGCS1/DNAAF1/SERPINB5/SOSTDC1/CCL11/APLN/ID2/CDH1 | 45 |
| BP | GO:0051090 | regulation of DNA-binding transcription factor activity | 45/1271 | 440/18723 | 0.003938 | 0.046265 | 0.038131 | SMARCB1/CTH/IRAK2/NLRC4/HCLS1/CTNNBIP1/ARRB2/AIM2/ADAM8/EDA2R/TRAPPC9/HSPA1B/FANK1/TNFSF11/CD200/IL1B/PLCG2/MID2/TRIB1/ARHGEF5/LTF/PRKCB/EOMES/NEUROG1/FOXH1/AGT/HES1/TLR2/TLR9/PPARG/TLR6/IL6/DAP/JUP/KLF4/ANXA4/TLR3/RTKN2/CDKN2A/SFRP4/HSPA1A/TLR4/FZD6/ID2/TNFRSF4 | 45 |
| BP | GO:0016055 | Wnt signaling pathway | 45/1271 | 444/18723 | 0.004648 | 0.050652 | 0.041747 | BARX1/EGF/DLX5/BAMBI/VANGL2/NKD1/CTNNBIP1/MESP1/CDK14/SIAH2/FZD5/LGR4/SOX30/SOX2/PLCG2/NRARP/NDRG2/TMEM237/WNK1/KREMEN1/GRK5/FGFR2/TIAM1/WNT16/WNT2B/GSK3A/CELSR1/PPM1N/DKK3/TLR2/CELSR2/FZD10/MARK1/ATP6V1C2/SOX10/ZBTB33/JUP/KLF4/RSPO3/FERMT1/SFRP4/SOSTDC1/FZD6/SFRP2/DKK1 | 45 |
| BP | GO:0198738 | cell-cell signaling by wnt | 45/1271 | 446/18723 | 0.005042 | 0.052555 | 0.043315 | BARX1/EGF/DLX5/BAMBI/VANGL2/NKD1/CTNNBIP1/MESP1/CDK14/SIAH2/FZD5/LGR4/SOX30/SOX2/PLCG2/NRARP/NDRG2/TMEM237/WNK1/KREMEN1/GRK5/FGFR2/TIAM1/WNT16/WNT2B/GSK3A/CELSR1/PPM1N/DKK3/TLR2/CELSR2/FZD10/MARK1/ATP6V1C2/SOX10/ZBTB33/JUP/KLF4/RSPO3/FERMT1/SFRP4/SOSTDC1/FZD6/SFRP2/DKK1 | 45 |
| BP | GO:0031349 | positive regulation of defense response | 44/1271 | 278/18723 | 1.24E-07 | 1.73E-05 | 1.42E-05 | CCK/ZBP1/CYBA/STAP1/TREM2/TLR10/F2RL1/NLRC4/CD226/FCN1/VAV1/AIM2/LILRA5/FFAR2/ADAM8/PLA2G3/TYROBP/TGM2/MAPK13/C3/TNFSF11/IL1B/PLCG2/PAK3/ALOX5AP/MNDA/PGC/LILRA2/PLA2G7/PENK/AGT/PIK3CG/IL21/TLR2/GJA1/TLR9/IL6/FPR2/CCL5/KLK7/MMP12/TLR3/LBP/TLR4 | 44 |
| BP | GO:0002831 | regulation of response to biotic stimulus | 44/1271 | 327/18723 | 1.07E-05 | 0.000646 | 0.000532 | ZBP1/CYBA/TREM2/TARBP2/CXCL6/LILRB1/CD180/F2RL1/NLRC4/CD226/FCN1/VAV1/ARRB2/SUSD4/AIM2/IL17F/FFAR2/ADAM8/LY96/TYROBP/PLCG2/PAK3/CR1/MNDA/BIRC3/IL17A/TRIB1/PGC/LILRA2/LTF/FGR/APOBEC3G/IL21/PPARG/BMP6/FPR2/CCL5/KLK7/ZDHHC11/MMP12/LBP/VSIG4/TLR4/SERPINB4 | 44 |
| BP | GO:0051346 | negative regulation of hydrolase activity | 44/1271 | 379/18723 | 0.000348 | 0.00835 | 0.006882 | DPEP1/SERPINI1/CST7/DNAJB6/RARRES1/CRYAB/APOC1/ARRB2/SERPINA11/SIAH2/CPEB2/C3/HERPUD1/SMR3B/SERPINI2/CR1/WNK1/SERPINB10/BIRC3/SPINK1/SERPINB7/LTF/TFPI2/AGT/NCKAP1L/TIMP1/MMP9/FZD10/SERPINB11/WFDC2/SPINK7/FICD/KLF4/SERPINB5/SPINK9/A2ML1/DHCR24/RGS2/PI3/SFRP2/LEPR/F11R/SERPINB4/SERPINB13 | 44 |
| BP | GO:0006816 | calcium ion transport | 44/1271 | 422/18723 | 0.003036 | 0.038687 | 0.031885 | JSRP1/BSPRY/CYBA/CD19/PKDREJ/LILRB1/P2RX1/EGF/P2RX5/STAC2/ARRB2/LILRA5/WNK3/PTK2B/SLC35G1/CXCR4/HSPA2/PLCG2/PDE4B/LILRB2/MS4A1/HOMER2/ANXA6/SPINK1/BHLHA15/LILRA2/PRKCB/RYR1/MCOLN3/ORAI2/SLC24A5/CATSPER1/AGT/PIK3CG/GJA1/CACNG6/TLR9/SLC8A2/CYP27B1/CCL5/PSEN2/CXCL12/PTPRC/JPH1 | 44 |
| BP | GO:0042060 | wound healing | 44/1271 | 422/18723 | 0.003036 | 0.038687 | 0.031885 | XBP1/P2RY1/ARL8B/F12/KRT1/P2RX1/IL24/TSPAN8/F2RL1/SYTL4/MPL/VAV1/ENPP4/F5/MIA3/PLEK/CXCR4/MERTK/PLCG2/OCLN/SELP/ANXA6/FGFR2/TFPI2/DSP/CORO1B/PF4V1/AJUBA/PIK3CG/TIMP1/SAA1/GJA1/COMP/CLDN3/IL6/RAP2B/MMP12/PPL/FCER1G/FERMT1/TLR4/FZD6/CD9/F11R | 44 |
| BP | GO:0043588 | skin development | 43/1271 | 263/18723 | 6.68E-08 | 9.97E-06 | 8.22E-06 | SOX21/KRT1/KRT10/ACVR1B/SHARPIN/KRT2/FLG2/LGR4/LIPK/CLIC4/KDF1/KRT27/LTB/FGFR2/EXPH5/RYR1/DSP/WNT16/JAG1/FRAS1/FOXE1/GRHL2/KRT76/ZBED2/ASCL4/COMP/TCHH/CYP27B1/JUP/IRF6/TP63/LCE3B/PPL/ALOXE3/FERMT1/KRT16/SOSTDC1/LCE3D/DHCR24/FZD6/AQP3/LCE3E/LCE3A | 43 |
| BP | GO:0006959 | humoral immune response | 43/1271 | 317/18723 | 1.09E-05 | 0.000655 | 0.00054 | POU2AF1/TREM2/CXCL6/KRT1/IGLL1/CXCL1/CR2/CR1L/FCN1/JCHAIN/SUSD4/FCN2/IL17F/CXCL3/RNASE6/GPR183/FCN3/C3/TREM1/C1QB/IL1B/CR1/MS4A1/IL17A/CXCL2/AIRE/PGC/POU2F2/C1QA/LTF/CCL13/CXCL13/PF4V1/GNLY/ST6GAL1/WFDC2/IL6/CAMP/KLK7/LYZ/PTPRC/VSIG4/PI3 | 43 |
| BP | GO:0001503 | ossification | 43/1271 | 408/18723 | 0.002759 | 0.036767 | 0.030303 | GDPD2/DLX5/BAMBI/TCIRG1/CTNNBIP1/TUFT1/SRGN/MN1/PTK2B/LGR4/RASSF2/ADRB2/CHRDL2/NPPC/TNFSF11/PLXNB1/SOX2/BMP5/KREMEN1/KAZALD1/FGFR2/PTN/LTF/RYR1/FGR/BMP4/SEMA7A/PENK/JAG1/SOX11/MMP9/COMP/PPARG/RIPPLY2/IL6/BMP6/CYP27B1/MGP/TP63/GPNMB/MMP13/SFRP2/DKK1 | 43 |
| BP | GO:0071219 | cellular response to molecule of bacterial origin | 42/1271 | 221/18723 | 9.96E-10 | 3.39E-07 | 2.80E-07 | NUGGC/XBP1/STAP1/TREM2/CXCL6/LILRB1/IRAK2/CD180/CXCL1/IL24/CD86/CD80/CD14/CXCL3/FZD5/LY96/IL1B/PLCG2/PDE4B/LILRB2/STAR/TLR1/TRIB1/CXCL2/LILRA2/LTF/CXCL13/PF4V1/TLR2/TLR6/IL6/BMP6/CAMP/CCL5/RARA/TNIP3/IL1F10/LBP/CEBPE/TLR4/IL37/ANKRD1 | 42 |
| BP | GO:0071216 | cellular response to biotic stimulus | 42/1271 | 246/18723 | 2.68E-08 | 4.86E-06 | 4.01E-06 | NUGGC/XBP1/STAP1/TREM2/CXCL6/LILRB1/IRAK2/CD180/CXCL1/IL24/CD86/CD80/CD14/CXCL3/FZD5/LY96/IL1B/PLCG2/PDE4B/LILRB2/STAR/TLR1/TRIB1/CXCL2/LILRA2/LTF/CXCL13/PF4V1/TLR2/TLR6/IL6/BMP6/CAMP/CCL5/RARA/TNIP3/IL1F10/LBP/CEBPE/TLR4/IL37/ANKRD1 | 42 |
| BP | GO:0002429 | immune response-activating cell surface receptor signaling pathway | 42/1271 | 291/18723 | 2.87E-06 | 0.000231 | 0.00019 | CD19/STAP1/KLHL6/CTLA4/LAT2/MYO1G/THEMIS2/SKAP1/IGLL1/CR2/CD226/FCN1/CD38/CD79A/BANK1/VAV1/PIK3CD/FFAR2/LPXN/TYROBP/LAX1/CD247/PLCG2/PAK3/CR1/PDE4B/WNK1/MNDA/MS4A1/LILRA2/FPR1/PRKCB/FGR/PLEKHA1/ITK/BTN3A1/ADA/NCKAP1L/FPR2/C3AR1/FCER1G/PTPRC | 42 |
| BP | GO:0002757 | immune response-activating signal transduction | 42/1271 | 291/18723 | 2.87E-06 | 0.000231 | 0.00019 | CD19/STAP1/KLHL6/CTLA4/LAT2/MYO1G/THEMIS2/SKAP1/IGLL1/CR2/CD226/FCN1/CD38/CD79A/BANK1/VAV1/PIK3CD/FFAR2/LPXN/TYROBP/LAX1/CD247/PLCG2/PAK3/CR1/PDE4B/WNK1/MNDA/MS4A1/LILRA2/FPR1/PRKCB/FGR/PLEKHA1/ITK/BTN3A1/ADA/NCKAP1L/FPR2/C3AR1/FCER1G/PTPRC | 42 |
| BP | GO:0050863 | regulation of T cell activation | 42/1271 | 329/18723 | 5.85E-05 | 0.002362 | 0.001947 | XBP1/RHOH/CTLA4/SLAMF1/IRF4/LILRB1/RASAL3/CD86/VAV1/CD80/ADAM8/AIF1/ZMIZ1/LAX1/TNFSF11/PLA2G2D/IL1B/CLECL1/NRARP/CR1/LILRB2/HLX/IL7R/BMP4/IL2RA/PCK1/TNFSF8/ADA/NCKAP1L/HES1/IL21/PRDM1/CLPTM1/IL6/TOX/CCL5/RARA/CD74/GPNMB/IL20RB/PTPRC/VSIG4 | 42 |
| BP | GO:0051480 | regulation of cytosolic calcium ion concentration | 42/1271 | 353/18723 | 0.00028 | 0.007227 | 0.005956 | JSRP1/CYBA/CD19/P2RY1/NPY2R/P2RX1/GPR18/F2RL1/KCNA5/PROK2/P2RX5/P2RY10/CD38/CCR3/PTK2B/SLC35G1/GPR65/CXCR4/TGM2/PLCG2/CD52/CXCR1/MS4A1/ACKR4/FPR1/RYR1/MCOLN3/CXCL13/PTGIR/AGT/PIK3CG/GPR174/SAA1/GJA1/LPAR6/NMU/SLC8A2/FPR2/C3AR1/PTPRC/JPH1/EDN2 | 42 |
| BP | GO:0009306 | protein secretion | 42/1271 | 359/18723 | 0.000399 | 0.009121 | 0.007517 | TREM2/RAB3C/PDIA4/RAB3B/F2RL1/KCNA5/SYTL4/UNC13B/TCIRG1/CD38/OXCT1/SCG2/SEL1L/RAB11FIP2/ADAM8/ARL4D/EFNA5/MIA3/NNAT/PLEK/UCP2/CD200/IL1B/PCLO/ARFGAP3/EXPH5/TIAM1/FAM3B/SYBU/STXBP5/SAA1/TLR2/GJA1/COMP/NMU/IL6/BMP6/PARD6A/CCL5/VSNL1/TLR4/ANKRD1 | 42 |
| BP | GO:0035592 | establishment of protein localization to extracellular region | 42/1271 | 360/18723 | 0.000423 | 0.009494 | 0.007825 | TREM2/RAB3C/PDIA4/RAB3B/F2RL1/KCNA5/SYTL4/UNC13B/TCIRG1/CD38/OXCT1/SCG2/SEL1L/RAB11FIP2/ADAM8/ARL4D/EFNA5/MIA3/NNAT/PLEK/UCP2/CD200/IL1B/PCLO/ARFGAP3/EXPH5/TIAM1/FAM3B/SYBU/STXBP5/SAA1/TLR2/GJA1/COMP/NMU/IL6/BMP6/PARD6A/CCL5/VSNL1/TLR4/ANKRD1 | 42 |
| BP | GO:0071692 | protein localization to extracellular region | 42/1271 | 368/18723 | 0.000665 | 0.01313 | 0.010821 | TREM2/RAB3C/PDIA4/RAB3B/F2RL1/KCNA5/SYTL4/UNC13B/TCIRG1/CD38/OXCT1/SCG2/SEL1L/RAB11FIP2/ADAM8/ARL4D/EFNA5/MIA3/NNAT/PLEK/UCP2/CD200/IL1B/PCLO/ARFGAP3/EXPH5/TIAM1/FAM3B/SYBU/STXBP5/SAA1/TLR2/GJA1/COMP/NMU/IL6/BMP6/PARD6A/CCL5/VSNL1/TLR4/ANKRD1 | 42 |
| BP | GO:0002460 | adaptive immune response based on somatic recombination of immune receptors built from immunoglobulin superfamily domains | 41/1271 | 356/18723 | 0.000639 | 0.012767 | 0.010522 | CD19/KLHL6/SLAMF1/MYO1G/TREM2/IRF4/LILRB1/IGLL1/BATF/CR2/CD226/CR1L/TCIRG1/CD80/SUSD4/FZD5/PRF1/C3/C1QB/IL1B/CR1/RORC/HLX/AIRE/IL7R/C1QA/CXCL13/CTSH/JAG1/ADA/NCKAP1L/GAPT/CD1E/IL6/CD74/FCER1G/EMP2/IL20RB/PTPRC/TLR4/GZMM | 41 |
| BP | GO:0045860 | positive regulation of protein kinase activity | 41/1271 | 386/18723 | 0.002969 | 0.038262 | 0.031535 | STAP1/CHRNA3/EGF/MST1R/CD86/GHR/CCND2/LILRA5/FZD5/PTK2B/ADAM8/EFNA5/FLT3/RASSF2/ADRB2/TNFSF11/IL1B/WNK1/ERN1/MAPRE3/TIAM1/ARHGEF5/LTF/AJUBA/AGT/PIK3CG/PRLR/NCKAP1L/EFNA1/TLR9/FZD10/TLR6/VLDLR/SLC8A2/CCL5/DYNAP/TLR3/EMP2/PTPRC/TLR4/DKK1 | 41 |
| BP | GO:0006631 | fatty acid metabolic process | 41/1271 | 390/18723 | 0.003566 | 0.043058 | 0.035487 | ACOT2/XBP1/SLC27A6/ADH7/CYP4F12/TYRP1/PLA2G4D/CYP4F8/ABHD6/ELOVL4/ALDH3A2/APOC1/ACOXL/ACSM1/ACADSB/PLA2G3/TYSND1/ACSS2/C3/PDK1/IL1B/ELOVL6/GSTA1/ALOX5AP/CYP2C18/CYP2J2/TBXAS1/EIF6/PCK1/DEGS1/ABHD12/LTC4S/PTGR1/PPARG/PTGES/ELOVL2/CD74/ALOXE3/CROT/PTGDS/EDN2 | 41 |
| BP | GO:0002366 | leukocyte activation involved in immune response | 40/1271 | 275/18723 | 4.05E-06 | 0.000294 | 0.000243 | XBP1/CD19/POU2AF1/SLAMF1/LAT2/TREM2/IRF4/LILRB1/CD180/BATF/F2RL1/CD86/CD80/PIK3CD/PTK2B/PLA2G3/TYROBP/GPR183/PLCG2/CR1/RORC/HLX/CBL/LILRA2/EOMES/FGR/PCK1/ADA/PIK3CG/NCKAP1L/IL21/GAPT/IL6/RARA/CD74/FCER1G/LBP/PTPRC/TLR4/PTGDS | 40 |
| BP | GO:0002263 | cell activation involved in immune response | 40/1271 | 279/18723 | 5.79E-06 | 0.00039 | 0.000321 | XBP1/CD19/POU2AF1/SLAMF1/LAT2/TREM2/IRF4/LILRB1/CD180/BATF/F2RL1/CD86/CD80/PIK3CD/PTK2B/PLA2G3/TYROBP/GPR183/PLCG2/CR1/RORC/HLX/CBL/LILRA2/EOMES/FGR/PCK1/ADA/PIK3CG/NCKAP1L/IL21/GAPT/IL6/RARA/CD74/FCER1G/LBP/PTPRC/TLR4/PTGDS | 40 |
| BP | GO:0007162 | negative regulation of cell adhesion | 40/1271 | 303/18723 | 4.10E-05 | 0.001926 | 0.001587 | CTLA4/LILRB1/CD86/PDE3B/CD80/SIPA1/EFNA5/AKNA/LPXN/MIA3/PLXNC1/LAX1/PLXNB1/EPCAM/PLA2G2D/ARHGDIB/NRARP/CR1/LILRB2/WNK1/HLX/PODXL/BMP4/IL2RA/JAG1/ADAMDEC1/NCKAP1L/ARHGDIG/BMP6/KLF4/CD74/MMP12/GPNMB/CDKN2A/IL20RB/CXCL12/PTPRC/VSIG4/CD9/CDH1 | 40 |
| BP | GO:0007204 | positive regulation of cytosolic calcium ion concentration | 40/1271 | 319/18723 | 0.000128 | 0.004063 | 0.003349 | JSRP1/CYBA/CD19/P2RY1/NPY2R/P2RX1/GPR18/F2RL1/PROK2/P2RX5/P2RY10/CD38/CCR3/PTK2B/GPR65/CXCR4/TGM2/PLCG2/CD52/CXCR1/MS4A1/ACKR4/FPR1/RYR1/MCOLN3/CXCL13/PTGIR/AGT/PIK3CG/GPR174/SAA1/GJA1/LPAR6/NMU/SLC8A2/FPR2/C3AR1/PTPRC/JPH1/EDN2 | 40 |
| BP | GO:0043087 | regulation of GTPase activity | 40/1271 | 348/18723 | 0.000769 | 0.014648 | 0.012073 | ARHGEF26/RGS7/RASAL3/F2RL1/EPHB3/RASAL1/ARHGAP9/CCL18/VAV1/ARRB2/GARNL3/SIPA1/RAB11FIP2/PTK2B/GPR65/EFNA5/TBC1D9/PLXNC1/CPEB2/TGM2/PLXNB1/PREX1/WNK1/TBCK/TIAM1/ARHGEF5/CCL13/CXCL13/AJUBA/ARHGAP15/FZD10/ADRB1/RANGAP1/FICD/ARAP2/CCL5/RGS1/EVI5L/CCL11/F11R | 40 |
| BP | GO:0002449 | lymphocyte mediated immunity | 40/1271 | 350/18723 | 0.000859 | 0.015714 | 0.012951 | CD19/SLAMF1/MYO1G/TREM2/ARL8B/LILRB1/IGLL1/BATF/SLAMF7/CR2/CD226/CR1L/TCIRG1/VAV1/ARRB2/SUSD4/FZD5/PRF1/C3/C1QB/IL1B/CR1/GZMB/AIRE/IL7R/C1QA/CTSH/JAG1/NCKAP1L/IL21/GAPT/CD1E/IL6/CD74/FCER1G/EMP2/IL20RB/PTPRC/GZMM/SERPINB4 | 40 |
| BP | GO:0009615 | response to virus | 40/1271 | 367/18723 | 0.002092 | 0.029774 | 0.024539 | ZBP1/POU2AF1/TARBP2/LILRB1/ITGAX/ISG20/MST1R/F2RL1/AIM2/RNASE6/KCNJ8/CXCR4/PRF1/FCN3/CD207/PIM2/BIRC3/POU2F2/BCL2L1/FGR/PENK/DNAJC3/APOBEC3G/IL21/TLR2/TLR9/MX1/IL6/SLFN11/CCL5/IFI27/ZDHHC11/MMP12/TLR3/CCL11/ITGB8/CXCL12/PTPRC/IVNS1ABP/IFI44L | 40 |
| BP | GO:0050678 | regulation of epithelial cell proliferation | 40/1271 | 381/18723 | 0.004056 | 0.047027 | 0.038759 | CYBA/XBP1/IL26/TBX1/EGF/EAF2/DLX5/ITGA4/SCG2/PIK3CD/CCR3/HES5/MYC/SOX2/NRARP/BMP5/TNMD/KDF1/MYDGF/STAT5A/FGFR2/SLURP1/PTN/BMP4/MARVELD3/SOX11/SGPP2/HES1/TGFBR3/GJA1/PPARG/BMP6/FGFBP1/TP63/SERPINB5/MMP12/CCL11/CXCL12/SFRP2/APLN | 40 |
| BP | GO:0002699 | positive regulation of immune effector process | 39/1271 | 235/18723 | 1.81E-07 | 2.35E-05 | 1.93E-05 | XBP1/STAP1/SLAMF1/MZB1/TREM2/LILRB1/F2RL1/CD226/CD86/VAV1/CD80/SLC7A5/IL17F/FFAR2/FZD5/PLA2G3/TYROBP/DNAJB9/C3/IL1B/PLCG2/CR1/HLX/IL17A/PGC/FGR/PCK1/SEMA7A/IL21/TLR9/CD1E/IL6/RARA/KLK7/CD74/LBP/PTPRC/TLR4/TNFRSF4 | 39 |
| BP | GO:0070663 | regulation of leukocyte proliferation | 39/1271 | 245/18723 | 5.46E-07 | 5.84E-05 | 4.81E-05 | CTLA4/SLAMF1/MZB1/LILRB1/IKZF3/RASAL3/CD86/CD38/MPL/CD80/AIF1/TYROBP/GPR183/PLA2G2D/IL1B/CLECL1/CR1/LILRB2/OCSTAMP/MNDA/BMP4/IL2RA/TNFSF8/ADA/SOX11/NCKAP1L/ATM/HES1/IL21/TLR9/IL6/CCL5/CD74/GPNMB/IL20RB/PTPRC/VSIG4/TLR4/TNFRSF4 | 39 |
| BP | GO:0051251 | positive regulation of lymphocyte activation | 39/1271 | 362/18723 | 0.002895 | 0.037613 | 0.031 | XBP1/RHOH/SLAMF1/LILRB1/RASAL3/IGLL1/CD86/CD38/MPL/VAV1/CD80/ADAM8/AIF1/ZMIZ1/TYROBP/GPR183/TNFSF11/IL1B/CLECL1/CR1/LILRB2/SH3KBP1/HLX/IL7R/IL2RA/PCK1/ADA/NCKAP1L/HES1/IL21/TLR9/IL6/TOX/CCL5/RARA/CD74/PTPRC/TLR4/TNFRSF4 | 39 |
| BP | GO:0150063 | visual system development | 39/1271 | 375/18723 | 0.005272 | 0.053533 | 0.044121 | CRYBA4/THRB/B9D1/TBC1D32/NKD1/CRYAB/TCIRG1/BFSP2/TULP3/HES5/FZD5/PPP1R13L/C3/SLITRK6/NHS/SOX2/CLIC4/DIO3/MERTK/MEIS1/STRA6/ROM1/C1QA/PTN/PDE6A/BMP4/NR2E3/WNT16/JAG1/RPGRIP1/WNT2B/SOX11/PRDM1/GRHL2/RET/BMP6/KLF4/RARA/TULP1 | 39 |
| BP | GO:0071222 | cellular response to lipopolysaccharide | 38/1271 | 209/18723 | 2.18E-08 | 4.09E-06 | 3.37E-06 | NUGGC/XBP1/STAP1/CXCL6/LILRB1/IRAK2/CD180/CXCL1/IL24/CD86/CD80/CD14/CXCL3/LY96/IL1B/PLCG2/PDE4B/LILRB2/STAR/TRIB1/CXCL2/LILRA2/LTF/CXCL13/PF4V1/TLR2/IL6/BMP6/CAMP/CCL5/RARA/TNIP3/IL1F10/LBP/CEBPE/TLR4/IL37/ANKRD1 | 38 |
| BP | GO:0050670 | regulation of lymphocyte proliferation | 38/1271 | 225/18723 | 1.63E-07 | 2.17E-05 | 1.78E-05 | CTLA4/SLAMF1/MZB1/LILRB1/IKZF3/RASAL3/CD86/CD38/MPL/CD80/AIF1/TYROBP/GPR183/PLA2G2D/IL1B/CLECL1/CR1/LILRB2/MNDA/BMP4/IL2RA/TNFSF8/ADA/SOX11/NCKAP1L/ATM/HES1/IL21/TLR9/IL6/CCL5/CD74/GPNMB/IL20RB/PTPRC/VSIG4/TLR4/TNFRSF4 | 38 |
| BP | GO:0032944 | regulation of mononuclear cell proliferation | 38/1271 | 227/18723 | 2.06E-07 | 2.61E-05 | 2.15E-05 | CTLA4/SLAMF1/MZB1/LILRB1/IKZF3/RASAL3/CD86/CD38/MPL/CD80/AIF1/TYROBP/GPR183/PLA2G2D/IL1B/CLECL1/CR1/LILRB2/MNDA/BMP4/IL2RA/TNFSF8/ADA/SOX11/NCKAP1L/ATM/HES1/IL21/TLR9/IL6/CCL5/CD74/GPNMB/IL20RB/PTPRC/VSIG4/TLR4/TNFRSF4 | 38 |
| BP | GO:0001655 | urogenital system development | 38/1271 | 338/18723 | 0.00154 | 0.024117 | 0.019877 | COL4A4/ROBO2/EAF2/TBC1D32/EPHB3/VANGL2/CTNNBIP1/HES5/IRX2/LGR4/KCNJ8/PSAPL1/MYC/EPCAM/STRA6/FGFR2/SERPINB7/PECAM1/PODXL/BMP4/CTSH/TNS2/JAG1/FRAS1/WNT2B/ITGA8/SOX11/AGT/HES1/PRDM1/CRLF1/MMP9/RET/BMP6/RARA/TP63/SERPINB5/LRP2 | 38 |
| BP | GO:0045861 | negative regulation of proteolysis | 38/1271 | 351/18723 | 0.00299 | 0.038352 | 0.031609 | DPEP1/SERPINI1/CST7/DNAJB6/RARRES1/CRYAB/ARRB2/SERPINA11/SIAH2/C3/HERPUD1/SMR3B/SERPINI2/CR1/SERPINB10/CHAC1/BIRC3/SPINK1/SERPINB7/LTF/TFPI2/AGT/TIMP1/EFNA1/MMP9/SERPINB11/WFDC2/SPINK7/KLF4/SVIP/SERPINB5/SPINK9/A2ML1/DHCR24/PI3/SFRP2/SERPINB4/SERPINB13 | 38 |
| BP | GO:0051271 | negative regulation of cellular component movement | 38/1271 | 367/18723 | 0.006263 | 0.059874 | 0.049347 | STAP1/DPEP1/IL24/GPR18/ABHD6/IGFBP3/AIF1/MIA3/DNAJA4/CD200/CLIC4/ARHGAP4/ARHGDIB/BMP5/TP53INP1/TRIB1/SLURP1/PTN/MARVELD3/CXCL13/CORO1B/JAG1/ADA/TIMP1/TGFBR3/GJA1/PPARG/CLDN3/JUP/KLF4/RAP2B/CD74/HAS1/KRT16/CXCL12/CD9/SFRP2/CDH1 | 38 |
| BP | GO:0002573 | myeloid leukocyte differentiation | 37/1271 | 208/18723 | 6.00E-08 | 9.35E-06 | 7.71E-06 | TREM2/IRF4/LILRB1/BATF/F2RL1/IL31RA/HCLS1/TCIRG1/CTNNBIP1/LILRB3/PIK3CD/PLA2G3/TYROBP/RASSF2/GPR183/TNFSF11/MYC/PIR/OCSTAMP/IL17A/TRIB1/LTF/BMP4/TLR2/GPR137/MMP9/PPARG/MT1G/RARA/CD74/TLR3/ITGB8/CEBPE/TLR4/ID2/BATF2/JUN | 37 |
| BP | GO:0022409 | positive regulation of cell-cell adhesion | 37/1271 | 284/18723 | 0.000103 | 0.003502 | 0.002886 | XBP1/RHOH/SLAMF1/CHST2/LILRB1/RASAL3/SKAP1/ITGA4/CD86/VAV1/CD80/ADAM8/AIF1/ZMIZ1/TNFSF11/SOX2/IL1B/CLECL1/CR1/LILRB2/HLX/SELP/IL7R/PODXL/IL2RA/PCK1/CXCL13/ADA/NCKAP1L/HES1/IL21/IL6/CCL5/RARA/CD74/PTPRC/F11R | 37 |
| BP | GO:0051047 | positive regulation of secretion | 37/1271 | 310/18723 | 0.000591 | 0.012249 | 0.010095 | SLC6A4/CCK/CYBA/XBP1/TREM2/P2RY1/NPY2R/F2RL1/SYTL4/UNC13B/CD38/OXCT1/ADAM8/PLA2G3/NNAT/TNFSF11/IL1B/EXPH5/FGR/SYBU/SOX11/AGT/STXBP5/TLR2/GJA1/PTGES/NMU/NPPB/BMP6/PARD6A/VSNL1/RETN/RAB27B/CXCL12/TLR4/APLN/ANKRD1 | 37 |
| BP | GO:0060249 | anatomical structure homeostasis | 37/1271 | 314/18723 | 0.000752 | 0.014474 | 0.011929 | STK39/USH1G/P2RY1/KRT1/P2RX1/VSIG1/TCIRG1/CD38/JCHAIN/WNK3/PTK2B/ADAM8/ADRB2/TNFSF11/OCLN/SLC2A1/IL17A/HOMER2/RDH12/PECAM1/LTF/MC4R/CTSH/GPR137/GJA1/TLR9/IL20RA/CLDN3/IL6/SLC8A2/CXADR/LYZ/IL20RB/TULP1/GJB6/TLR4/F11R | 37 |
| BP | GO:0071496 | cellular response to external stimulus | 37/1271 | 320/18723 | 0.001066 | 0.018507 | 0.015253 | CYBA/XBP1/NUAK2/ITGA4/SIPA1/MN1/AIF1/WIPI1/UCP2/DAPL1/IL1B/MAP1LC3B/SLC2A1/CBL/RRAGD/BHLHA15/PTN/PCK1/PENK/WNT2B/AGT/GJA1/MAP3K1/CPEB4/BMP6/DAP/CYP27B1/TLR3/PTPRC/TLR4/SFRP2/AQP3/MMP7/ANKRD1/F11R/SLC38A2/JUN | 37 |
| BP | GO:0003002 | regionalization | 37/1271 | 331/18723 | 0.001948 | 0.02832 | 0.023341 | TBX1/BARX1/ROBO2/TBC1D32/NKD1/CTNNBIP1/MESP1/TULP3/HES5/FZD5/IRX2/C3/NRARP/BTG2/FGFR2/C1QA/PGAP1/BMP4/NEUROG1/FOXH1/DZIP1L/WNT2B/HES1/CELSR2/LHX3/HOXB2/RIPPLY2/TP63/DNAAF1/HOXD10/LRP2/EN1/SOSTDC1/BHLHE41/SFRP2/DKK1/ETS2 | 37 |
| BP | GO:0010951 | negative regulation of endopeptidase activity | 35/1271 | 252/18723 | 4.27E-05 | 0.00199 | 0.001641 | DPEP1/SERPINI1/CST7/DNAJB6/RARRES1/CRYAB/ARRB2/SERPINA11/SIAH2/C3/HERPUD1/SMR3B/SERPINI2/CR1/SERPINB10/BIRC3/SPINK1/SERPINB7/LTF/TFPI2/AGT/TIMP1/MMP9/SERPINB11/WFDC2/SPINK7/KLF4/SERPINB5/SPINK9/A2ML1/DHCR24/PI3/SFRP2/SERPINB4/SERPINB13 | 35 |
| BP | GO:0010466 | negative regulation of peptidase activity | 35/1271 | 262/18723 | 9.53E-05 | 0.003308 | 0.002726 | DPEP1/SERPINI1/CST7/DNAJB6/RARRES1/CRYAB/ARRB2/SERPINA11/SIAH2/C3/HERPUD1/SMR3B/SERPINI2/CR1/SERPINB10/BIRC3/SPINK1/SERPINB7/LTF/TFPI2/AGT/TIMP1/MMP9/SERPINB11/WFDC2/SPINK7/KLF4/SERPINB5/SPINK9/A2ML1/DHCR24/PI3/SFRP2/SERPINB4/SERPINB13 | 35 |
| BP | GO:0003018 | vascular process in circulatory system | 35/1271 | 263/18723 | 0.000103 | 0.003502 | 0.002886 | SLC6A4/NPR3/P2RY1/P2RX1/F2RL1/KCNA5/CD38/CTNNBIP1/SLC7A5/ADRB2/KCNJ8/OCLN/SLC2A1/SLC7A8/TBXAS1/UTS2B/SLC15A2/ACE2/AGT/GJA1/SLC7A2/ADRB1/COMP/SLCO2B1/DOCK4/NPPB/BMP6/SLC8A2/LRP2/SLC5A5/RGS2/APLN/LEPR/EDN2/SLC38A2 | 35 |
| BP | GO:0050730 | regulation of peptidyl-tyrosine phosphorylation | 35/1271 | 264/18723 | 0.000111 | 0.003625 | 0.002987 | CCK/STAP1/TREM2/IL24/EGF/SAMSN1/IL31RA/HCLS1/GHR/BANK1/ARRB2/CD80/HES5/LILRA5/PTK2B/EFNA5/FLT3/CNTN1/PLCG2/CBL/SPINK1/PECAM1/IL22RA2/AGT/HES1/IL21/CRLF1/EFNA1/IL6/BMP6/CCL5/RAP2B/CD74/PTPRC/SFRP2 | 35 |
| BP | GO:0044403 | biological process involved in symbiotic interaction | 35/1271 | 290/18723 | 0.00067 | 0.013191 | 0.010872 | SMARCB1/SLAMF1/ARL8B/CXCL6/CR2/F2RL1/FCN1/ULK2/CD86/CD80/HSPA1B/CXCR4/FCN3/TREM1/TFAP4/CR1/MID2/CBL/LTF/BCL2L1/CLEC4M/SELPLG/ACE2/CTSL/CAMP/CCL5/IFI27/CXADR/DCD/SIGLEC1/CD74/HSPA1A/F11R/TNFRSF4/JUN | 35 |
| BP | GO:0010720 | positive regulation of cell development | 35/1271 | 298/18723 | 0.001091 | 0.018877 | 0.015559 | DCT/NEFL/MAPT/ROBO2/MPL/ELL3/TYROBP/PLXNC1/CXCR4/TGM2/TNFSF11/PLXNB1/PREX1/IL1B/PAK3/STAR/TRIB1/TIAM1/PTN/CUX2/BMP4/SEMA7A/SOX11/HES1/TLR2/SOX10/RET/IL6/CRABP2/BNC1/LRP2/CXCL12/RASSF10/ID2/F11R | 35 |
| BP | GO:0043062 | extracellular structure organization | 35/1271 | 302/18723 | 0.001377 | 0.022142 | 0.018249 | LAMB4/COL4A4/TLL2/VIT/MMP3/DNAJB6/COL4A6/MMP1/CTSS/PRDX4/COL4A2/MMP27/MMP10/ADAM8/MIA/FGFR4/TEX14/KAZALD1/C6orf15/COL15A1/AGT/COL11A2/MMP9/COMP/IL6/KLK7/SERPINB5/MMP12/HAS1/MMP13/FERMT1/COL9A1/SFRP2/MMP7/ADAMTS4 | 35 |
| BP | GO:0070371 | ERK1 and ERK2 cascade | 35/1271 | 330/18723 | 0.005876 | 0.056892 | 0.04689 | IL26/SLAMF1/TREM2/P2RY1/EGF/F2RL1/CCL18/ARRB2/PTK2B/RPS6KA6/FGF19/GPR183/TNFSF11/MYC/IL1B/FGFR4/NDRG2/DUSP4/FGFR2/TIAM1/BMP4/CCL13/SEMA7A/CTSH/AGT/FPR2/MARCO/YWHAZ/CCL5/KLF4/CD74/GPNMB/CCL11/PTPRC/TLR4 | 35 |
| BP | GO:0019216 | regulation of lipid metabolic process | 35/1271 | 331/18723 | 0.006155 | 0.059073 | 0.048687 | CD19/TREM2/AADAC/PDE3B/ABHD6/APOC1/PTK2B/FLT3/PLA2G3/TYSND1/FGF19/PSAPL1/C3/PDK1/IL1B/FGFR4/PLCG2/BMP5/RORC/STAR/EIF6/FGR/PCK1/AGT/PIK3CG/LGALS12/DKK3/NR1D2/PPARG/BMP6/FPR2/CYP27B1/CD74/EEF1A2/ID2 | 35 |
| BP | GO:0071674 | mononuclear cell migration | 34/1271 | 196/18723 | 3.78E-07 | 4.48E-05 | 3.69E-05 | STK39/SLAMF1/MYO1G/ITGA4/CCL18/PIK3CD/PTK2B/ADAM8/AIF1/MIA3/CXCR4/GPR183/TNFSF11/CD200/BMP5/WNK1/CXCR1/AIRE/PLA2G7/PECAM1/CCL13/CXCL13/C10orf99/PIK3CG/SAA1/RET/IL6/FPR2/C3AR1/CCL5/S100A14/CCL11/CXCL12/F11R | 34 |
| BP | GO:0009913 | epidermal cell differentiation | 34/1271 | 202/18723 | 7.80E-07 | 7.59E-05 | 6.25E-05 | KRT10/SHARPIN/KRT2/HES5/OVOL3/SLITRK6/ERCC3/LIPK/CLIC4/KDF1/EXPH5/MCOLN3/BMP4/DSP/WNT16/JAG1/HES1/GRHL2/ZBED2/MYO6/ESRP1/TCHH/CYP27B1/IRF6/KLF4/TP63/LCE3B/PPL/SFRP4/KRT16/LCE3D/AQP3/LCE3E/LCE3A | 34 |
| BP | GO:0002685 | regulation of leukocyte migration | 34/1271 | 210/18723 | 1.94E-06 | 0.000168 | 0.000138 | STK39/RHOH/STAP1/SLAMF1/TREM2/CHST2/GPR18/F2RL1/ITGA4/PTK2B/ADAM8/AIF1/MIA3/CD200/BMP5/WNK1/SELP/AIRE/PLA2G7/PTN/CXCL13/C10orf99/ADA/NCKAP1L/IL6/FPR2/C3AR1/CCL5/CD74/S100A14/LBP/CXCL12/CD9/EDN2 | 34 |
| BP | GO:0001894 | tissue homeostasis | 34/1271 | 268/18723 | 0.00032 | 0.008068 | 0.006649 | STK39/USH1G/KRT1/VSIG1/TCIRG1/CD38/JCHAIN/WNK3/PTK2B/ADAM8/ADRB2/TNFSF11/OCLN/SLC2A1/IL17A/HOMER2/RDH12/PECAM1/LTF/MC4R/CTSH/GPR137/GJA1/TLR9/IL20RA/CLDN3/IL6/CXADR/LYZ/IL20RB/TULP1/GJB6/TLR4/F11R | 34 |
| BP | GO:0098742 | cell-cell adhesion via plasma-membrane adhesion molecules | 34/1271 | 273/18723 | 0.000451 | 0.009835 | 0.008106 | IGSF9/ROBO2/EMB/LRFN5/SELL/PCDH17/EPCAM/CLDN22/WNK1/SELP/PECAM1/PTPRF/PCDH7/AJUBA/CELSR1/CLDN17/PCDH20/CELSR2/DSC3/TENM2/RET/CLDN3/DSC2/KLF4/PCDH19/CXADR/DSG3/CLDN10/MPZL2/FAT2/CDH18/CNTN6/CDH1/CDH7 | 34 |
| BP | GO:1903532 | positive regulation of secretion by cell | 34/1271 | 282/18723 | 0.000808 | 0.01524 | 0.012561 | SLC6A4/CCK/TREM2/P2RY1/NPY2R/F2RL1/SYTL4/UNC13B/CD38/OXCT1/ADAM8/PLA2G3/NNAT/TNFSF11/IL1B/EXPH5/FGR/SYBU/SOX11/AGT/STXBP5/TLR2/GJA1/PTGES/NMU/BMP6/PARD6A/VSNL1/RETN/RAB27B/CXCL12/TLR4/APLN/ANKRD1 | 34 |
| BP | GO:0030198 | extracellular matrix organization | 34/1271 | 301/18723 | 0.002447 | 0.033025 | 0.027219 | LAMB4/COL4A4/TLL2/VIT/MMP3/DNAJB6/COL4A6/MMP1/CTSS/PRDX4/COL4A2/MMP27/MMP10/ADAM8/MIA/FGFR4/KAZALD1/C6orf15/COL15A1/AGT/COL11A2/MMP9/COMP/IL6/KLK7/SERPINB5/MMP12/HAS1/MMP13/FERMT1/COL9A1/SFRP2/MMP7/ADAMTS4 | 34 |
| BP | GO:0045229 | external encapsulating structure organization | 34/1271 | 304/18723 | 0.002874 | 0.037613 | 0.031 | LAMB4/COL4A4/TLL2/VIT/MMP3/DNAJB6/COL4A6/MMP1/CTSS/PRDX4/COL4A2/MMP27/MMP10/ADAM8/MIA/FGFR4/KAZALD1/C6orf15/COL15A1/AGT/COL11A2/MMP9/COMP/IL6/KLK7/SERPINB5/MMP12/HAS1/MMP13/FERMT1/COL9A1/SFRP2/MMP7/ADAMTS4 | 34 |
| BP | GO:0046394 | carboxylic acid biosynthetic process | 34/1271 | 314/18723 | 0.004788 | 0.051073 | 0.042094 | XBP1/CTH/OSBPL1A/SHMT1/OSBPL6/ELOVL4/CYP39A1/APOC1/ACSM1/PLA2G3/ACSS2/FGF19/IL1B/FGFR4/GLUD2/ELOVL6/GAD1/ALOX5AP/STAR/TBXAS1/EIF6/GATM/DEGS1/SDSL/PHGDH/KYNU/LTC4S/PTGES/ELOVL2/GGT7/CD74/ALOXE3/PTGDS/EDN2 | 34 |
| BP | GO:0016053 | organic acid biosynthetic process | 34/1271 | 316/18723 | 0.005278 | 0.053533 | 0.044121 | XBP1/CTH/OSBPL1A/SHMT1/OSBPL6/ELOVL4/CYP39A1/APOC1/ACSM1/PLA2G3/ACSS2/FGF19/IL1B/FGFR4/GLUD2/ELOVL6/GAD1/ALOX5AP/STAR/TBXAS1/EIF6/GATM/DEGS1/SDSL/PHGDH/KYNU/LTC4S/PTGES/ELOVL2/GGT7/CD74/ALOXE3/PTGDS/EDN2 | 34 |
| BP | GO:0097530 | granulocyte migration | 33/1271 | 148/18723 | 9.25E-10 | 3.36E-07 | 2.77E-07 | RHOH/SLAMF1/CXCL6/DPEP1/CXCL1/CCL18/VAV1/SCG2/PIK3CD/CXCL3/ADAM8/TREM1/PREX1/PDE4B/CXCR1/IL17A/CXCL2/PECAM1/CCL13/CXCL13/PF4V1/PIK3CG/NCKAP1L/SAA1/C3AR1/CCL5/CXADR/CD74/S100A14/FCER1G/LBP/CCL11/EDN2 | 33 |
| BP | GO:0001906 | cell killing | 33/1271 | 188/18723 | 4.22E-07 | 4.89E-05 | 4.03E-05 | STAP1/ARL8B/CXCL6/LILRB1/SLAMF7/F2RL1/CD226/CR1L/VAV1/ARRB2/TYROBP/PRF1/C3/TREM1/GZMB/IL7R/LTF/BCL2L1/CCL13/CTSH/NCKAP1L/IL21/GNLY/CD1E/CAMP/DCD/LCE3B/LYZ/EMP2/PTPRC/GZMM/LCE3A/SERPINB4 | 33 |
| BP | GO:0045216 | cell-cell junction organization | 33/1271 | 200/18723 | 1.78E-06 | 0.000156 | 0.000129 | PKP3/F2RL1/FZD5/POF1B/IL1B/OCLN/CLDN22/PARD3/IL17A/PECAM1/MARVELD3/DSP/ACE2/PKP1/AGT/CLDN17/MPP7/GJA1/GRHL2/CLDN3/BMP6/JUP/MARVELD2/PARD6A/CXADR/CLDN10/GJB6/PERP/CD9/CDH18/F11R/CDH1/CDH7 | 33 |
| BP | GO:0050866 | negative regulation of cell activation | 33/1271 | 210/18723 | 5.30E-06 | 0.00037 | 0.000305 | CTLA4/TREM2/LILRB1/CST7/SAMSN1/IL31RA/CD86/LRFN5/BANK1/CD80/TYROBP/LAX1/CD200/PLA2G2D/MERTK/NRARP/CR1/LILRB2/MNDA/HLX/FGR/BMP4/IL2RA/SOX11/NCKAP1L/ATM/CD74/GPNMB/IL20RB/PTPRC/VSIG4/CD9/ID2 | 33 |
| BP | GO:1903039 | positive regulation of leukocyte cell-cell adhesion | 33/1271 | 239/18723 | 7.85E-05 | 0.002832 | 0.002334 | XBP1/RHOH/SLAMF1/CHST2/LILRB1/RASAL3/SKAP1/ITGA4/CD86/VAV1/CD80/ADAM8/AIF1/ZMIZ1/TNFSF11/IL1B/CLECL1/CR1/LILRB2/HLX/SELP/IL7R/IL2RA/PCK1/ADA/NCKAP1L/HES1/IL21/IL6/CCL5/RARA/CD74/PTPRC | 33 |
| BP | GO:0001822 | kidney development | 33/1271 | 293/18723 | 0.002941 | 0.037992 | 0.031313 | COL4A4/ROBO2/TBC1D32/VANGL2/CTNNBIP1/HES5/IRX2/LGR4/KCNJ8/MYC/EPCAM/STRA6/FGFR2/SERPINB7/PECAM1/PODXL/BMP4/CTSH/TNS2/JAG1/FRAS1/WNT2B/ITGA8/SOX11/AGT/HES1/PRDM1/CRLF1/MMP9/RET/BMP6/RARA/LRP2 | 33 |
| BP | GO:0051056 | regulation of small GTPase mediated signal transduction | 33/1271 | 302/18723 | 0.0047 | 0.050652 | 0.041747 | RASAL3/GPR18/F2RL1/TAGAP/RASAL1/ARHGAP9/P2RY10/VAV1/FBP1/GARNL3/SIPA1/SSX2IP/GPR65/TGM2/PREX1/PSD4/ARHGAP4/ARHGDIB/CBL/TIAM1/ARHGEF5/RACGAP1/DENND4C/DENND3/PLEKHG3/ARHGAP15/PIK3CG/MYO9B/EPS8L1/GPR174/LPAR6/ARHGAP32/F11R | 33 |
| BP | GO:0072001 | renal system development | 33/1271 | 302/18723 | 0.0047 | 0.050652 | 0.041747 | COL4A4/ROBO2/TBC1D32/VANGL2/CTNNBIP1/HES5/IRX2/LGR4/KCNJ8/MYC/EPCAM/STRA6/FGFR2/SERPINB7/PECAM1/PODXL/BMP4/CTSH/TNS2/JAG1/FRAS1/WNT2B/ITGA8/SOX11/AGT/HES1/PRDM1/CRLF1/MMP9/RET/BMP6/RARA/LRP2 | 33 |
| BP | GO:0051222 | positive regulation of protein transport | 33/1271 | 303/18723 | 0.004941 | 0.051832 | 0.042719 | CHP2/UBL4B/TREM2/EDEM1/F2RL1/SYTL4/HCLS1/UNC13B/CD38/OXCT1/OAZ3/HSPA1L/ZDHHC2/FZD5/ADAM8/NNAT/IL1B/GZMB/EXPH5/ARHGEF5/SYBU/GSK3A/TLR2/GJA1/NMU/BMP6/JUP/PARD6A/VPS28/VSNL1/TLR4/ANKRD1/CDH1 | 33 |
| BP | GO:0032635 | interleukin-6 production | 32/1271 | 165/18723 | 5.86E-08 | 9.35E-06 | 7.71E-06 | CYBA/XBP1/POU2AF1/SLAMF1/TREM2/F2RL1/BANK1/ARRB2/IL17F/LILRA5/AIF1/TYROBP/MAPK13/CD200/IL1B/PLCG2/LILRB2/TLR1/IL17A/LILRA2/POU2F2/NCKAP1L/TLR2/TLR9/TLR6/IL6/CD74/TLR3/IL17D/LBP/TLR4/IL37 | 32 |
| BP | GO:0032675 | regulation of interleukin-6 production | 32/1271 | 165/18723 | 5.86E-08 | 9.35E-06 | 7.71E-06 | CYBA/XBP1/POU2AF1/SLAMF1/TREM2/F2RL1/BANK1/ARRB2/IL17F/LILRA5/AIF1/TYROBP/MAPK13/CD200/IL1B/PLCG2/LILRB2/TLR1/IL17A/LILRA2/POU2F2/NCKAP1L/TLR2/TLR9/TLR6/IL6/CD74/TLR3/IL17D/LBP/TLR4/IL37 | 32 |
| BP | GO:0045637 | regulation of myeloid cell differentiation | 32/1271 | 210/18723 | 1.40E-05 | 0.00081 | 0.000668 | TREM2/LILRB1/ACVR1B/HCLS1/KLF13/MPL/CTNNBIP1/LILRB3/PTK2B/PITHD1/PLA2G3/TYROBP/RASSF2/HSPA1B/TNFSF11/MYC/OCSTAMP/MEIS1/IL17A/TRIB1/EIF6/LTF/JAG1/NCKAP1L/GPR137/RARA/CD74/TLR3/HSPA1A/TLR4/ID2/JUN | 32 |
| BP | GO:0009612 | response to mechanical stimulus | 32/1271 | 216/18723 | 2.49E-05 | 0.00132 | 0.001088 | CYBA/P2RY1/PKDREJ/KCNA5/PTK2B/CXCR4/SLITRK6/IL1B/SLC2A1/BTG2/STRA6/PTN/NEUROG1/AGT/PHF24/ABHD12/GJA1/MAP3K1/BMP6/JUP/RETN/PPL/TLR3/CXCL12/MPO/TLR4/NRXN1/MMP7/ANKRD1/F11R/SLC38A2/JUN | 32 |
| BP | GO:0002221 | pattern recognition receptor signaling pathway | 31/1271 | 172/18723 | 5.17E-07 | 5.63E-05 | 4.64E-05 | CYBA/TREM2/IRF4/IRAK2/TLR10/F2RL1/FCN1/CTSS/ARRB2/CD14/RAB11FIP2/FFAR2/LY96/LGR4/HSPA1B/PLCG2/TLR1/BIRC3/LILRA2/LTF/CLEC4E/SLC15A2/TLR2/TLR9/TLR6/TNIP3/S100A14/TLR3/HSPA1A/LBP/TLR4 | 31 |
| BP | GO:0071706 | tumor necrosis factor superfamily cytokine production | 31/1271 | 186/18723 | 2.92E-06 | 0.000231 | 0.00019 | CYBA/SLAMF1/TREM2/LILRB1/GPR18/CD86/ARRB2/CD14/IL17F/LILRA5/FZD5/ADAM8/LY96/TYROBP/PLCG2/UBE2J1/TLR1/IL17A/LILRA2/LTF/CYBB/TLR2/TLR9/IL6/RARA/TLR3/GPNMB/LBP/PTPRC/TLR4/IL37 | 31 |
| BP | GO:1903555 | regulation of tumor necrosis factor superfamily cytokine production | 31/1271 | 186/18723 | 2.92E-06 | 0.000231 | 0.00019 | CYBA/SLAMF1/TREM2/LILRB1/GPR18/CD86/ARRB2/CD14/IL17F/LILRA5/FZD5/ADAM8/LY96/TYROBP/PLCG2/UBE2J1/TLR1/IL17A/LILRA2/LTF/CYBB/TLR2/TLR9/IL6/RARA/TLR3/GPNMB/LBP/PTPRC/TLR4/IL37 | 31 |
| BP | GO:0002695 | negative regulation of leukocyte activation | 31/1271 | 187/18723 | 3.28E-06 | 0.000252 | 0.000207 | CTLA4/LILRB1/CST7/SAMSN1/IL31RA/CD86/LRFN5/BANK1/CD80/TYROBP/LAX1/CD200/PLA2G2D/MERTK/NRARP/CR1/LILRB2/MNDA/HLX/FGR/BMP4/IL2RA/SOX11/NCKAP1L/ATM/CD74/GPNMB/IL20RB/PTPRC/VSIG4/ID2 | 31 |
| BP | GO:0051091 | positive regulation of DNA-binding transcription factor activity | 31/1271 | 260/18723 | 0.001605 | 0.024883 | 0.020509 | SMARCB1/CTH/IRAK2/NLRC4/HCLS1/AIM2/ADAM8/EDA2R/TRAPPC9/HSPA1B/FANK1/TNFSF11/CD200/IL1B/PLCG2/MID2/ARHGEF5/LTF/PRKCB/NEUROG1/AGT/TLR2/TLR9/PPARG/TLR6/IL6/JUP/TLR3/RTKN2/HSPA1A/TLR4 | 31 |
| BP | GO:0021700 | developmental maturation | 31/1271 | 280/18723 | 0.004975 | 0.052051 | 0.0429 | NEFL/UNC13B/BFSP2/HES5/ZDHHC2/FZD5/PTK2B/ROPN1/PLA2G3/ROPN1B/BCL11A/NPPC/C3/PLXNB1/SPINK1/BHLHA15/C1QA/LTF/RYR1/SYBU/HES1/IL21/PPARG/SOX10/RET/BNC1/YWHAZ/LHX6/FERMT1/NRXN1/PAEP | 31 |
| BP | GO:0002064 | epithelial cell development | 30/1271 | 220/18723 | 0.000203 | 0.005737 | 0.004728 | ARHGEF26/F2RL1/VSIG1/BFSP2/KRT2/CXCR4/SLITRK6/POF1B/IL1B/CLIC4/BMP5/TNMD/KDF1/EXPH5/PECAM1/PODXL/BMP4/COL15A1/JAG1/GSK3A/KLF5/PRDM1/GRHL2/CLDN3/BMP6/RAB25/MARVELD2/RARA/TP63/F11R | 30 |
| BP | GO:0050920 | regulation of chemotaxis | 30/1271 | 223/18723 | 0.000258 | 0.006925 | 0.005707 | STK39/STAP1/SLAMF1/TREM2/ROBO2/GPR18/F2RL1/SCG2/PTK2B/AIF1/NOVA2/CXCR4/GPR183/WNK1/PLA2G7/TIAM1/PTN/CXCL13/SEMA7A/CORO1B/NCKAP1L/IL6/FPR2/C3AR1/CCL5/CD74/S100A14/LBP/CXCL12/EDN2 | 30 |
| BP | GO:0002703 | regulation of leukocyte mediated immunity | 30/1271 | 226/18723 | 0.000326 | 0.008079 | 0.006659 | STAP1/SLAMF1/TREM2/CXCL6/LILRB1/CR2/F2RL1/CD226/CR1L/VAV1/ARRB2/SUSD4/FZD5/PLA2G3/TYROBP/C3/IL1B/PLCG2/CR1/IL7R/FGR/NCKAP1L/IL21/CD1E/IL6/TLR3/IL20RB/PTPRC/TLR4/SERPINB4 | 30 |
| BP | GO:0045926 | negative regulation of growth | 30/1271 | 249/18723 | 0.001608 | 0.024883 | 0.020509 | SLC6A4/MAP2/TLL2/ACVR1B/EAF2/FSTL4/NKD1/ULK2/CRYAB/FBP1/SIPA1/ADRB2/HSPA1B/BCL11A/ARHGAP4/MEIS1/BMP4/SEMA7A/GSK3A/AGT/GJA1/ADRB1/MT1X/NPPB/CYP27B1/MT1G/CDKN2A/HSPA1A/RGS2/SFRP2 | 30 |
| BP | GO:0045165 | cell fate commitment | 30/1271 | 258/18723 | 0.002771 | 0.036822 | 0.030348 | ARX/TBX1/IRF4/BATF/MESP1/HES5/SOX2/FGFR2/EOMES/BMP4/WNT16/NEUROG1/JAG1/WNT2B/HES1/PRDM1/CTSL/LHX3/PPARG/ESRP1/IL6/TOX/KLF4/RARA/HOXD10/SOSTDC1/SFRP2/DKK1/ETS2/ID2 | 30 |
| BP | GO:1990266 | neutrophil migration | 29/1271 | 122/18723 | 1.99E-09 | 6.03E-07 | 4.97E-07 | RHOH/CXCL6/DPEP1/CXCL1/CCL18/VAV1/PIK3CD/CXCL3/ADAM8/TREM1/PREX1/PDE4B/CXCR1/CXCL2/PECAM1/CCL13/CXCL13/PF4V1/PIK3CG/NCKAP1L/SAA1/C3AR1/CCL5/CXADR/CD74/FCER1G/LBP/CCL11/EDN2 | 29 |
| BP | GO:0071621 | granulocyte chemotaxis | 29/1271 | 125/18723 | 3.63E-09 | 9.43E-07 | 7.77E-07 | SLAMF1/CXCL6/DPEP1/CXCL1/CCL18/VAV1/SCG2/PIK3CD/CXCL3/TREM1/PREX1/PDE4B/CXCR1/CXCL2/CCL13/CXCL13/PF4V1/PIK3CG/NCKAP1L/SAA1/C3AR1/CCL5/CXADR/CD74/S100A14/FCER1G/LBP/CCL11/EDN2 | 29 |
| BP | GO:1902107 | positive regulation of leukocyte differentiation | 29/1271 | 157/18723 | 7.04E-07 | 7.11E-05 | 5.86E-05 | XBP1/RHOH/TREM2/HCLS1/CD86/CTNNBIP1/CD80/ADAM8/PLA2G3/ZMIZ1/TYROBP/TNFSF11/CR1/LILRB2/OCSTAMP/HLX/IL17A/TRIB1/IL7R/IL2RA/PCK1/ADA/NCKAP1L/TOX/RARA/CD74/PTPRC/ID2/JUN | 29 |
| BP | GO:1903708 | positive regulation of hemopoiesis | 29/1271 | 157/18723 | 7.04E-07 | 7.11E-05 | 5.86E-05 | XBP1/RHOH/TREM2/HCLS1/CD86/CTNNBIP1/CD80/ADAM8/PLA2G3/ZMIZ1/TYROBP/TNFSF11/CR1/LILRB2/OCSTAMP/HLX/IL17A/TRIB1/IL7R/IL2RA/PCK1/ADA/NCKAP1L/TOX/RARA/CD74/PTPRC/ID2/JUN | 29 |
| BP | GO:0050731 | positive regulation of peptidyl-tyrosine phosphorylation | 29/1271 | 193/18723 | 4.52E-05 | 0.002016 | 0.001662 | CCK/STAP1/TREM2/IL24/EGF/IL31RA/HCLS1/GHR/BANK1/ARRB2/CD80/HES5/LILRA5/PTK2B/EFNA5/FLT3/CNTN1/PLCG2/PECAM1/AGT/HES1/IL21/CRLF1/EFNA1/IL6/BMP6/CCL5/CD74/PTPRC | 29 |
| BP | GO:0050870 | positive regulation of T cell activation | 29/1271 | 216/18723 | 0.000335 | 0.008197 | 0.006755 | XBP1/RHOH/SLAMF1/LILRB1/RASAL3/CD86/VAV1/CD80/ADAM8/AIF1/ZMIZ1/TNFSF11/IL1B/CLECL1/CR1/LILRB2/HLX/IL7R/IL2RA/PCK1/ADA/NCKAP1L/HES1/IL21/IL6/CCL5/RARA/CD74/PTPRC | 29 |
| BP | GO:0045088 | regulation of innate immune response | 29/1271 | 218/18723 | 0.000392 | 0.009121 | 0.007517 | ZBP1/TREM2/LILRB1/NLRC4/CD226/FCN1/VAV1/ARRB2/SUSD4/AIM2/FFAR2/ADAM8/TYROBP/PLCG2/PAK3/CR1/MNDA/BIRC3/LILRA2/FGR/IL21/PPARG/FPR2/CCL5/MMP12/LBP/VSIG4/TLR4/SERPINB4 | 29 |
| BP | GO:0051924 | regulation of calcium ion transport | 29/1271 | 255/18723 | 0.004437 | 0.050369 | 0.041514 | JSRP1/CYBA/CD19/LILRB1/P2RX1/EGF/P2RX5/STAC2/ARRB2/LILRA5/WNK3/PTK2B/CXCR4/HSPA2/PLCG2/PDE4B/LILRB2/HOMER2/SPINK1/LILRA2/AGT/PIK3CG/GJA1/CACNG6/TLR9/CCL5/PSEN2/CXCL12/JPH1 | 29 |
| BP | GO:0034976 | response to endoplasmic reticulum stress | 29/1271 | 256/18723 | 0.004689 | 0.050652 | 0.041747 | XBP1/CLGN/CTH/EDEM1/ERLEC1/PDIA4/NHLRC1/SEL1L/RHBDD1/DNAJB9/HERPUD1/UGGT1/DNAJC10/FBXO44/UBE2J1/CHAC1/RHBDD2/ERN1/SDF2L1/TMEM117/BHLHA15/BCL2L1/DNAJC3/MAN1A1/FICD/SVIP/HSPA1A/GORASP2/JUN | 29 |
| BP | GO:0090596 | sensory organ morphogenesis | 29/1271 | 256/18723 | 0.004689 | 0.050652 | 0.041747 | TTC39C/TBX1/USH1G/THRB/HOXA1/DLX5/NKD1/FZD5/SLITRK6/DIO3/MEIS1/LRIG3/STRA6/FGFR2/ROM1/PTN/BMP4/NR2E3/WNT16/NEUROG1/JAG1/RPGRIP1/WNT2B/SOX11/PRDM1/MYO6/TULP1/GJB6/FZD6 | 29 |
| BP | GO:0002833 | positive regulation of response to biotic stimulus | 28/1271 | 168/18723 | 8.67E-06 | 0.00055 | 0.000453 | ZBP1/CYBA/CD180/F2RL1/NLRC4/CD226/FCN1/VAV1/AIM2/IL17F/FFAR2/ADAM8/LY96/TYROBP/PLCG2/PAK3/MNDA/IL17A/PGC/LILRA2/IL21/BMP6/FPR2/CCL5/KLK7/MMP12/LBP/TLR4 | 28 |
| BP | GO:0032640 | tumor necrosis factor production | 28/1271 | 181/18723 | 3.57E-05 | 0.001736 | 0.001431 | CYBA/SLAMF1/TREM2/LILRB1/GPR18/ARRB2/CD14/LILRA5/FZD5/LY96/TYROBP/PLCG2/UBE2J1/TLR1/IL17A/LILRA2/LTF/CYBB/TLR2/TLR9/IL6/RARA/TLR3/GPNMB/LBP/PTPRC/TLR4/IL37 | 28 |
| BP | GO:0032680 | regulation of tumor necrosis factor production | 28/1271 | 181/18723 | 3.57E-05 | 0.001736 | 0.001431 | CYBA/SLAMF1/TREM2/LILRB1/GPR18/ARRB2/CD14/LILRA5/FZD5/LY96/TYROBP/PLCG2/UBE2J1/TLR1/IL17A/LILRA2/LTF/CYBB/TLR2/TLR9/IL6/RARA/TLR3/GPNMB/LBP/PTPRC/TLR4/IL37 | 28 |
| BP | GO:0002285 | lymphocyte activation involved in immune response | 28/1271 | 194/18723 | 0.000124 | 0.003974 | 0.003275 | XBP1/CD19/POU2AF1/IRF4/LILRB1/CD180/BATF/F2RL1/CD86/CD80/PTK2B/GPR183/PLCG2/CR1/RORC/HLX/EOMES/PCK1/ADA/NCKAP1L/IL21/GAPT/IL6/RARA/CD74/FCER1G/PTPRC/TLR4 | 28 |
| BP | GO:0022408 | negative regulation of cell-cell adhesion | 28/1271 | 196/18723 | 0.000148 | 0.004535 | 0.003738 | CTLA4/LILRB1/CD86/CD80/AKNA/MIA3/LAX1/EPCAM/PLA2G2D/NRARP/CR1/LILRB2/WNK1/HLX/PODXL/BMP4/IL2RA/JAG1/NCKAP1L/BMP6/KLF4/CD74/GPNMB/IL20RB/CXCL12/VSIG4/CD9/CDH1 | 28 |
| BP | GO:0050851 | antigen receptor-mediated signaling pathway | 28/1271 | 240/18723 | 0.003586 | 0.043058 | 0.035487 | CD19/STAP1/KLHL6/CTLA4/LAT2/THEMIS2/SKAP1/IGLL1/CD226/CD38/CD79A/BANK1/PIK3CD/LPXN/LAX1/CD247/PLCG2/PDE4B/WNK1/MNDA/MS4A1/PRKCB/PLEKHA1/ITK/BTN3A1/ADA/NCKAP1L/PTPRC | 28 |
| BP | GO:0002700 | regulation of production of molecular mediator of immune response | 27/1271 | 164/18723 | 1.57E-05 | 0.000882 | 0.000727 | XBP1/SLAMF1/MZB1/LILRB1/F2RL1/CD226/CD86/SLC7A5/IL17F/FFAR2/FZD5/DNAJB9/IL1B/PLCG2/CR1/IL17A/PGC/SEMA7A/IL21/TLR9/IL6/KLK7/CD74/TLR3/PTPRC/TLR4/TNFRSF4 | 27 |
| BP | GO:0045619 | regulation of lymphocyte differentiation | 27/1271 | 174/18723 | 4.62E-05 | 0.002032 | 0.001675 | XBP1/RHOH/CTLA4/IRF4/IKZF3/CD86/CD80/ADAM8/ZMIZ1/NRARP/CR1/LILRB2/HLX/IL7R/BMP4/IL2RA/PCK1/ADA/NCKAP1L/PRDM1/TLR9/CLPTM1/TOX/RARA/CD74/PTPRC/ID2 | 27 |
| BP | GO:0071466 | cellular response to xenobiotic stimulus | 27/1271 | 177/18723 | 6.26E-05 | 0.002472 | 0.002037 | GSTA5/GSTA4/CYP4F12/DPEP1/AADAC/CYP2W1/CYP3A7/FBP1/AIM2/ACSM1/CXCR4/MYC/IL1B/GSTA1/PDE4B/RORC/STAR/CYP3A5/AHRR/CYP2C18/CYP2J2/NQO1/SOX10/GSTA2/GSTA3/TLR3/ANKRD1 | 27 |
| BP | GO:0042098 | T cell proliferation | 27/1271 | 199/18723 | 0.000447 | 0.009834 | 0.008105 | CTLA4/SLAMF1/LILRB1/RASAL3/CD86/CD80/AIF1/PLA2G2D/IL1B/CLECL1/CR1/LILRB2/BMP4/IL2RA/TNFSF8/BTN3A1/PIK3CG/NCKAP1L/HES1/IL21/IL6/CCL5/GPNMB/IL20RB/PTPRC/VSIG4/TNFRSF4 | 27 |
| BP | GO:0042445 | hormone metabolic process | 27/1271 | 218/18723 | 0.001801 | 0.026313 | 0.021687 | BCO1/ADH7/PCSK2/CYP2W1/GHR/CYP3A7/TG/DIO3/BMP5/STAR/CYP3A5/CYP2C18/RDH12/PLEKHA1/ACE2/FOXE1/ADH1A/PNLIP/DKK3/CTSL/AKR1B10/BMP6/CRABP2/CYP27B1/SLC16A10/SLC5A5/UGT2B17 | 27 |
| BP | GO:2000116 | regulation of cysteine-type endopeptidase activity | 27/1271 | 235/18723 | 0.005147 | 0.053212 | 0.043856 | CCK/DPEP1/MAPT/P2RX1/CST7/DNAJB6/NLRC4/CRYAB/ARRB2/AIM2/SIAH2/CASP10/MYC/SOX2/HERPUD1/TFAP4/BIRC3/LTF/CTSH/MMP9/PPARG/DAP/KLF4/TP63/DHCR24/PERP/SFRP2 | 27 |
| BP | GO:1901617 | organic hydroxy compound biosynthetic process | 27/1271 | 237/18723 | 0.005762 | 0.056802 | 0.046815 | DCT/PMVK/P2RY1/CYB5R3/TYR/OSBPL1A/TYRP1/OSBPL6/CYP39A1/PTK2B/PLEK/FGF19/PPIP5K1/FGFR4/PLCG2/BMP5/STAR/PCK1/SLC24A5/GPR37/DKK3/PLTP/BMP6/CYP27B1/HMGCS1/DHCR24/SC5D | 27 |
| BP | GO:0030593 | neutrophil chemotaxis | 26/1271 | 103/18723 | 3.46E-09 | 9.42E-07 | 7.77E-07 | CXCL6/DPEP1/CXCL1/CCL18/VAV1/PIK3CD/CXCL3/TREM1/PREX1/PDE4B/CXCR1/CXCL2/CCL13/CXCL13/PF4V1/PIK3CG/NCKAP1L/SAA1/C3AR1/CCL5/CXADR/CD74/FCER1G/LBP/CCL11/EDN2 | 26 |
| BP | GO:0042116 | macrophage activation | 26/1271 | 106/18723 | 6.66E-09 | 1.45E-06 | 1.20E-06 | SPACA3/STAP1/TREM2/MAPT/CST7/IL31RA/LRFN5/AIF1/PLA2G3/TYROBP/CD200/PLCG2/TLR1/C1QA/TLR2/TLR6/IL6/FPR2/CD93/CD74/TLR3/LBP/PTPRC/VSIG4/TLR4/EDN2 | 26 |
| BP | GO:0007043 | cell-cell junction assembly | 26/1271 | 146/18723 | 5.26E-06 | 0.00037 | 0.000305 | PKP3/FZD5/POF1B/IL1B/OCLN/CLDN22/PARD3/IL17A/PECAM1/MARVELD3/ACE2/PKP1/AGT/CLDN17/MPP7/GJA1/GRHL2/CLDN3/JUP/MARVELD2/CLDN10/GJB6/CD9/CDH18/F11R/CDH7 | 26 |
| BP | GO:0051250 | negative regulation of lymphocyte activation | 26/1271 | 157/18723 | 2.01E-05 | 0.001108 | 0.000913 | CTLA4/LILRB1/SAMSN1/CD86/BANK1/CD80/TYROBP/LAX1/PLA2G2D/MERTK/NRARP/CR1/LILRB2/MNDA/HLX/FGR/BMP4/IL2RA/SOX11/NCKAP1L/ATM/CD74/GPNMB/IL20RB/VSIG4/ID2 | 26 |
| BP | GO:0043405 | regulation of MAP kinase activity | 26/1271 | 177/18723 | 0.00016 | 0.004832 | 0.003983 | EGF/MST1R/GHR/FZD5/PTK2B/ADAM8/FLT3/LAX1/TNFSF11/IL1B/ERN1/TRIB1/TIAM1/ARHGEF5/BMP4/AJUBA/AGT/PIK3CG/TLR9/FZD10/PPARG/TLR6/TLR4/RGS2/SFRP2/DKK1 | 26 |
| BP | GO:0050864 | regulation of B cell activation | 26/1271 | 198/18723 | 0.000928 | 0.016639 | 0.013714 | XBP1/CD19/CTLA4/MZB1/IKZF3/THEMIS2/IGLL1/SAMSN1/CD38/BANK1/TYROBP/GPR183/CR1/MNDA/SH3KBP1/ADA/NCKAP1L/ATM/IL21/TLR9/IL6/CD74/PTPRC/TLR4/ID2/TNFRSF4 | 26 |
| BP | GO:0060348 | bone development | 26/1271 | 205/18723 | 0.001544 | 0.024121 | 0.01988 | ZNF385A/LILRB1/DLX5/GHR/TULP3/TYROBP/TGM2/NPPC/TNFSF11/PLXNB1/MEIS1/ANXA6/FGFR2/LTF/RYR1/BMP4/NEUROG1/GJA1/COMP/RIPPLY2/BMP6/RARA/MMP13/SFRP4/PTPRC/SFRP2 | 26 |
| BP | GO:0007596 | blood coagulation | 26/1271 | 217/18723 | 0.003423 | 0.04183 | 0.034476 | P2RY1/F12/KRT1/P2RX1/TSPAN8/F2RL1/MPL/VAV1/ENPP4/F5/PLEK/MERTK/PLCG2/SELP/TFPI2/PF4V1/PIK3CG/SAA1/COMP/IL6/RAP2B/FCER1G/TLR4/FZD6/CD9/F11R | 26 |
| BP | GO:0007599 | hemostasis | 26/1271 | 222/18723 | 0.004643 | 0.050652 | 0.041747 | P2RY1/F12/KRT1/P2RX1/TSPAN8/F2RL1/MPL/VAV1/ENPP4/F5/PLEK/MERTK/PLCG2/SELP/TFPI2/PF4V1/PIK3CG/SAA1/COMP/IL6/RAP2B/FCER1G/TLR4/FZD6/CD9/F11R | 26 |
| BP | GO:0050817 | coagulation | 26/1271 | 222/18723 | 0.004643 | 0.050652 | 0.041747 | P2RY1/F12/KRT1/P2RX1/TSPAN8/F2RL1/MPL/VAV1/ENPP4/F5/PLEK/MERTK/PLCG2/SELP/TFPI2/PF4V1/PIK3CG/SAA1/COMP/IL6/RAP2B/FCER1G/TLR4/FZD6/CD9/F11R | 26 |
| BP | GO:0050769 | positive regulation of neurogenesis | 26/1271 | 225/18723 | 0.005537 | 0.055995 | 0.046151 | DCT/NEFL/MAPT/ROBO2/ELL3/PLXNC1/CXCR4/TGM2/PLXNB1/IL1B/PAK3/STAR/TIAM1/PTN/CUX2/SEMA7A/SOX11/HES1/TLR2/SOX10/IL6/CRABP2/LRP2/CXCL12/RASSF10/ID2 | 26 |
| BP | GO:0046777 | protein autophosphorylation | 26/1271 | 227/18723 | 0.006209 | 0.05948 | 0.049022 | STK39/ACVR1B/STK33/EPHB3/ULK2/WNK3/PTK2B/STK17B/FLT3/RASSF2/PIM2/FGFR4/MAP4K1/WNK1/GRK5/ERN1/FGFR2/FGR/ITK/ATM/HTATIP2/SLK/RAP2B/GPNMB/AURKB/PTPRC | 26 |
| BP | GO:0032755 | positive regulation of interleukin-6 production | 25/1271 | 93/18723 | 1.68E-09 | 5.38E-07 | 4.43E-07 | CYBA/XBP1/POU2AF1/F2RL1/IL17F/LILRA5/AIF1/TYROBP/MAPK13/IL1B/PLCG2/LILRB2/TLR1/IL17A/LILRA2/POU2F2/TLR2/TLR9/TLR6/IL6/CD74/TLR3/IL17D/LBP/TLR4 | 25 |
| BP | GO:0002702 | positive regulation of production of molecular mediator of immune response | 25/1271 | 117/18723 | 2.37E-07 | 2.93E-05 | 2.42E-05 | XBP1/SLAMF1/MZB1/LILRB1/F2RL1/CD226/CD86/SLC7A5/IL17F/FFAR2/FZD5/DNAJB9/IL1B/PLCG2/IL17A/PGC/SEMA7A/IL21/TLR9/IL6/KLK7/CD74/PTPRC/TLR4/TNFRSF4 | 25 |
| BP | GO:0002224 | toll-like receptor signaling pathway | 25/1271 | 121/18723 | 4.65E-07 | 5.28E-05 | 4.35E-05 | CYBA/TREM2/IRF4/IRAK2/TLR10/F2RL1/CTSS/ARRB2/CD14/RAB11FIP2/LY96/LGR4/PLCG2/TLR1/BIRC3/LILRA2/LTF/TLR2/TLR9/TLR6/TNIP3/S100A14/TLR3/LBP/TLR4 | 25 |
| BP | GO:0050729 | positive regulation of inflammatory response | 25/1271 | 142/18723 | 9.76E-06 | 0.000598 | 0.000493 | ZBP1/STAP1/TREM2/TLR10/LILRA5/FFAR2/ADAM8/PLA2G3/TGM2/MAPK13/C3/TNFSF11/IL1B/PLCG2/ALOX5AP/PLA2G7/AGT/PIK3CG/IL21/TLR2/TLR9/IL6/TLR3/LBP/TLR4 | 25 |
| BP | GO:0046631 | alpha-beta T cell activation | 25/1271 | 156/18723 | 5.07E-05 | 0.002159 | 0.001779 | IRF4/LILRB1/RASAL3/BATF/GPR18/PSMB11/CD86/TCIRG1/CD80/GPR183/PLA2G2D/RORC/HLX/EOMES/ITK/TNFSF8/ADA/NCKAP1L/IL21/PRDM1/CTSL/IL6/TOX/RARA/PTPRC | 25 |
| BP | GO:0048660 | regulation of smooth muscle cell proliferation | 25/1271 | 180/18723 | 0.000504 | 0.010836 | 0.008931 | NPR3/CYBA/CTNNBIP1/IGFBP3/HES5/AIF1/TGM2/NPPC/NDRG2/ERN1/TRIB1/FGFR2/BMP4/PTGIR/AGT/GJA1/MMP9/PPARG/IL6/CCL5/KLF4/TLR4/APLN/ID2/JUN | 25 |
| BP | GO:0048659 | smooth muscle cell proliferation | 25/1271 | 184/18723 | 0.0007 | 0.013729 | 0.011316 | NPR3/CYBA/CTNNBIP1/IGFBP3/HES5/AIF1/TGM2/NPPC/NDRG2/ERN1/TRIB1/FGFR2/BMP4/PTGIR/AGT/GJA1/MMP9/PPARG/IL6/CCL5/KLF4/TLR4/APLN/ID2/JUN | 25 |
| BP | GO:0050679 | positive regulation of epithelial cell proliferation | 25/1271 | 207/18723 | 0.003648 | 0.04342 | 0.035786 | CYBA/XBP1/TBX1/EGF/DLX5/ITGA4/SCG2/PIK3CD/CCR3/MYC/NRARP/BMP5/MYDGF/STAT5A/FGFR2/PTN/BMP4/SOX11/BMP6/FGFBP1/TP63/MMP12/CCL11/CXCL12/APLN | 25 |
| BP | GO:0072330 | monocarboxylic acid biosynthetic process | 25/1271 | 214/18723 | 0.005609 | 0.056105 | 0.046241 | XBP1/OSBPL1A/OSBPL6/ELOVL4/CYP39A1/APOC1/ACSM1/PLA2G3/ACSS2/FGF19/IL1B/FGFR4/ELOVL6/STAR/TBXAS1/EIF6/GATM/DEGS1/LTC4S/PTGES/ELOVL2/CD74/ALOXE3/PTGDS/EDN2 | 25 |
| BP | GO:0002687 | positive regulation of leukocyte migration | 24/1271 | 135/18723 | 1.23E-05 | 0.00073 | 0.000602 | STK39/SLAMF1/TREM2/F2RL1/ITGA4/PTK2B/ADAM8/AIF1/MIA3/WNK1/SELP/PLA2G7/PTN/CXCL13/NCKAP1L/IL6/FPR2/C3AR1/CCL5/CD74/S100A14/LBP/CXCL12/EDN2 | 24 |
| BP | GO:0045580 | regulation of T cell differentiation | 24/1271 | 146/18723 | 4.68E-05 | 0.002041 | 0.001682 | XBP1/RHOH/CTLA4/IRF4/CD86/CD80/ADAM8/ZMIZ1/NRARP/CR1/LILRB2/HLX/IL7R/BMP4/IL2RA/PCK1/ADA/NCKAP1L/PRDM1/CLPTM1/TOX/RARA/CD74/PTPRC | 24 |
| BP | GO:0070665 | positive regulation of leukocyte proliferation | 24/1271 | 150/18723 | 7.30E-05 | 0.002743 | 0.00226 | SLAMF1/RASAL3/CD86/CD38/MPL/CD80/AIF1/GPR183/IL1B/CLECL1/LILRB2/OCSTAMP/IL2RA/ADA/NCKAP1L/HES1/IL21/TLR9/IL6/CCL5/CD74/PTPRC/TLR4/TNFRSF4 | 24 |
| BP | GO:0035966 | response to topologically incorrect protein | 24/1271 | 159/18723 | 0.000185 | 0.005321 | 0.004386 | XBP1/CTH/F12/EDEM1/ERLEC1/HSPA1L/HSPA13/HSPA1B/RHBDD1/DNAJB9/HSPA2/HERPUD1/UGGT1/CHAC1/RHBDD2/ERN1/SDF2L1/BHLHA15/DNAJC3/COMP/DNAJB1/FICD/HSPA1A/HSPA4L | 24 |
| BP | GO:0042129 | regulation of T cell proliferation | 24/1271 | 171/18723 | 0.000554 | 0.011624 | 0.00958 | CTLA4/SLAMF1/LILRB1/RASAL3/CD86/CD80/AIF1/PLA2G2D/IL1B/CLECL1/CR1/LILRB2/BMP4/IL2RA/TNFSF8/NCKAP1L/HES1/IL21/IL6/CCL5/GPNMB/IL20RB/PTPRC/VSIG4 | 24 |
| BP | GO:0001659 | temperature homeostasis | 24/1271 | 174/18723 | 0.000714 | 0.013953 | 0.0115 | NPR3/UCP1/IRF4/ABHD6/LGR4/ADRB2/NOVA2/CXCR4/UCP2/TNFSF11/IL1B/ELOVL6/GATM/TSHR/PRLR/G0S2/GJA1/ADRB1/PTGES/NMU/TLR4/APLN/LEPR/EDN2 | 24 |
| BP | GO:0071902 | positive regulation of protein serine/threonine kinase activity | 24/1271 | 200/18723 | 0.004701 | 0.050652 | 0.041747 | EGF/MST1R/GHR/CCND2/FZD5/PTK2B/ADAM8/FLT3/ADRB2/TNFSF11/IL1B/ERN1/MAPRE3/TIAM1/ARHGEF5/LTF/AJUBA/PIK3CG/TLR9/FZD10/TLR6/SLC8A2/TLR4/DKK1 | 24 |
| BP | GO:0009952 | anterior/posterior pattern specification | 24/1271 | 201/18723 | 0.005005 | 0.052263 | 0.043074 | TBX1/BARX1/NKD1/CTNNBIP1/MESP1/TULP3/HES5/FZD5/NRARP/BTG2/PGAP1/BMP4/NEUROG1/FOXH1/WNT2B/HES1/CELSR2/HOXB2/RIPPLY2/HOXD10/EN1/BHLHE41/SFRP2/ETS2 | 24 |
| BP | GO:0051701 | biological process involved in interaction with host | 24/1271 | 203/18723 | 0.005662 | 0.056317 | 0.046416 | SLAMF1/ARL8B/CR2/FCN1/ULK2/CD86/CD80/HSPA1B/CXCR4/FCN3/CR1/MID2/CBL/BCL2L1/CLEC4M/SELPLG/ACE2/CTSL/CXADR/SIGLEC1/CD74/HSPA1A/F11R/TNFRSF4 | 24 |
| BP | GO:0042100 | B cell proliferation | 23/1271 | 99/18723 | 1.44E-07 | 1.96E-05 | 1.61E-05 | CD19/CTLA4/MZB1/IKZF3/CD180/CR2/CD38/CD79A/TYROBP/GPR183/MNDA/MS4A1/IL7R/ADA/NCKAP1L/ATM/IL21/GAPT/TLR9/CD74/PTPRC/TLR4/TNFRSF4 | 23 |
| BP | GO:0072676 | lymphocyte migration | 23/1271 | 117/18723 | 3.26E-06 | 0.000252 | 0.000207 | STK39/MYO1G/ITGA4/CCL18/PIK3CD/PTK2B/ADAM8/AIF1/MIA3/GPR183/CD200/WNK1/AIRE/CCL13/CXCL13/C10orf99/PIK3CG/SAA1/RET/CCL5/CCL11/CXCL12/F11R | 23 |
| BP | GO:0050671 | positive regulation of lymphocyte proliferation | 23/1271 | 137/18723 | 4.76E-05 | 0.002061 | 0.001699 | SLAMF1/RASAL3/CD86/CD38/MPL/CD80/AIF1/GPR183/IL1B/CLECL1/LILRB2/IL2RA/ADA/NCKAP1L/HES1/IL21/TLR9/IL6/CCL5/CD74/PTPRC/TLR4/TNFRSF4 | 23 |
| BP | GO:0032946 | positive regulation of mononuclear cell proliferation | 23/1271 | 138/18723 | 5.36E-05 | 0.002251 | 0.001855 | SLAMF1/RASAL3/CD86/CD38/MPL/CD80/AIF1/GPR183/IL1B/CLECL1/LILRB2/IL2RA/ADA/NCKAP1L/HES1/IL21/TLR9/IL6/CCL5/CD74/PTPRC/TLR4/TNFRSF4 | 23 |
| BP | GO:0030216 | keratinocyte differentiation | 23/1271 | 139/18723 | 6.01E-05 | 0.00241 | 0.001986 | KRT10/SHARPIN/KRT2/LIPK/CLIC4/KDF1/EXPH5/DSP/WNT16/JAG1/GRHL2/ZBED2/TCHH/CYP27B1/IRF6/TP63/LCE3B/PPL/KRT16/LCE3D/AQP3/LCE3E/LCE3A | 23 |
| BP | GO:0030183 | B cell differentiation | 23/1271 | 141/18723 | 7.55E-05 | 0.002779 | 0.002291 | XBP1/CD19/POU2AF1/IKZF3/CR2/ITGA4/TCIRG1/CD79A/PTK2B/FLT3/GPR183/DNAJB9/PLCG2/CR1/MS4A1/ADA/NCKAP1L/ATM/IL21/TLR9/IL6/PTPRC/ID2 | 23 |
| BP | GO:0050921 | positive regulation of chemotaxis | 23/1271 | 141/18723 | 7.55E-05 | 0.002779 | 0.002291 | STK39/SLAMF1/TREM2/F2RL1/SCG2/PTK2B/AIF1/CXCR4/WNK1/PLA2G7/TIAM1/PTN/CXCL13/NCKAP1L/IL6/FPR2/C3AR1/CCL5/CD74/S100A14/LBP/CXCL12/EDN2 | 23 |
| BP | GO:0048771 | tissue remodeling | 23/1271 | 175/18723 | 0.001757 | 0.025742 | 0.021216 | TCIRG1/CD38/PTK2B/ADAM8/LGR4/RASSF2/ADRB2/TGM2/TNFSF11/PTN/MC4R/WNT16/JAG1/AGT/IL21/TIMP1/GPR137/GJA1/IL20RA/IL6/GPNMB/RSPO3/LEPR | 23 |
| BP | GO:0048017 | inositol lipid-mediated signaling | 23/1271 | 182/18723 | 0.002927 | 0.037903 | 0.031239 | NPR3/PIK3C2G/TPTE2/TREM2/EGF/F2RL1/HCLS1/PIK3CD/PLCD3/FLT3/PLXNB1/MYDGF/SELP/CBL/FGR/PLEKHA1/WNT16/AGT/PIK3CG/PLD1/PTPN13/CCL5/KLF4 | 23 |
| BP | GO:0008217 | regulation of blood pressure | 23/1271 | 186/18723 | 0.003852 | 0.04565 | 0.037624 | NPR3/STK39/CYBA/CYP4F12/P2RX1/F2RL1/ADRB2/WNK1/UTS2B/ACE2/GSK3A/AGT/GJA1/ADRB1/PPARG/NMU/NPPB/NPY/EMP2/APLN/ID2/F11R/EDN2 | 23 |
| BP | GO:0032677 | regulation of interleukin-8 production | 22/1271 | 102/18723 | 1.04E-06 | 9.80E-05 | 8.07E-05 | F2RL1/FCN1/CD14/IL17F/FFAR2/HSPA1B/IL1B/TLR1/LILRA2/TLR2/TLR9/TLR6/IL6/KLF4/ANXA4/CD74/TLR3/IL17D/HSPA1A/LBP/PTPRC/TLR4 | 22 |
| BP | GO:0032637 | interleukin-8 production | 22/1271 | 103/18723 | 1.24E-06 | 0.000115 | 9.45E-05 | F2RL1/FCN1/CD14/IL17F/FFAR2/HSPA1B/IL1B/TLR1/LILRA2/TLR2/TLR9/TLR6/IL6/KLF4/ANXA4/CD74/TLR3/IL17D/HSPA1A/LBP/PTPRC/TLR4 | 22 |
| BP | GO:0001909 | leukocyte mediated cytotoxicity | 22/1271 | 124/18723 | 2.89E-05 | 0.001473 | 0.001214 | STAP1/ARL8B/CXCL6/LILRB1/SLAMF7/F2RL1/CD226/VAV1/ARRB2/TYROBP/PRF1/TREM1/GZMB/IL7R/CTSH/NCKAP1L/IL21/CD1E/EMP2/PTPRC/GZMM/SERPINB4 | 22 |
| BP | GO:0051092 | positive regulation of NF-kappaB transcription factor activity | 22/1271 | 152/18723 | 0.000607 | 0.012484 | 0.010289 | CTH/IRAK2/NLRC4/AIM2/ADAM8/EDA2R/TRAPPC9/HSPA1B/TNFSF11/IL1B/PLCG2/MID2/LTF/PRKCB/AGT/TLR2/TLR9/TLR6/TLR3/RTKN2/HSPA1A/TLR4 | 22 |
| BP | GO:0120254 | olefinic compound metabolic process | 22/1271 | 153/18723 | 0.000664 | 0.01313 | 0.010821 | BCO1/ADH7/CYP4F12/ABHD6/CYP3A7/BMP5/GSTA1/STAR/CYP3A5/CYP2C18/CYP2J2/RDH12/TBXAS1/ADH1A/PNLIP/ABHD12/DKK3/AKR1B10/ELOVL2/BMP6/ALOXE3/PTGDS | 22 |
| BP | GO:0048736 | appendage development | 22/1271 | 172/18723 | 0.003073 | 0.038951 | 0.032103 | B9D1/TBC1D32/DLX5/TULP3/KDF1/KREMEN1/FGFR2/BMP4/FRAS1/SOX11/GRHL2/COMP/CRABP2/IRF6/ZNF219/RARA/TP63/HOXD10/EN1/FZD6/SFRP2/DKK1 | 22 |
| BP | GO:0060173 | limb development | 22/1271 | 172/18723 | 0.003073 | 0.038951 | 0.032103 | B9D1/TBC1D32/DLX5/TULP3/KDF1/KREMEN1/FGFR2/BMP4/FRAS1/SOX11/GRHL2/COMP/CRABP2/IRF6/ZNF219/RARA/TP63/HOXD10/EN1/FZD6/SFRP2/DKK1 | 22 |
| BP | GO:0052126 | movement in host environment | 22/1271 | 175/18723 | 0.0038 | 0.045124 | 0.037191 | SLAMF1/ARL8B/CR2/FCN1/CD86/CD80/HSPA1B/CXCR4/FCN3/CR1/MID2/CBL/CLEC4M/SELPLG/ACE2/CTSL/CXADR/SIGLEC1/CD74/HSPA1A/F11R/TNFRSF4 | 22 |
| BP | GO:0048015 | phosphatidylinositol-mediated signaling | 22/1271 | 178/18723 | 0.004665 | 0.050652 | 0.041747 | NPR3/PIK3C2G/TPTE2/TREM2/EGF/F2RL1/HCLS1/PIK3CD/PLCD3/FLT3/PLXNB1/MYDGF/SELP/CBL/FGR/PLEKHA1/WNT16/AGT/PIK3CG/PTPN13/CCL5/KLF4 | 22 |
| BP | GO:0097696 | receptor signaling pathway via STAT | 22/1271 | 181/18723 | 0.005688 | 0.056479 | 0.046549 | IL26/IL24/EGF/IL31RA/HCLS1/GHR/HES5/PTK2B/FLT3/STAT5A/IL7R/IL22RA2/AGT/PRLR/HES1/IL21/CRLF1/PPARG/RET/IL6/CCL5/PTPRC | 22 |
| BP | GO:1903557 | positive regulation of tumor necrosis factor superfamily cytokine production | 21/1271 | 107/18723 | 8.84E-06 | 0.000554 | 0.000456 | CYBA/CD86/CD14/IL17F/LILRA5/FZD5/ADAM8/LY96/TYROBP/PLCG2/TLR1/IL17A/LILRA2/CYBB/TLR2/TLR9/IL6/TLR3/LBP/PTPRC/TLR4 | 21 |
| BP | GO:0046632 | alpha-beta T cell differentiation | 21/1271 | 112/18723 | 1.84E-05 | 0.001024 | 0.000844 | IRF4/BATF/GPR18/PSMB11/CD86/CD80/GPR183/PLA2G2D/RORC/HLX/EOMES/ITK/TNFSF8/ADA/NCKAP1L/IL21/PRDM1/CTSL/IL6/TOX/RARA | 21 |
| BP | GO:0002761 | regulation of myeloid leukocyte differentiation | 21/1271 | 120/18723 | 5.37E-05 | 0.002251 | 0.001855 | TREM2/LILRB1/HCLS1/CTNNBIP1/LILRB3/PLA2G3/TYROBP/RASSF2/TNFSF11/MYC/OCSTAMP/IL17A/TRIB1/LTF/GPR137/RARA/CD74/TLR3/TLR4/ID2/JUN | 21 |
| BP | GO:0002688 | regulation of leukocyte chemotaxis | 21/1271 | 122/18723 | 6.89E-05 | 0.002645 | 0.00218 | STK39/STAP1/SLAMF1/GPR18/F2RL1/PTK2B/AIF1/WNK1/PLA2G7/PTN/CXCL13/NCKAP1L/IL6/FPR2/C3AR1/CCL5/CD74/S100A14/LBP/CXCL12/EDN2 | 21 |
| BP | GO:0006690 | icosanoid metabolic process | 21/1271 | 123/18723 | 7.79E-05 | 0.00283 | 0.002332 | CYP4F12/DPEP1/CYP4F8/ABHD6/PLA2G3/IL1B/GSTA1/ALOX5AP/CYP2C18/CYP2J2/TBXAS1/ABHD12/TLR2/LTC4S/PTGR1/PTGES/GGT7/CD74/ALOXE3/PTGDS/EDN2 | 21 |
| BP | GO:0006986 | response to unfolded protein | 21/1271 | 137/18723 | 0.000367 | 0.008628 | 0.007111 | XBP1/CTH/EDEM1/ERLEC1/HSPA1L/HSPA13/HSPA1B/RHBDD1/DNAJB9/HSPA2/HERPUD1/CHAC1/RHBDD2/ERN1/BHLHA15/DNAJC3/COMP/DNAJB1/FICD/HSPA1A/HSPA4L | 21 |
| BP | GO:1903038 | negative regulation of leukocyte cell-cell adhesion | 21/1271 | 141/18723 | 0.000545 | 0.011466 | 0.00945 | CTLA4/LILRB1/CD86/CD80/MIA3/LAX1/PLA2G2D/NRARP/CR1/LILRB2/WNK1/HLX/BMP4/IL2RA/NCKAP1L/KLF4/CD74/GPNMB/IL20RB/CXCL12/VSIG4 | 21 |
| BP | GO:0001890 | placenta development | 21/1271 | 144/18723 | 0.000723 | 0.01402 | 0.011555 | DNAJB6/KRT8/PLCD3/FZD5/SOX15/BMP5/FGFR2/PTN/EOMES/GJB5/ADA/HES1/PRDM1/GJA1/LHX3/GRHL2/PPARG/CYP27B1/E2F8/RSPO3/ITGB8 | 21 |
| BP | GO:0035148 | tube formation | 21/1271 | 148/18723 | 0.001037 | 0.018227 | 0.015022 | EGF/HOXA1/VANGL2/TULP3/PIK3CD/HES5/IRX2/TGM2/BMP5/FGFR2/PODXL/BMP4/SOX11/CELSR1/GRHL2/RET/YWHAZ/RARA/LRP2/FZD6/SFRP2 | 21 |
| BP | GO:0044409 | entry into host | 21/1271 | 151/18723 | 0.001343 | 0.021978 | 0.018114 | SLAMF1/CR2/FCN1/CD86/CD80/HSPA1B/CXCR4/FCN3/CR1/MID2/CBL/CLEC4M/SELPLG/ACE2/CTSL/CXADR/SIGLEC1/CD74/HSPA1A/F11R/TNFRSF4 | 21 |
| BP | GO:0003205 | cardiac chamber development | 21/1271 | 161/18723 | 0.002981 | 0.038329 | 0.03159 | TBX1/NPY2R/ROBO2/MESP1/PPP1R13L/BMP5/STRA6/FGFR2/BMP4/DSP/FOXH1/JAG1/GSK3A/SOX11/HES1/PRDM1/TGFBR3/GRHL2/LRP2/MYL3/SFRP2 | 21 |
| BP | GO:0002706 | regulation of lymphocyte mediated immunity | 21/1271 | 168/18723 | 0.004933 | 0.051832 | 0.042719 | SLAMF1/TREM2/LILRB1/CR2/CD226/CR1L/VAV1/ARRB2/SUSD4/FZD5/C3/IL1B/CR1/IL7R/NCKAP1L/IL21/CD1E/IL6/IL20RB/PTPRC/SERPINB4 | 21 |
| BP | GO:0048469 | cell maturation | 21/1271 | 171/18723 | 0.006044 | 0.058213 | 0.047978 | BFSP2/HES5/FZD5/PTK2B/ROPN1/PLA2G3/ROPN1B/BCL11A/NPPC/C3/SPINK1/BHLHA15/C1QA/HES1/IL21/PPARG/SOX10/RET/BNC1/LHX6/PAEP | 21 |
| BP | GO:0070098 | chemokine-mediated signaling pathway | 20/1271 | 88/18723 | 1.34E-06 | 0.000122 | 0.0001 | STK39/TREM2/CXCL6/CXCL1/CCL18/MPL/CCR3/CXCL3/PTK2B/CXCR4/WNK1/CXCR1/ACKR4/CXCL2/CCL13/CXCL13/PF4V1/CCL5/CCL11/CXCL12 | 20 |
| BP | GO:1990868 | response to chemokine | 20/1271 | 97/18723 | 6.60E-06 | 0.000429 | 0.000353 | STK39/TREM2/CXCL6/CXCL1/CCL18/MPL/CCR3/CXCL3/PTK2B/CXCR4/WNK1/CXCR1/ACKR4/CXCL2/CCL13/CXCL13/PF4V1/CCL5/CCL11/CXCL12 | 20 |
| BP | GO:1990869 | cellular response to chemokine | 20/1271 | 97/18723 | 6.60E-06 | 0.000429 | 0.000353 | STK39/TREM2/CXCL6/CXCL1/CCL18/MPL/CCR3/CXCL3/PTK2B/CXCR4/WNK1/CXCR1/ACKR4/CXCL2/CCL13/CXCL13/PF4V1/CCL5/CCL11/CXCL12 | 20 |
| BP | GO:0033559 | unsaturated fatty acid metabolic process | 20/1271 | 116/18723 | 9.93E-05 | 0.003426 | 0.002824 | CYP4F12/CYP4F8/ABHD6/ELOVL4/PLA2G3/IL1B/ELOVL6/GSTA1/CYP2C18/CYP2J2/TBXAS1/DEGS1/ABHD12/PTGR1/PTGES/ELOVL2/CD74/ALOXE3/PTGDS/EDN2 | 20 |
| BP | GO:0055123 | digestive system development | 20/1271 | 137/18723 | 0.000944 | 0.016867 | 0.013901 | BARX1/EPHB3/LGR4/HLX/STRA6/FGFR2/BMP4/ADA/SOX11/KLF5/HES1/FOXE1/PRDM1/SOX10/RET/TP63/DNAAF1/NPY/SFRP2/ID2 | 20 |
| BP | GO:0035296 | regulation of tube diameter | 20/1271 | 141/18723 | 0.001355 | 0.021978 | 0.018114 | SLC6A4/NPR3/P2RY1/P2RX1/F2RL1/KCNA5/CD38/ADRB2/TBXAS1/UTS2B/ACE2/AGT/GJA1/ADRB1/COMP/DOCK4/NPPB/RGS2/APLN/EDN2 | 20 |
| BP | GO:0097746 | blood vessel diameter maintenance | 20/1271 | 141/18723 | 0.001355 | 0.021978 | 0.018114 | SLC6A4/NPR3/P2RY1/P2RX1/F2RL1/KCNA5/CD38/ADRB2/TBXAS1/UTS2B/ACE2/AGT/GJA1/ADRB1/COMP/DOCK4/NPPB/RGS2/APLN/EDN2 | 20 |
| BP | GO:0035150 | regulation of tube size | 20/1271 | 142/18723 | 0.001479 | 0.023294 | 0.019198 | SLC6A4/NPR3/P2RY1/P2RX1/F2RL1/KCNA5/CD38/ADRB2/TBXAS1/UTS2B/ACE2/AGT/GJA1/ADRB1/COMP/DOCK4/NPPB/RGS2/APLN/EDN2 | 20 |
| BP | GO:0014065 | phosphatidylinositol 3-kinase signaling | 20/1271 | 144/18723 | 0.001755 | 0.025742 | 0.021216 | PIK3C2G/TPTE2/TREM2/EGF/F2RL1/HCLS1/PIK3CD/FLT3/PLXNB1/MYDGF/SELP/CBL/FGR/PLEKHA1/WNT16/AGT/PIK3CG/PTPN13/CCL5/KLF4 | 20 |
| BP | GO:0046718 | viral entry into host cell | 20/1271 | 144/18723 | 0.001755 | 0.025742 | 0.021216 | SLAMF1/CR2/FCN1/CD86/CD80/HSPA1B/CXCR4/FCN3/CR1/MID2/CLEC4M/SELPLG/ACE2/CTSL/CXADR/SIGLEC1/CD74/HSPA1A/F11R/TNFRSF4 | 20 |
| BP | GO:0050829 | defense response to Gram-negative bacterium | 19/1271 | 88/18723 | 5.50E-06 | 0.000379 | 0.000313 | TREM2/CXCL6/F2RL1/FCN2/IL17F/RNASE6/TREM1/PRB3/IL17A/SELP/LTF/TLR9/IL6/CAMP/LCE3B/LYZ/LBP/TLR4/LCE3A | 19 |
| BP | GO:0002690 | positive regulation of leukocyte chemotaxis | 19/1271 | 94/18723 | 1.51E-05 | 0.000864 | 0.000712 | STK39/SLAMF1/F2RL1/PTK2B/AIF1/WNK1/PLA2G7/PTN/CXCL13/NCKAP1L/IL6/FPR2/C3AR1/CCL5/CD74/S100A14/LBP/CXCL12/EDN2 | 19 |
| BP | GO:0045639 | positive regulation of myeloid cell differentiation | 19/1271 | 103/18723 | 5.73E-05 | 0.002362 | 0.001947 | TREM2/ACVR1B/HCLS1/MPL/CTNNBIP1/PITHD1/PLA2G3/TYROBP/HSPA1B/TNFSF11/OCSTAMP/IL17A/TRIB1/JAG1/NCKAP1L/CD74/HSPA1A/ID2/JUN | 19 |
| BP | GO:0062207 | regulation of pattern recognition receptor signaling pathway | 19/1271 | 105/18723 | 7.52E-05 | 0.002779 | 0.002291 | CYBA/TREM2/IRF4/F2RL1/ARRB2/LGR4/HSPA1B/TLR1/BIRC3/LILRA2/LTF/SLC15A2/TLR2/TLR9/TLR6/TLR3/HSPA1A/LBP/TLR4 | 19 |
| BP | GO:1903707 | negative regulation of hemopoiesis | 19/1271 | 106/18723 | 8.59E-05 | 0.003011 | 0.002482 | CTLA4/LILRB1/TMEM176B/LILRB3/TMEM176A/MYC/NRARP/CR1/HLX/TRIB1/LTF/BMP4/GPR137/RARA/CD74/TLR3/IL17D/TLR4/ID2 | 19 |
| BP | GO:0043406 | positive regulation of MAP kinase activity | 19/1271 | 112/18723 | 0.000183 | 0.005303 | 0.004371 | EGF/MST1R/GHR/FZD5/PTK2B/ADAM8/FLT3/TNFSF11/IL1B/ERN1/TIAM1/ARHGEF5/AJUBA/PIK3CG/TLR9/FZD10/TLR6/TLR4/DKK1 | 19 |
| BP | GO:0071675 | regulation of mononuclear cell migration | 19/1271 | 115/18723 | 0.00026 | 0.006925 | 0.005707 | STK39/SLAMF1/ITGA4/PTK2B/ADAM8/AIF1/MIA3/CD200/BMP5/WNK1/AIRE/PLA2G7/CXCL13/C10orf99/FPR2/C3AR1/CCL5/S100A14/CXCL12 | 19 |
| BP | GO:0003206 | cardiac chamber morphogenesis | 19/1271 | 121/18723 | 0.000505 | 0.010836 | 0.008931 | TBX1/NPY2R/ROBO2/MESP1/PPP1R13L/BMP5/FGFR2/BMP4/DSP/FOXH1/JAG1/GSK3A/SOX11/HES1/TGFBR3/GRHL2/LRP2/MYL3/SFRP2 | 19 |
| BP | GO:0019730 | antimicrobial humoral response | 19/1271 | 122/18723 | 0.000561 | 0.011711 | 0.009652 | CXCL6/CXCL1/JCHAIN/IL17F/CXCL3/RNASE6/IL17A/CXCL2/PGC/LTF/CCL13/CXCL13/PF4V1/GNLY/WFDC2/CAMP/KLK7/LYZ/PI3 | 19 |
| BP | GO:0003231 | cardiac ventricle development | 19/1271 | 123/18723 | 0.000622 | 0.012506 | 0.010307 | NPY2R/ROBO2/MESP1/PPP1R13L/STRA6/FGFR2/BMP4/DSP/FOXH1/JAG1/GSK3A/SOX11/HES1/PRDM1/TGFBR3/GRHL2/LRP2/MYL3/SFRP2 | 19 |
| BP | GO:0030168 | platelet activation | 19/1271 | 123/18723 | 0.000622 | 0.012506 | 0.010307 | P2RY1/P2RX1/MPL/VAV1/PLEK/MERTK/PLCG2/SELP/PF4V1/PIK3CG/SAA1/COMP/IL6/RAP2B/FCER1G/TLR4/FZD6/CD9/F11R | 19 |
| BP | GO:0048565 | digestive tract development | 19/1271 | 127/18723 | 0.000927 | 0.016639 | 0.013714 | EPHB3/LGR4/HLX/STRA6/FGFR2/BMP4/ADA/SOX11/KLF5/HES1/FOXE1/PRDM1/SOX10/RET/TP63/DNAAF1/NPY/SFRP2/ID2 | 19 |
| BP | GO:0045089 | positive regulation of innate immune response | 19/1271 | 131/18723 | 0.001353 | 0.021978 | 0.018114 | ZBP1/NLRC4/CD226/FCN1/VAV1/AIM2/FFAR2/ADAM8/TYROBP/PLCG2/PAK3/MNDA/LILRA2/IL21/FPR2/CCL5/MMP12/LBP/TLR4 | 19 |
| BP | GO:0050853 | B cell receptor signaling pathway | 19/1271 | 131/18723 | 0.001353 | 0.021978 | 0.018114 | CD19/STAP1/KLHL6/CTLA4/LAT2/IGLL1/CD38/CD79A/BANK1/PIK3CD/LPXN/PLCG2/MNDA/MS4A1/PRKCB/PLEKHA1/ITK/NCKAP1L/PTPRC | 19 |
| BP | GO:0031644 | regulation of nervous system process | 19/1271 | 144/18723 | 0.004042 | 0.047027 | 0.038759 | CCK/SHISA9/ITGAX/NPY2R/CST7/UNC13B/PTK2B/ADRB2/SMR3B/TMEM108/PARD3/SH3GL1/CUX2/AGT/NCMAP/NMU/SOX10/SLC8A2/NRXN1 | 19 |
| BP | GO:0016331 | morphogenesis of embryonic epithelium | 19/1271 | 147/18723 | 0.00507 | 0.052744 | 0.043471 | VANGL2/TULP3/HES5/IRX2/BMP5/KDF1/FGFR2/BMP4/WNT16/WNT2B/SOX11/CELSR1/GRHL2/RET/RARA/TP63/LRP2/FZD6/SFRP2 | 19 |
| BP | GO:0050764 | regulation of phagocytosis | 18/1271 | 95/18723 | 6.18E-05 | 0.002459 | 0.002027 | SPACA3/CYBA/STAP1/TREM2/F2RL1/TGM2/C3/IL1B/MERTK/PLCG2/IL2RG/FGR/NCKAP1L/TLR2/FPR2/FCER1G/TULP1/PTPRC | 18 |
| BP | GO:0032602 | chemokine production | 18/1271 | 99/18723 | 0.000108 | 0.003591 | 0.00296 | TREM2/CXCL6/F2RL1/IL17F/FFAR2/AIF1/TREM1/IL1B/IL17A/AIRE/TLR2/TLR9/IL6/KLF4/CD74/TLR3/LBP/TLR4 | 18 |
| BP | GO:1902106 | negative regulation of leukocyte differentiation | 18/1271 | 102/18723 | 0.00016 | 0.004832 | 0.003983 | CTLA4/LILRB1/TMEM176B/LILRB3/TMEM176A/MYC/NRARP/CR1/HLX/TRIB1/LTF/BMP4/GPR137/RARA/CD74/TLR3/TLR4/ID2 | 18 |
| BP | GO:0032760 | positive regulation of tumor necrosis factor production | 18/1271 | 103/18723 | 0.000182 | 0.005303 | 0.004371 | CYBA/CD14/LILRA5/FZD5/LY96/TYROBP/PLCG2/TLR1/IL17A/LILRA2/CYBB/TLR2/TLR9/IL6/TLR3/LBP/PTPRC/TLR4 | 18 |
| BP | GO:0045621 | positive regulation of lymphocyte differentiation | 18/1271 | 104/18723 | 0.000207 | 0.005808 | 0.004787 | XBP1/RHOH/CD86/CD80/ADAM8/ZMIZ1/CR1/LILRB2/HLX/IL7R/IL2RA/PCK1/ADA/NCKAP1L/TOX/RARA/CD74/PTPRC | 18 |
| BP | GO:0071887 | leukocyte apoptotic process | 18/1271 | 106/18723 | 0.000264 | 0.006957 | 0.005734 | LILRB1/HCLS1/PIK3CD/ADAM8/CD3G/MERTK/IL7R/BMP4/IL2RA/ADA/CTSL/EFNA1/IL6/CCL5/CD74/AURKB/CDKN2A/CXCL12 | 18 |
| BP | GO:0002526 | acute inflammatory response | 18/1271 | 112/18723 | 0.000528 | 0.011152 | 0.009191 | F12/IL31RA/CTNNBIP1/FFAR2/ADAM8/C3/TNFSF11/TREM1/PLA2G2D/SAA2/IL1B/ALOX5AP/PIK3CG/SAA1/PTGES/IL6/LBP/IL20RB | 18 |
| BP | GO:0072175 | epithelial tube formation | 18/1271 | 132/18723 | 0.003552 | 0.043058 | 0.035487 | EGF/VANGL2/TULP3/PIK3CD/HES5/IRX2/BMP5/FGFR2/PODXL/BMP4/SOX11/CELSR1/GRHL2/RET/RARA/LRP2/FZD6/SFRP2 | 18 |
| BP | GO:0003158 | endothelium development | 18/1271 | 136/18723 | 0.004891 | 0.051632 | 0.042555 | ARHGEF26/ITGAX/F2RL1/MESP1/CXCR4/IL1B/CLIC4/TNMD/PTN/PECAM1/BMP4/COL15A1/JAG1/GJA1/CLDN3/BMP6/MARVELD2/F11R | 18 |
| BP | GO:0072073 | kidney epithelium development | 18/1271 | 136/18723 | 0.004891 | 0.051632 | 0.042555 | ROBO2/CTNNBIP1/HES5/IRX2/LGR4/MYC/EPCAM/FGFR2/PECAM1/PODXL/BMP4/JAG1/WNT2B/AGT/HES1/CRLF1/RET/RARA | 18 |
| BP | GO:0050714 | positive regulation of protein secretion | 18/1271 | 137/18723 | 0.005284 | 0.053533 | 0.044121 | TREM2/F2RL1/SYTL4/UNC13B/CD38/OXCT1/ADAM8/NNAT/EXPH5/SYBU/TLR2/GJA1/NMU/BMP6/PARD6A/VSNL1/TLR4/ANKRD1 | 18 |
| BP | GO:0032757 | positive regulation of interleukin-8 production | 17/1271 | 62/18723 | 4.93E-07 | 5.48E-05 | 4.52E-05 | F2RL1/FCN1/CD14/FFAR2/HSPA1B/IL1B/TLR1/LILRA2/TLR2/TLR9/IL6/CD74/TLR3/IL17D/HSPA1A/LBP/TLR4 | 17 |
| BP | GO:0045582 | positive regulation of T cell differentiation | 17/1271 | 91/18723 | 0.000118 | 0.003817 | 0.003146 | XBP1/RHOH/CD86/CD80/ADAM8/ZMIZ1/CR1/LILRB2/HLX/IL7R/IL2RA/PCK1/ADA/NCKAP1L/RARA/CD74/PTPRC | 17 |
| BP | GO:0032642 | regulation of chemokine production | 17/1271 | 98/18723 | 0.000299 | 0.00757 | 0.006239 | TREM2/CXCL6/F2RL1/IL17F/FFAR2/AIF1/IL1B/IL17A/AIRE/TLR2/TLR9/IL6/KLF4/CD74/TLR3/LBP/TLR4 | 17 |
| BP | GO:0031341 | regulation of cell killing | 17/1271 | 99/18723 | 0.000338 | 0.008227 | 0.006781 | STAP1/CXCL6/LILRB1/F2RL1/CD226/CR1L/VAV1/ARRB2/TYROBP/PRF1/IL7R/BCL2L1/NCKAP1L/IL21/CD1E/PTPRC/SERPINB4 | 17 |
| BP | GO:0050830 | defense response to Gram-positive bacterium | 17/1271 | 101/18723 | 0.000431 | 0.009585 | 0.0079 | FCN2/IL17F/RNASE6/IL17A/IL7R/FGR/TNFSF8/C10orf99/TLR2/IL6/GBP6/CAMP/LCE3B/LYZ/LBP/GBP2/LCE3A | 17 |
| BP | GO:0032963 | collagen metabolic process | 17/1271 | 104/18723 | 0.000611 | 0.012506 | 0.010307 | MMP3/MMP1/CTSS/ARRB2/MMP27/MMP10/NPPC/SERPINB7/BMP4/TNS2/TRAM2/CTSL/MMP9/IL6/MMP12/MMP13/MMP7 | 17 |
| BP | GO:0032526 | response to retinoic acid | 17/1271 | 107/18723 | 0.000852 | 0.01563 | 0.012882 | SLC6A4/TBX1/CD38/PTK2B/FGFR2/PCK1/CTSH/ATM/GJA1/FZD10/PTGES/RET/BMP6/RXRG/KLF4/RARA/AQP3 | 17 |
| BP | GO:0002456 | T cell mediated immunity | 17/1271 | 109/18723 | 0.001054 | 0.018469 | 0.015222 | SLAMF1/MYO1G/LILRB1/FZD5/PRF1/IL1B/AIRE/IL7R/CTSH/JAG1/NCKAP1L/CD1E/IL6/EMP2/IL20RB/PTPRC/GZMM | 17 |
| BP | GO:0006805 | xenobiotic metabolic process | 17/1271 | 111/18723 | 0.001295 | 0.021525 | 0.01774 | GSTA5/GSTA4/CYP4F12/AADAC/CYP2W1/CYP3A7/ACSM1/GSTA1/RORC/STAR/CYP3A5/AHRR/CYP2C18/CYP2J2/NQO1/GSTA2/GSTA3 | 17 |
| BP | GO:0032609 | interferon-gamma production | 17/1271 | 112/18723 | 0.001433 | 0.022795 | 0.018787 | SLAMF1/LILRB1/F2RL1/CD226/SLC7A5/CD14/FZD5/IL1B/CR1/PDE4B/BTN3A1/IL21/TLR9/RARA/TLR3/IL20RB/TLR4 | 17 |
| BP | GO:0032649 | regulation of interferon-gamma production | 17/1271 | 112/18723 | 0.001433 | 0.022795 | 0.018787 | SLAMF1/LILRB1/F2RL1/CD226/SLC7A5/CD14/FZD5/IL1B/CR1/PDE4B/BTN3A1/IL21/TLR9/RARA/TLR3/IL20RB/TLR4 | 17 |
| BP | GO:0002286 | T cell activation involved in immune response | 17/1271 | 114/18723 | 0.001744 | 0.025742 | 0.021216 | IRF4/LILRB1/BATF/F2RL1/CD86/CD80/GPR183/RORC/HLX/EOMES/PCK1/NCKAP1L/IL21/IL6/RARA/CD74/FCER1G | 17 |
| BP | GO:0021782 | glial cell development | 17/1271 | 116/18723 | 0.002111 | 0.02989 | 0.024635 | TREM2/MAPT/HES5/PARD3/C1QA/SOX11/PHGDH/TLR2/NCMAP/SH3TC2/NDRG1/SOX10/IL6/FPR2/MXRA8/TLR4/CD9 | 17 |
| BP | GO:0035967 | cellular response to topologically incorrect protein | 17/1271 | 116/18723 | 0.002111 | 0.02989 | 0.024635 | XBP1/CTH/ERLEC1/HSPA1L/HSPA13/HSPA1B/RHBDD1/DNAJB9/HSPA2/HERPUD1/UGGT1/RHBDD2/ERN1/SDF2L1/BHLHA15/FICD/HSPA1A | 17 |
| BP | GO:0050868 | negative regulation of T cell activation | 17/1271 | 122/18723 | 0.003619 | 0.043355 | 0.035733 | CTLA4/LILRB1/CD86/CD80/LAX1/PLA2G2D/NRARP/CR1/LILRB2/HLX/BMP4/IL2RA/NCKAP1L/CD74/GPNMB/IL20RB/VSIG4 | 17 |
| BP | GO:0030888 | regulation of B cell proliferation | 16/1271 | 64/18723 | 4.04E-06 | 0.000294 | 0.000243 | CTLA4/MZB1/IKZF3/CD38/TYROBP/GPR183/MNDA/ADA/NCKAP1L/ATM/IL21/TLR9/CD74/PTPRC/TLR4/TNFRSF4 | 16 |
| BP | GO:0072678 | T cell migration | 16/1271 | 66/18723 | 6.22E-06 | 0.000413 | 0.000341 | STK39/MYO1G/ITGA4/PIK3CD/ADAM8/AIF1/GPR183/CD200/WNK1/AIRE/CXCL13/C10orf99/PIK3CG/CCL5/CXCL12/F11R | 16 |
| BP | GO:0070830 | bicellular tight junction assembly | 16/1271 | 70/18723 | 1.39E-05 | 0.00081 | 0.000668 | FZD5/POF1B/OCLN/CLDN22/PARD3/IL17A/PECAM1/MARVELD3/CLDN17/MPP7/GJA1/GRHL2/CLDN3/MARVELD2/CLDN10/F11R | 16 |
| BP | GO:0033627 | cell adhesion mediated by integrin | 16/1271 | 72/18723 | 2.03E-05 | 0.001108 | 0.000913 | SKAP1/ITGA4/PDE3B/LPXN/WNK1/PODXL/CXCL13/ADA/PIK3CG/NCKAP1L/EFNA1/RET/CCL5/FERMT1/ITGB8/SFRP2 | 16 |
| BP | GO:0120192 | tight junction assembly | 16/1271 | 74/18723 | 2.92E-05 | 0.001474 | 0.001215 | FZD5/POF1B/OCLN/CLDN22/PARD3/IL17A/PECAM1/MARVELD3/CLDN17/MPP7/GJA1/GRHL2/CLDN3/MARVELD2/CLDN10/F11R | 16 |
| BP | GO:0043297 | apical junction assembly | 16/1271 | 78/18723 | 5.77E-05 | 0.002362 | 0.001947 | FZD5/POF1B/OCLN/CLDN22/PARD3/IL17A/PECAM1/MARVELD3/CLDN17/MPP7/GJA1/GRHL2/CLDN3/MARVELD2/CLDN10/F11R | 16 |
| BP | GO:0120193 | tight junction organization | 16/1271 | 80/18723 | 7.96E-05 | 0.002836 | 0.002337 | FZD5/POF1B/OCLN/CLDN22/PARD3/IL17A/PECAM1/MARVELD3/CLDN17/MPP7/GJA1/GRHL2/CLDN3/MARVELD2/CLDN10/F11R | 16 |
| BP | GO:0050672 | negative regulation of lymphocyte proliferation | 16/1271 | 83/18723 | 0.000126 | 0.004015 | 0.003309 | CTLA4/LILRB1/CD86/CD80/TYROBP/PLA2G2D/CR1/LILRB2/MNDA/BMP4/IL2RA/SOX11/ATM/GPNMB/IL20RB/VSIG4 | 16 |
| BP | GO:0032945 | negative regulation of mononuclear cell proliferation | 16/1271 | 84/18723 | 0.000146 | 0.004535 | 0.003738 | CTLA4/LILRB1/CD86/CD80/TYROBP/PLA2G2D/CR1/LILRB2/MNDA/BMP4/IL2RA/SOX11/ATM/GPNMB/IL20RB/VSIG4 | 16 |
| BP | GO:0001776 | leukocyte homeostasis | 16/1271 | 87/18723 | 0.000223 | 0.006242 | 0.005144 | TNFRSF17/CXCL6/TCIRG1/MPL/PIK3CD/FLT3/GPR183/MERTK/PDE4B/IL2RA/ADA/NCKAP1L/GAPT/GPR174/IL6/CD74 | 16 |
| BP | GO:0046849 | bone remodeling | 16/1271 | 90/18723 | 0.000334 | 0.008197 | 0.006755 | TCIRG1/CD38/PTK2B/ADAM8/LGR4/RASSF2/ADRB2/TNFSF11/PTN/MC4R/WNT16/GPR137/GJA1/IL20RA/IL6/LEPR | 16 |
| BP | GO:0070664 | negative regulation of leukocyte proliferation | 16/1271 | 90/18723 | 0.000334 | 0.008197 | 0.006755 | CTLA4/LILRB1/CD86/CD80/TYROBP/PLA2G2D/CR1/LILRB2/MNDA/BMP4/IL2RA/SOX11/ATM/GPNMB/IL20RB/VSIG4 | 16 |
| BP | GO:0048640 | negative regulation of developmental growth | 16/1271 | 112/18723 | 0.003648 | 0.04342 | 0.035786 | SLC6A4/MAP2/TLL2/FSTL4/NKD1/ULK2/ADRB2/BCL11A/ARHGAP4/MEIS1/BMP4/SEMA7A/GSK3A/GJA1/ADRB1/RGS2 | 16 |
| BP | GO:0006720 | isoprenoid metabolic process | 16/1271 | 116/18723 | 0.005171 | 0.053212 | 0.043856 | BCO1/ADH7/PMVK/CYP2W1/CYP3A7/ALDH3A2/STAR/CYP3A5/CYP2C18/RDH12/ADH1A/PNLIP/AKR1B10/CRABP2/HMGCS1/LRP2 | 16 |
| BP | GO:0045446 | endothelial cell differentiation | 16/1271 | 118/18723 | 0.006107 | 0.058709 | 0.048387 | ARHGEF26/F2RL1/MESP1/CXCR4/IL1B/CLIC4/TNMD/PTN/PECAM1/BMP4/COL15A1/JAG1/CLDN3/BMP6/MARVELD2/F11R | 16 |
| BP | GO:0050766 | positive regulation of phagocytosis | 15/1271 | 66/18723 | 2.76E-05 | 0.00142 | 0.00117 | SPACA3/CYBA/STAP1/TREM2/F2RL1/C3/IL1B/MERTK/PLCG2/IL2RG/NCKAP1L/FPR2/FCER1G/TULP1/PTPRC | 15 |
| BP | GO:0034121 | regulation of toll-like receptor signaling pathway | 15/1271 | 75/18723 | 0.000132 | 0.004131 | 0.003405 | CYBA/TREM2/IRF4/F2RL1/ARRB2/LGR4/TLR1/BIRC3/LILRA2/LTF/TLR2/TLR9/TLR6/TLR3/LBP | 15 |
| BP | GO:0061844 | antimicrobial humoral immune response mediated by antimicrobial peptide | 15/1271 | 79/18723 | 0.000242 | 0.006681 | 0.005507 | CXCL6/CXCL1/IL17F/CXCL3/RNASE6/IL17A/CXCL2/PGC/LTF/CCL13/CXCL13/PF4V1/GNLY/CAMP/KLK7 | 15 |
| BP | GO:2000106 | regulation of leukocyte apoptotic process | 15/1271 | 81/18723 | 0.000321 | 0.008068 | 0.006649 | LILRB1/HCLS1/PIK3CD/ADAM8/CD3G/MERTK/IL7R/BMP4/ADA/EFNA1/CCL5/CD74/AURKB/CDKN2A/CXCL12 | 15 |
| BP | GO:0002312 | B cell activation involved in immune response | 15/1271 | 82/18723 | 0.000369 | 0.008628 | 0.007111 | XBP1/CD19/POU2AF1/CD180/BATF/PTK2B/GPR183/PLCG2/CR1/ADA/IL21/GAPT/IL6/PTPRC/TLR4 | 15 |
| BP | GO:0002275 | myeloid cell activation involved in immune response | 15/1271 | 91/18723 | 0.001145 | 0.01957 | 0.01613 | SLAMF1/LAT2/TREM2/F2RL1/PIK3CD/PLA2G3/TYROBP/PLCG2/CBL/LILRA2/FGR/PIK3CG/FCER1G/LBP/PTGDS | 15 |
| BP | GO:0060349 | bone morphogenesis | 15/1271 | 93/18723 | 0.001436 | 0.022795 | 0.018787 | DLX5/GHR/NPPC/ANXA6/FGFR2/LTF/BMP4/NEUROG1/COMP/RIPPLY2/BMP6/RARA/MMP13/SFRP4/SFRP2 | 15 |
| BP | GO:0003073 | regulation of systemic arterial blood pressure | 15/1271 | 96/18723 | 0.001989 | 0.028593 | 0.023566 | CYBA/CYP4F12/F2RL1/ADRB2/WNK1/ACE2/GSK3A/AGT/GJA1/ADRB1/NMU/NPPB/EMP2/APLN/EDN2 | 15 |
| BP | GO:0034620 | cellular response to unfolded protein | 15/1271 | 96/18723 | 0.001989 | 0.028593 | 0.023566 | XBP1/CTH/ERLEC1/HSPA1L/HSPA13/HSPA1B/RHBDD1/DNAJB9/HSPA2/HERPUD1/RHBDD2/ERN1/BHLHA15/FICD/HSPA1A | 15 |
| BP | GO:0006721 | terpenoid metabolic process | 15/1271 | 97/18723 | 0.002208 | 0.03071 | 0.02531 | BCO1/ADH7/CYP2W1/CYP3A7/ALDH3A2/STAR/CYP3A5/CYP2C18/RDH12/ADH1A/PNLIP/AKR1B10/CRABP2/HMGCS1/LRP2 | 15 |
| BP | GO:0002367 | cytokine production involved in immune response | 15/1271 | 98/18723 | 0.002448 | 0.033025 | 0.027219 | SLAMF1/LILRB1/F2RL1/CD226/SLC7A5/FFAR2/FZD5/TREM1/IL1B/PLCG2/SEMA7A/IL6/CD74/TLR3/TLR4 | 15 |
| BP | GO:0042102 | positive regulation of T cell proliferation | 15/1271 | 101/18723 | 0.003297 | 0.040852 | 0.03367 | SLAMF1/RASAL3/CD86/CD80/AIF1/IL1B/CLECL1/LILRB2/IL2RA/NCKAP1L/HES1/IL21/IL6/CCL5/PTPRC | 15 |
| BP | GO:0035710 | CD4-positive, alpha-beta T cell activation | 15/1271 | 102/18723 | 0.003629 | 0.043381 | 0.035754 | IRF4/BATF/CD86/TCIRG1/CD80/GPR183/PLA2G2D/RORC/HLX/NCKAP1L/IL21/CTSL/IL6/TOX/RARA | 15 |
| BP | GO:0048661 | positive regulation of smooth muscle cell proliferation | 15/1271 | 104/18723 | 0.004375 | 0.05031 | 0.041465 | CYBA/HES5/AIF1/TGM2/ERN1/FGFR2/BMP4/AGT/GJA1/MMP9/IL6/CCL5/TLR4/ID2/JUN | 15 |
| BP | GO:0099565 | chemical synaptic transmission, postsynaptic | 15/1271 | 106/18723 | 0.005241 | 0.053533 | 0.044121 | CHRNA3/NPY2R/P2RX1/P2RX5/UNC13B/ARRB2/PTK2B/ADRB2/TMEM108/SH3GL1/GRIK2/CUX2/GSK3A/SLC8A2/NRXN1 | 15 |
| BP | GO:0036503 | ERAD pathway | 15/1271 | 107/18723 | 0.005723 | 0.056722 | 0.046749 | CLGN/EDEM1/ERLEC1/SEL1L/RHBDD1/DNAJB9/HERPUD1/UGGT1/DNAJC10/FBXO44/UBE2J1/RHBDD2/SDF2L1/MAN1A1/SVIP | 15 |
| BP | GO:0002920 | regulation of humoral immune response | 14/1271 | 45/18723 | 9.33E-07 | 8.92E-05 | 7.35E-05 | TREM2/CR2/CR1L/SUSD4/IL17F/C3/IL1B/CR1/IL17A/PGC/CXCL13/KLK7/PTPRC/VSIG4 | 14 |
| BP | GO:0001774 | microglial cell activation | 14/1271 | 47/18723 | 1.68E-06 | 0.00015 | 0.000124 | STAP1/TREM2/MAPT/CST7/AIF1/TYROBP/TLR1/C1QA/TLR2/TLR6/IL6/FPR2/TLR3/PTPRC | 14 |
| BP | GO:0033628 | regulation of cell adhesion mediated by integrin | 14/1271 | 48/18723 | 2.22E-06 | 0.000189 | 0.000156 | SKAP1/PDE3B/LPXN/WNK1/PODXL/CXCL13/ADA/PIK3CG/NCKAP1L/EFNA1/RET/CCL5/FERMT1/SFRP2 | 14 |
| BP | GO:0031663 | lipopolysaccharide-mediated signaling pathway | 14/1271 | 60/18723 | 3.71E-05 | 0.001791 | 0.001476 | IRAK2/CD180/CD14/LY96/IL1B/PLCG2/TRIB1/LILRA2/LTF/TLR2/BMP6/CCL5/LBP/TLR4 | 14 |
| BP | GO:0034113 | heterotypic cell-cell adhesion | 14/1271 | 61/18723 | 4.52E-05 | 0.002016 | 0.001662 | ITGAX/SKAP1/ITGA4/CD200/IL1B/LILRB2/WNK1/DSP/JUP/DSC2/KLF4/CXADR/PTPRC/PERP | 14 |
| BP | GO:0043030 | regulation of macrophage activation | 14/1271 | 61/18723 | 4.52E-05 | 0.002016 | 0.001662 | SPACA3/STAP1/TREM2/CST7/IL31RA/LRFN5/CD200/TLR6/IL6/CD74/LBP/PTPRC/VSIG4/TLR4 | 14 |
| BP | GO:0048247 | lymphocyte chemotaxis | 14/1271 | 64/18723 | 7.94E-05 | 0.002836 | 0.002337 | STK39/CCL18/PIK3CD/PTK2B/ADAM8/GPR183/WNK1/CCL13/CXCL13/C10orf99/PIK3CG/SAA1/CCL5/CCL11 | 14 |
| BP | GO:0003208 | cardiac ventricle morphogenesis | 14/1271 | 71/18723 | 0.000256 | 0.006925 | 0.005707 | NPY2R/MESP1/PPP1R13L/FGFR2/DSP/FOXH1/JAG1/GSK3A/SOX11/TGFBR3/GRHL2/LRP2/MYL3/SFRP2 | 14 |
| BP | GO:0032722 | positive regulation of chemokine production | 14/1271 | 71/18723 | 0.000256 | 0.006925 | 0.005707 | F2RL1/IL17F/FFAR2/AIF1/IL1B/IL17A/AIRE/TLR2/TLR9/IL6/CD74/TLR3/LBP/TLR4 | 14 |
| BP | GO:0050795 | regulation of behavior | 14/1271 | 71/18723 | 0.000256 | 0.006925 | 0.005707 | CCK/NPY2R/HOXA1/STRA6/MC4R/KCNA2/PENK/ADA/GJA1/ADRB1/NMU/RETN/LEPR/PTGDS | 14 |
| BP | GO:0032729 | positive regulation of interferon-gamma production | 14/1271 | 72/18723 | 0.000298 | 0.00757 | 0.006239 | SLAMF1/LILRB1/F2RL1/CD226/SLC7A5/CD14/FZD5/IL1B/PDE4B/BTN3A1/IL21/TLR9/TLR3/TLR4 | 14 |
| BP | GO:0032732 | positive regulation of interleukin-1 production | 14/1271 | 73/18723 | 0.000346 | 0.008344 | 0.006877 | F2RL1/NLRC4/AIM2/LILRA5/FZD5/TYROBP/MNDA/IL17A/PANX2/LILRA2/SAA1/TLR6/IL6/TLR4 | 14 |
| BP | GO:0033077 | T cell differentiation in thymus | 14/1271 | 75/18723 | 0.000462 | 0.010035 | 0.008271 | CD3D/PSMB11/FZD5/ADAM8/CD3G/IL1B/AIRE/IL7R/BMP4/ADA/CLPTM1/TOX/CD74/PTPRC | 14 |
| BP | GO:0001892 | embryonic placenta development | 14/1271 | 82/18723 | 0.001163 | 0.01957 | 0.01613 | DNAJB6/KRT8/PLCD3/FZD5/SOX15/BMP5/FGFR2/EOMES/GJB5/HES1/PRDM1/GRHL2/E2F8/RSPO3 | 14 |
| BP | GO:0001910 | regulation of leukocyte mediated cytotoxicity | 14/1271 | 82/18723 | 0.001163 | 0.01957 | 0.01613 | STAP1/CXCL6/LILRB1/F2RL1/CD226/VAV1/ARRB2/TYROBP/IL7R/NCKAP1L/IL21/CD1E/PTPRC/SERPINB4 | 14 |
| BP | GO:0043367 | CD4-positive, alpha-beta T cell differentiation | 14/1271 | 83/18723 | 0.001314 | 0.021708 | 0.017892 | IRF4/BATF/CD86/CD80/GPR183/PLA2G2D/RORC/HLX/NCKAP1L/IL21/CTSL/IL6/TOX/RARA | 14 |
| BP | GO:0016101 | diterpenoid metabolic process | 14/1271 | 87/18723 | 0.002087 | 0.029774 | 0.024539 | BCO1/ADH7/CYP2W1/CYP3A7/ALDH3A2/STAR/CYP3A5/CYP2C18/RDH12/ADH1A/PNLIP/AKR1B10/CRABP2/LRP2 | 14 |
| BP | GO:0034109 | homotypic cell-cell adhesion | 14/1271 | 90/18723 | 0.002887 | 0.037613 | 0.031 | MPL/PLEK/TNFSF11/DSP/PIK3CG/COMP/IL6/JUP/DSC2/CCL5/RAP2B/CXADR/CD9/F11R | 14 |
| BP | GO:0002065 | columnar/cuboidal epithelial cell differentiation | 14/1271 | 91/18723 | 0.003204 | 0.039869 | 0.032859 | B9D1/IL31RA/BMP5/FGFR2/BMP4/GSK3A/SOX11/KLF5/HES1/PRDM1/BMP6/RARA/TP63/NPY | 14 |
| BP | GO:0030316 | osteoclast differentiation | 14/1271 | 94/18723 | 0.004328 | 0.049878 | 0.041109 | TREM2/LILRB1/TCIRG1/LILRB3/TYROBP/RASSF2/GPR183/TNFSF11/OCSTAMP/IL17A/LTF/GPR137/TLR3/TLR4 | 14 |
| BP | GO:0002718 | regulation of cytokine production involved in immune response | 14/1271 | 96/18723 | 0.005241 | 0.053533 | 0.044121 | SLAMF1/LILRB1/F2RL1/CD226/SLC7A5/FFAR2/FZD5/IL1B/PLCG2/SEMA7A/IL6/CD74/TLR3/TLR4 | 14 |
| BP | GO:0006968 | cellular defense response | 13/1271 | 54/18723 | 4.93E-05 | 0.002115 | 0.001743 | TCIRG1/CCR3/PTK2B/LY96/TYROBP/PRF1/LILRB2/MNDA/FCMR/ITK/NCF2/GNLY/LBP | 13 |
| BP | GO:2000401 | regulation of lymphocyte migration | 13/1271 | 61/18723 | 0.000187 | 0.005373 | 0.004429 | STK39/ITGA4/PTK2B/ADAM8/AIF1/MIA3/CD200/WNK1/AIRE/CXCL13/C10orf99/CCL5/CXCL12 | 13 |
| BP | GO:0022617 | extracellular matrix disassembly | 13/1271 | 63/18723 | 0.000263 | 0.006957 | 0.005734 | MMP3/MMP1/CTSS/MMP10/ADAM8/FGFR4/MMP9/IL6/KLK7/MMP12/MMP13/MMP7/ADAMTS4 | 13 |
| BP | GO:0002720 | positive regulation of cytokine production involved in immune response | 13/1271 | 65/18723 | 0.000364 | 0.008582 | 0.007073 | SLAMF1/LILRB1/F2RL1/CD226/SLC7A5/FFAR2/FZD5/IL1B/PLCG2/SEMA7A/IL6/CD74/TLR4 | 13 |
| BP | GO:0045682 | regulation of epidermis development | 13/1271 | 65/18723 | 0.000364 | 0.008582 | 0.007073 | KRT10/KRT2/HES5/KDF1/BMP4/HES1/GRHL2/ZBED2/ESRP1/CYP27B1/TP63/SFRP4/AQP3 | 13 |
| BP | GO:0071677 | positive regulation of mononuclear cell migration | 13/1271 | 65/18723 | 0.000364 | 0.008582 | 0.007073 | STK39/SLAMF1/ITGA4/PTK2B/ADAM8/AIF1/WNK1/PLA2G7/CXCL13/FPR2/CCL5/S100A14/CXCL12 | 13 |
| BP | GO:0031640 | killing of cells of other organism | 13/1271 | 68/18723 | 0.000575 | 0.01196 | 0.009857 | CXCL6/F2RL1/PRF1/TREM1/LTF/BCL2L1/CCL13/GNLY/CAMP/DCD/LCE3B/LYZ/LCE3A | 13 |
| BP | GO:0003151 | outflow tract morphogenesis | 13/1271 | 74/18723 | 0.001314 | 0.021708 | 0.017892 | TBX1/NPY2R/ROBO2/FGFR2/RYR1/BMP4/FOXH1/JAG1/SOX11/HES1/TGFBR3/LRP2/SFRP2 | 13 |
| BP | GO:0002292 | T cell differentiation involved in immune response | 13/1271 | 75/18723 | 0.001493 | 0.023451 | 0.019328 | IRF4/BATF/CD86/CD80/GPR183/RORC/HLX/EOMES/PCK1/IL21/IL6/RARA/FCER1G | 13 |
| BP | GO:0014068 | positive regulation of phosphatidylinositol 3-kinase signaling | 13/1271 | 79/18723 | 0.002421 | 0.03288 | 0.027099 | TREM2/EGF/F2RL1/HCLS1/FLT3/PLXNB1/MYDGF/SELP/CBL/FGR/WNT16/AGT/CCL5 | 13 |
| BP | GO:0071260 | cellular response to mechanical stimulus | 13/1271 | 81/18723 | 0.003038 | 0.038687 | 0.031885 | CYBA/IL1B/SLC2A1/AGT/GJA1/MAP3K1/BMP6/TLR3/TLR4/MMP7/ANKRD1/F11R/SLC38A2 | 13 |
| BP | GO:0060021 | roof of mouth development | 13/1271 | 85/18723 | 0.004654 | 0.050652 | 0.041747 | TBX1/TBC1D32/DLX5/EPHB3/PLEKHA1/FRAS1/SOX11/FOXE1/TGFBR3/COL11A2/GABRB3/IRF6/ITGB8 | 13 |
| BP | GO:2000117 | negative regulation of cysteine-type endopeptidase activity | 13/1271 | 86/18723 | 0.005151 | 0.053212 | 0.043856 | DPEP1/CST7/DNAJB6/CRYAB/ARRB2/SIAH2/HERPUD1/BIRC3/LTF/MMP9/KLF4/DHCR24/SFRP2 | 13 |
| BP | GO:0010232 | vascular transport | 13/1271 | 88/18723 | 0.006272 | 0.059874 | 0.049347 | SLC7A5/KCNJ8/SLC2A1/SLC7A8/SLC15A2/GJA1/SLC7A2/SLCO2B1/SLC8A2/LRP2/SLC5A5/LEPR/SLC38A2 | 13 |
| BP | GO:0002673 | regulation of acute inflammatory response | 12/1271 | 48/18723 | 6.42E-05 | 0.0025 | 0.002061 | F12/FFAR2/ADAM8/C3/TNFSF11/PLA2G2D/IL1B/ALOX5AP/PIK3CG/PTGES/IL6/IL20RB | 12 |
| BP | GO:0045104 | intermediate filament cytoskeleton organization | 12/1271 | 51/18723 | 0.000121 | 0.003905 | 0.003219 | KRT3/NEFL/DNAJB6/NEFM/BFSP2/KRT2/KRT18/DSP/PKP1/TCHH/PPL/KRT16 | 12 |
| BP | GO:0043277 | apoptotic cell clearance | 12/1271 | 52/18723 | 0.000148 | 0.004535 | 0.003738 | RHOH/TREM2/TXNDC5/FCN1/FCN2/TYROBP/TGM2/FCN3/C3/MERTK/MARCO/RARA | 12 |
| BP | GO:0045103 | intermediate filament-based process | 12/1271 | 52/18723 | 0.000148 | 0.004535 | 0.003738 | KRT3/NEFL/DNAJB6/NEFM/BFSP2/KRT2/KRT18/DSP/PKP1/TCHH/PPL/KRT16 | 12 |
| BP | GO:0002707 | negative regulation of lymphocyte mediated immunity | 12/1271 | 53/18723 | 0.000179 | 0.005285 | 0.004356 | SLAMF1/LILRB1/CR2/CR1L/ARRB2/SUSD4/CR1/IL7R/NCKAP1L/IL20RB/PTPRC/SERPINB4 | 12 |
| BP | GO:0002763 | positive regulation of myeloid leukocyte differentiation | 12/1271 | 58/18723 | 0.000438 | 0.009661 | 0.007963 | TREM2/HCLS1/CTNNBIP1/PLA2G3/TYROBP/TNFSF11/OCSTAMP/IL17A/TRIB1/CD74/ID2/JUN | 12 |
| BP | GO:0032613 | interleukin-10 production | 12/1271 | 62/18723 | 0.000825 | 0.015244 | 0.012564 | TREM2/IRF4/LILRB1/F2RL1/LILRA5/TYROBP/PLCG2/TLR2/TLR9/IL6/IL20RB/TLR4 | 12 |
| BP | GO:0032623 | interleukin-2 production | 12/1271 | 62/18723 | 0.000825 | 0.015244 | 0.012564 | IRF4/CD86/CD80/IL17F/IL1B/PLCG2/CR1/PDE4B/HOMER2/IL20RB/PTPRC/VSIG4 | 12 |
| BP | GO:0032653 | regulation of interleukin-10 production | 12/1271 | 62/18723 | 0.000825 | 0.015244 | 0.012564 | TREM2/IRF4/LILRB1/F2RL1/LILRA5/TYROBP/PLCG2/TLR2/TLR9/IL6/IL20RB/TLR4 | 12 |
| BP | GO:0032663 | regulation of interleukin-2 production | 12/1271 | 62/18723 | 0.000825 | 0.015244 | 0.012564 | IRF4/CD86/CD80/IL17F/IL1B/PLCG2/CR1/PDE4B/HOMER2/IL20RB/PTPRC/VSIG4 | 12 |
| BP | GO:0032731 | positive regulation of interleukin-1 beta production | 12/1271 | 62/18723 | 0.000825 | 0.015244 | 0.012564 | F2RL1/NLRC4/AIM2/LILRA5/FZD5/TYROBP/MNDA/IL17A/LILRA2/TLR6/IL6/TLR4 | 12 |
| BP | GO:0002704 | negative regulation of leukocyte mediated immunity | 12/1271 | 63/18723 | 0.000957 | 0.017051 | 0.014053 | SLAMF1/LILRB1/CR2/CR1L/ARRB2/SUSD4/CR1/IL7R/NCKAP1L/IL20RB/PTPRC/SERPINB4 | 12 |
| BP | GO:0045670 | regulation of osteoclast differentiation | 12/1271 | 64/18723 | 0.001107 | 0.019088 | 0.015732 | TREM2/LILRB1/LILRB3/TYROBP/RASSF2/TNFSF11/OCSTAMP/IL17A/LTF/GPR137/TLR3/TLR4 | 12 |
| BP | GO:0042130 | negative regulation of T cell proliferation | 12/1271 | 67/18723 | 0.001675 | 0.02536 | 0.020901 | CTLA4/LILRB1/CD86/CD80/PLA2G2D/CR1/LILRB2/BMP4/IL2RA/GPNMB/IL20RB/VSIG4 | 12 |
| BP | GO:0045123 | cellular extravasation | 12/1271 | 70/18723 | 0.002464 | 0.03316 | 0.02733 | TRIM55/CHST2/ITGA4/SELL/PIK3CD/ADAM8/SELP/PECAM1/SELPLG/PIK3CG/CXCL12/F11R | 12 |
| BP | GO:0035567 | non-canonical Wnt signaling pathway | 12/1271 | 72/18723 | 0.003141 | 0.03963 | 0.032662 | VANGL2/NKD1/FZD5/TIAM1/CELSR1/CELSR2/FZD10/RSPO3/SFRP4/FZD6/SFRP2/DKK1 | 12 |
| BP | GO:0006801 | superoxide metabolic process | 12/1271 | 74/18723 | 0.003961 | 0.04643 | 0.038267 | CYBA/MAPT/F2RL1/NCF4/TYROBP/PREX1/NQO1/CYBB/AGT/NCF2/FPR2/MPO | 12 |
| BP | GO:0014823 | response to activity | 12/1271 | 76/18723 | 0.004945 | 0.051832 | 0.042719 | CYBA/OXCT1/COL4A2/CXCR4/STAR/CBL/PTN/PCK1/TNS2/AGT/IL6/BMP6 | 12 |
| BP | GO:0045730 | respiratory burst | 11/1271 | 37/18723 | 2.23E-05 | 0.001201 | 0.00099 | CYBA/TREM2/JCHAIN/NCF4/PIK3CD/CD52/CYBB/PIK3CG/NCF2/LBP/MPO | 11 |
| BP | GO:2000404 | regulation of T cell migration | 11/1271 | 42/18723 | 8.16E-05 | 0.002888 | 0.00238 | STK39/ITGA4/ADAM8/AIF1/CD200/WNK1/AIRE/CXCL13/C10orf99/CCL5/CXCL12 | 11 |
| BP | GO:0034142 | toll-like receptor 4 signaling pathway | 11/1271 | 43/18723 | 0.000103 | 0.003502 | 0.002886 | TREM2/F2RL1/CD14/RAB11FIP2/LY96/LILRA2/LTF/TNIP3/S100A14/LBP/TLR4 | 11 |
| BP | GO:0062208 | positive regulation of pattern recognition receptor signaling pathway | 11/1271 | 44/18723 | 0.000129 | 0.004063 | 0.003349 | CYBA/F2RL1/HSPA1B/TLR1/LTF/TLR2/TLR9/TLR3/HSPA1A/LBP/TLR4 | 11 |
| BP | GO:2000107 | negative regulation of leukocyte apoptotic process | 11/1271 | 46/18723 | 0.000198 | 0.005647 | 0.004655 | LILRB1/HCLS1/MERTK/IL7R/BMP4/ADA/EFNA1/CCL5/CD74/AURKB/CXCL12 | 11 |
| BP | GO:0048512 | circadian behavior | 11/1271 | 47/18723 | 0.000243 | 0.006681 | 0.005507 | NPY2R/C3orf70/CIART/STAR/KCNA2/ADA/NR1D2/ADRB1/NMU/ID2/PTGDS | 11 |
| BP | GO:0097028 | dendritic cell differentiation | 11/1271 | 47/18723 | 0.000243 | 0.006681 | 0.005507 | TREM2/IRF4/LILRB1/BATF/F2RL1/TMEM176B/FLT3/TMEM176A/LILRB2/ITGB8/BATF2 | 11 |
| BP | GO:0007622 | rhythmic behavior | 11/1271 | 49/18723 | 0.000358 | 0.008565 | 0.007059 | NPY2R/C3orf70/CIART/STAR/KCNA2/ADA/NR1D2/ADRB1/NMU/ID2/PTGDS | 11 |
| BP | GO:0006636 | unsaturated fatty acid biosynthetic process | 11/1271 | 51/18723 | 0.000517 | 0.011048 | 0.009106 | ELOVL4/PLA2G3/IL1B/ELOVL6/TBXAS1/DEGS1/PTGES/ELOVL2/CD74/PTGDS/EDN2 | 11 |
| BP | GO:0002218 | activation of innate immune response | 11/1271 | 52/18723 | 0.000616 | 0.012506 | 0.010307 | ZBP1/NLRC4/FCN1/AIM2/FFAR2/TYROBP/PLCG2/PAK3/MNDA/LILRA2/TLR4 | 11 |
| BP | GO:1905517 | macrophage migration | 11/1271 | 55/18723 | 0.001012 | 0.017909 | 0.014761 | STAP1/SLAMF1/TREM2/TRIM55/PTK2B/CD200/SAA1/C3AR1/CCL5/CD9/EDN2 | 11 |
| BP | GO:1904645 | response to amyloid-beta | 11/1271 | 56/18723 | 0.001183 | 0.019847 | 0.016358 | TREM2/MMP3/ITGA4/ADRB2/GJA1/MMP9/TLR6/FPR2/MMP12/MMP13/TLR4 | 11 |
| BP | GO:0045604 | regulation of epidermal cell differentiation | 11/1271 | 58/18723 | 0.001598 | 0.024883 | 0.020509 | HES5/KDF1/BMP4/HES1/GRHL2/ZBED2/ESRP1/CYP27B1/TP63/SFRP4/AQP3 | 11 |
| BP | GO:0002820 | negative regulation of adaptive immune response | 11/1271 | 59/18723 | 0.001846 | 0.026903 | 0.022173 | SLAMF1/LILRB1/CR2/SAMSN1/CR1L/SUSD4/CR1/IL7R/NCKAP1L/IL20RB/PTPRC | 11 |
| BP | GO:0032615 | interleukin-12 production | 11/1271 | 62/18723 | 0.002783 | 0.036822 | 0.030348 | SLAMF1/LILRB1/ARRB2/LILRA5/PLCG2/LTB/IL17A/TLR2/TLR9/TLR3/TLR4 | 11 |
| BP | GO:0032655 | regulation of interleukin-12 production | 11/1271 | 62/18723 | 0.002783 | 0.036822 | 0.030348 | SLAMF1/LILRB1/ARRB2/LILRA5/PLCG2/LTB/IL17A/TLR2/TLR9/TLR3/TLR4 | 11 |
| BP | GO:0061077 | chaperone-mediated protein folding | 11/1271 | 67/18723 | 0.005165 | 0.053212 | 0.043856 | PDIA4/DNAJB6/HSPA1L/HSPA13/HSPA1B/HSPA2/SDF2L1/FKBP2/DNAJB1/CD74/HSPA1A | 11 |
| BP | GO:0042531 | positive regulation of tyrosine phosphorylation of STAT protein | 11/1271 | 68/18723 | 0.005793 | 0.056802 | 0.046815 | IL24/IL31RA/HCLS1/GHR/HES5/FLT3/HES1/IL21/CRLF1/IL6/CCL5 | 11 |
| BP | GO:0002335 | mature B cell differentiation | 10/1271 | 33/18723 | 4.39E-05 | 0.002009 | 0.001656 | XBP1/CD19/POU2AF1/PTK2B/GPR183/PLCG2/CR1/ADA/IL21/IL6 | 10 |
| BP | GO:0150077 | regulation of neuroinflammatory response | 10/1271 | 40/18723 | 0.00026 | 0.006925 | 0.005707 | STAP1/TREM2/MMP3/CST7/CD200/IL1B/PLCG2/MMP9/IL6/PTPRC | 10 |
| BP | GO:0032733 | positive regulation of interleukin-10 production | 10/1271 | 41/18723 | 0.000323 | 0.008074 | 0.006655 | TREM2/IRF4/F2RL1/LILRA5/PLCG2/TLR2/TLR9/IL6/IL20RB/TLR4 | 10 |
| BP | GO:0030574 | collagen catabolic process | 10/1271 | 42/18723 | 0.000398 | 0.009121 | 0.007517 | MMP3/MMP1/CTSS/MMP27/MMP10/CTSL/MMP9/MMP12/MMP13/MMP7 | 10 |
| BP | GO:0030890 | positive regulation of B cell proliferation | 10/1271 | 42/18723 | 0.000398 | 0.009121 | 0.007517 | CD38/GPR183/ADA/NCKAP1L/IL21/TLR9/CD74/PTPRC/TLR4/TNFRSF4 | 10 |
| BP | GO:0150076 | neuroinflammatory response | 10/1271 | 44/18723 | 0.000593 | 0.012252 | 0.010098 | STAP1/TREM2/MMP3/CST7/CD200/IL1B/PLCG2/MMP9/IL6/PTPRC | 10 |
| BP | GO:0048546 | digestive tract morphogenesis | 10/1271 | 45/18723 | 0.000717 | 0.013961 | 0.011506 | EPHB3/HLX/STRA6/FGFR2/BMP4/SOX11/SOX10/TP63/SFRP2/ID2 | 10 |
| BP | GO:0048483 | autonomic nervous system development | 10/1271 | 47/18723 | 0.001029 | 0.018144 | 0.014954 | ARX/TBX1/HLX/RHOXF1/SOX11/HES1/HOXB2/SOX10/RET/TP63 | 10 |
| BP | GO:0006692 | prostanoid metabolic process | 10/1271 | 49/18723 | 0.001443 | 0.022795 | 0.018787 | CYP4F8/PLA2G3/IL1B/GSTA1/TBXAS1/PTGR1/PTGES/CD74/PTGDS/EDN2 | 10 |
| BP | GO:0006693 | prostaglandin metabolic process | 10/1271 | 49/18723 | 0.001443 | 0.022795 | 0.018787 | CYP4F8/PLA2G3/IL1B/GSTA1/TBXAS1/PTGR1/PTGES/CD74/PTGDS/EDN2 | 10 |
| BP | GO:1904894 | positive regulation of receptor signaling pathway via STAT | 10/1271 | 49/18723 | 0.001443 | 0.022795 | 0.018787 | IL26/GHR/HES5/PTK2B/IL7R/AGT/PRLR/HES1/IL6/CCL5 | 10 |
| BP | GO:0045058 | T cell selection | 10/1271 | 50/18723 | 0.001696 | 0.025477 | 0.020998 | IRF4/BATF/CD3D/CD3G/AIRE/CTSL/IL6/TOX/CD74/PTPRC | 10 |
| BP | GO:0014009 | glial cell proliferation | 10/1271 | 51/18723 | 0.001983 | 0.028593 | 0.023566 | TREM2/PTK2B/IL1B/PTN/PENK/SOX11/HES1/SOX10/IL6/LEPR | 10 |
| BP | GO:0002823 | negative regulation of adaptive immune response based on somatic recombination of immune receptors built from immunoglobulin superfamily domains | 10/1271 | 54/18723 | 0.003088 | 0.039053 | 0.032187 | SLAMF1/LILRB1/CR2/CR1L/SUSD4/CR1/IL7R/NCKAP1L/IL20RB/PTPRC | 10 |
| BP | GO:0031529 | ruffle organization | 10/1271 | 56/18723 | 0.004063 | 0.047027 | 0.038759 | ARHGEF26/STAP1/TCIRG1/AIF1/PLEK/ARFIP2/PLEKHA1/CORO1B/EPS8L1/SH3YL1 | 10 |
| BP | GO:0046456 | icosanoid biosynthetic process | 10/1271 | 56/18723 | 0.004063 | 0.047027 | 0.038759 | PLA2G3/IL1B/ALOX5AP/TBXAS1/LTC4S/PTGES/GGT7/CD74/PTGDS/EDN2 | 10 |
| BP | GO:0031424 | keratinization | 10/1271 | 58/18723 | 0.005268 | 0.053533 | 0.044121 | SHARPIN/KRT2/LIPK/TCHH/LCE3B/PPL/KRT16/LCE3D/LCE3E/LCE3A | 10 |
| BP | GO:0043506 | regulation of JUN kinase activity | 10/1271 | 58/18723 | 0.005268 | 0.053533 | 0.044121 | FZD5/PTK2B/ERN1/TIAM1/ARHGEF5/TLR9/FZD10/TLR6/SFRP2/DKK1 | 10 |
| BP | GO:0002922 | positive regulation of humoral immune response | 9/1271 | 24/18723 | 1.53E-05 | 0.000869 | 0.000716 | TREM2/IL17F/C3/IL1B/CR1/IL17A/PGC/KLK7/PTPRC | 9 |
| BP | GO:0060706 | cell differentiation involved in embryonic placenta development | 9/1271 | 25/18723 | 2.25E-05 | 0.001201 | 0.00099 | DNAJB6/KRT8/FZD5/SOX15/EOMES/GJB5/PRDM1/GRHL2/E2F8 | 9 |
| BP | GO:0002313 | mature B cell differentiation involved in immune response | 9/1271 | 28/18723 | 6.31E-05 | 0.002475 | 0.00204 | XBP1/POU2AF1/PTK2B/GPR183/PLCG2/CR1/ADA/IL21/IL6 | 9 |
| BP | GO:0003382 | epithelial cell morphogenesis | 9/1271 | 33/18723 | 0.000259 | 0.006925 | 0.005707 | ARHGEF26/VSIG1/POF1B/CLIC4/TNMD/COL15A1/GRHL2/CLDN3/RAB25 | 9 |
| BP | GO:2000403 | positive regulation of lymphocyte migration | 9/1271 | 35/18723 | 0.000419 | 0.00944 | 0.00778 | STK39/ITGA4/PTK2B/ADAM8/AIF1/WNK1/CXCL13/CCL5/CXCL12 | 9 |
| BP | GO:0030224 | monocyte differentiation | 9/1271 | 36/18723 | 0.000525 | 0.011145 | 0.009186 | IL31RA/CTNNBIP1/MYC/PIR/BMP4/PPARG/MT1G/CD74/JUN | 9 |
| BP | GO:0043368 | positive T cell selection | 9/1271 | 37/18723 | 0.000653 | 0.012991 | 0.010707 | IRF4/BATF/CD3D/CD3G/CTSL/IL6/TOX/CD74/PTPRC | 9 |
| BP | GO:0051084 | 'de novo' posttranslational protein folding | 9/1271 | 39/18723 | 0.000984 | 0.017471 | 0.0144 | HSPA1L/HSPA13/HSPA1B/HSPA2/UGGT1/SDF2L1/DNAJB1/CD74/HSPA1A | 9 |
| BP | GO:0043507 | positive regulation of JUN kinase activity | 9/1271 | 42/18723 | 0.001723 | 0.025664 | 0.021152 | FZD5/PTK2B/ERN1/TIAM1/ARHGEF5/TLR9/FZD10/TLR6/DKK1 | 9 |
| BP | GO:0006458 | 'de novo' protein folding | 9/1271 | 43/18723 | 0.00205 | 0.029333 | 0.024175 | HSPA1L/HSPA13/HSPA1B/HSPA2/UGGT1/SDF2L1/DNAJB1/CD74/HSPA1A | 9 |
| BP | GO:0042554 | superoxide anion generation | 9/1271 | 44/18723 | 0.002425 | 0.03288 | 0.027099 | CYBA/MAPT/F2RL1/NCF4/TYROBP/CYBB/AGT/NCF2/FPR2 | 9 |
| BP | GO:0046427 | positive regulation of receptor signaling pathway via JAK-STAT | 9/1271 | 44/18723 | 0.002425 | 0.03288 | 0.027099 | IL26/GHR/HES5/PTK2B/AGT/PRLR/HES1/IL6/CCL5 | 9 |
| BP | GO:0060711 | labyrinthine layer development | 9/1271 | 44/18723 | 0.002425 | 0.03288 | 0.027099 | DNAJB6/PLCD3/FZD5/BMP5/FGFR2/GJB5/HES1/GRHL2/RSPO3 | 9 |
| BP | GO:0001754 | eye photoreceptor cell differentiation | 9/1271 | 47/18723 | 0.003882 | 0.045708 | 0.037672 | THRB/NKD1/DIO3/ROM1/PTN/NR2E3/RPGRIP1/PRDM1/TULP1 | 9 |
| BP | GO:0031641 | regulation of myelination | 9/1271 | 47/18723 | 0.003882 | 0.045708 | 0.037672 | ITGAX/CST7/HES5/TG/PARD3/PTN/NCMAP/SOX10/RARA | 9 |
| BP | GO:0045747 | positive regulation of Notch signaling pathway | 9/1271 | 47/18723 | 0.003882 | 0.045708 | 0.037672 | ROBO2/MESP1/HES5/ZMIZ1/TSPAN5/JAG1/HES1/TP63/CNTN6 | 9 |
| BP | GO:0001913 | T cell mediated cytotoxicity | 9/1271 | 49/18723 | 0.005184 | 0.053212 | 0.043856 | LILRB1/PRF1/IL7R/CTSH/NCKAP1L/CD1E/EMP2/PTPRC/GZMM | 9 |
| BP | GO:0002762 | negative regulation of myeloid leukocyte differentiation | 9/1271 | 49/18723 | 0.005184 | 0.053212 | 0.043856 | LILRB1/LILRB3/MYC/TRIB1/LTF/GPR137/RARA/TLR3/TLR4 | 9 |
| BP | GO:0050832 | defense response to fungus | 9/1271 | 49/18723 | 0.005184 | 0.053212 | 0.043856 | CLEC4D/PLCG2/IL17A/LTF/CLEC4E/C10orf99/GNLY/DCD/MPO | 9 |
| BP | GO:0002639 | positive regulation of immunoglobulin production | 9/1271 | 50/18723 | 0.005949 | 0.057397 | 0.047305 | XBP1/MZB1/CD86/DNAJB9/IL21/TLR9/IL6/PTPRC/TNFRSF4 | 9 |
| BP | GO:0042572 | retinol metabolic process | 9/1271 | 50/18723 | 0.005949 | 0.057397 | 0.047305 | BCO1/ADH7/CYP3A7/CYP3A5/CYP2C18/RDH12/ADH1A/PNLIP/AKR1B10 | 9 |
| BP | GO:0030449 | regulation of complement activation | 8/1271 | 21/18723 | 4.04E-05 | 0.001914 | 0.001578 | TREM2/CR2/CR1L/SUSD4/C3/IL1B/CR1/VSIG4 | 8 |
| BP | GO:0033630 | positive regulation of cell adhesion mediated by integrin | 8/1271 | 21/18723 | 4.04E-05 | 0.001914 | 0.001578 | SKAP1/PODXL/CXCL13/NCKAP1L/RET/CCL5/FERMT1/SFRP2 | 8 |
| BP | GO:0045109 | intermediate filament organization | 8/1271 | 25/18723 | 0.000168 | 0.00498 | 0.004104 | NEFL/DNAJB6/NEFM/BFSP2/KRT2/DSP/PKP1/TCHH | 8 |
| BP | GO:0098581 | detection of external biotic stimulus | 8/1271 | 25/18723 | 0.000168 | 0.00498 | 0.004104 | TREM2/NLRC4/LY96/TLR1/TLR2/TLR6/LBP/TLR4 | 8 |
| BP | GO:0002675 | positive regulation of acute inflammatory response | 8/1271 | 28/18723 | 0.000402 | 0.009121 | 0.007517 | FFAR2/ADAM8/C3/TNFSF11/IL1B/ALOX5AP/PIK3CG/IL6 | 8 |
| BP | GO:0034123 | positive regulation of toll-like receptor signaling pathway | 8/1271 | 28/18723 | 0.000402 | 0.009121 | 0.007517 | CYBA/F2RL1/TLR1/LTF/TLR2/TLR9/TLR3/LBP | 8 |
| BP | GO:2000406 | positive regulation of T cell migration | 8/1271 | 29/18723 | 0.000523 | 0.011133 | 0.009176 | STK39/ITGA4/ADAM8/AIF1/WNK1/CXCL13/CCL5/CXCL12 | 8 |
| BP | GO:0060795 | cell fate commitment involved in formation of primary germ layer | 8/1271 | 31/18723 | 0.000851 | 0.01563 | 0.012882 | MESP1/SOX2/EOMES/BMP4/KLF4/SFRP2/DKK1/ETS2 | 8 |
| BP | GO:0048333 | mesodermal cell differentiation | 8/1271 | 33/18723 | 0.001327 | 0.021854 | 0.018011 | MESP1/FGFR2/BMP4/ITGA8/GJA1/KLF4/SFRP2/DKK1 | 8 |
| BP | GO:0050869 | negative regulation of B cell activation | 8/1271 | 34/18723 | 0.001633 | 0.024883 | 0.020509 | CTLA4/SAMSN1/BANK1/TYROBP/CR1/MNDA/ATM/ID2 | 8 |
| BP | GO:0051085 | chaperone cofactor-dependent protein refolding | 8/1271 | 34/18723 | 0.001633 | 0.024883 | 0.020509 | HSPA1L/HSPA13/HSPA1B/HSPA2/SDF2L1/DNAJB1/CD74/HSPA1A | 8 |
| BP | GO:0051482 | positive regulation of cytosolic calcium ion concentration involved in phospholipase C-activating G protein-coupled signaling pathway | 8/1271 | 34/18723 | 0.001633 | 0.024883 | 0.020509 | GPR18/F2RL1/P2RY10/GPR65/TGM2/GPR174/LPAR6/C3AR1 | 8 |
| BP | GO:0055094 | response to lipoprotein particle | 8/1271 | 34/18723 | 0.001633 | 0.024883 | 0.020509 | TREM2/MIA3/PPARG/TLR6/HMGCS1/FCER1G/TLR4/CD9 | 8 |
| BP | GO:0018149 | peptide cross-linking | 8/1271 | 35/18723 | 0.001993 | 0.028593 | 0.023566 | KRT1/KRT10/KRT2/TGM6/TGM2/TGM7/DSP/PI3 | 8 |
| BP | GO:0035633 | maintenance of blood-brain barrier | 8/1271 | 35/18723 | 0.001993 | 0.028593 | 0.023566 | WNK3/OCLN/PECAM1/GJA1/CLDN3/IL6/GJB6/F11R | 8 |
| BP | GO:0010092 | specification of animal organ identity | 8/1271 | 36/18723 | 0.002413 | 0.03288 | 0.027099 | ROBO2/MESP1/FGFR2/BMP4/FOXH1/WNT2B/LRP2/DKK1 | 8 |
| BP | GO:0071402 | cellular response to lipoprotein particle stimulus | 8/1271 | 36/18723 | 0.002413 | 0.03288 | 0.027099 | TREM2/MIA3/PPARG/TLR6/HMGCS1/FCER1G/TLR4/CD9 | 8 |
| BP | GO:0031646 | positive regulation of nervous system process | 8/1271 | 37/18723 | 0.002898 | 0.037613 | 0.031 | CCK/ITGAX/CST7/UNC13B/PARD3/NCMAP/NMU/SOX10 | 8 |
| BP | GO:1905332 | positive regulation of morphogenesis of an epithelium | 8/1271 | 37/18723 | 0.002898 | 0.037613 | 0.031 | ITGAX/EGF/PIK3CD/LGR4/BMP4/WNT2B/AGT/GJA1 | 8 |
| BP | GO:0009595 | detection of biotic stimulus | 8/1271 | 38/18723 | 0.003456 | 0.042148 | 0.034738 | TREM2/NLRC4/LY96/TLR1/TLR2/TLR6/LBP/TLR4 | 8 |
| BP | GO:0045823 | positive regulation of heart contraction | 8/1271 | 39/18723 | 0.004094 | 0.047284 | 0.038971 | ACE2/ADA/GSK3A/ADRB1/NMU/RGS2/APLN/EDN2 | 8 |
| BP | GO:0034122 | negative regulation of toll-like receptor signaling pathway | 8/1271 | 40/18723 | 0.004819 | 0.051309 | 0.042288 | TREM2/IRF4/F2RL1/ARRB2/LGR4/LILRA2/TLR9/TLR6 | 8 |
| BP | GO:1903524 | positive regulation of blood circulation | 8/1271 | 41/18723 | 0.005639 | 0.056191 | 0.046312 | ACE2/ADA/GSK3A/ADRB1/NMU/RGS2/APLN/EDN2 | 8 |
| BP | GO:1905521 | regulation of macrophage migration | 8/1271 | 41/18723 | 0.005639 | 0.056191 | 0.046312 | STAP1/SLAMF1/TREM2/PTK2B/CD200/C3AR1/CCL5/CD9 | 8 |
| BP | GO:0032490 | detection of molecule of bacterial origin | 7/1271 | 12/18723 | 3.82E-06 | 0.000285 | 0.000235 | TREM2/LY96/TLR1/TLR2/TLR6/LBP/TLR4 | 7 |
| BP | GO:0033631 | cell-cell adhesion mediated by integrin | 7/1271 | 16/18723 | 4.34E-05 | 0.002003 | 0.00165 | SKAP1/ITGA4/WNK1/PODXL/CXCL13/ADA/CCL5 | 7 |
| BP | GO:0032604 | granulocyte macrophage colony-stimulating factor production | 7/1271 | 17/18723 | 6.94E-05 | 0.002645 | 0.00218 | CD80/IL17F/IL1B/LILRA2/TLR9/IL17D/PAEP | 7 |
| BP | GO:0032645 | regulation of granulocyte macrophage colony-stimulating factor production | 7/1271 | 17/18723 | 6.94E-05 | 0.002645 | 0.00218 | CD80/IL17F/IL1B/LILRA2/TLR9/IL17D/PAEP | 7 |
| BP | GO:0031649 | heat generation | 7/1271 | 18/18723 | 0.000107 | 0.003591 | 0.00296 | ADRB2/TNFSF11/IL1B/ADRB1/PTGES/NMU/APLN | 7 |
| BP | GO:0050802 | circadian sleep/wake cycle, sleep | 7/1271 | 21/18723 | 0.000326 | 0.008079 | 0.006659 | NPY2R/STAR/KCNA2/ADA/ADRB1/NMU/PTGDS | 7 |
| BP | GO:0060713 | labyrinthine layer morphogenesis | 7/1271 | 22/18723 | 0.00045 | 0.009835 | 0.008106 | DNAJB6/FZD5/BMP5/FGFR2/GJB5/GRHL2/RSPO3 | 7 |
| BP | GO:0002755 | MyD88-dependent toll-like receptor signaling pathway | 7/1271 | 24/18723 | 0.000811 | 0.01524 | 0.012561 | IRAK2/TLR10/TLR1/TLR2/TLR9/TLR6/TLR4 | 7 |
| BP | GO:0022410 | circadian sleep/wake cycle process | 7/1271 | 24/18723 | 0.000811 | 0.01524 | 0.012561 | NPY2R/STAR/KCNA2/ADA/ADRB1/NMU/PTGDS | 7 |
| BP | GO:0070841 | inclusion body assembly | 7/1271 | 24/18723 | 0.000811 | 0.01524 | 0.012561 | MAPT/DNAJB6/HSPA1B/DNAJA4/HSPA2/DNAJB1/HSPA1A | 7 |
| BP | GO:0032753 | positive regulation of interleukin-4 production | 7/1271 | 25/18723 | 0.001061 | 0.018473 | 0.015225 | IRF4/CD86/SLC7A5/CLECL1/RARA/FCER1G/IL20RB | 7 |
| BP | GO:0033622 | integrin activation | 7/1271 | 25/18723 | 0.001061 | 0.018473 | 0.015225 | MZB1/SKAP1/PLEK/SELP/CXCL13/FERMT1/CXCL12 | 7 |
| BP | GO:0060669 | embryonic placenta morphogenesis | 7/1271 | 26/18723 | 0.001367 | 0.022052 | 0.018175 | DNAJB6/FZD5/BMP5/FGFR2/GJB5/GRHL2/RSPO3 | 7 |
| BP | GO:1900017 | positive regulation of cytokine production involved in inflammatory response | 7/1271 | 26/18723 | 0.001367 | 0.022052 | 0.018175 | IL17F/PLA2G3/IL17A/TLR6/IL6/IL17D/TLR4 | 7 |
| BP | GO:0036037 | CD8-positive, alpha-beta T cell activation | 7/1271 | 27/18723 | 0.001739 | 0.025742 | 0.021216 | LILRB1/GPR18/PSMB11/EOMES/TNFSF8/NCKAP1L/TOX | 7 |
| BP | GO:0042745 | circadian sleep/wake cycle | 7/1271 | 27/18723 | 0.001739 | 0.025742 | 0.021216 | NPY2R/STAR/KCNA2/ADA/ADRB1/NMU/PTGDS | 7 |
| BP | GO:0010818 | T cell chemotaxis | 7/1271 | 28/18723 | 0.002185 | 0.030457 | 0.025102 | STK39/PIK3CD/GPR183/WNK1/CXCL13/PIK3CG/CCL5 | 7 |
| BP | GO:0030970 | retrograde protein transport, ER to cytosol | 7/1271 | 29/18723 | 0.002713 | 0.036252 | 0.029878 | EDEM1/ERLEC1/SEL1L/RHBDD1/HERPUD1/UBE2J1/SVIP | 7 |
| BP | GO:0098868 | bone growth | 7/1271 | 29/18723 | 0.002713 | 0.036252 | 0.029878 | NPPC/ANXA6/FGFR2/COMP/RARA/MMP13/LEPR | 7 |
| BP | GO:1903513 | endoplasmic reticulum to cytosol transport | 7/1271 | 29/18723 | 0.002713 | 0.036252 | 0.029878 | EDEM1/ERLEC1/SEL1L/RHBDD1/HERPUD1/UBE2J1/SVIP | 7 |
| BP | GO:0010453 | regulation of cell fate commitment | 7/1271 | 30/18723 | 0.003335 | 0.040852 | 0.03367 | MESP1/BMP4/HES1/ESRP1/SOSTDC1/SFRP2/DKK1 | 7 |
| BP | GO:0070229 | negative regulation of lymphocyte apoptotic process | 7/1271 | 30/18723 | 0.003335 | 0.040852 | 0.03367 | IL7R/BMP4/ADA/EFNA1/CCL5/CD74/AURKB | 7 |
| BP | GO:0001516 | prostaglandin biosynthetic process | 7/1271 | 31/18723 | 0.004059 | 0.047027 | 0.038759 | PLA2G3/IL1B/TBXAS1/PTGES/CD74/PTGDS/EDN2 | 7 |
| BP | GO:0046457 | prostanoid biosynthetic process | 7/1271 | 31/18723 | 0.004059 | 0.047027 | 0.038759 | PLA2G3/IL1B/TBXAS1/PTGES/CD74/PTGDS/EDN2 | 7 |
| BP | GO:0030431 | sleep | 7/1271 | 32/18723 | 0.004897 | 0.051632 | 0.042555 | NPY2R/STAR/KCNA2/ADA/ADRB1/NMU/PTGDS | 7 |
| BP | GO:0045684 | positive regulation of epidermis development | 7/1271 | 32/18723 | 0.004897 | 0.051632 | 0.042555 | KRT10/KRT2/KDF1/BMP4/ZBED2/CYP27B1/SFRP4 | 7 |
| BP | GO:0060914 | heart formation | 7/1271 | 32/18723 | 0.004897 | 0.051632 | 0.042555 | ROBO2/MESP1/BMP4/FOXH1/HES1/LRP2/DKK1 | 7 |
| BP | GO:0032633 | interleukin-4 production | 7/1271 | 33/18723 | 0.005858 | 0.056821 | 0.046831 | IRF4/CD86/SLC7A5/CLECL1/RARA/FCER1G/IL20RB | 7 |
| BP | GO:0032673 | regulation of interleukin-4 production | 7/1271 | 33/18723 | 0.005858 | 0.056821 | 0.046831 | IRF4/CD86/SLC7A5/CLECL1/RARA/FCER1G/IL20RB | 7 |
| BP | GO:0035025 | positive regulation of Rho protein signal transduction | 7/1271 | 33/18723 | 0.005858 | 0.056821 | 0.046831 | GPR18/F2RL1/P2RY10/GPR65/GPR174/LPAR6/F11R | 7 |
| BP | GO:0048730 | epidermis morphogenesis | 7/1271 | 33/18723 | 0.005858 | 0.056821 | 0.046831 | FLG2/KRT27/FGFR2/FOXE1/KLF4/TP63/SOSTDC1 | 7 |
| BP | GO:0033632 | regulation of cell-cell adhesion mediated by integrin | 6/1271 | 11/18723 | 3.32E-05 | 0.001646 | 0.001356 | SKAP1/WNK1/PODXL/CXCL13/ADA/CCL5 | 6 |
| BP | GO:0090084 | negative regulation of inclusion body assembly | 6/1271 | 11/18723 | 3.32E-05 | 0.001646 | 0.001356 | DNAJB6/HSPA1B/DNAJA4/HSPA2/DNAJB1/HSPA1A | 6 |
| BP | GO:0060100 | positive regulation of phagocytosis, engulfment | 6/1271 | 13/18723 | 0.00011 | 0.003598 | 0.002965 | STAP1/TREM2/F2RL1/C3/PLCG2/NCKAP1L | 6 |
| BP | GO:1905155 | positive regulation of membrane invagination | 6/1271 | 13/18723 | 0.00011 | 0.003598 | 0.002965 | STAP1/TREM2/F2RL1/C3/PLCG2/NCKAP1L | 6 |
| BP | GO:0043374 | CD8-positive, alpha-beta T cell differentiation | 6/1271 | 14/18723 | 0.000181 | 0.005296 | 0.004365 | GPR18/PSMB11/EOMES/TNFSF8/NCKAP1L/TOX | 6 |
| BP | GO:0032725 | positive regulation of granulocyte macrophage colony-stimulating factor production | 6/1271 | 15/18723 | 0.000284 | 0.007266 | 0.005989 | CD80/IL1B/LILRA2/TLR9/IL17D/PAEP | 6 |
| BP | GO:0060099 | regulation of phagocytosis, engulfment | 6/1271 | 15/18723 | 0.000284 | 0.007266 | 0.005989 | STAP1/TREM2/F2RL1/C3/PLCG2/NCKAP1L | 6 |
| BP | GO:1905153 | regulation of membrane invagination | 6/1271 | 16/18723 | 0.000428 | 0.009568 | 0.007886 | STAP1/TREM2/F2RL1/C3/PLCG2/NCKAP1L | 6 |
| BP | GO:0036092 | phosphatidylinositol-3-phosphate biosynthetic process | 6/1271 | 17/18723 | 0.000624 | 0.012506 | 0.010307 | PIK3C2G/PIK3CD/SYNJ2/INPP4A/PIK3CG/ATM | 6 |
| BP | GO:0048521 | negative regulation of behavior | 6/1271 | 17/18723 | 0.000624 | 0.012506 | 0.010307 | CCK/NPY2R/MC4R/ADA/NMU/RETN | 6 |
| BP | GO:0090083 | regulation of inclusion body assembly | 6/1271 | 17/18723 | 0.000624 | 0.012506 | 0.010307 | DNAJB6/HSPA1B/DNAJA4/HSPA2/DNAJB1/HSPA1A | 6 |
| BP | GO:0002923 | regulation of humoral immune response mediated by circulating immunoglobulin | 6/1271 | 18/18723 | 0.000883 | 0.015931 | 0.01313 | TREM2/CR2/CR1L/SUSD4/CR1/PTPRC | 6 |
| BP | GO:0003128 | heart field specification | 6/1271 | 18/18723 | 0.000883 | 0.015931 | 0.01313 | ROBO2/MESP1/BMP4/FOXH1/LRP2/DKK1 | 6 |
| BP | GO:0034134 | toll-like receptor 2 signaling pathway | 6/1271 | 18/18723 | 0.000883 | 0.015931 | 0.01313 | CYBA/TREM2/F2RL1/TLR1/TLR2/TLR6 | 6 |
| BP | GO:0045187 | regulation of circadian sleep/wake cycle, sleep | 6/1271 | 18/18723 | 0.000883 | 0.015931 | 0.01313 | NPY2R/KCNA2/ADA/ADRB1/NMU/PTGDS | 6 |
| BP | GO:0002523 | leukocyte migration involved in inflammatory response | 6/1271 | 19/18723 | 0.001216 | 0.020277 | 0.016712 | SLAMF1/TRIM55/FFAR2/ADAM8/PTN/LBP | 6 |
| BP | GO:0043031 | negative regulation of macrophage activation | 6/1271 | 19/18723 | 0.001216 | 0.020277 | 0.016712 | CST7/IL31RA/LRFN5/CD200/PTPRC/VSIG4 | 6 |
| BP | GO:0032930 | positive regulation of superoxide anion generation | 6/1271 | 20/18723 | 0.001639 | 0.024883 | 0.020509 | CYBA/MAPT/F2RL1/TYROBP/AGT/FPR2 | 6 |
| BP | GO:0046629 | gamma-delta T cell activation | 6/1271 | 20/18723 | 0.001639 | 0.024883 | 0.020509 | LILRB1/GPR18/ITK/NCKAP1L/CXADR/PTPRC | 6 |
| BP | GO:0007252 | I-kappaB phosphorylation | 6/1271 | 21/18723 | 0.002164 | 0.030401 | 0.025056 | PRDX4/PLCG2/TLR2/TLR9/TLR3/TLR4 | 6 |
| BP | GO:0070269 | pyroptosis | 6/1271 | 21/18723 | 0.002164 | 0.030401 | 0.025056 | ZBP1/TREM2/NLRC4/AIM2/GZMB/GSDMC | 6 |
| BP | GO:0090026 | positive regulation of monocyte chemotaxis | 6/1271 | 21/18723 | 0.002164 | 0.030401 | 0.025056 | AIF1/PLA2G7/FPR2/CCL5/S100A14/CXCL12 | 6 |
| BP | GO:0002220 | innate immune response activating cell surface receptor signaling pathway | 6/1271 | 22/18723 | 0.002806 | 0.036863 | 0.030382 | FCN1/FFAR2/TYROBP/PLCG2/PAK3/LILRA2 | 6 |
| BP | GO:0042749 | regulation of circadian sleep/wake cycle | 6/1271 | 22/18723 | 0.002806 | 0.036863 | 0.030382 | NPY2R/KCNA2/ADA/ADRB1/NMU/PTGDS | 6 |
| BP | GO:0045061 | thymic T cell selection | 6/1271 | 22/18723 | 0.002806 | 0.036863 | 0.030382 | CD3D/CD3G/AIRE/TOX/CD74/PTPRC | 6 |
| BP | GO:0002758 | innate immune response-activating signal transduction | 6/1271 | 23/18723 | 0.003582 | 0.043058 | 0.035487 | FCN1/FFAR2/TYROBP/PLCG2/PAK3/LILRA2 | 6 |
| BP | GO:0018279 | protein N-linked glycosylation via asparagine | 6/1271 | 23/18723 | 0.003582 | 0.043058 | 0.035487 | RPN1/UGGT1/UBE2J1/FUT8/ST6GAL1/MGAT2 | 6 |
| BP | GO:0033081 | regulation of T cell differentiation in thymus | 6/1271 | 23/18723 | 0.003582 | 0.043058 | 0.035487 | ADAM8/IL7R/BMP4/ADA/CLPTM1/TOX | 6 |
| BP | GO:0042026 | protein refolding | 6/1271 | 23/18723 | 0.003582 | 0.043058 | 0.035487 | HSPA1L/HSPA13/HSPA1B/DNAJA4/HSPA2/HSPA1A | 6 |
| BP | GO:0001779 | natural killer cell differentiation | 6/1271 | 24/18723 | 0.004505 | 0.050533 | 0.041649 | SLAMF1/PIK3CD/MERTK/PRDM1/TOX/PTPRC | 6 |
| BP | GO:0001911 | negative regulation of leukocyte mediated cytotoxicity | 6/1271 | 24/18723 | 0.004505 | 0.050533 | 0.041649 | LILRB1/ARRB2/IL7R/NCKAP1L/PTPRC/SERPINB4 | 6 |
| BP | GO:0018196 | peptidyl-asparagine modification | 6/1271 | 24/18723 | 0.004505 | 0.050533 | 0.041649 | RPN1/UGGT1/UBE2J1/FUT8/ST6GAL1/MGAT2 | 6 |
| BP | GO:0032928 | regulation of superoxide anion generation | 6/1271 | 24/18723 | 0.004505 | 0.050533 | 0.041649 | CYBA/MAPT/F2RL1/TYROBP/AGT/FPR2 | 6 |
| BP | GO:0060571 | morphogenesis of an epithelial fold | 6/1271 | 24/18723 | 0.004505 | 0.050533 | 0.041649 | BMP5/FGFR2/BMP4/WNT2B/TP63/SOSTDC1 | 6 |
| BP | GO:0003071 | renal system process involved in regulation of systemic arterial blood pressure | 6/1271 | 25/18723 | 0.005593 | 0.056045 | 0.046192 | CYBA/CYP4F12/F2RL1/AGT/GJA1/EMP2 | 6 |
| BP | GO:0010996 | response to auditory stimulus | 6/1271 | 25/18723 | 0.005593 | 0.056045 | 0.046192 | SLITRK6/STRA6/PTN/NEUROG1/ABHD12/NRXN1 | 6 |
| BP | GO:0031664 | regulation of lipopolysaccharide-mediated signaling pathway | 6/1271 | 25/18723 | 0.005593 | 0.056045 | 0.046192 | CD180/LY96/TRIB1/LILRA2/LTF/BMP6 | 6 |
| BP | GO:0061436 | establishment of skin barrier | 6/1271 | 25/18723 | 0.005593 | 0.056045 | 0.046192 | KRT1/FLG2/KDF1/TP63/ALOXE3/KRT16 | 6 |
| BP | GO:0062009 | secondary palate development | 6/1271 | 25/18723 | 0.005593 | 0.056045 | 0.046192 | TBX1/SOX11/FOXE1/TGFBR3/COL11A2/ITGB8 | 6 |
| BP | GO:0070391 | response to lipoteichoic acid | 5/1271 | 10/18723 | 0.00027 | 0.007011 | 0.005778 | TREM2/CD14/TLR2/LBP/TLR4 | 5 |
| BP | GO:0071223 | cellular response to lipoteichoic acid | 5/1271 | 10/18723 | 0.00027 | 0.007011 | 0.005778 | TREM2/CD14/TLR2/LBP/TLR4 | 5 |
| BP | GO:2000252 | negative regulation of feeding behavior | 5/1271 | 10/18723 | 0.00027 | 0.007011 | 0.005778 | CCK/NPY2R/MC4R/NMU/RETN | 5 |
| BP | GO:0098883 | synapse pruning | 5/1271 | 11/18723 | 0.000468 | 0.010113 | 0.008335 | TREM2/C3/C1QB/C1QA/DKK1 | 5 |
| BP | GO:0010454 | negative regulation of cell fate commitment | 5/1271 | 12/18723 | 0.000757 | 0.014474 | 0.011929 | MESP1/HES1/SOSTDC1/SFRP2/DKK1 | 5 |
| BP | GO:0045916 | negative regulation of complement activation | 5/1271 | 12/18723 | 0.000757 | 0.014474 | 0.011929 | CR2/CR1L/SUSD4/CR1/VSIG4 | 5 |
| BP | GO:0060907 | positive regulation of macrophage cytokine production | 5/1271 | 12/18723 | 0.000757 | 0.014474 | 0.011929 | LILRB1/PLCG2/SEMA7A/CD74/TLR4 | 5 |
| BP | GO:0002430 | complement receptor mediated signaling pathway | 5/1271 | 13/18723 | 0.001161 | 0.01957 | 0.01613 | CR2/CR1/FPR1/FPR2/C3AR1 | 5 |
| BP | GO:0034135 | regulation of toll-like receptor 2 signaling pathway | 5/1271 | 13/18723 | 0.001161 | 0.01957 | 0.01613 | CYBA/TREM2/F2RL1/TLR1/TLR6 | 5 |
| BP | GO:0036005 | response to macrophage colony-stimulating factor | 5/1271 | 13/18723 | 0.001161 | 0.01957 | 0.01613 | STAP1/TREM2/MST1R/TLR2/TLR4 | 5 |
| BP | GO:0036006 | cellular response to macrophage colony-stimulating factor stimulus | 5/1271 | 13/18723 | 0.001161 | 0.01957 | 0.01613 | STAP1/TREM2/MST1R/TLR2/TLR4 | 5 |
| BP | GO:0070424 | regulation of nucleotide-binding oligomerization domain containing signaling pathway | 5/1271 | 13/18723 | 0.001161 | 0.01957 | 0.01613 | HSPA1B/BIRC3/SLC15A2/HSPA1A/TLR4 | 5 |
| BP | GO:0002756 | MyD88-independent toll-like receptor signaling pathway | 5/1271 | 14/18723 | 0.001706 | 0.025477 | 0.020998 | RAB11FIP2/TLR6/TNIP3/TLR3/TLR4 | 5 |
| BP | GO:0007171 | activation of transmembrane receptor protein tyrosine kinase activity | 5/1271 | 14/18723 | 0.001706 | 0.025477 | 0.020998 | CHRNA3/EGF/EFNA5/ADRB2/PRLR | 5 |
| BP | GO:0031650 | regulation of heat generation | 5/1271 | 14/18723 | 0.001706 | 0.025477 | 0.020998 | TNFSF11/IL1B/PTGES/NMU/APLN | 5 |
| BP | GO:0045059 | positive thymic T cell selection | 5/1271 | 14/18723 | 0.001706 | 0.025477 | 0.020998 | CD3D/CD3G/TOX/CD74/PTPRC | 5 |
| BP | GO:0002679 | respiratory burst involved in defense response | 5/1271 | 15/18723 | 0.002417 | 0.03288 | 0.027099 | TREM2/PIK3CD/PIK3CG/LBP/MPO | 5 |
| BP | GO:0042976 | activation of Janus kinase activity | 5/1271 | 15/18723 | 0.002417 | 0.03288 | 0.027099 | GHR/PTK2B/AGT/PRLR/CCL5 | 5 |
| BP | GO:0048484 | enteric nervous system development | 5/1271 | 15/18723 | 0.002417 | 0.03288 | 0.027099 | ARX/HLX/RHOXF1/SOX10/RET | 5 |
| BP | GO:0001977 | renal system process involved in regulation of blood volume | 5/1271 | 16/18723 | 0.003321 | 0.040852 | 0.03367 | CYBA/CYP4F12/F2RL1/GJA1/EMP2 | 5 |
| BP | GO:0002921 | negative regulation of humoral immune response | 5/1271 | 16/18723 | 0.003321 | 0.040852 | 0.03367 | CR2/CR1L/SUSD4/CR1/VSIG4 | 5 |
| BP | GO:0060572 | morphogenesis of an epithelial bud | 5/1271 | 16/18723 | 0.003321 | 0.040852 | 0.03367 | FGFR2/BMP4/WNT2B/TP63/SOSTDC1 | 5 |
| BP | GO:0001710 | mesodermal cell fate commitment | 5/1271 | 17/18723 | 0.004445 | 0.050369 | 0.041514 | MESP1/BMP4/KLF4/SFRP2/DKK1 | 5 |
| BP | GO:0001780 | neutrophil homeostasis | 5/1271 | 17/18723 | 0.004445 | 0.050369 | 0.041514 | MPL/PIK3CD/MERTK/PDE4B/IL6 | 5 |
| BP | GO:0010934 | macrophage cytokine production | 5/1271 | 17/18723 | 0.004445 | 0.050369 | 0.041514 | LILRB1/PLCG2/SEMA7A/CD74/TLR4 | 5 |
| BP | GO:0010935 | regulation of macrophage cytokine production | 5/1271 | 17/18723 | 0.004445 | 0.050369 | 0.041514 | LILRB1/PLCG2/SEMA7A/CD74/TLR4 | 5 |
| BP | GO:0016264 | gap junction assembly | 5/1271 | 17/18723 | 0.004445 | 0.050369 | 0.041514 | IL1B/ACE2/AGT/GJA1/GJB6 | 5 |
| BP | GO:0033623 | regulation of integrin activation | 5/1271 | 17/18723 | 0.004445 | 0.050369 | 0.041514 | SKAP1/PLEK/SELP/CXCL13/FERMT1 | 5 |
| BP | GO:0002544 | chronic inflammatory response | 5/1271 | 18/18723 | 0.005815 | 0.056802 | 0.046815 | CXCL13/GJA1/PTGES/CCL5/CCL11 | 5 |
| BP | GO:0003417 | growth plate cartilage development | 5/1271 | 18/18723 | 0.005815 | 0.056802 | 0.046815 | NPPC/ANXA6/COMP/RARA/MMP13 | 5 |
| BP | GO:0006957 | complement activation, alternative pathway | 5/1271 | 18/18723 | 0.005815 | 0.056802 | 0.046815 | CR2/SUSD4/C3/CR1/VSIG4 | 5 |
| BP | GO:0031643 | positive regulation of myelination | 5/1271 | 18/18723 | 0.005815 | 0.056802 | 0.046815 | ITGAX/CST7/PARD3/NCMAP/SOX10 | 5 |
| BP | GO:0051782 | negative regulation of cell division | 5/1271 | 18/18723 | 0.005815 | 0.056802 | 0.046815 | MYC/TEX14/C10orf99/E2F8/AURKB | 5 |
| BP | GO:0150078 | positive regulation of neuroinflammatory response | 5/1271 | 18/18723 | 0.005815 | 0.056802 | 0.046815 | STAP1/TREM2/IL1B/PLCG2/IL6 | 5 |
| BP | GO:0001915 | negative regulation of T cell mediated cytotoxicity | 4/1271 | 10/18723 | 0.003186 | 0.039738 | 0.032752 | LILRB1/IL7R/NCKAP1L/PTPRC | 4 |
| BP | GO:0002934 | desmosome organization | 4/1271 | 10/18723 | 0.003186 | 0.039738 | 0.032752 | PKP3/DSP/JUP/PERP | 4 |
| BP | GO:0003129 | heart induction | 4/1271 | 10/18723 | 0.003186 | 0.039738 | 0.032752 | ROBO2/MESP1/BMP4/DKK1 | 4 |
| BP | GO:0042756 | drinking behavior | 4/1271 | 10/18723 | 0.003186 | 0.039738 | 0.032752 | ACE2/AGT/EN1/APLN | 4 |
| BP | GO:1903867 | extraembryonic membrane development | 4/1271 | 10/18723 | 0.003186 | 0.039738 | 0.032752 | DNAJB6/FZD5/BMP5/E2F8 | 4 |
| BP | GO:0001867 | complement activation, lectin pathway | 4/1271 | 11/18723 | 0.004739 | 0.050652 | 0.041747 | KRT1/FCN1/FCN2/FCN3 | 4 |
| BP | GO:0002759 | regulation of antimicrobial humoral response | 4/1271 | 11/18723 | 0.004739 | 0.050652 | 0.041747 | IL17F/IL17A/PGC/KLK7 | 4 |
| BP | GO:0002924 | negative regulation of humoral immune response mediated by circulating immunoglobulin | 4/1271 | 11/18723 | 0.004739 | 0.050652 | 0.041747 | CR2/CR1L/SUSD4/CR1 | 4 |
| BP | GO:0003093 | regulation of glomerular filtration | 4/1271 | 11/18723 | 0.004739 | 0.050652 | 0.041747 | CYBA/F2RL1/GJA1/EMP2 | 4 |
| BP | GO:0003139 | secondary heart field specification | 4/1271 | 11/18723 | 0.004739 | 0.050652 | 0.041747 | MESP1/BMP4/FOXH1/LRP2 | 4 |
| BP | GO:0014820 | tonic smooth muscle contraction | 4/1271 | 11/18723 | 0.004739 | 0.050652 | 0.041747 | CD38/AGT/NMU/EDN2 | 4 |
| BP | GO:0031652 | positive regulation of heat generation | 4/1271 | 11/18723 | 0.004739 | 0.050652 | 0.041747 | TNFSF11/IL1B/NMU/APLN | 4 |
| BP | GO:0032308 | positive regulation of prostaglandin secretion | 4/1271 | 11/18723 | 0.004739 | 0.050652 | 0.041747 | PLA2G3/TNFSF11/IL1B/PTGES | 4 |
| BP | GO:0032493 | response to bacterial lipoprotein | 4/1271 | 11/18723 | 0.004739 | 0.050652 | 0.041747 | CD14/TLR1/TLR2/TLR6 | 4 |
| BP | GO:0033625 | positive regulation of integrin activation | 4/1271 | 11/18723 | 0.004739 | 0.050652 | 0.041747 | SKAP1/PLEK/CXCL13/FERMT1 | 4 |
| BP | GO:0070243 | regulation of thymocyte apoptotic process | 4/1271 | 11/18723 | 0.004739 | 0.050652 | 0.041747 | ADAM8/BMP4/ADA/EFNA1 | 4 |
| BP | GO:0071492 | cellular response to UV-A | 4/1271 | 11/18723 | 0.004739 | 0.050652 | 0.041747 | MMP3/MMP1/TIMP1/MMP9 | 4 |
| BP | GO:1900426 | positive regulation of defense response to bacterium | 4/1271 | 11/18723 | 0.004739 | 0.050652 | 0.041747 | CYBA/F2RL1/PGC/KLK7 | 4 |
| BP | GO:2000826 | regulation of heart morphogenesis | 4/1271 | 11/18723 | 0.004739 | 0.050652 | 0.041747 | ROBO2/MESP1/BMP4/DKK1 | 4 |
| CC | GO:0070820 | tertiary granule | 39/1331 | 164/19550 | 3.71E-12 | 2.15E-09 | 1.95E-09 | CYBA/ARL8A/ITGAX/CXCL1/SIGLEC5/CLEC4D/CTSS/TCIRG1/FLG2/CD53/ENPP4/ADAM8/TNFAIP6/CR1/LILRB2/SERPINB10/LAIR1/FPR1/MGAM/LTF/DSP/CLEC12A/CTSH/CYBB/PKP1/NCKAP1L/ADGRE3/PLD1/MMP9/GPR84/FPR2/CD93/CAMP/RAP2B/SVIP/FCAR/FCER1G/LYZ/CRISP3 | 39 |
| CC | GO:0030667 | secretory granule membrane | 53/1331 | 311/19550 | 4.98E-10 | 1.44E-07 | 1.31E-07 | SPACA3/CYBA/ARL8A/ITGAX/P2RX1/SIGLEC5/CLEC4D/SYTL4/TCIRG1/CD38/LILRB3/EQTN/SELL/CD14/CD53/ENPP4/ADAM8/TYROBP/TMEM184A/GLIPR1/CR1/LILRB2/TMEM95/SERPINB10/CXCR1/SELP/LAIR1/FPR1/PECAM1/MGAM/DSP/CLEC12A/TEX101/DEGS1/CYBB/PCDH7/PKP1/NCKAP1L/STXBP5/ADGRE3/TLR2/PLD1/GPR84/FPR2/C3AR1/CD93/RAP2B/SVIP/FCAR/FCER1G/RAB27B/PTPRC/CD9 | 53 |
| CC | GO:0101003 | ficolin-1-rich granule membrane | 20/1331 | 61/19550 | 1.68E-09 | 3.23E-07 | 2.94E-07 | ARL8A/ITGAX/SIGLEC5/CLEC4D/TCIRG1/ENPP4/ADAM8/CR1/LILRB2/SERPINB10/FPR1/MGAM/DSP/PKP1/NCKAP1L/ADGRE3/FPR2/CD93/FCAR/FCER1G | 20 |
| CC | GO:0101002 | ficolin-1-rich granule | 37/1331 | 185/19550 | 2.50E-09 | 3.62E-07 | 3.30E-07 | ARL8A/ITGAX/KRT1/CALML5/SIGLEC5/GMFG/CLEC4D/FCN1/CTSS/TCIRG1/PRDX4/BIN2/ENPP4/ADAM8/TNFAIP6/HSPA1B/CR1/LILRB2/GNS/SERPINB10/MNDA/FPR1/MGAM/ARSB/DSP/CTSH/PKP1/NCKAP1L/ADGRE3/MMP9/FPR2/JUP/CD93/FCAR/FCER1G/HSPA1A/PGM2 | 37 |
| CC | GO:0001533 | cornified envelope | 15/1331 | 45/19550 | 1.43E-07 | 1.65E-05 | 1.50E-05 | KRT1/PKP3/KRT10/RPTN/KRT2/FLG2/DSP/PKP1/DSC3/TCHH/JUP/DSC2/DSG3/PPL/PI3 | 15 |
| CC | GO:0070821 | tertiary granule membrane | 19/1331 | 73/19550 | 2.73E-07 | 2.63E-05 | 2.40E-05 | CYBA/ITGAX/SIGLEC5/CLEC4D/CD53/ADAM8/LILRB2/LAIR1/MGAM/CLEC12A/CYBB/PLD1/GPR84/FPR2/CD93/RAP2B/SVIP/FCAR/FCER1G | 19 |
| CC | GO:0030057 | desmosome | 10/1331 | 25/19550 | 2.63E-06 | 0.000217 | 0.000197 | PKP3/POF1B/DSP/PKP1/DSC3/JUP/DSC2/DSG3/PPL/PERP | 10 |
| CC | GO:0005911 | cell-cell junction | 60/1331 | 494/19550 | 9.06E-06 | 0.000558 | 0.000508 | SKAP1/PKP3/CDC42BPA/KCNA5/VANGL2/VAV1/KRT8/CD53/WNK3/SSX2IP/FZD5/NHS/EPCAM/POF1B/CLIC4/FGFR4/OCLN/SHROOM1/SLC2A1/CLDN22/PARD3/SH3KBP1/KRT18/PANX2/TIAM1/PECAM1/PODXL/GJB5/KCNA2/ITK/MARVELD3/DSP/JAG1/AJUBA/PKP1/CLDN17/MPP7/GJA1/DSC3/GRHL2/NDRG1/CLDN3/JUP/MARVELD2/DSC2/PARD6A/RAP2B/CXADR/DSG3/PPL/MXRA8/CLDN10/GJB6/FAT2/PERP/CDH18/AQP3/F11R/CDH1/CDH7 | 60 |
| CC | GO:0045121 | membrane raft | 45/1331 | 335/19550 | 9.65E-06 | 0.000558 | 0.000508 | SLC6A4/CD19/KCNA3/LAT2/TREM2/CHRNA3/MAPT/P2RX1/SKAP1/MS4A4A/CD226/KCNA5/MALL/CD79A/CD14/PTK2B/EFNA5/LY6K/CNTN1/CR1/SLC2A1/SYNJ2/MS4A1/TLR1/GRIP1/BIRC3/ARID3C/CBL/PECAM1/PODXL/SELPLG/TEX101/ACE2/TLR2/GJA1/TLR6/RET/RAP2B/CXADR/PSEN2/EMP2/PTPRC/ATP1B3/CDH1/MAL2 | 45 |
| CC | GO:0098857 | membrane microdomain | 45/1331 | 335/19550 | 9.65E-06 | 0.000558 | 0.000508 | SLC6A4/CD19/KCNA3/LAT2/TREM2/CHRNA3/MAPT/P2RX1/SKAP1/MS4A4A/CD226/KCNA5/MALL/CD79A/CD14/PTK2B/EFNA5/LY6K/CNTN1/CR1/SLC2A1/SYNJ2/MS4A1/TLR1/GRIP1/BIRC3/ARID3C/CBL/PECAM1/PODXL/SELPLG/TEX101/ACE2/TLR2/GJA1/TLR6/RET/RAP2B/CXADR/PSEN2/EMP2/PTPRC/ATP1B3/CDH1/MAL2 | 45 |
| CC | GO:0009897 | external side of plasma membrane | 51/1331 | 421/19550 | 4.47E-05 | 0.002347 | 0.002137 | CD19/CTLA4/SLAMF1/LILRB1/ITGAX/P2RX1/IGLL1/CD3D/SLAMF7/CD163L1/CD226/IL31RA/FCN1/CD86/CD79A/GHR/CD80/SLC7A5/CD14/CCR3/EFNA5/CD3G/CXCR4/IL2RG/CXCR1/MS4A1/IL17A/ACKR4/SELP/KRT18/IL7R/TNFRSF9/IL2RA/SEMA7A/BTN3A1/ADA/PRLR/LY6G5C/TGFBR3/CRLF1/CLPTM1/CD1E/LRP2/CD74/FCER1G/CXCL12/PTPRC/TLR4/CD9/LEPR/TNFRSF4 | 51 |
| CC | GO:0005788 | endoplasmic reticulum lumen | 40/1331 | 313/19550 | 9.09E-05 | 0.004106 | 0.003739 | COL4A4/MZB1/BCHE/ERLEC1/PDIA4/TXNDC5/COL4A6/CYP2W1/COL4A2/SCG2/IGFBP3/F5/ARSF/FLT3/MIA3/TSPAN5/DBI/CALU/DNAJB9/C3/SHISA5/UGGT1/DNAJC10/MYDGF/SDF2L1/TMEM43/ARSB/BMP4/PDIA6/COL15A1/ACE2/PENK/DNAJC3/TIMP1/COL21A1/ARSD/COL11A2/IL6/MXRA8/COL9A1 | 40 |
| CC | GO:0016324 | apical plasma membrane | 45/1331 | 367/19550 | 9.24E-05 | 0.004106 | 0.003739 | STK39/CYBA/P2RY1/CYP4F12/DPEP1/KCNA5/VANGL2/TCIRG1/SLC7A5/AQP6/ADRB2/NHS/EPCAM/NAALADL1/OCLN/SHROOM1/SLC2A1/SLC7A8/PARD3/ACY3/MGAM/PODXL/SLC15A2/ACE2/JAG1/CTSL/GJA1/PLD1/TLR9/SLC22A12/SLCO2B1/MARVELD2/PARD6A/PSEN2/LRP2/SLC26A4/KIAA1614/RAB27B/EMP2/ATP1B3/GJB6/FZD6/CD9/MAL2/SLC9A3 | 45 |
| CC | GO:0043296 | apical junction complex | 22/1331 | 145/19550 | 0.000326 | 0.01345 | 0.012247 | WNK3/FZD5/NHS/EPCAM/POF1B/OCLN/SHROOM1/CLDN22/PARD3/MARVELD3/CLDN17/MPP7/CLDN3/JUP/MARVELD2/PARD6A/RAP2B/CXADR/MXRA8/CLDN10/F11R/CDH1 | 22 |
| CC | GO:0035579 | specific granule membrane | 16/1331 | 91/19550 | 0.000393 | 0.015152 | 0.013797 | CYBA/P2RX1/CLEC4D/CD53/ADAM8/LAIR1/CLEC12A/DEGS1/CYBB/PLD1/GPR84/FPR2/C3AR1/CD93/RAP2B/FCAR | 16 |
| CC | GO:0070160 | tight junction | 20/1331 | 129/19550 | 0.000449 | 0.016116 | 0.014675 | WNK3/FZD5/NHS/EPCAM/POF1B/OCLN/CLDN22/PARD3/MARVELD3/CLDN17/MPP7/GJA1/CLDN3/MARVELD2/PARD6A/RAP2B/CXADR/MXRA8/CLDN10/F11R | 20 |
| CC | GO:1990124 | messenger ribonucleoprotein complex | 5/1331 | 11/19550 | 0.000474 | 0.016116 | 0.014675 | PKP3/CPEB2/PKP1/CPEB4/HNRNPA3 | 5 |
| CC | GO:0042581 | specific granule | 23/1331 | 160/19550 | 0.000531 | 0.017066 | 0.01554 | CYBA/P2RX1/CXCL1/CLEC4D/CD53/ADAM8/LAIR1/LTF/CLEC12A/DEGS1/CYBB/PLD1/GPR84/FPR2/C3AR1/JUP/CD93/CAMP/RAP2B/FCAR/RETN/LYZ/CRISP3 | 23 |
| CC | GO:0005923 | bicellular tight junction | 19/1331 | 122/19550 | 0.000582 | 0.017708 | 0.016125 | WNK3/FZD5/NHS/EPCAM/POF1B/OCLN/CLDN22/PARD3/MARVELD3/CLDN17/MPP7/CLDN3/MARVELD2/PARD6A/RAP2B/CXADR/MXRA8/CLDN10/F11R | 19 |
| CC | GO:0045177 | apical part of cell | 48/1331 | 435/19550 | 0.000654 | 0.018903 | 0.017213 | STK39/CYBA/P2RY1/CYP4F12/DPEP1/KCNA5/VANGL2/TCIRG1/SLC7A5/AQP6/ADRB2/NHS/EPCAM/NAALADL1/CLIC4/OCLN/SHROOM1/SLC2A1/SLC7A8/PARD3/HOMER2/ACY3/MGAM/PODXL/SLC15A2/ACE2/JAG1/CTSL/GJA1/PLD1/TLR9/SLC22A12/SLCO2B1/MYO6/MARVELD2/PARD6A/PSEN2/LRP2/SLC26A4/KIAA1614/RAB27B/EMP2/ATP1B3/GJB6/FZD6/CD9/MAL2/SLC9A3 | 48 |
| CC | GO:0030139 | endocytic vesicle | 39/1331 | 336/19550 | 0.000782 | 0.021523 | 0.019598 | CYBA/CTLA4/SLAMF1/TYRP1/EGF/CD3D/CTSS/TCIRG1/ARRB2/RAB20/NCF4/FZD5/ADAM8/CD3G/ADRB2/CD207/OCLN/SH3KBP1/TLR1/IL7R/LTF/CLEC4E/ACE2/CYBB/NCF2/GNLY/SAA1/TLR2/CTSL/PLD1/TLR9/STX6/MYO6/TLR6/MARCO/LRP2/CD74/MPO/CD9 | 39 |
| CC | GO:0005882 | intermediate filament | 27/1331 | 217/19550 | 0.00176 | 0.04623 | 0.042096 | KRT3/NEFL/KRT85/KRT1/KRT10/NEFM/BFSP2/KRT2/KRT8/LMNB1/KRT75/KRT27/KRT18/KRT33A/EIF6/DSP/PKP1/LMNTD2/KRT76/KRT80/JUP/IFFO2/PPL/KRT16/KRT13/KRT6B/KRT24 | 27 |
| CC | GO:0140534 | endoplasmic reticulum protein-containing complex | 18/1331 | 125/19550 | 0.002005 | 0.050393 | 0.045887 | SPCS3/SPTSSB/MZB1/SEC11C/SSR4/SEL1L/KRTCAP2/RPN1/ELOVL6/DNAJC10/PIGA/RHBDD2/ERN1/SDF2L1/RYR1/PDIA6/PIGK/SRPRB | 18 |
| CC | GO:0016327 | apicolateral plasma membrane | 6/1331 | 21/19550 | 0.002197 | 0.052908 | 0.048177 | KRT8/OCLN/CLDN3/JUP/CXADR/FZD6 | 6 |
| MF | GO:0140375 | immune receptor activity | 33/1309 | 144/18368 | 1.49E-09 | 1.45E-06 | 1.33E-06 | LILRB1/CR2/LILRA1/IL31RA/MPL/LILRB3/GHR/CCR3/LILRA5/IL17RD/FLT3/CXCR4/CR1/LILRB2/LILRA6/IL2RG/CXCR1/ACKR4/LILRA2/IL7R/FPR1/IL22RA2/IL2RA/CTSH/PRLR/CRLF1/IL20RA/FPR2/C3AR1/CD74/FCER1G/IL20RB/LEPR | 33 |
| MF | GO:0051787 | misfolded protein binding | 12/1309 | 29/18368 | 2.71E-07 | 0.000132 | 0.000121 | F12/EDEM1/HSPA1L/HSPA13/HSPA1B/DNAJB9/HSPA2/DNAJC10/RHBDD2/SDF2L1/DNAJC3/HSPA1A | 12 |
| MF | GO:0030546 | signaling receptor activator activity | 63/1309 | 495/18368 | 5.01E-06 | 0.001145 | 0.00105 | CCK/IL26/CXCL6/CXCL1/IL24/PNOC/EGF/GMFG/CCL18/SCG2/IL17F/CXCL3/EFNA5/TG/MIA/FGF19/NPPC/TNFSF11/CMTM2/IL1B/BMP5/LTB/IL17A/CXCL2/SLURP1/PTN/FAM3B/UTS2B/BMP4/CCL13/WNT16/CXCL13/SEMA7A/TNFSF8/SST/PENK/C10orf99/JAG1/PF4V1/WNT2B/AGT/IL21/TIMP1/CRLF1/NRG4/NPPB/IL6/BMP6/CCL5/RETN/GPNMB/NPY/IL17D/IL1F10/CCL11/CXCL12/IL37/SFRP2/APLN/DKK1/PSPN/EDN2/MACC1 | 63 |
| MF | GO:0032396 | inhibitory MHC class I receptor activity | 7/1309 | 12/18368 | 5.29E-06 | 0.001145 | 0.00105 | LILRB1/LILRA1/LILRB3/LILRA5/LILRB2/LILRA6/LILRA2 | 7 |
| MF | GO:0048018 | receptor ligand activity | 62/1309 | 487/18368 | 5.89E-06 | 0.001145 | 0.00105 | CCK/IL26/CXCL6/CXCL1/IL24/PNOC/EGF/GMFG/CCL18/SCG2/IL17F/CXCL3/TG/MIA/FGF19/NPPC/TNFSF11/CMTM2/IL1B/BMP5/LTB/IL17A/CXCL2/SLURP1/PTN/FAM3B/UTS2B/BMP4/CCL13/WNT16/CXCL13/SEMA7A/TNFSF8/SST/PENK/C10orf99/JAG1/PF4V1/WNT2B/AGT/IL21/TIMP1/CRLF1/NRG4/NPPB/IL6/BMP6/CCL5/RETN/GPNMB/NPY/IL17D/IL1F10/CCL11/CXCL12/IL37/SFRP2/APLN/DKK1/PSPN/EDN2/MACC1 | 62 |
| MF | GO:0032393 | MHC class I receptor activity | 8/1309 | 17/18368 | 8.85E-06 | 0.001433 | 0.001314 | LILRB1/LILRA1/LILRB3/LILRA5/LILRB2/LILRA6/LILRA2/CTSH | 8 |
| MF | GO:0005125 | cytokine activity | 36/1309 | 235/18368 | 1.08E-05 | 0.001498 | 0.001374 | IL26/CXCL6/CXCL1/IL24/CCL18/SCG2/IL17F/CXCL3/TNFSF11/CMTM2/IL1B/BMP5/LTB/IL17A/CXCL2/SLURP1/FAM3B/BMP4/CCL13/WNT16/CXCL13/TNFSF8/C10orf99/PF4V1/WNT2B/IL21/TIMP1/CRLF1/IL6/BMP6/CCL5/IL17D/IL1F10/CCL11/CXCL12/IL37 | 36 |
| MF | GO:0071723 | lipopeptide binding | 6/1309 | 10/18368 | 2.12E-05 | 0.002574 | 0.002361 | CD14/TLR1/TLR2/CD1E/TLR6/LBP | 6 |
| MF | GO:0004896 | cytokine receptor activity | 19/1309 | 97/18368 | 4.67E-05 | 0.004611 | 0.004229 | IL31RA/MPL/GHR/CCR3/IL17RD/FLT3/CXCR4/IL2RG/CXCR1/ACKR4/IL7R/IL22RA2/IL2RA/PRLR/CRLF1/IL20RA/CD74/IL20RB/LEPR | 19 |
| MF | GO:0038187 | pattern recognition receptor activity | 9/1309 | 26/18368 | 4.74E-05 | 0.004611 | 0.004229 | CLEC4D/FCN1/CD14/LY96/CLEC4E/TLR2/TLR9/MARCO/TLR4 | 9 |
| MF | GO:0001664 | G protein-coupled receptor binding | 40/1309 | 295/18368 | 6.50E-05 | 0.005369 | 0.004925 | P2RY1/CXCL6/CXCL1/PNOC/BAMBI/FCN1/PROK2/CCL18/TULP3/ARRB2/CXCL3/HSPA1B/C3/HOMER2/SH3GL1/CXCL2/FPR1/UTS2B/CCL13/WNT16/CXCL13/PENK/C10orf99/PF4V1/WNT2B/AGT/SAA1/ADRB1/NMU/MARCO/CCL5/REEP2/S100A14/RSPO3/NPY/HSPA1A/CCL11/CXCL12/APLN/EDN2 | 40 |
| MF | GO:0001530 | lipopolysaccharide binding | 10/1309 | 33/18368 | 6.63E-05 | 0.005369 | 0.004925 | TREM2/CD14/LY96/SELP/BPIFC/LTF/TLR2/CAMP/LBP/TLR4 | 10 |
| MF | GO:0003953 | NAD+ nucleosidase activity | 9/1309 | 28/18368 | 9.21E-05 | 0.006884 | 0.006315 | TLR10/CD38/BANK1/TLR1/TLR2/TLR9/TLR6/TLR3/TLR4 | 9 |
| MF | GO:0004252 | serine-type endopeptidase activity | 27/1309 | 174/18368 | 0.000105 | 0.007278 | 0.006676 | PCSK2/TLL2/F12/SEC11C/MMP3/MMP1/CTSS/MMP10/ADAM8/TYSND1/RHBDD1/GZMB/TMPRSS13/KLK11/RHBDD2/LTF/CTSH/HTRA4/MMP9/KLK7/MMP12/MMP13/TMPRSS11B/TMPRSS11D/PRSS50/MMP7/GZMM | 27 |
| MF | GO:0008009 | chemokine activity | 12/1309 | 49/18368 | 0.000127 | 0.008227 | 0.007546 | CXCL6/CXCL1/CCL18/CXCL3/CXCL2/CCL13/CXCL13/C10orf99/PF4V1/CCL5/CCL11/CXCL12 | 12 |
| MF | GO:0045236 | CXCR chemokine receptor binding | 7/1309 | 18/18368 | 0.000145 | 0.008823 | 0.008093 | CXCL6/CXCL1/CXCL3/CXCL2/CXCL13/PF4V1/CXCL12 | 7 |
| MF | GO:0017171 | serine hydrolase activity | 28/1309 | 195/18368 | 0.000302 | 0.017207 | 0.015783 | PCSK2/TLL2/F12/AADAC/SEC11C/MMP3/MMP1/CTSS/MMP10/ADAM8/TYSND1/RHBDD1/GZMB/TMPRSS13/KLK11/RHBDD2/LTF/CTSH/HTRA4/MMP9/KLK7/MMP12/MMP13/TMPRSS11B/TMPRSS11D/PRSS50/MMP7/GZMM | 28 |
| MF | GO:0004364 | glutathione transferase activity | 8/1309 | 26/18368 | 0.000319 | 0.017207 | 0.015783 | GSTA5/GSTA4/GSTA1/ALOX5AP/LTC4S/PTGES/GSTA2/GSTA3 | 8 |
| MF | GO:0030280 | structural constituent of skin epidermis | 6/1309 | 15/18368 | 0.00037 | 0.018925 | 0.017359 | KRT1/KRT10/KRT2/PNPLA1/PKP1/PI3 | 6 |
| MF | GO:0005539 | glycosaminoglycan binding | 31/1309 | 230/18368 | 0.000458 | 0.02163 | 0.019841 | TREM2/CXCL6/VIT/JCHAIN/SELL/TNFAIP6/PLA2G2D/TMEM184A/FGFR4/GNS/SELP/ANXA6/FGFR2/LRTM1/PTN/LTF/BMP4/PTPRF/CXCL13/PF4V1/SAA1/TGFBR3/TLR2/COMP/FGFBP1/GPNMB/RSPO3/PTPRC/MPO/MMP7/HAPLN1 | 31 |
| MF | GO:0008236 | serine-type peptidase activity | 27/1309 | 191/18368 | 0.000492 | 0.02163 | 0.019841 | PCSK2/TLL2/F12/SEC11C/MMP3/MMP1/CTSS/MMP10/ADAM8/TYSND1/RHBDD1/GZMB/TMPRSS13/KLK11/RHBDD2/LTF/CTSH/HTRA4/MMP9/KLK7/MMP12/MMP13/TMPRSS11B/TMPRSS11D/PRSS50/MMP7/GZMM | 27 |
| MF | GO:0004867 | serine-type endopeptidase inhibitor activity | 17/1309 | 98/18368 | 0.000523 | 0.02163 | 0.019841 | SERPINI1/SERPINA11/SERPINI2/SERPINB10/SPINK1/SERPINB7/TFPI2/AGT/SERPINB11/WFDC2/SPINK7/SERPINB5/SPINK9/A2ML1/PI3/SERPINB4/SERPINB13 | 17 |
| MF | GO:0098632 | cell-cell adhesion mediator activity | 11/1309 | 49/18368 | 0.000542 | 0.02163 | 0.019841 | IGSF9/PKP3/EMB/EPCAM/CD200/KRT18/DSP/JUP/DSC2/CXADR/CNTN6 | 11 |
| MF | GO:0050135 | NAD(P)+ nucleosidase activity | 6/1309 | 16/18368 | 0.000556 | 0.02163 | 0.019841 | TLR10/CD38/TLR1/TLR2/TLR6/TLR4 | 6 |
| MF | GO:0061809 | NAD+ nucleotidase, cyclic ADP-ribose generating | 6/1309 | 16/18368 | 0.000556 | 0.02163 | 0.019841 | TLR10/CD38/TLR1/TLR2/TLR6/TLR4 | 6 |
| MF | GO:0086080 | protein binding involved in heterotypic cell-cell adhesion | 5/1309 | 11/18368 | 0.000586 | 0.021899 | 0.020087 | CD200/DSP/JUP/DSC2/CXADR | 5 |
| MF | GO:0061135 | endopeptidase regulator activity | 27/1309 | 194/18368 | 0.00063 | 0.022678 | 0.020801 | DPEP1/SERPINI1/CST7/NLRC4/RARRES1/SERPINA11/C3/SMR3B/SERPINI2/SERPINB10/BIRC3/SPINK1/SERPINB7/LTF/TFPI2/AGT/TIMP1/SERPINB11/WFDC2/SPINK7/SERPINB5/SPINK9/A2ML1/PI3/SFRP2/SERPINB4/SERPINB13 | 27 |
| MF | GO:0043394 | proteoglycan binding | 9/1309 | 36/18368 | 0.000748 | 0.025954 | 0.023806 | CTSS/FCN2/PLA2G2D/PTN/PTPRF/CTSL/COMP/GPNMB/PTPRC | 9 |
| MF | GO:0019955 | cytokine binding | 21/1309 | 139/18368 | 0.000842 | 0.02781 | 0.025509 | IL31RA/ITGA4/GHR/CCR3/IL17F/CXCR4/IL2RG/CXCR1/ACKR4/TNFRSF9/IL22RA2/IL2RA/PRLR/TGFBR3/CRLF1/IL20RA/COMP/CD74/IL20RB/SOSTDC1/LEPR | 21 |
| MF | GO:0001618 | virus receptor activity | 14/1309 | 76/18368 | 0.000858 | 0.02781 | 0.025509 | SLAMF1/CR2/CD86/CD80/HSPA1B/CXCR4/CR1/CLEC4M/SELPLG/ACE2/CXADR/HSPA1A/F11R/TNFRSF4 | 14 |
| MF | GO:0004875 | complement receptor activity | 5/1309 | 12/18368 | 0.000945 | 0.029643 | 0.02719 | CR2/CR1/FPR1/FPR2/C3AR1 | 5 |
| MF | GO:0140272 | exogenous protein binding | 14/1309 | 77/18368 | 0.000982 | 0.02981 | 0.027344 | SLAMF1/CR2/CD86/CD80/HSPA1B/CXCR4/CR1/CLEC4M/SELPLG/ACE2/CXADR/HSPA1A/F11R/TNFRSF4 | 14 |
| MF | GO:0004866 | endopeptidase inhibitor activity | 25/1309 | 180/18368 | 0.001012 | 0.02981 | 0.027344 | DPEP1/SERPINI1/CST7/RARRES1/SERPINA11/C3/SMR3B/SERPINI2/SERPINB10/BIRC3/SPINK1/SERPINB7/LTF/TFPI2/AGT/TIMP1/SERPINB11/WFDC2/SPINK7/SERPINB5/SPINK9/A2ML1/PI3/SERPINB4/SERPINB13 | 25 |
| MF | GO:0005178 | integrin binding | 21/1309 | 144/18368 | 0.001334 | 0.038141 | 0.034986 | ITGAX/TSPAN8/CD226/ITGA4/IL1B/ICAM2/PTN/ADAM23/SEMA7A/LGALS12/COMP/TSPAN4/CXADR/GPNMB/FERMT1/EMP2/ITGB8/CXCL12/CD9/SFRP2/F11R | 21 |
| MF | GO:0042379 | chemokine receptor binding | 13/1309 | 72/18368 | 0.00157 | 0.04293 | 0.039378 | CXCL6/CXCL1/CCL18/CXCL3/CXCL2/CCL13/CXCL13/C10orf99/PF4V1/CCL5/S100A14/CCL11/CXCL12 | 13 |
| MF | GO:0008201 | heparin binding | 23/1309 | 166/18368 | 0.001629 | 0.04293 | 0.039378 | CXCL6/SELL/PLA2G2D/TMEM184A/FGFR4/SELP/FGFR2/LRTM1/PTN/LTF/BMP4/PTPRF/CXCL13/PF4V1/SAA1/TGFBR3/COMP/FGFBP1/GPNMB/RSPO3/PTPRC/MPO/MMP7 | 23 |
| MF | GO:0016712 | oxidoreductase activity, acting on paired donors, with incorporation or reduction of molecular oxygen, reduced flavin or flavoprotein as one donor, and incorporation of one atom of oxygen | 9/1309 | 40/18368 | 0.001678 | 0.04293 | 0.039378 | CYP4F12/CYP4F8/CYP2W1/CYP3A7/CYP39A1/CYP3A5/CYP2C18/CYP2J2/CYP3A43 | 9 |
| MF | GO:0044183 | protein folding chaperone | 9/1309 | 40/18368 | 0.001678 | 0.04293 | 0.039378 | CLGN/DNAJB6/HSPA1L/HSPA13/HSPA1B/HSPA2/DNAJB1/CD74/HSPA1A | 9 |
| MF | GO:0030414 | peptidase inhibitor activity | 25/1309 | 187/18368 | 0.001744 | 0.043471 | 0.039874 | DPEP1/SERPINI1/CST7/RARRES1/SERPINA11/C3/SMR3B/SERPINI2/SERPINB10/BIRC3/SPINK1/SERPINB7/LTF/TFPI2/AGT/TIMP1/SERPINB11/WFDC2/SPINK7/SERPINB5/SPINK9/A2ML1/PI3/SERPINB4/SERPINB13 | 25 |
