# Supplementary material for: Bioinformatics investigation of adaptive immune‐related genes in peri‐implantitis and periodontitis: Characteristics and diagnostic values
Source: Immun Inflamm Dis. 2024 May 23;12(5):e1272. doi: 10.1002/iid3.1272 (PMC11112631; doi:10.1002/iid3.1272)
Supplement: Supplementary file 4 — Supporting information. [file IID3-12-e1272-s003.docx]

**Supplementary Table 4. KEGG enrichment analysis of differential expression genes in PI vs HP groups**

| **ID** | **Description** | **GeneRatio** | **BgRatio** | **pvalue** | **p.adjust** | **qvalue** | **geneID** | **Count** | **GeneRatio** |
| --- | --- | --- | --- | --- | --- | --- | --- | --- | --- |
| hsa04060 | Cytokine-cytokine receptor interaction | 47/631 | 295/8159 | 1.22E-06 | 0.000168 | 0.000152 | TNFRSF17/IL26/CXCL6/TNFRSF25/CXCL1/IL24/ACVR1B/IL31RA/CCL18/MPL/GHR/CCR3/IL17F/CXCL3/EDA2R/CXCR4/TNFSF11/IL1B/BMP5/LTB/IL2RG/CXCR1/IL17A/ACKR4/CXCL2/IL7R/TNFRSF9/BMP4/IL2RA/CCL13/CXCL13/TNFSF8/PF4V1/PRLR/IL21/IL20RA/IL6/BMP6/CCL5/IL17D/IL1F10/IL20RB/CCL11/CXCL12/IL37/LEPR/TNFRSF4 | 47 | 0.0745 |
| hsa05417 | Lipid and atherosclerosis | 37/631 | 215/8159 | 2.72E-06 | 0.00022 | 0.000199 | CYBA/XBP1/CALML5/MMP3/CXCL1/MMP1/VAV1/HSPA1L/NCF4/CD14/PIK3CD/CXCL3/LY96/HSPA1B/MAPK13/HSPA2/IL1B/ERN1/SELP/CXCL2/CYP2J2/POU2F2/BCL2L1/CYBB/NCF2/TLR2/MMP9/PPARG/TLR6/VLDLR/IL6/CCL5/RXRG/HSPA1A/LBP/TLR4/JUN | 37 | 0.0586 |
| hsa04062 | Chemokine signaling pathway | 30/631 | 192/8159 | 0.000151 | 0.007004 | 0.006326 | GNGT2/CXCL6/CXCL1/CCL18/GNG7/VAV1/ARRB2/PIK3CD/CCR3/CXCL3/PTK2B/CXCR4/PREX1/PLCG2/PARD3/CXCR1/GRK5/CXCL2/TIAM1/PRKCB/FGR/ITK/CCL13/CXCL13/PF4V1/GSK3A/PIK3CG/CCL5/CCL11/CXCL12 | 30 | 0.0475 |
| hsa04141 | Protein processing in endoplasmic reticulum | 27/631 | 171/8159 | 0.000269 | 0.00969 | 0.008752 | XBP1/SSR3/EDEM1/ERLEC1/PDIA4/TXNDC5/CRYAB/SSR4/HSPA1L/SEL1L/HSPA1B/RPN1/HSPA2/HERPUD1/UGGT1/DNAJC10/SSR2/UBE2J1/ERN1/PDIA6/SEC24A/DNAJC3/MAN1A1/DNAJB1/SVIP/HSPA1A/HSPA4L | 27 | 0.0428 |
| hsa05202 | Transcriptional misregulation in cancer | 26/631 | 193/8159 | 0.003691 | 0.049824 | 0.045 | MMP3/CD86/CDK14/CCND2/CD14/IGFBP3/EWSR1/FLT3/MYC/BCL2A1/GZMB/MEIS1/BIRC3/FUT8/BCL2L1/WNT16/ATM/MMP9/PPARG/IL6/JUP/RXRG/RARA/CEBPE/MPO/ID2 | 26 | 0.0412 |
| hsa04514 | Cell adhesion molecules | 25/631 | 157/8159 | 0.000394 | 0.010636 | 0.009606 | CTLA4/CD226/ITGA4/CD86/CD80/SELL/CNTN1/SLITRK6/OCLN/CLDN22/ICAM2/SELP/PECAM1/SELPLG/PTPRF/ITGA8/CLDN17/CLDN3/SIGLEC1/CLDN10/ITGB8/PTPRC/NRXN1/F11R/CDH1 | 25 | 0.0396 |
| hsa05152 | Tuberculosis | 25/631 | 180/8159 | 0.002901 | 0.042717 | 0.038582 | ITGAX/IRAK2/CALML5/CR1L/CTSS/TCIRG1/CD14/CASP10/MAPK13/C3/IL1B/CR1/TLR1/CLEC4M/CLEC4E/TLR2/TLR9/TLR6/IL6/CYP27B1/CAMP/CD74/FCER1G/LBP/TLR4 | 25 | 0.0396 |
| hsa04670 | Leukocyte transendothelial migration | 24/631 | 114/8159 | 4.83E-06 | 0.000304 | 0.000275 | CYBA/RHOH/ITGA4/VAV1/NCF4/SIPA1/PIK3CD/PTK2B/CXCR4/MAPK13/PLCG2/OCLN/CLDN22/PECAM1/PRKCB/ITK/CYBB/CLDN17/NCF2/MMP9/CLDN3/CLDN10/CXCL12/F11R | 24 | 0.038 |
| hsa05323 | Rheumatoid arthritis | 22/631 | 93/8159 | 1.56E-06 | 0.000168 | 0.000152 | CTLA4/CXCL6/MMP3/CXCL1/MMP1/CD86/TCIRG1/CD80/CXCL3/TNFSF11/IL1B/LTB/IL17A/CXCL2/TLR2/CTSL/ATP6V1C2/IL6/CCL5/CXCL12/TLR4/JUN | 22 | 0.0349 |
| hsa04061 | Viral protein interaction with cytokine and cytokine receptor | 22/631 | 100/8159 | 5.63E-06 | 0.000304 | 0.000275 | CXCL6/CXCL1/IL24/CCL18/CCR3/CXCL3/CXCR4/IL2RG/CXCR1/ACKR4/CXCL2/IL2RA/CCL13/CXCL13/PF4V1/IL20RA/IL6/CCL5/IL20RB/CCL11/CXCL12/IL37 | 22 | 0.0349 |
| hsa05162 | Measles | 21/631 | 139/8159 | 0.002204 | 0.0357 | 0.032244 | SLAMF1/CD3D/HSPA1L/CCND2/PIK3CD/CD3G/HSPA1B/HSPA2/IL1B/IL2RG/STAT5A/BCL2L1/CLEC4M/IL2RA/TLR2/TLR9/MX1/IL6/HSPA1A/TLR4/JUN | 21 | 0.0333 |
| hsa04640 | Hematopoietic cell lineage | 19/631 | 99/8159 | 0.000173 | 0.007004 | 0.006326 | CD19/CD3D/CR2/CR1L/ITGA4/CD38/CD14/CD37/FLT3/CD3G/IL1B/CR1/MS4A1/IL7R/DNTT/IL2RA/CD1E/IL6/CD9 | 19 | 0.0301 |
| hsa04064 | NF-kappa B signaling pathway | 19/631 | 104/8159 | 0.000336 | 0.009906 | 0.008947 | CXCL1/CD14/CXCL3/LY96/EDA2R/TNFSF11/IL1B/PLCG2/BCL2A1/LTB/BIRC3/CXCL2/PRKCB/BCL2L1/CCL13/ATM/LBP/CXCL12/TLR4 | 19 | 0.0301 |
| hsa05146 | Amoebiasis | 18/631 | 102/8159 | 0.000736 | 0.017032 | 0.015383 | LAMB4/COL4A4/CXCL1/COL4A6/COL4A2/CD14/PIK3CD/CXCL3/IL1B/SERPINB10/CXCL2/PRKCB/TLR2/CD1E/IL6/TLR4/SERPINB4/SERPINB13 | 18 | 0.0285 |
| hsa05134 | Legionellosis | 17/631 | 57/8159 | 7.60E-07 | 0.000168 | 0.000152 | CXCL1/NLRC4/CR1L/HSPA1L/CD14/CXCL3/HSPA1B/C3/HSPA2/IL1B/CR1/CXCL2/TLR2/IL6/EEF1A2/HSPA1A/TLR4 | 17 | 0.0269 |
| hsa05150 | Staphylococcus aureus infection | 17/631 | 96/8159 | 0.000983 | 0.021236 | 0.01918 | KRT10/C3/C1QB/KRT27/SELP/KRT18/KRT33A/C1QA/FPR1/SELPLG/FPR2/C3AR1/CAMP/FCAR/KRT16/KRT13/KRT24 | 17 | 0.0269 |
| hsa05142 | Chagas disease | 17/631 | 102/8159 | 0.001959 | 0.0357 | 0.032244 | CD3D/PIK3CD/PPP2R2C/CD3G/CD247/MAPK13/C3/C1QB/IL1B/C1QA/TLR2/TLR9/TLR6/IL6/CCL5/TLR4/JUN | 17 | 0.0269 |
| hsa04620 | Toll-like receptor signaling pathway | 17/631 | 104/8159 | 0.002427 | 0.037443 | 0.033818 | CD86/CD80/CD14/PIK3CD/LY96/MAPK13/IL1B/TLR1/TLR2/TLR9/TLR6/IL6/CCL5/TLR3/LBP/TLR4/JUN | 17 | 0.0269 |
| hsa04659 | Th17 cell differentiation | 17/631 | 108/8159 | 0.003644 | 0.049824 | 0.045 | IRF4/CD3D/IL17F/CD3G/CD247/MAPK13/IL1B/RORC/IL2RG/IL17A/STAT5A/IL2RA/IL21/IL6/RXRG/RARA/JUN | 17 | 0.0269 |
| hsa04657 | IL-17 signaling pathway | 16/631 | 94/8159 | 0.002104 | 0.0357 | 0.032244 | CXCL6/MMP3/CXCL1/MMP1/IL17F/CXCL3/MAPK13/IL1B/IL17A/CXCL2/MMP9/IL6/MMP13/IL17D/CCL11/JUN | 16 | 0.0254 |
| hsa05140 | Leishmaniasis | 15/631 | 77/8159 | 0.000693 | 0.017032 | 0.015383 | CYBA/CR1L/ITGA4/NCF4/MAPK13/C3/IL1B/CR1/PRKCB/CYBB/NCF2/TLR2/EEF1A2/TLR4/JUN | 15 | 0.0238 |
| hsa04662 | B cell receptor signaling pathway | 15/631 | 82/8159 | 0.001366 | 0.027658 | 0.02498 | CD19/LILRB1/CR2/LILRA1/CD79A/LILRB3/VAV1/PIK3CD/LILRA5/PLCG2/LILRB2/LILRA6/LILRA2/PRKCB/JUN | 15 | 0.0238 |
| hsa05144 | Malaria | 12/631 | 50/8159 | 0.000325 | 0.009906 | 0.008947 | CR1L/KLRB1/GYPC/IL1B/CR1/SELP/PECAM1/TLR2/TLR9/COMP/IL6/TLR4 | 12 | 0.019 |
| hsa05340 | Primary immunodeficiency | 9/631 | 38/8159 | 0.001986 | 0.0357 | 0.032244 | CD19/IGLL1/CD3D/CD79A/IL2RG/AIRE/IL7R/ADA/PTPRC | 9 | 0.0143 |
