# Supplementary material for: Bioinformatics investigation of adaptive immune‐related genes in peri‐implantitis and periodontitis: Characteristics and diagnostic values
Source: Immun Inflamm Dis. 2024 May 23;12(5):e1272. doi: 10.1002/iid3.1272 (PMC11112631; doi:10.1002/iid3.1272)
Supplement: Supplementary file 5 — Supporting information. [file IID3-12-e1272-s001.docx]

**Supplementary Table 5. GO enrichment analysis of** **differential expression genes in P vs HP groups**

| **ONTOLOGY** | **ID** | **Description** | **GeneRatio** | **BgRatio** | **pvalue** | **p.adjust** | **qvalue** | **geneID** | **Count** |
| --- | --- | --- | --- | --- | --- | --- | --- | --- | --- |
| BP | GO:0002764 | immune response-regulating signaling pathway | 79/1200 | 468/18723 | 1.61E-15 | 8.57E-12 | 7.12E-12 | CTSS/CD19/MYO1G/CD226/LILRB2/CR2/LAT2/CD38/LY96/IRF4/KLHL6/PLCG2/VAV1/BANK1/BTLA/CD79A/STAP1/WNK1/TLR10/MFHAS1/CYBA/MAP3K1/PLEKHA1/SKAP1/CTLA4/TREM2/LYN/CD247/FCRL3/PRKCQ/IGLL1/PTPRC/LGR4/TLR2/BTK/CD14/BIRC3/SCIMP/LILRB1/PRKCB/KCNN4/VAV3/TLR1/FGR/FFAR2/TRAT1/THEMIS2/MS4A1/FCN1/LPXN/F2RL1/ITK/PAK3/NCKAP1L/LAX1/CR1/HSPA1B/LCK/RAB11FIP2/ARRB2/TYROBP/CLEC4E/BTN3A1/MNDA/LTF/CLEC6A/C3AR1/HSPA1A/NLRP6/NFATC2/S100A14/PIK3CD/IRAK2/FPR1/TNIP3/ADA/PDE4B/LILRA2/BTN1A1 | 79 |
| BP | GO:1903131 | mononuclear cell differentiation | 73/1200 | 426/18723 | 8.90E-15 | 2.36E-11 | 1.97E-11 | CD19/LILRB2/CR2/POU2AF1/CD3D/IRF4/XBP1/PLCG2/CD86/VAV1/IKZF3/CD79A/SLAMF1/BATF/ITGA4/TMEM176B/CTNNBIP1/CD3G/IL21/MYC/FLT3/FUT7/ST3GAL1/PREX1/RHOH/CTLA4/TREM2/LYN/FCRL3/PTPRC/PTK2B/GPR183/DNAJB9/BTK/IL1B/PSMB11/LILRB1/TCIRG1/GPR18/PCK1/PIK3R1/MS4A1/F2RL1/ITK/NCKAP1L/CR1/IL7R/CTSL/CD80/PPARG/EGR3/LCK/FZD5/PLA2G2D/RORC/VNN1/IL2RA/ADAM8/BMP4/CD40LG/CD46/PIK3CD/MERTK/MT1G/PIR/TMEM176A/EOMES/NRARP/ADA/TNFSF8/IL31RA/KLF6/TOX | 73 |
| BP | GO:0001819 | positive regulation of cytokine production | 73/1200 | 467/18723 | 1.04E-12 | 1.11E-09 | 9.19E-10 | CD226/LILRB2/POU2AF1/LY96/IRF4/XBP1/PLCG2/CD86/NLRC4/IL26/SLAMF1/BATF/CYBA/SULF2/AIF1/IL21/CD200/TREM2/CLECL1/CYBB/AIM2/PRKCQ/PTPRC/TLR2/IL1B/CD14/LTB/SCIMP/LILRB1/IL17F/TLR1/P2RX7/FGR/FFAR2/PIK3R1/FCN1/LILRA5/F2RL1/ITK/SCAMP5/CD80/PANX2/PIK3CG/HSPA1B/IL17A/HMHB1/C3/TYROBP/SAA1/ALOX15B/BTN3A1/SEMA7A/FZD5/MNDA/CLEC6A/C3AR1/ADAM8/HTR2B/HSPA1A/CD40LG/IL12RB2/CD46/SLC7A5/PIK3CD/FERMT1/MMP12/SERPINB7/PLA2G3/PAEP/WNT11/ADRA2A/PDE4B/LILRA2 | 73 |
| BP | GO:0002253 | activation of immune response | 66/1200 | 375/18723 | 4.83E-14 | 8.55E-11 | 7.10E-11 | CD19/MYO1G/CD226/CR2/LAT2/CD38/KLHL6/PLCG2/VAV1/BANK1/CD79A/NLRC4/STAP1/KRT1/WNK1/ZBP1/CR1L/PLEKHA1/SUSD4/SKAP1/CTLA4/TREM2/LYN/CD247/AIM2/FCRL3/IGLL1/PTPRC/BTK/IL1B/PRKCB/KCNN4/FCN2/VAV3/CFH/FGR/FFAR2/TRAT1/THEMIS2/MS4A1/FCN1/LPXN/ITK/PAK3/NCKAP1L/LAX1/CR1/C3/LCK/FCN3/TYROBP/BTN3A1/MNDA/CLEC6A/C3AR1/NFATC2/CD46/PIK3CD/C1QA/FPR1/MBL2/C1QB/ADA/PDE4B/LILRA2/BTN1A1 | 66 |
| BP | GO:0050900 | leukocyte migration | 63/1200 | 369/18723 | 7.53E-13 | 9.99E-10 | 8.31E-10 | MYO1G/VAV1/STAP1/WNK1/SLAMF1/STK39/ITGA4/TRIM55/AIF1/FUT7/PREX1/RHOH/CD200/SELL/TREM2/PECAM1/CXCR4/LYN/CHST2/PTK2B/DPEP1/GPR183/CXCL1/P2RX4/CXADR/SELP/CXCL3/VAV3/FFAR2/GPR18/MMP9/CXCL2/F2RL1/NCKAP1L/C10orf99/ADTRP/TREM1/CCL5/PIK3CG/IL17A/CXCL6/LCK/SAA1/CCL18/PLA2G7/PTN/C3AR1/ADAM8/SCG2/CXCL13/MIA3/S100A14/PIK3CD/TNFSF11/RET/ADA/CCL13/PDE4B/CXCR1/EDN2/PF4V1/BMP5/ECM1 | 63 |
| BP | GO:0019221 | cytokine-mediated signaling pathway | 62/1200 | 472/18723 | 5.55E-08 | 9.51E-06 | 7.91E-06 | TNFRSF17/LILRB2/STAP1/WNK1/ZBP1/STK39/MST1R/KRT8/KRT18/FLT3/LILRB3/TREM2/CXCR4/AIM2/TNFRSF19/GHR/PTPRC/PTK2B/DOK1/CXCL1/IL1B/BIRC3/MPL/LILRB1/CXCL3/IL17F/EDA2R/CXCL2/LILRA5/LILRA6/F2RL1/IL7R/CCL5/LILRA1/IL22RA2/PPARG/HSPA1B/IL17A/CXCL6/IFI27/CCL18/IL2RA/SHARPIN/HSPA1A/CXCL13/IL2RG/IL12RB2/MX1/CCR3/IRAK2/MMP12/TNFSF11/IL13RA2/IL31RA/CCL13/CXCR1/LILRA2/EDN2/PF4V1/ECM1/STAT5A/CRLF1 | 62 |
| BP | GO:0042110 | T cell activation | 62/1200 | 487/18723 | 1.73E-07 | 2.19E-05 | 1.82E-05 | SLAMF7/LILRB2/CD3D/IRF4/XBP1/CD86/VAV1/SLAMF1/BATF/RASAL3/GPNMB/AIF1/CD3G/IL21/FUT7/PREX1/RHOH/CTLA4/CLECL1/LYN/PRKCQ/PTPRC/GPR183/ICOS/IL1B/PSMB11/CXADR/LILRB1/TCIRG1/GPR18/PCK1/F2RL1/ITK/NCKAP1L/LAX1/CR1/IL7R/CTSL/CD80/CCL5/PIK3CG/EGR3/LCK/BTN3A1/FZD5/PLA2G2D/RORC/VNN1/IL2RA/ADAM8/BMP4/CD40LG/CD8B/CD46/PIK3CD/DLG1/TNFSF11/EOMES/NRARP/ADA/TNFSF8/TOX | 62 |
| CC | GO:0005911 | cell-cell junction | 62/1263 | 494/19550 | 3.84E-07 | 0.000123 | 0.00011 | DSP/VAV1/CD53/SH3KBP1/AMOTL1/KRT8/VANGL2/DSC2/PKP3/JAG1/DSC3/TRIM29/KRT18/SLC2A1/CLDN10/SKAP1/WNK3/PECAM1/TRPC4/LYN/PERP/JUP/POF1B/PODXL/GJB5/AKR1B1/PKP1/TIAM1/KCNA2/CXADR/PARD3/MAGI1/AQP3/PIK3R1/DSG3/CLDN3/ITK/KCNA5/NHS/GJA1/PANX2/KCNJ2/FGFR4/GJA3/GRHL2/CDC42BPA/MARVELD2/ALOX15B/OCLN/DNMBP/FZD5/PARD6A/CDH1/SSX2IP/FLRT3/EPCAM/DLG1/AJUBA/CDH18/PPL/SHROOM1/CLDN9 | 62 |
| BP | GO:0030098 | lymphocyte differentiation | 61/1200 | 374/18723 | 1.30E-11 | 9.84E-09 | 8.18E-09 | CD19/LILRB2/CR2/POU2AF1/CD3D/IRF4/XBP1/PLCG2/CD86/VAV1/IKZF3/CD79A/SLAMF1/BATF/ITGA4/CD3G/IL21/FLT3/FUT7/ST3GAL1/PREX1/RHOH/CTLA4/FCRL3/PTPRC/PTK2B/GPR183/DNAJB9/BTK/IL1B/PSMB11/TCIRG1/GPR18/PCK1/PIK3R1/MS4A1/ITK/NCKAP1L/CR1/IL7R/CTSL/CD80/EGR3/LCK/FZD5/PLA2G2D/RORC/VNN1/IL2RA/ADAM8/BMP4/CD40LG/CD46/PIK3CD/MERTK/EOMES/NRARP/ADA/TNFSF8/KLF6/TOX | 61 |
| BP | GO:0032103 | positive regulation of response to external stimulus | 61/1200 | 427/18723 | 3.01E-09 | 9.41E-07 | 7.82E-07 | CD226/LY96/PLCG2/VAV1/CD180/NLRC4/STAP1/WNK1/ZBP1/TLR10/SLAMF1/STK39/CYBA/AIF1/IL21/ALOX5AP/TREM2/CXCR4/LYN/AIM2/PTK2B/TLR2/BTK/TIAM1/IL1B/P2RX4/SCIMP/IL17F/LY86/FFAR2/FCN1/LILRA5/F2RL1/PAK3/NCKAP1L/FABP4/CCL5/PIK3CG/IL17A/C3/TYROBP/PLA2G7/MNDA/CLEC6A/PTN/C3AR1/ADAM8/NTF3/SCG2/CXCL13/S100A14/KLK7/PGC/MMP12/TNFSF11/PLA2G3/AGTR1/LILRA2/EDN2/TGM2/F2 | 61 |
| BP | GO:0002683 | negative regulation of immune system process | 60/1200 | 434/18723 | 1.41E-08 | 3.25E-06 | 2.70E-06 | SAMSN1/LILRB2/CR2/CD86/BANK1/STAP1/SLAMF1/CR1L/GPNMB/TMEM176B/SUSD4/MYC/LILRB3/CD200/CTLA4/TREM2/CST7/LYN/FCRL3/PTPRC/BTK/LILRB1/FGR/GPR18/PIK3R1/TGFB3/LPXN/TARBP2/FBN1/NCKAP1L/LAX1/ADTRP/CR1/IL7R/CD80/PPARG/ARRB2/TYROBP/LRFN5/MILR1/MNDA/PLA2G2D/LTF/IL2RA/BMP4/NLRP6/MIA3/CD46/MERTK/DLG1/MMP12/TMEM176A/PLK2/IL13RA2/NRARP/TAPBPL/ADA/IL31RA/SOX11/BMP5 | 60 |
| BP | GO:0022407 | regulation of cell-cell adhesion | 60/1200 | 448/18723 | 4.53E-08 | 8.29E-06 | 6.89E-06 | LILRB2/XBP1/CD86/VAV1/WNK1/SLAMF1/AKNA/RASAL3/GPNMB/ITGA4/AIF1/JAG1/IL21/FUT7/RHOH/SKAP1/CTLA4/CLECL1/SOX2/LYN/CHST2/PRKCQ/PTPRC/PODXL/ICOS/EFNA5/ZDHHC2/IL1B/EPHB3/LILRB1/SELP/MAGI1/PCK1/NCKAP1L/LAX1/ADTRP/CR1/IL7R/CD80/CCL5/EGR3/LCK/PLA2G2D/VNN1/IL2RA/ADAM8/BMP4/CDH1/CXCL13/CD40LG/MIA3/CD46/EPCAM/DLG1/TNFSF11/MEGF10/NRARP/ADA/HAS2/KLF4 | 60 |
| MF | GO:0030546 | signaling receptor activator activity | 56/1229 | 495/18368 | 7.94E-05 | 0.005208 | 0.0047 | PNOC/CCK/GMFG/IL26/IL24/GPNMB/JAG1/IL21/NPPC/EFNA5/CXCL1/IL1B/LTB/CXCL3/IL17F/WNT2B/WNT3/CXCL2/TGFB3/EGF/FBN1/C10orf99/CCL5/SLURP1/IL17A/FAM3B/CXCL6/FGF19/CCL18/SEMA7A/PTN/CMTM2/NTF3/BMP4/SCG2/CXCL13/CD40LG/GDF6/MIA/FLRT3/UTS2/TNFSF11/WNT11/TNFSF8/WNT16/TIMP1/CCL13/TFF1/EDN2/PF4V1/BMP5/F2/SST/PMCH/CGA/CRLF1 | 56 |
| BP | GO:0002768 | immune response-regulating cell surface receptor signaling pathway | 55/1200 | 315/18723 | 8.89E-12 | 7.87E-09 | 6.54E-09 | CD19/MYO1G/CD226/LILRB2/CR2/LAT2/CD38/KLHL6/PLCG2/VAV1/BANK1/BTLA/CD79A/STAP1/WNK1/MAP3K1/PLEKHA1/SKAP1/CTLA4/LYN/CD247/FCRL3/PRKCQ/IGLL1/PTPRC/BTK/LILRB1/PRKCB/KCNN4/VAV3/FGR/FFAR2/TRAT1/THEMIS2/MS4A1/FCN1/LPXN/ITK/PAK3/NCKAP1L/LAX1/CR1/LCK/TYROBP/BTN3A1/MNDA/CLEC6A/C3AR1/NFATC2/PIK3CD/FPR1/ADA/PDE4B/LILRA2/BTN1A1 | 55 |
| BP | GO:0045785 | positive regulation of cell adhesion | 55/1200 | 437/18723 | 1.23E-06 | 0.00012 | 9.95E-05 | LILRB2/XBP1/CD86/VAV1/SLAMF1/RASAL3/ITGA4/AIF1/IL21/NPY2R/FUT7/PREX1/RHOH/SKAP1/NID1/CLECL1/SOX2/LYN/CHST2/JUP/PRKCQ/PTPRC/PTK2B/PODXL/ICOS/IL1B/LILRB1/SELP/VIT/MAGI1/VAV3/PCK1/NCKAP1L/CR1/IL7R/CD80/CCL5/EGR3/LCK/SAA1/PTN/AGR2/VNN1/IL2RA/ADAM8/CXCL13/CD40LG/CD46/FERMT1/TNFSF11/MEGF10/RET/ADA/HAS2/TGM2 | 55 |
| BP | GO:0072503 | cellular divalent inorganic cation homeostasis | 55/1200 | 486/18723 | 2.88E-05 | 0.001404 | 0.001167 | CD19/JSRP1/CD38/PLCG2/P2RY1/CYBA/P2RY10/NPY2R/GPR65/P2RX5/CXCR4/TRPC4/LYN/PTPRC/PTK2B/SLC39A8/GPR174/P2RX4/HERPUD1/PRKCB/P2RX1/TCIRG1/P2RX7/GPR18/MT1X/MS4A1/CD52/F2RL1/ANXA6/KCNA5/GJA1/CCL5/SLC39A7/PIK3CG/LCK/SAA1/PROK2/CACNA1A/LPAR6/C3AR1/HTR2B/CXCL13/TRPA1/MT1G/CCR3/FPR1/KNG1/PTGIR/AGTR1/CCL13/CXCR1/EDN2/TGM2/F2/CNGB1 | 55 |
| MF | GO:0048018 | receptor ligand activity | 55/1229 | 487/18368 | 9.60E-05 | 0.005508 | 0.00497 | PNOC/CCK/GMFG/IL26/IL24/GPNMB/JAG1/IL21/NPPC/CXCL1/IL1B/LTB/CXCL3/IL17F/WNT2B/WNT3/CXCL2/TGFB3/EGF/FBN1/C10orf99/CCL5/SLURP1/IL17A/FAM3B/CXCL6/FGF19/CCL18/SEMA7A/PTN/CMTM2/NTF3/BMP4/SCG2/CXCL13/CD40LG/GDF6/MIA/FLRT3/UTS2/TNFSF11/WNT11/TNFSF8/WNT16/TIMP1/CCL13/TFF1/EDN2/PF4V1/BMP5/F2/SST/PMCH/CGA/CRLF1 | 55 |
| BP | GO:0070661 | leukocyte proliferation | 54/1200 | 318/18723 | 4.14E-11 | 2.75E-08 | 2.28E-08 | CD19/MZB1/LILRB2/CR2/CD38/CD86/CD180/IKZF3/CD79A/SLAMF1/NPR3/RASAL3/GPNMB/AIF1/IL21/FLT3/CTLA4/TREM2/CLECL1/LYN/FCRL3/PRKCQ/PTPRC/GPR183/BTK/OCSTAMP/IL1B/MPL/LILRB1/VAV3/TCIRG1/MS4A1/F2RL1/NCKAP1L/CR1/IL7R/CD80/CCL5/PIK3CG/GAPT/TYROBP/BTN3A1/MNDA/PLA2G2D/IL2RA/BMP4/CD40LG/NFATC2/CD46/DLG1/TNFSF11/ADA/TNFSF8/SOX11 | 54 |
| BP | GO:1903706 | regulation of hemopoiesis | 54/1200 | 367/18723 | 8.68E-09 | 2.40E-06 | 2.00E-06 | HCLS1/LILRB2/IRF4/XBP1/CD86/RASSF2/IKZF3/JAG1/TMEM176B/CTNNBIP1/MYC/MEIS1/RHOH/LILRB3/SCIN/CTLA4/TREM2/LYN/FCRL3/PTPRC/PTK2B/BTK/OCSTAMP/MPL/LILRB1/PCK1/PIK3R1/FBN1/NCKAP1L/CR1/IL7R/CD80/PITHD1/HSPA1B/EGR3/IL17A/TESC/TYROBP/LTF/PTN/VNN1/IL2RA/ADAM8/BMP4/HSPA1A/ACVR1B/CD46/MITF/TMEM176A/TNFSF11/PLA2G3/NRARP/ADA/TOX | 54 |
| BP | GO:0050867 | positive regulation of cell activation | 54/1200 | 420/18723 | 7.90E-07 | 8.39E-05 | 6.97E-05 | CD226/LILRB2/CD38/XBP1/CD86/VAV1/STAP1/SH3KBP1/SLAMF1/RASAL3/AIF1/IL21/CLEC4D/PLEK/RHOH/TREM2/CLECL1/LYN/FCRL3/PRKCQ/IGLL1/PTPRC/GPR183/BTK/ICOS/IL1B/MPL/LILRB1/SELP/VAV3/FGR/PCK1/LILRA5/F2RL1/NCKAP1L/SPACA3/CR1/IL7R/CD80/CCL5/EGR3/LCK/TYROBP/VNN1/IL2RA/ADAM8/CD40LG/NFATC2/CD46/TNFSF11/PLA2G3/ADA/LILRA2/TOX | 54 |
| BP | GO:0002443 | leukocyte mediated immunity | 54/1200 | 440/18723 | 3.30E-06 | 0.000262 | 0.000218 | CD19/SLAMF7/MYO1G/CD226/CR2/LAT2/PLCG2/VAV1/ARL8B/STAP1/SLAMF1/BATF/CR1L/JAG1/SUSD4/IL21/FUT7/TREM2/LYN/IGLL1/PTPRC/PRF1/BTK/IL1B/SCIMP/LILRB1/TCIRG1/FGR/F2RL1/NCKAP1L/CR1/IL7R/TREM1/PIK3CG/C3/CXCL6/GAPT/ARRB2/TYROBP/MILR1/FZD5/GZMB/NLRP6/CD40LG/CD46/PIK3CD/C1QA/PLA2G3/IL13RA2/MBL2/C1QB/AZGP1/CD1E/F2 | 54 |
| BP | GO:0050727 | regulation of inflammatory response | 53/1200 | 386/18723 | 1.23E-07 | 1.76E-05 | 1.46E-05 | MMP3/TNFAIP6/PLCG2/STAP1/KRT1/ZBP1/TLR10/STK39/AKNA/MFHAS1/IL21/FUT7/ALOX5AP/CD200/TREM2/CST7/LYN/PTPRC/F12/TLR2/BTK/IL1B/BIRC3/FGR/FFAR2/MMP9/LILRA5/FABP4/CCL5/IL22RA2/PPARG/PIK3CG/C3/SAA1/LRFN5/GPR31/PLA2G7/SEMA7A/PLA2G2D/IL2RA/SHARPIN/ADAM8/NLRP6/NR1D2/ACE2/PLK2/TNFSF11/PLA2G3/ADA/AGTR1/SIGLEC10/KLF4/TGM2 | 53 |
| BP | GO:0055074 | calcium ion homeostasis | 53/1200 | 460/18723 | 2.46E-05 | 0.001267 | 0.001053 | CD19/JSRP1/CD38/PLCG2/P2RY1/CYBA/P2RY10/NPY2R/GPR65/P2RX5/CXCR4/TRPC4/LYN/PTPRC/PTK2B/GPR174/P2RX4/HERPUD1/PRKCB/P2RX1/TCIRG1/P2RX7/GPR18/MS4A1/CD52/F2RL1/ANXA6/KCNA5/GJA1/CCL5/PIK3CG/LCK/SAA1/PROK2/CACNA1A/LPAR6/C3AR1/HTR2B/CXCL13/S100A14/TRPA1/CCR3/FPR1/KNG1/TNFSF11/PTGIR/AGTR1/CCL13/CXCR1/EDN2/TGM2/F2/CNGB1 | 53 |
| BP | GO:0009410 | response to xenobiotic stimulus | 53/1200 | 462/18723 | 2.77E-05 | 0.001361 | 0.001131 | GSTA4/CD38/CYBA/FBP1/AADAC/GSTA5/MYC/SLC6A4/CYBB/CXCR4/LYN/AIM2/PTK2B/DPEP1/NPPC/IL1B/ENG/CYP2W1/VAV3/NCKAP1L/ACSM1/LCK/BCHE/CYP2C18/AK4/CYP2J2/AHRR/CYP3A7/CYP3A5/RORC/PTN/GCLC/HTR2B/CDH1/CYP4F12/STAR/NFATC2/TRPA1/GSTA1/ANKRD1/SLC22A12/RET/ADA/PDE4B/SULT2A1/GSTA2/GAD2/UGT2B15/HMGCS1/FMO2/SST/GAD1/UGT2B28 | 53 |
| BP | GO:0043410 | positive regulation of MAPK cascade | 53/1200 | 480/18723 | 7.69E-05 | 0.002857 | 0.002374 | PLCG2/RASSF2/BANK1/MYDGF/IL26/SLAMF1/STK39/MST1R/MFHAS1/P2RY1/GPNMB/ERN1/FLT3/TBX1/TREM2/SOX2/FCRL3/TNFRSF19/GHR/ADRB2/PTPRC/PTK2B/GPR183/TIAM1/IL1B/SCIMP/EDA2R/LILRA5/F2RL1/EGF/CCL5/PIK3CG/FGFR4/FGF19/ARRB2/CCL18/FGFR2/SEMA7A/FZD5/ADAM8/NTF3/BMP4/HTR2B/GDF6/AJUBA/MARCO/TNFSF11/RET/WNT16/ADRA2A/CCL13/NTRK2/FZD10 | 53 |
| BP | GO:0007159 | leukocyte cell-cell adhesion | 52/1200 | 371/18723 | 8.29E-08 | 1.29E-05 | 1.08E-05 | LILRB2/XBP1/CD86/VAV1/WNK1/SLAMF1/RASAL3/GPNMB/ITGA4/AIF1/IL21/FUT7/RHOH/SKAP1/SELL/CTLA4/CLECL1/PECAM1/LYN/CHST2/PRKCQ/PTPRC/SLC39A8/ICOS/IL1B/LILRB1/SELP/PCK1/NCKAP1L/LAX1/ADTRP/CR1/IL7R/CD80/CCL5/CLEC4M/EGR3/LCK/PLA2G2D/VNN1/IL2RA/ADAM8/BMP4/CD40LG/MIA3/CD46/DLG1/TNFSF11/NRARP/ADA/HAS2/KLF4 | 52 |
| BP | GO:0050878 | regulation of body fluid levels | 52/1200 | 379/18723 | 1.66E-07 | 2.15E-05 | 1.78E-05 | XBP1/PLCG2/VAV1/KRT16/KRT1/WNK1/NPR3/STK39/P2RY1/CYBA/PLEK/F5/LYN/GRHL3/PRKCQ/F12/AKR1B1/MPL/GP1BA/SELP/KCNN4/TP63/P2RX1/VAV3/AQP3/F2RL1/ENPP4/GJA1/ADTRP/PIK3CG/LCK/SAA1/AGR2/CSN3/NLRP6/CYP4F12/CD40LG/MERTK/STATH/SLC7A11/KNG1/CYP4F11/TSPAN8/ADA/HAS2/TFPI2/ADRA2A/PF4V1/NEUROG1/F2/STAT5A/MLLT6 | 52 |
| BP | GO:0052547 | regulation of peptidase activity | 52/1200 | 461/18723 | 5.14E-05 | 0.002169 | 0.001802 | SERPINI1/CCK/MAPT/APH1B/NLRC4/MYC/SOX2/CST7/LYN/AIM2/PERP/DPEP1/BIRC3/HERPUD1/TP63/COL4A3/P2RX1/RARRES1/CRYAB/MMP9/CLDN3/CASP10/CR1/SERPINB5/PPARG/A2ML1/C3/LCK/SERPINA11/ARRB2/DNAJB6/LTF/SERPINB13/NLRP6/EFNA3/SERPINB11/KNG1/SPINK1/SERPINB7/SPOCK1/SERPINI2/TFPI2/TIMP1/PRSS22/KLF4/FOXL2/SMR3B/SPINK7/CIDEB/ECM1/SPINK9/SIAH2 | 52 |
| BP | GO:0008544 | epidermis development | 51/1200 | 324/18723 | 2.22E-09 | 7.39E-07 | 6.14E-07 | DSP/SOX21/KRT16/JAG1/ALDH3A2/IRF6/HES5/CRABP2/GRHL3/TNFRSF19/GJB5/LGR4/CALML5/DCT/TP63/AQP3/EDA2R/ACER1/KRT2/OVOL3/ESRP1/GRHL2/KRT27/EXPH5/ALOX15B/KRT85/SFRP4/FGFR2/SOX18/LIPK/SHARPIN/KRT10/BMP4/ACVR1B/KLK7/FERMT1/KRT31/KRT34/ERCC3/WNT16/PPL/KRT76/EMP1/LIPM/KLF4/KRT25/LCE3B/SPRR1A/TGM3/LAMC2/TCHH | 51 |
| BP | GO:0042060 | wound healing | 51/1200 | 422/18723 | 9.48E-06 | 0.000607 | 0.000504 | DSP/XBP1/PLCG2/VAV1/ARL8B/KRT1/IL24/P2RY1/PLEK/CXCR4/F5/LYN/GRHL3/PRKCQ/F12/MPL/GP1BA/SELP/P2RX1/VAV3/TGFB3/CLDN3/KANK1/F2RL1/ANXA6/ENPP4/GJA1/ADTRP/PIK3CG/LCK/SAA1/OCLN/FGFR2/NLRP6/CD40LG/MIA3/MERTK/AJUBA/SLC7A11/FERMT1/KNG1/MMP12/CYP4F11/TSPAN8/TFPI2/TIMP1/ADRA2A/PPL/PF4V1/F2/ODAM | 51 |
| BP | GO:0006874 | cellular calcium ion homeostasis | 51/1200 | 448/18723 | 4.77E-05 | 0.002059 | 0.001712 | CD19/JSRP1/CD38/PLCG2/P2RY1/CYBA/P2RY10/NPY2R/GPR65/P2RX5/CXCR4/TRPC4/LYN/PTPRC/PTK2B/GPR174/P2RX4/HERPUD1/PRKCB/P2RX1/TCIRG1/P2RX7/GPR18/MS4A1/CD52/F2RL1/ANXA6/KCNA5/GJA1/CCL5/PIK3CG/LCK/SAA1/PROK2/CACNA1A/LPAR6/C3AR1/HTR2B/CXCL13/TRPA1/CCR3/FPR1/KNG1/PTGIR/AGTR1/CCL13/CXCR1/EDN2/TGM2/F2/CNGB1 | 51 |
| CC | GO:0009897 | external side of plasma membrane | 51/1263 | 421/19550 | 1.11E-05 | 0.000622 | 0.00056 | CD19/SLAMF7/CD226/CD3D/CD86/CD79A/SLAMF1/CD3G/KRT18/CTLA4/ITGAX/CD163L1/CXCR4/IGLL1/GHR/PTPRC/EFNA5/ENG/CD14/GP1BA/LILRB1/SELP/P2RX1/MS4A1/FCN1/IL7R/FCRL6/NRCAM/CD80/IL17A/BTN3A1/SEMA7A/IL2RA/TNFRSF9/CLEC2D/CD40LG/IL2RG/IL12RB2/SLC7A5/CCR3/IL13RA2/ADA/AZGP1/IL31RA/TGFBR3/CD1E/CXCR1/LY6G5C/F2/BTN1A1/CRLF1 | 51 |
| BP | GO:0002429 | immune response-activating cell surface receptor signaling pathway | 50/1200 | 291/18723 | 1.42E-10 | 7.56E-08 | 6.28E-08 | CD19/MYO1G/CD226/CR2/LAT2/CD38/KLHL6/PLCG2/VAV1/BANK1/CD79A/STAP1/WNK1/PLEKHA1/SKAP1/CTLA4/LYN/CD247/FCRL3/IGLL1/PTPRC/BTK/PRKCB/KCNN4/VAV3/FGR/FFAR2/TRAT1/THEMIS2/MS4A1/FCN1/LPXN/ITK/PAK3/NCKAP1L/LAX1/CR1/LCK/TYROBP/BTN3A1/MNDA/CLEC6A/C3AR1/NFATC2/PIK3CD/FPR1/ADA/PDE4B/LILRA2/BTN1A1 | 50 |
| BP | GO:0002757 | immune response-activating signal transduction | 50/1200 | 291/18723 | 1.42E-10 | 7.56E-08 | 6.28E-08 | CD19/MYO1G/CD226/CR2/LAT2/CD38/KLHL6/PLCG2/VAV1/BANK1/CD79A/STAP1/WNK1/PLEKHA1/SKAP1/CTLA4/LYN/CD247/FCRL3/IGLL1/PTPRC/BTK/PRKCB/KCNN4/VAV3/FGR/FFAR2/TRAT1/THEMIS2/MS4A1/FCN1/LPXN/ITK/PAK3/NCKAP1L/LAX1/CR1/LCK/TYROBP/BTN3A1/MNDA/CLEC6A/C3AR1/NFATC2/PIK3CD/FPR1/ADA/PDE4B/LILRA2/BTN1A1 | 50 |
| BP | GO:0060326 | cell chemotaxis | 50/1200 | 310/18723 | 1.37E-09 | 4.85E-07 | 4.03E-07 | VAV1/STAP1/WNK1/SLAMF1/STK39/AIF1/PREX1/BIN2/CXCR4/LYN/PRKCQ/PTK2B/DPEP1/GPR183/CXCL1/TIAM1/P2RX4/CXADR/CXCL3/VAV3/FFAR2/GPR18/CXCL2/F2RL1/NCKAP1L/C10orf99/TREM1/CCL5/PIK3CG/EGR3/CXCL6/ARRB2/SAA1/CCL18/PLA2G7/PTN/C3AR1/ADAM8/SCG2/CXCL13/S100A14/PIK3CD/CCR3/TNFSF11/AGTR1/CCL13/PDE4B/CXCR1/EDN2/PF4V1 | 50 |
| BP | GO:0002697 | regulation of immune effector process | 50/1200 | 339/18723 | 2.85E-08 | 5.82E-06 | 4.84E-06 | MZB1/CD226/CR2/IRF4/XBP1/PLCG2/CD86/VAV1/STAP1/SLAMF1/CR1L/SUSD4/IL21/FUT7/TREM2/LYN/FCRL3/PTPRC/DNAJB9/BTK/IL1B/SCIMP/LILRB1/IL17F/CFH/FGR/FFAR2/PCK1/TGFB3/F2RL1/NCKAP1L/CR1/IL7R/CD80/IL17A/C3/CXCL6/ARRB2/TYROBP/SEMA7A/FZD5/CD40LG/CD46/SLC7A5/KLK7/PGC/PLA2G3/IL13RA2/AZGP1/CD1E | 50 |
| BP | GO:0002696 | positive regulation of leukocyte activation | 50/1200 | 409/18723 | 8.43E-06 | 0.00056 | 0.000465 | CD226/LILRB2/CD38/XBP1/CD86/VAV1/STAP1/SH3KBP1/SLAMF1/RASAL3/AIF1/IL21/CLEC4D/RHOH/TREM2/CLECL1/LYN/FCRL3/PRKCQ/IGLL1/PTPRC/GPR183/BTK/ICOS/IL1B/MPL/LILRB1/VAV3/FGR/PCK1/F2RL1/NCKAP1L/SPACA3/CR1/IL7R/CD80/CCL5/EGR3/LCK/TYROBP/VNN1/IL2RA/ADAM8/CD40LG/NFATC2/CD46/TNFSF11/PLA2G3/ADA/TOX | 50 |
| BP | GO:0050673 | epithelial cell proliferation | 50/1200 | 437/18723 | 4.95E-05 | 0.002104 | 0.001749 | EAF2/XBP1/MYDGF/IL26/CYBA/ITGA4/SULF2/ERN1/MYC/TBX1/IRF6/SOX2/HES5/LGR4/DLX5/TP63/COL4A3/KRT2/EHF/EGF/GJA1/SERPINB5/PPARG/SLURP1/EGR3/PROK2/FGFR2/FGFBP1/PTN/BMP4/SGPP2/HTR2B/SCG2/PIK3CD/DLG1/FERMT1/CCR3/MMP12/TNFSF11/NRARP/HAS2/AGTR1/WNT16/TGFBR3/SOX11/BMP5/TNMD/ODAM/ECM1/STAT5A | 50 |
| BP | GO:0052548 | regulation of endopeptidase activity | 49/1200 | 432/18723 | 7.30E-05 | 0.002849 | 0.002368 | SERPINI1/CCK/MAPT/APH1B/NLRC4/MYC/SOX2/CST7/LYN/AIM2/PERP/DPEP1/BIRC3/HERPUD1/TP63/COL4A3/P2RX1/RARRES1/CRYAB/MMP9/CASP10/CR1/SERPINB5/PPARG/A2ML1/C3/LCK/SERPINA11/ARRB2/DNAJB6/LTF/SERPINB13/NLRP6/EFNA3/SERPINB11/KNG1/SPINK1/SERPINB7/SPOCK1/SERPINI2/TFPI2/TIMP1/KLF4/FOXL2/SMR3B/SPINK7/CIDEB/SPINK9/SIAH2 | 49 |
| BP | GO:0003012 | muscle system process | 49/1200 | 452/18723 | 0.000219 | 0.006102 | 0.005071 | DSP/JSRP1/CD38/P2RY1/CYBA/SULF2/AIF1/DSC2/TNNT3/NPY2R/RCSD1/CXCR4/JUP/ADRB2/TIAM1/IL1B/P2RX4/KCNJ8/P2RX1/CRYAB/PPP1R13L/ANXA6/KCNA5/GJA1/KCNJ2/PIK3CG/TNNT1/CHRNA3/PPP1R12B/GATM/PROK2/STAC2/HTR2B/RGS4/ACE2/UTS2/DLG1/HTR7/ADA/ADRA2A/PDE4B/KLF4/EDN2/NMUR1/NEUROG1/MYBPC2/MYL1/RNF207/SLN | 49 |
| BP | GO:0046651 | lymphocyte proliferation | 48/1200 | 288/18723 | 9.58E-10 | 3.92E-07 | 3.25E-07 | CD19/MZB1/LILRB2/CR2/CD38/CD86/CD180/IKZF3/CD79A/SLAMF1/RASAL3/GPNMB/AIF1/IL21/FLT3/CTLA4/CLECL1/LYN/FCRL3/PRKCQ/PTPRC/GPR183/BTK/IL1B/MPL/LILRB1/VAV3/MS4A1/NCKAP1L/CR1/IL7R/CD80/CCL5/PIK3CG/GAPT/TYROBP/BTN3A1/MNDA/PLA2G2D/IL2RA/BMP4/CD40LG/NFATC2/CD46/DLG1/ADA/TNFSF8/SOX11 | 48 |
| BP | GO:0032943 | mononuclear cell proliferation | 48/1200 | 291/18723 | 1.37E-09 | 4.85E-07 | 4.03E-07 | CD19/MZB1/LILRB2/CR2/CD38/CD86/CD180/IKZF3/CD79A/SLAMF1/RASAL3/GPNMB/AIF1/IL21/FLT3/CTLA4/CLECL1/LYN/FCRL3/PRKCQ/PTPRC/GPR183/BTK/IL1B/MPL/LILRB1/VAV3/MS4A1/NCKAP1L/CR1/IL7R/CD80/CCL5/PIK3CG/GAPT/TYROBP/BTN3A1/MNDA/PLA2G2D/IL2RA/BMP4/CD40LG/NFATC2/CD46/DLG1/ADA/TNFSF8/SOX11 | 48 |
| BP | GO:0042113 | B cell activation | 48/1200 | 334/18723 | 1.21E-07 | 1.76E-05 | 1.46E-05 | CD19/MZB1/SAMSN1/CR2/POU2AF1/LAT2/CD38/XBP1/PLCG2/CD86/CD180/BANK1/IKZF3/CD79A/SH3KBP1/BATF/ITGA4/FCRL1/IL21/FLT3/ST3GAL1/CTLA4/LYN/FCRL3/IGLL1/PTPRC/PTK2B/GPR183/DNAJB9/BTK/PRKCB/VAV3/TCIRG1/PIK3R1/THEMIS2/MS4A1/NCKAP1L/LAX1/CR1/IL7R/GAPT/TYROBP/MNDA/CD40LG/NFATC2/PIK3CD/ADA/KLF6 | 48 |
| BP | GO:1903037 | regulation of leukocyte cell-cell adhesion | 48/1200 | 336/18723 | 1.45E-07 | 1.95E-05 | 1.62E-05 | LILRB2/XBP1/CD86/VAV1/WNK1/SLAMF1/RASAL3/GPNMB/ITGA4/AIF1/IL21/FUT7/RHOH/SKAP1/CTLA4/CLECL1/LYN/CHST2/PRKCQ/PTPRC/ICOS/IL1B/LILRB1/SELP/PCK1/NCKAP1L/LAX1/ADTRP/CR1/IL7R/CD80/CCL5/EGR3/LCK/PLA2G2D/VNN1/IL2RA/ADAM8/BMP4/CD40LG/MIA3/CD46/DLG1/TNFSF11/NRARP/ADA/HAS2/KLF4 | 48 |
| BP | GO:0032102 | negative regulation of response to external stimulus | 48/1200 | 420/18723 | 7.15E-05 | 0.002815 | 0.00234 | CCK/TNFAIP6/STAP1/KRT1/MFHAS1/AIF1/SUSD4/CD200/TREM2/CST7/LYN/PTPRC/F12/ROBO2/GP1BA/LILRB1/FGR/KREMEN1/GPR18/WNT3/CLDN3/TARBP2/GJA1/ADTRP/CR1/IL22RA2/PPARG/ARRB2/SAA1/LRFN5/GPR31/SEMA7A/LTF/IL2RA/SHARPIN/NLRP6/NR1D2/CXCL13/KNG1/MMP12/PLK2/NUCB2/TSPAN8/ADA/SIGLEC10/LILRA2/KLF4/F2 | 48 |
| BP | GO:0033674 | positive regulation of kinase activity | 48/1200 | 467/18723 | 0.000833 | 0.016032 | 0.013324 | CD19/MAPT/CD86/RASSF2/STAP1/WNK1/MST1R/ERN1/CCND2/FLT3/LMO4/TREM2/AGAP2/LYN/GHR/ADRB2/PTPRC/PTK2B/EFNA5/TIAM1/IL1B/EPHB3/VAV3/FGR/LILRA5/EGF/NCKAP1L/CCL5/PIK3CG/FGFR4/CHRNA3/MAPRE3/FGFR2/FZD5/LTF/ADAM8/NTF3/HTR2B/MERTK/DLG1/AJUBA/TNFSF11/RET/WNT11/ADRA2A/F2/NTRK2/FZD10 | 48 |
| BP | GO:0001667 | ameboidal-type cell migration | 47/1200 | 475/18723 | 0.002049 | 0.030401 | 0.025265 | PLCG2/KRT16/MESP1/ENPP2/AMOTL1/ITGA4/RAB25/ADAMTS9/TBX1/JUP/PTK2B/P2RX4/MMP9/KRT2/LPXN/KANK1/ANXA6/PAK3/EGF/GJA1/ADTRP/ARHGDIB/PPARG/PIK3CG/EGR3/ARHGAP4/SEMA7A/FGFBP1/SOX18/PTN/BMP4/HTR2B/SCG2/CXCL13/ACVR1B/MIA3/S100P/PIK3CD/FERMT1/PLK2/RET/HAS2/WNT11/TIMP1/KLF4/DNAJA4/STAT5A | 47 |
| CC | GO:0062023 | collagen-containing extracellular matrix | 47/1263 | 425/19550 | 0.000223 | 0.00831 | 0.007478 | CTSS/LAMB4/COL4A4/KRT1/ADAMTS9/NID1/F12/MGP/VIT/FCN2/COL4A3/MMP9/WNT2B/ADAMDEC1/ADAMTS3/TGFB3/FCN1/COL4A2/ANXA6/FBN1/CTSL/COL4A1/LAMC3/FCN3/FGFR2/SEMA7A/KAZALD1/PTN/THBS2/PRELP/COL15A1/DLG1/C1QA/COL4A6/KNG1/TINAGL1/MBL2/C1QB/AZGP1/TIMP1/ABI3BP/ADAMTS20/ZP1/TGM2/F2/ECM1/LAMC2 | 47 |
| MF | GO:0004175 | endopeptidase activity | 47/1229 | 430/18368 | 0.000622 | 0.022854 | 0.020624 | CTSS/TLL2/MMP3/PIGK/SEC11C/GZMA/MMP10/MMP7/ADAMTS9/OVCH2/ADAM28/TMPRSS13/RHBDD1/F12/PSMB11/MMP1/MMP9/KLK11/ADAMDEC1/ADAMTS6/ADAMTS3/CASP10/MMP27/ADAM23/HTRA4/CTSL/PCSK2/TMPRSS11D/CAPNS2/GZMH/TYSND1/PRSS57/LTF/GZMB/ADAM8/KLK10/ACE2/KLK7/PGC/MMP12/NRIP3/PRSS22/MMP13/CLCA2/ADAMTS20/F2/TMPRSS11B | 47 |
| BP | GO:0030099 | myeloid cell differentiation | 46/1200 | 381/18723 | 2.62E-05 | 0.001329 | 0.001105 | HCLS1/IRF4/RASSF2/MFHAS1/BATF/JAG1/CTNNBIP1/MYC/MEIS1/LILRB3/SCIN/TREM2/LYN/PTK2B/TLR2/GPR183/OCSTAMP/MPL/LILRB1/TCIRG1/MMP9/ZNF385A/PIK3R1/F2RL1/FBN1/NCKAP1L/PPARG/PITHD1/HSPA1B/IL17A/TESC/TSPAN2/TYROBP/LTF/BMP4/HSPA1A/ACVR1B/PIK3CD/MT1G/PIR/MITF/TNFSF11/PLA2G3/IL31RA/TGFBR3/CEBPE | 46 |
| BP | GO:0006816 | calcium ion transport | 46/1200 | 422/18723 | 0.000299 | 0.007662 | 0.006368 | CD19/LILRB2/JSRP1/PLCG2/CYBA/HSPA2/WNK3/P2RX5/CXCR4/TRPC4/LYN/FCRL3/PTPRC/PTK2B/P2RX4/LILRB1/PRKCB/KCNN4/P2RX1/P2RX7/MS4A1/LILRA5/ANXA6/BSPRY/EGF/GJA1/CCL5/PIK3CG/LCK/ARRB2/CACNA1A/STAC2/CATSPER1/PKDREJ/BHLHA15/HTR2B/RGS4/TRPA1/SPINK1/ADRA2A/PDE4B/CACNG6/LILRA2/NMUR1/F2/SLN | 46 |
| BP | GO:0051480 | regulation of cytosolic calcium ion concentration | 45/1200 | 353/18723 | 8.06E-06 | 0.000542 | 0.000451 | CD19/JSRP1/CD38/PLCG2/P2RY1/CYBA/P2RY10/NPY2R/GPR65/P2RX5/CXCR4/TRPC4/LYN/PTPRC/PTK2B/GPR174/P2RX4/P2RX1/P2RX7/GPR18/MS4A1/CD52/F2RL1/KCNA5/GJA1/PIK3CG/LCK/SAA1/PROK2/CACNA1A/LPAR6/C3AR1/HTR2B/CXCL13/TRPA1/CCR3/FPR1/KNG1/PTGIR/AGTR1/CXCR1/EDN2/TGM2/F2/CNGB1 | 45 |
| BP | GO:0002237 | response to molecule of bacterial origin | 45/1200 | 363/18723 | 1.65E-05 | 0.000944 | 0.000784 | LILRB2/LY96/NUGGC/XBP1/PLCG2/CD86/CD180/STAP1/IL24/TREM2/LYN/TLR2/CXCL1/IL1B/CD14/SCIMP/LILRB1/SELP/CXCL3/KCNJ8/LY86/TLR1/PCK1/CXCL2/GJA1/CD80/CCL5/CXCL6/GPR31/MPO/FGFR2/FZD5/LTF/CXCL13/STAR/IL12RB2/S100A14/ANKRD1/IRAK2/TNIP3/PTGIR/PDE4B/LILRA2/PF4V1/CEBPE | 45 |
| BP | GO:0061564 | axon development | 45/1200 | 467/18723 | 0.004139 | 0.049664 | 0.041275 | CCK/IGSF9/MAPT/CHL1/ARX/ITGA4/MAP2/BOC/RHOH/B4GALT6/EMB/CRABP2/PRKCQ/DLX5/FSTL4/EFNA5/NOVA2/TIAM1/EPHB3/ROBO2/PTPRF/PARD3/KREMEN1/WNT3/PLXNB1/PLXNC1/PAK3/NRCAM/TSPAN2/ARHGAP4/NEFL/FGFR2/SEMA7A/PTN/ATL1/FLRT3/EFNA3/PLA2G10/RET/KLF4/LRTM1/LHX3/NTRK2/SLITRK4/LAMC2 | 45 |
| BP | GO:1902105 | regulation of leukocyte differentiation | 44/1200 | 279/18723 | 2.64E-08 | 5.61E-06 | 4.67E-06 | HCLS1/LILRB2/IRF4/XBP1/CD86/RASSF2/IKZF3/TMEM176B/CTNNBIP1/MYC/RHOH/LILRB3/CTLA4/TREM2/LYN/FCRL3/PTPRC/BTK/OCSTAMP/LILRB1/PCK1/PIK3R1/FBN1/NCKAP1L/CR1/IL7R/CD80/EGR3/IL17A/TESC/TYROBP/LTF/VNN1/IL2RA/ADAM8/BMP4/CD46/MITF/TMEM176A/TNFSF11/PLA2G3/NRARP/ADA/TOX | 44 |
| BP | GO:0018108 | peptidyl-tyrosine phosphorylation | 44/1200 | 375/18723 | 7.68E-05 | 0.002857 | 0.002374 | HCLS1/CCK/SAMSN1/PLCG2/BANK1/STAP1/ENPP2/IL24/MST1R/IL21/FLT3/TREM2/PECAM1/HES5/LYN/GHR/PTPRC/PTK2B/BTK/EFNA5/EPHB3/FGR/MATK/LILRA5/ITK/EGF/CD80/CCL5/IL22RA2/FGFR4/LCK/ARRB2/FGFR2/CLEC6A/NTF3/WEE1/IL12RB2/MERTK/SPINK1/RET/IL31RA/ADRA2A/NTRK2/CRLF1 | 44 |
| BP | GO:0018212 | peptidyl-tyrosine modification | 44/1200 | 378/18723 | 9.25E-05 | 0.00319 | 0.002651 | HCLS1/CCK/SAMSN1/PLCG2/BANK1/STAP1/ENPP2/IL24/MST1R/IL21/FLT3/TREM2/PECAM1/HES5/LYN/GHR/PTPRC/PTK2B/BTK/EFNA5/EPHB3/FGR/MATK/LILRA5/ITK/EGF/CD80/CCL5/IL22RA2/FGFR4/LCK/ARRB2/FGFR2/CLEC6A/NTF3/WEE1/IL12RB2/MERTK/SPINK1/RET/IL31RA/ADRA2A/NTRK2/CRLF1 | 44 |
| CC | GO:0045121 | membrane raft | 44/1263 | 335/19550 | 5.79E-06 | 0.000404 | 0.000363 | CD19/CD226/LAT2/MAPT/CD79A/MS4A4A/KCNA3/SLC6A4/SLC2A1/SKAP1/TREM2/PECAM1/TRPC4/LYN/LY6K/PTPRC/PTK2B/PODXL/TLR2/BTK/EFNA5/CD14/BIRC3/CXADR/P2RX1/TLR1/MS4A1/KCNA5/GJA1/ADTRP/CR1/TEX101/CHRNA3/LCK/MAL2/MALL/CDH1/LYPD6/ACE2/PLLP/DLG1/ARID3C/RET/HAS2 | 44 |
| CC | GO:0098857 | membrane microdomain | 44/1263 | 335/19550 | 5.79E-06 | 0.000404 | 0.000363 | CD19/CD226/LAT2/MAPT/CD79A/MS4A4A/KCNA3/SLC6A4/SLC2A1/SKAP1/TREM2/PECAM1/TRPC4/LYN/LY6K/PTPRC/PTK2B/PODXL/TLR2/BTK/EFNA5/CD14/BIRC3/CXADR/P2RX1/TLR1/MS4A1/KCNA5/GJA1/ADTRP/CR1/TEX101/CHRNA3/LCK/MAL2/MALL/CDH1/LYPD6/ACE2/PLLP/DLG1/ARID3C/RET/HAS2 | 44 |
| BP | GO:0006909 | phagocytosis | 43/1200 | 308/18723 | 1.20E-06 | 0.00012 | 9.95E-05 | MYO1G/PLCG2/VAV1/ARL8B/STAP1/SLAMF1/MST1R/CYBA/NCF4/AIF1/RAB20/BIN2/TXNDC5/RHOH/TREM2/PECAM1/LYN/IGLL1/PTPRC/TLR2/IL1B/CD14/GULP1/FCN2/VAV3/FGR/FCN1/F2RL1/NCKAP1L/SPACA3/C3/RAB11FIP2/FCN3/TYROBP/NCF2/IL2RG/MERTK/MARCO/MEGF10/MBL2/TGM2/CEBPE/XKR9 | 43 |
| BP | GO:0010959 | regulation of metal ion transport | 43/1200 | 406/18723 | 0.000837 | 0.016051 | 0.01334 | CD19/LILRB2/JSRP1/PLCG2/WNK1/STK39/CYBA/HSPA2/WNK3/TREM2/P2RX5/CXCR4/LYN/FCRL3/ADRB2/PTK2B/P2RX4/LILRB1/KCNN4/P2RX1/P2RX7/LILRA5/KCNA5/EGF/GJA1/CCL5/KCNJ2/PIK3CG/TESC/ARRB2/STAC2/RGS4/DLG1/SPINK1/NKAIN3/ADRA2A/PDE4B/CACNG6/LILRA2/F2/MLLT6/RNF207/SLN | 43 |
| BP | GO:0030595 | leukocyte chemotaxis | 42/1200 | 230/18723 | 6.28E-10 | 2.78E-07 | 2.31E-07 | VAV1/STAP1/WNK1/SLAMF1/STK39/AIF1/PREX1/CXCR4/LYN/PTK2B/DPEP1/GPR183/CXCL1/CXADR/CXCL3/VAV3/FFAR2/GPR18/CXCL2/F2RL1/NCKAP1L/C10orf99/TREM1/CCL5/PIK3CG/CXCL6/SAA1/CCL18/PLA2G7/PTN/C3AR1/ADAM8/SCG2/CXCL13/S100A14/PIK3CD/TNFSF11/CCL13/PDE4B/CXCR1/EDN2/PF4V1 | 42 |
| BP | GO:0030217 | T cell differentiation | 42/1200 | 257/18723 | 1.92E-08 | 4.25E-06 | 3.53E-06 | LILRB2/CD3D/IRF4/XBP1/CD86/VAV1/BATF/CD3G/IL21/FUT7/PREX1/RHOH/CTLA4/PTPRC/GPR183/IL1B/PSMB11/TCIRG1/GPR18/PCK1/ITK/NCKAP1L/CR1/IL7R/CTSL/CD80/EGR3/LCK/FZD5/PLA2G2D/RORC/VNN1/IL2RA/ADAM8/BMP4/CD46/PIK3CD/EOMES/NRARP/ADA/TNFSF8/TOX | 42 |
| BP | GO:0022409 | positive regulation of cell-cell adhesion | 42/1200 | 284/18723 | 3.37E-07 | 4.16E-05 | 3.46E-05 | LILRB2/XBP1/CD86/VAV1/SLAMF1/RASAL3/ITGA4/AIF1/IL21/FUT7/RHOH/SKAP1/CLECL1/SOX2/LYN/CHST2/PRKCQ/PTPRC/PODXL/ICOS/IL1B/LILRB1/SELP/MAGI1/PCK1/NCKAP1L/CR1/IL7R/CD80/CCL5/EGR3/LCK/VNN1/IL2RA/ADAM8/CXCL13/CD40LG/CD46/TNFSF11/MEGF10/ADA/HAS2 | 42 |
| BP | GO:0007204 | positive regulation of cytosolic calcium ion concentration | 42/1200 | 319/18723 | 7.21E-06 | 0.000491 | 0.000408 | CD19/JSRP1/CD38/PLCG2/P2RY1/CYBA/P2RY10/NPY2R/GPR65/P2RX5/CXCR4/LYN/PTPRC/PTK2B/GPR174/P2RX4/P2RX1/P2RX7/GPR18/MS4A1/CD52/F2RL1/GJA1/PIK3CG/LCK/SAA1/PROK2/CACNA1A/LPAR6/C3AR1/HTR2B/CXCL13/TRPA1/CCR3/FPR1/KNG1/PTGIR/AGTR1/CXCR1/EDN2/TGM2/F2 | 42 |
| BP | GO:0001655 | urogenital system development | 42/1200 | 338/18723 | 2.96E-05 | 0.001417 | 0.001178 | EAF2/COL4A4/SULF2/VANGL2/JAG1/CTNNBIP1/MYC/ANKS6/NID1/PECAM1/HES5/PODXL/LGR4/AKR1B1/ITGA8/EPHB3/ROBO2/TNS2/KCNJ8/TP63/COL4A3/MMP9/WNT2B/ADAMTS6/FBN1/SERPINB5/COL4A1/ALOX15B/FGFR2/PSAPL1/BMP4/GDF6/IRX2/EPCAM/DLG1/SERPINB7/RET/HAS2/AGTR1/WNT11/SOX11/CRLF1 | 42 |
| BP | GO:0032496 | response to lipopolysaccharide | 42/1200 | 343/18723 | 4.18E-05 | 0.001834 | 0.001524 | LILRB2/LY96/NUGGC/XBP1/PLCG2/CD86/CD180/STAP1/IL24/TREM2/LYN/TLR2/CXCL1/IL1B/CD14/SCIMP/LILRB1/SELP/CXCL3/KCNJ8/LY86/PCK1/CXCL2/GJA1/CD80/CCL5/CXCL6/MPO/FGFR2/LTF/CXCL13/STAR/IL12RB2/S100A14/ANKRD1/IRAK2/TNIP3/PTGIR/PDE4B/LILRA2/PF4V1/CEBPE | 42 |
| BP | GO:0043087 | regulation of GTPase activity | 42/1200 | 348/18723 | 5.84E-05 | 0.002388 | 0.001984 | VAV1/WNK1/RASAL3/GPR65/CPEB2/PREX1/GRHL3/PTK2B/RGS7/TBC1D9/ARHGAP9/EFNA5/TIAM1/EPHB3/NGEF/VAV3/PLXNB1/PLXNC1/F2RL1/ARHGEF26/CCL5/ARAP2/RAB11FIP2/RASAL1/ARRB2/CCL18/DOCK9/SIPA1/NTF3/SIPA1L2/CXCL13/RGS4/AJUBA/EVI5L/WNT11/CCL13/ADRB1/TGM2/RGS1/NTRK2/ODAM/FZD10 | 42 |
| BP | GO:0051251 | positive regulation of lymphocyte activation | 42/1200 | 362/18723 | 0.000142 | 0.004442 | 0.003692 | LILRB2/CD38/XBP1/CD86/VAV1/SH3KBP1/SLAMF1/RASAL3/AIF1/IL21/RHOH/CLECL1/LYN/FCRL3/PRKCQ/IGLL1/PTPRC/GPR183/BTK/ICOS/IL1B/MPL/LILRB1/VAV3/PCK1/NCKAP1L/CR1/IL7R/CD80/CCL5/EGR3/LCK/TYROBP/VNN1/IL2RA/ADAM8/CD40LG/NFATC2/CD46/TNFSF11/ADA/TOX | 42 |
| CC | GO:0005788 | endoplasmic reticulum lumen | 42/1263 | 313/19550 | 5.48E-06 | 0.000404 | 0.000363 | MZB1/COL4A4/TMEM43/MYDGF/ERLEC1/CES2/FLT3/CALU/TXNDC5/DNAJC10/SDF2L1/F5/PDIA4/DNAJB9/CYP2W1/COL4A3/UGGT1/TSPAN5/WNT3/COL4A2/DBI/FBN1/IGFBP3/PDIA6/COL4A1/C3/BCHE/ARSF/SLC27A2/DNAJC3/BMP4/SCG2/ACE2/MIA3/COL15A1/COL4A6/KNG1/TIMP1/TMEM132A/CES1/F2/ARSD | 42 |
| BP | GO:0097529 | myeloid leukocyte migration | 41/1200 | 220/18723 | 5.30E-10 | 2.56E-07 | 2.13E-07 | VAV1/STAP1/SLAMF1/TRIM55/AIF1/FUT7/PREX1/RHOH/CD200/TREM2/PECAM1/LYN/PTK2B/DPEP1/CXCL1/P2RX4/CXADR/CXCL3/VAV3/CXCL2/NCKAP1L/TREM1/CCL5/PIK3CG/IL17A/CXCL6/SAA1/CCL18/PLA2G7/C3AR1/ADAM8/SCG2/CXCL13/S100A14/PIK3CD/TNFSF11/CCL13/PDE4B/CXCR1/EDN2/PF4V1 | 41 |
| BP | GO:0043062 | extracellular structure organization | 41/1200 | 302/18723 | 4.31E-06 | 0.000328 | 0.000273 | CTSS/TLL2/MMP3/LAMB4/COL4A4/PRDX4/SULF2/MMP10/MMP7/ADAMTS9/NID1/SLC39A8/ENG/VIT/COL4A3/MMP1/MMP9/ADAMTS6/ADAMTS3/ADAMTSL3/COL4A2/MMP27/ADTRP/SERPINB5/TCF15/COL4A1/FGFR4/DNAJB6/KAZALD1/ADAM8/COL15A1/MIA/KLK7/COL4A6/FERMT1/MMP12/C6orf15/HAS2/MMP13/TEX14/ADAMTS20 | 41 |
| BP | GO:0045229 | external encapsulating structure organization | 41/1200 | 304/18723 | 5.09E-06 | 0.00037 | 0.000308 | CTSS/TLL2/MMP3/LAMB4/COL4A4/PRDX4/SULF2/MMP10/MMP7/ADAMTS9/NID1/SLC39A8/ENG/VIT/COL4A3/MMP1/MMP9/ADAMTS6/ADAMTS3/ADAMTSL3/COL4A2/MMP27/ADTRP/SERPINB5/TCF15/COL4A1/FGFR4/DNAJB6/KAZALD1/ADAM8/COL15A1/MIA/KLK7/COL4A6/FERMT1/MMP12/C6orf15/HAS2/MMP13/ADAMTS20/TGM3 | 41 |
| BP | GO:0006959 | humoral immune response | 41/1200 | 317/18723 | 1.42E-05 | 0.000839 | 0.000697 | CR2/POU2AF1/RNASE6/KRT1/CR1L/JCHAIN/SUSD4/TREM2/ST6GAL1/IGLL1/PTPRC/GPR183/CXCL1/IL1B/CXCL3/FCN2/IL17F/CFH/CXCL2/MS4A1/FCN1/CR1/TREM1/IL17A/C3/CXCL6/FCN3/LYZ/LTF/CXCL13/CD46/C1QA/KLK7/DEFB1/PGC/MBL2/C1QB/CCL13/PF4V1/GNLY/F2 | 41 |
| BP | GO:0002831 | regulation of response to biotic stimulus | 41/1200 | 327/18723 | 2.97E-05 | 0.001417 | 0.001178 | CD226/LY96/PLCG2/VAV1/CD180/NLRC4/ZBP1/CYBA/SUSD4/IL21/TREM2/LYN/AIM2/APOBEC3G/BIRC3/SCIMP/LILRB1/IL17F/LY86/FGR/FFAR2/FCN1/TARBP2/F2RL1/PAK3/CR1/CCL5/PPARG/IL17A/CXCL6/ARRB2/TYROBP/MNDA/LTF/CLEC6A/ADAM8/KLK7/PGC/MMP12/LILRA2/ZDHHC11 | 41 |
| BP | GO:0050863 | regulation of T cell activation | 41/1200 | 329/18723 | 3.42E-05 | 0.001595 | 0.001326 | LILRB2/IRF4/XBP1/CD86/VAV1/SLAMF1/RASAL3/GPNMB/AIF1/IL21/RHOH/CTLA4/CLECL1/LYN/PRKCQ/PTPRC/ICOS/IL1B/LILRB1/PCK1/NCKAP1L/LAX1/CR1/IL7R/CD80/CCL5/EGR3/LCK/PLA2G2D/VNN1/IL2RA/ADAM8/BMP4/CD40LG/CD46/DLG1/TNFSF11/NRARP/ADA/TNFSF8/TOX | 41 |
| BP | GO:0071692 | protein localization to extracellular region | 41/1200 | 368/18723 | 0.000398 | 0.00924 | 0.00768 | CD38/RAB3C/RAB11B/SEL1L/PLEK/RAB3B/CD200/TREM2/ARFGAP3/PDIA4/TLR2/EFNA5/TIAM1/IL1B/KCNN4/NNAT/TCIRG1/UNC13B/TGFB3/F2RL1/KCNA5/FBN1/STXBP5/GJA1/ADTRP/CCL5/ARL4D/VSNL1/FAM3B/RAB11FIP2/EXPH5/SAA1/UCP2/ADAM8/PARD6A/SCG2/CPLX3/MIA3/ANKRD1/PCLO/ADRA2A | 41 |
| BP | GO:0050678 | regulation of epithelial cell proliferation | 41/1200 | 381/18723 | 0.000801 | 0.015527 | 0.012904 | EAF2/XBP1/MYDGF/IL26/CYBA/ITGA4/SULF2/MYC/TBX1/SOX2/HES5/DLX5/TP63/COL4A3/EGF/GJA1/SERPINB5/PPARG/SLURP1/EGR3/FGFR2/FGFBP1/PTN/BMP4/SGPP2/HTR2B/SCG2/PIK3CD/DLG1/CCR3/MMP12/NRARP/HAS2/AGTR1/TGFBR3/SOX11/BMP5/TNMD/ODAM/ECM1/STAT5A | 41 |
| BP | GO:0006631 | fatty acid metabolic process | 41/1200 | 390/18723 | 0.001259 | 0.021234 | 0.017648 | XBP1/PDK1/CES2/APOC1/ACOT2/ALOX5AP/ALDH3A2/DEGS1/AKR1B1/IL1B/ELOVL4/ACOXL/PLA2G4D/SLC27A6/PCK1/CYP4F8/TYRP1/ACADSB/ADTRP/ACSM1/PPARG/CYP7A1/C3/ABHD6/ALOX15B/CYP2C18/TYSND1/CYP2J2/SLC27A2/CYP4F12/ADH7/ELOVL2/GSTA1/SCD/CYP4F11/PLA2G3/PLA2G10/ELOVL6/EDN2/CES1/CYP24A1 | 41 |
| BP | GO:0001503 | ossification | 41/1200 | 408/18723 | 0.002901 | 0.039211 | 0.032588 | SRGN/RASSF2/TUFT1/GDPD2/GPNMB/JAG1/CTNNBIP1/SOX2/ADRB2/PTK2B/LGR4/DLX5/MGP/NPPC/BAMBI/TP63/TCIRG1/P2RX7/FGR/KREMEN1/MMP9/WNT3/CHRDL2/TGFB3/PLXNB1/PPARG/MN1/FGFR2/SEMA7A/KAZALD1/LTF/PTN/BMP4/STATH/TNFSF11/WNT11/MMP13/SOX11/BMP5/ECM1/CYP24A1 | 41 |
| CC | GO:0030667 | secretory granule membrane | 41/1263 | 311/19550 | 1.09E-05 | 0.000622 | 0.00056 | GLIPR1/DSP/LILRB2/CD38/CD53/SIGLEC5/CYBA/ARL8A/CLEC4D/LILRB3/SELL/ITGAX/PECAM1/PLD1/DEGS1/CYBB/PTPRC/TLR2/PKP1/CD14/SELP/P2RX1/TCIRG1/ENPP4/NCKAP1L/SPACA3/STXBP5/CR1/LAIR1/TEX101/TYROBP/CLEC12A/SLC27A2/VNN1/C3AR1/ADAM8/SVIP/CD46/FPR1/CXCR1/MGAM | 41 |
| BP | GO:0031349 | positive regulation of defense response | 40/1200 | 278/18723 | 1.30E-06 | 0.000124 | 0.000103 | CCK/CD226/PLCG2/VAV1/NLRC4/STAP1/ZBP1/TLR10/CYBA/IL21/ALOX5AP/TREM2/LYN/AIM2/TLR2/BTK/IL1B/FFAR2/FCN1/LILRA5/F2RL1/PAK3/GJA1/FABP4/CCL5/PIK3CG/C3/TYROBP/PLA2G7/MNDA/CLEC6A/ADAM8/KLK7/PGC/MMP12/TNFSF11/PLA2G3/AGTR1/LILRA2/TGM2 | 40 |
| BP | GO:0030198 | extracellular matrix organization | 40/1200 | 301/18723 | 9.45E-06 | 0.000607 | 0.000504 | CTSS/TLL2/MMP3/LAMB4/COL4A4/PRDX4/SULF2/MMP10/MMP7/ADAMTS9/NID1/SLC39A8/ENG/VIT/COL4A3/MMP1/MMP9/ADAMTS6/ADAMTS3/ADAMTSL3/COL4A2/MMP27/ADTRP/SERPINB5/TCF15/COL4A1/FGFR4/DNAJB6/KAZALD1/ADAM8/COL15A1/MIA/KLK7/COL4A6/FERMT1/MMP12/C6orf15/HAS2/MMP13/ADAMTS20 | 40 |
| BP | GO:0006936 | muscle contraction | 40/1200 | 347/18723 | 0.000232 | 0.006321 | 0.005254 | DSP/JSRP1/CD38/SULF2/DSC2/TNNT3/NPY2R/RCSD1/CXCR4/JUP/ADRB2/P2RX4/KCNJ8/P2RX1/CRYAB/PPP1R13L/ANXA6/KCNA5/GJA1/KCNJ2/PIK3CG/TNNT1/CHRNA3/PPP1R12B/PROK2/STAC2/HTR2B/ACE2/UTS2/DLG1/HTR7/ADA/ADRA2A/PDE4B/EDN2/NMUR1/NEUROG1/MYBPC2/MYL1/RNF207 | 40 |
| BP | GO:0002460 | adaptive immune response based on somatic recombination of immune receptors built from immunoglobulin superfamily domains | 40/1200 | 356/18723 | 0.000393 | 0.009162 | 0.007615 | CD19/MYO1G/CD226/CR2/IRF4/KLHL6/SLAMF1/BATF/CR1L/JAG1/SUSD4/FUT7/TREM2/PRKCQ/IGLL1/PTPRC/PRF1/BTK/IL1B/LILRB1/TCIRG1/NCKAP1L/CR1/IL7R/CD80/C3/GAPT/FZD5/CLEC6A/RORC/CXCL13/CD40LG/CD46/C1QA/IL13RA2/MBL2/C1QB/ADA/AZGP1/CD1E | 40 |
| BP | GO:0009306 | protein secretion | 40/1200 | 359/18723 | 0.000465 | 0.010216 | 0.008491 | CD38/RAB3C/RAB11B/SEL1L/PLEK/RAB3B/CD200/TREM2/ARFGAP3/PDIA4/TLR2/EFNA5/TIAM1/IL1B/KCNN4/NNAT/TCIRG1/UNC13B/TGFB3/F2RL1/KCNA5/STXBP5/GJA1/ADTRP/CCL5/ARL4D/VSNL1/FAM3B/RAB11FIP2/EXPH5/SAA1/UCP2/ADAM8/PARD6A/SCG2/CPLX3/MIA3/ANKRD1/PCLO/ADRA2A | 40 |
| BP | GO:0035592 | establishment of protein localization to extracellular region | 40/1200 | 360/18723 | 0.000492 | 0.010757 | 0.00894 | CD38/RAB3C/RAB11B/SEL1L/PLEK/RAB3B/CD200/TREM2/ARFGAP3/PDIA4/TLR2/EFNA5/TIAM1/IL1B/KCNN4/NNAT/TCIRG1/UNC13B/TGFB3/F2RL1/KCNA5/STXBP5/GJA1/ADTRP/CCL5/ARL4D/VSNL1/FAM3B/RAB11FIP2/EXPH5/SAA1/UCP2/ADAM8/PARD6A/SCG2/CPLX3/MIA3/ANKRD1/PCLO/ADRA2A | 40 |
| BP | GO:0002274 | myeloid leukocyte activation | 39/1200 | 223/18723 | 9.05E-09 | 2.40E-06 | 2.00E-06 | CD226/LAT2/IRF4/PLCG2/MAPT/STAP1/SLAMF1/MFHAS1/BATF/AIF1/CLEC4D/PREX1/RHOH/CD200/TREM2/CST7/LYN/PTPRC/TLR2/BTK/TLR1/FGR/F2RL1/SPACA3/CCL5/PIK3CG/CXCL6/TYROBP/LRFN5/MILR1/PIK3CD/MT1G/C1QA/PLA2G3/PLA2G10/IL13RA2/IL31RA/LILRA2/EDN2 | 39 |
| BP | GO:0070663 | regulation of leukocyte proliferation | 39/1200 | 245/18723 | 1.26E-07 | 1.76E-05 | 1.46E-05 | MZB1/LILRB2/CD38/CD86/IKZF3/SLAMF1/RASAL3/GPNMB/AIF1/IL21/CTLA4/CLECL1/LYN/FCRL3/PRKCQ/PTPRC/GPR183/BTK/OCSTAMP/IL1B/MPL/LILRB1/VAV3/NCKAP1L/CR1/CD80/CCL5/TYROBP/MNDA/PLA2G2D/IL2RA/BMP4/CD40LG/NFATC2/CD46/DLG1/ADA/TNFSF8/SOX11 | 39 |
| BP | GO:0016042 | lipid catabolic process | 39/1200 | 320/18723 | 8.58E-05 | 0.003048 | 0.002533 | PLCG2/ENPP2/AADAC/APOC1/PDE3B/BCO1/PLD1/NEU4/IL1B/CYP2W1/ACOXL/PLA2G4D/PCK1/ACER1/PLCD3/ADTRP/PIK3CG/CYP7A1/ABHD6/ENPP6/PLA2G7/HSD11B1/TYSND1/PLA2G2D/SLC27A2/LIPK/FUCA1/CYP4F12/AKR1B10/CYP4F11/PLA2G10/CYP39A1/ADRA2A/SULT2A1/PNLIP/LIPM/CES1/PNPLA1/CYP24A1 | 39 |
| BP | GO:0150063 | visual system development | 39/1200 | 375/18723 | 0.001984 | 0.029952 | 0.024893 | CRYBA4/BFSP2/JAG1/MEIS1/B9D1/SOX2/HES5/GRHL3/TCIRG1/CRYAB/WNT2B/PPP1R13L/NHS/FBN1/THRB/COL4A1/C3/GRHL2/LAMC3/RPGRIP1/FZD5/PTN/NR2E3/PDE6A/BMP4/TULP3/MERTK/DLG1/C1QA/SLC7A11/MITF/RET/WNT16/DIO3/KLF4/FOXL2/SOX11/MAB21L1/NTRK2 | 39 |
| BP | GO:0048880 | sensory system development | 39/1200 | 381/18723 | 0.002628 | 0.036172 | 0.030062 | CRYBA4/BFSP2/JAG1/MEIS1/B9D1/SOX2/HES5/GRHL3/TCIRG1/CRYAB/WNT2B/PPP1R13L/NHS/FBN1/THRB/COL4A1/C3/GRHL2/LAMC3/RPGRIP1/FZD5/PTN/NR2E3/PDE6A/BMP4/TULP3/MERTK/DLG1/C1QA/SLC7A11/MITF/RET/WNT16/DIO3/KLF4/FOXL2/SOX11/MAB21L1/NTRK2 | 39 |
| BP | GO:0032970 | regulation of actin filament-based process | 39/1200 | 397/18723 | 0.005293 | 0.056864 | 0.047259 | HCLS1/GMFG/DSP/ARHGAP18/RHOBTB3/STAP1/DSC2/PLEKHH2/GPR65/PLEK/PREX1/RHPN2/RHOH/SCIN/GRHL3/JUP/PTK2B/EFNA5/CAPZA3/PIK3R1/TGFB3/PFN3/KANK1/F2RL1/PAK3/NCKAP1L/ARHGDIB/SYNPO2/KCNJ2/NTF3/ARFIP2/RGS4/DLG1/ARHGAP28/WNT11/PDE4B/RND3/ODAM/FZD10 | 39 |
| MF | GO:0005539 | glycosaminoglycan binding | 39/1229 | 230/18368 | 6.86E-08 | 6.30E-05 | 5.68E-05 | TNFAIP6/GPNMB/SULF2/JCHAIN/MMP7/SELL/TREM2/PTPRC/TLR2/ENG/PTPRF/SELP/VIT/CFH/ADAMTS3/ANXA6/FBN1/GNS/FGFR4/CXCL6/SAA1/MPO/FGFR2/PRSS57/FGFBP1/PLA2G2D/LTF/PTN/BMP4/THBS2/CXCL13/PRELP/KNG1/TGFBR3/PF4V1/F2/LRTM1/LAMC2/RSPO4 | 39 |
| BP | GO:0050670 | regulation of lymphocyte proliferation | 38/1200 | 225/18723 | 3.69E-08 | 7.26E-06 | 6.03E-06 | MZB1/LILRB2/CD38/CD86/IKZF3/SLAMF1/RASAL3/GPNMB/AIF1/IL21/CTLA4/CLECL1/LYN/FCRL3/PRKCQ/PTPRC/GPR183/BTK/IL1B/MPL/LILRB1/VAV3/NCKAP1L/CR1/CD80/CCL5/TYROBP/MNDA/PLA2G2D/IL2RA/BMP4/CD40LG/NFATC2/CD46/DLG1/ADA/TNFSF8/SOX11 | 38 |
| BP | GO:0032944 | regulation of mononuclear cell proliferation | 38/1200 | 227/18723 | 4.70E-08 | 8.33E-06 | 6.92E-06 | MZB1/LILRB2/CD38/CD86/IKZF3/SLAMF1/RASAL3/GPNMB/AIF1/IL21/CTLA4/CLECL1/LYN/FCRL3/PRKCQ/PTPRC/GPR183/BTK/IL1B/MPL/LILRB1/VAV3/NCKAP1L/CR1/CD80/CCL5/TYROBP/MNDA/PLA2G2D/IL2RA/BMP4/CD40LG/NFATC2/CD46/DLG1/ADA/TNFSF8/SOX11 | 38 |
| BP | GO:0002366 | leukocyte activation involved in immune response | 38/1200 | 275/18723 | 6.31E-06 | 0.000435 | 0.000362 | CD19/POU2AF1/LAT2/IRF4/XBP1/PLCG2/CD86/CD180/SLAMF1/BATF/IL21/ST3GAL1/TREM2/LYN/PTPRC/PTK2B/GPR183/BTK/LILRB1/FGR/PCK1/F2RL1/NCKAP1L/CR1/CD80/PIK3CG/GAPT/TYROBP/MILR1/RORC/CD40LG/CD46/PIK3CD/PLA2G3/EOMES/IL13RA2/ADA/LILRA2 | 38 |
| BP | GO:0002263 | cell activation involved in immune response | 38/1200 | 279/18723 | 8.86E-06 | 0.000581 | 0.000483 | CD19/POU2AF1/LAT2/IRF4/XBP1/PLCG2/CD86/CD180/SLAMF1/BATF/IL21/ST3GAL1/TREM2/LYN/PTPRC/PTK2B/GPR183/BTK/LILRB1/FGR/PCK1/F2RL1/NCKAP1L/CR1/CD80/PIK3CG/GAPT/TYROBP/MILR1/RORC/CD40LG/CD46/PIK3CD/PLA2G3/EOMES/IL13RA2/ADA/LILRA2 | 38 |
| BP | GO:0007162 | negative regulation of cell adhesion | 38/1200 | 303/18723 | 5.70E-05 | 0.002357 | 0.001959 | LILRB2/CD86/WNK1/AKNA/GPNMB/JAG1/PDE3B/CTLA4/PTPRC/PODXL/EFNA5/ARHGDIG/LILRB1/PIK3R1/ADAMDEC1/LPXN/PLXNB1/KANK1/PLXNC1/NCKAP1L/LAX1/ADTRP/CR1/ARHGDIB/CD80/PLA2G2D/IL2RA/SIPA1/BMP4/CDH1/MIA3/EPCAM/DLG1/KNG1/MMP12/NRARP/SPOCK1/KLF4 | 38 |
| BP | GO:0002449 | lymphocyte mediated immunity | 38/1200 | 350/18723 | 0.001037 | 0.018474 | 0.015354 | CD19/SLAMF7/MYO1G/CD226/CR2/VAV1/ARL8B/SLAMF1/BATF/CR1L/JAG1/SUSD4/IL21/FUT7/TREM2/IGLL1/PTPRC/PRF1/BTK/IL1B/LILRB1/TCIRG1/NCKAP1L/CR1/IL7R/C3/GAPT/ARRB2/FZD5/GZMB/CD40LG/CD46/C1QA/IL13RA2/MBL2/C1QB/AZGP1/CD1E | 38 |
| BP | GO:0042742 | defense response to bacterium | 38/1200 | 350/18723 | 0.001037 | 0.018474 | 0.015354 | RNASE6/NLRC4/CYBA/JCHAIN/TREM2/IGLL1/TLR2/SELP/FCN2/IL17F/FGR/GBP6/F2RL1/C10orf99/IL7R/TREM1/IL17A/CXCL6/GSDMC/LYZ/MPO/DCD/LTF/NLRP6/CXCL13/S100A14/KLK7/DEFB1/STATH/PGC/PLAC8/MBL2/TNFSF8/PRB3/GNLY/LCE3B/F2/CEBPE | 38 |
| BP | GO:0051346 | negative regulation of hydrolase activity | 38/1200 | 379/18723 | 0.004194 | 0.049664 | 0.041275 | SERPINI1/WNK1/GZMA/APOC1/CPEB2/CST7/DPEP1/BIRC3/HERPUD1/COL4A3/RARRES1/CRYAB/MMP9/NCKAP1L/CR1/SERPINB5/A2ML1/C3/SERPINA11/ARRB2/DNAJB6/LTF/SERPINB13/SERPINB11/KNG1/SPINK1/SERPINB7/SPOCK1/SERPINI2/TFPI2/TIMP1/KLF4/SMR3B/SPINK7/ECM1/SPINK9/FZD10/SIAH2 | 38 |
| BP | GO:0002699 | positive regulation of immune effector process | 37/1200 | 235/18723 | 3.48E-07 | 4.20E-05 | 3.49E-05 | MZB1/CD226/XBP1/PLCG2/CD86/VAV1/STAP1/SLAMF1/IL21/TREM2/LYN/PTPRC/DNAJB9/BTK/IL1B/SCIMP/LILRB1/IL17F/FGR/FFAR2/PCK1/F2RL1/CR1/CD80/IL17A/C3/TYROBP/SEMA7A/FZD5/CD46/SLC7A5/KLK7/PGC/PLA2G3/IL13RA2/AZGP1/CD1E | 37 |
| BP | GO:1903039 | positive regulation of leukocyte cell-cell adhesion | 37/1200 | 239/18723 | 5.34E-07 | 6.17E-05 | 5.13E-05 | LILRB2/XBP1/CD86/VAV1/SLAMF1/RASAL3/ITGA4/AIF1/IL21/FUT7/RHOH/SKAP1/CLECL1/LYN/CHST2/PRKCQ/PTPRC/ICOS/IL1B/LILRB1/SELP/PCK1/NCKAP1L/CR1/IL7R/CD80/CCL5/EGR3/LCK/VNN1/IL2RA/ADAM8/CD40LG/CD46/TNFSF11/ADA/HAS2 | 37 |
| BP | GO:0050851 | antigen receptor-mediated signaling pathway | 37/1200 | 240/18723 | 5.93E-07 | 6.71E-05 | 5.57E-05 | CD19/CD226/LAT2/CD38/KLHL6/PLCG2/BANK1/CD79A/STAP1/WNK1/PLEKHA1/SKAP1/CTLA4/LYN/CD247/FCRL3/IGLL1/PTPRC/BTK/PRKCB/KCNN4/VAV3/TRAT1/THEMIS2/MS4A1/LPXN/ITK/NCKAP1L/LAX1/LCK/BTN3A1/MNDA/NFATC2/PIK3CD/ADA/PDE4B/BTN1A1 | 37 |
| BP | GO:0043588 | skin development | 37/1200 | 263/18723 | 5.48E-06 | 0.000387 | 0.000322 | DSP/SOX21/KRT16/KRT1/JAG1/IRF6/GRHL3/JUP/TNFRSF19/LGR4/LTB/TP63/AQP3/ACER1/KRT2/TCF15/GRHL2/KRT27/EXPH5/ALOX15B/FGFR2/SOX18/LIPK/SHARPIN/KRT10/ACVR1B/FERMT1/WNT16/PPL/KRT76/LIPM/ASCL4/KRT25/LCE3B/SPRR1A/TGM3/TCHH | 37 |
| BP | GO:0001654 | eye development | 37/1200 | 371/18723 | 0.005101 | 0.056103 | 0.046627 | CRYBA4/BFSP2/JAG1/MEIS1/B9D1/SOX2/HES5/GRHL3/TCIRG1/CRYAB/WNT2B/PPP1R13L/NHS/FBN1/THRB/COL4A1/GRHL2/LAMC3/RPGRIP1/FZD5/PTN/NR2E3/PDE6A/BMP4/TULP3/MERTK/DLG1/SLC7A11/MITF/RET/WNT16/DIO3/KLF4/FOXL2/SOX11/MAB21L1/NTRK2 | 37 |
| BP | GO:0071216 | cellular response to biotic stimulus | 36/1200 | 246/18723 | 2.93E-06 | 0.000241 | 0.0002 | LILRB2/LY96/NUGGC/XBP1/PLCG2/CD86/CD180/STAP1/IL24/TREM2/LYN/TLR2/BTK/CXCL1/IL1B/CD14/SCIMP/LILRB1/CXCL3/LY86/TLR1/CXCL2/CD80/CCL5/CXCL6/FZD5/LTF/CXCL13/STAR/ANKRD1/IRAK2/TNIP3/PDE4B/LILRA2/PF4V1/CEBPE | 36 |
| BP | GO:0043270 | positive regulation of ion transport | 36/1200 | 275/18723 | 3.59E-05 | 0.001658 | 0.001378 | CTSS/CD19/CCK/PLCG2/WNK1/STK39/P2RY1/HSPA2/SLC6A4/NPY2R/RAB3B/WNK3/TREM2/P2RX5/ADRB2/IL1B/P2RX4/KCNN4/P2RX1/P2RX7/LILRA5/CCL5/KCNJ2/TESC/ARRB2/STAC2/ACE2/DLG1/TNFSF11/PLA2G3/PLA2G10/ADRA2A/LILRA2/F2/MLLT6/RNF207 | 36 |
| BP | GO:0072001 | renal system development | 36/1200 | 302/18723 | 0.000245 | 0.006597 | 0.005482 | COL4A4/SULF2/VANGL2/JAG1/CTNNBIP1/MYC/ANKS6/NID1/PECAM1/HES5/PODXL/LGR4/AKR1B1/ITGA8/ROBO2/TNS2/KCNJ8/COL4A3/MMP9/WNT2B/ADAMTS6/FBN1/COL4A1/FGFR2/BMP4/GDF6/IRX2/EPCAM/DLG1/SERPINB7/RET/HAS2/AGTR1/WNT11/SOX11/CRLF1 | 36 |
| BP | GO:0043010 | camera-type eye development | 36/1200 | 322/18723 | 0.000821 | 0.015851 | 0.013174 | CRYBA4/BFSP2/JAG1/MEIS1/B9D1/HES5/GRHL3/TCIRG1/CRYAB/WNT2B/PPP1R13L/NHS/FBN1/THRB/COL4A1/GRHL2/LAMC3/RPGRIP1/FZD5/PTN/NR2E3/PDE6A/BMP4/TULP3/MERTK/DLG1/SLC7A11/MITF/RET/WNT16/DIO3/KLF4/FOXL2/SOX11/MAB21L1/NTRK2 | 36 |
| CC | GO:0030139 | endocytic vesicle | 36/1263 | 336/19550 | 0.002004 | 0.044736 | 0.040254 | CTSS/CD3D/SH3KBP1/SLAMF1/CYBA/NCF4/RAB20/CD3G/RAB11B/CTLA4/PLD1/CYBB/LYN/ADRB2/TLR2/SCIMP/TCIRG1/TLR1/WNT3/TYRP1/EGF/IL7R/CTSL/ARRB2/SAA1/CLEC4E/OCLN/NCF2/MPO/FZD5/LTF/ATP6V0D2/ADAM8/ACE2/MARCO/GNLY | 36 |
| MF | GO:1901681 | sulfur compound binding | 36/1229 | 265/18368 | 3.96E-05 | 0.003033 | 0.002737 | GPNMB/MMP7/SELL/TPK1/PTPRC/PTPRF/SELP/CFH/ADAMTS3/ANXA6/DBI/FBN1/OGDHL/FGFR4/CXCL6/SAA1/MPO/FGFR2/PANK1/PRSS57/FGFBP1/PLA2G2D/LTF/PTN/BMP4/THBS2/CXCL13/PRELP/KNG1/TGFBR3/SULT2A1/PF4V1/F2/LRTM1/LAMC2/RSPO4 | 36 |
| BP | GO:0071219 | cellular response to molecule of bacterial origin | 35/1200 | 221/18723 | 6.22E-07 | 6.89E-05 | 5.72E-05 | LILRB2/LY96/NUGGC/XBP1/PLCG2/CD86/CD180/STAP1/IL24/TREM2/LYN/TLR2/CXCL1/IL1B/CD14/SCIMP/LILRB1/CXCL3/LY86/TLR1/CXCL2/CD80/CCL5/CXCL6/FZD5/LTF/CXCL13/STAR/ANKRD1/IRAK2/TNIP3/PDE4B/LILRA2/PF4V1/CEBPE | 35 |
| BP | GO:0001822 | kidney development | 35/1200 | 293/18723 | 0.000285 | 0.007345 | 0.006104 | COL4A4/SULF2/VANGL2/JAG1/CTNNBIP1/MYC/ANKS6/NID1/PECAM1/HES5/PODXL/LGR4/AKR1B1/ITGA8/ROBO2/TNS2/KCNJ8/COL4A3/MMP9/WNT2B/ADAMTS6/FBN1/FGFR2/BMP4/GDF6/IRX2/EPCAM/DLG1/SERPINB7/RET/HAS2/AGTR1/WNT11/SOX11/CRLF1 | 35 |
| BP | GO:0019216 | regulation of lipid metabolic process | 35/1200 | 331/18723 | 0.002498 | 0.035674 | 0.029648 | CD19/PLCG2/PDK1/AADAC/APOC1/PDE3B/FLT3/TREM2/AGAP2/LYN/PTK2B/IL1B/VAV3/FGR/PCK1/PPARG/PIK3CG/CYP7A1/FGFR4/C3/FGF19/ABHD6/TYSND1/RORC/PSAPL1/HTR2B/NR1D2/STAR/PLA2G3/AGTR1/ADRA2A/BMP5/CES1/F2/CGA | 35 |
| MF | GO:0001664 | G protein-coupled receptor binding | 35/1229 | 295/18368 | 0.000706 | 0.024912 | 0.022481 | PNOC/P2RY1/CXCL1/BAMBI/CXCL3/WNT2B/WNT3/CXCL2/FCN1/SH3GL1/C10orf99/CCL5/HSPA1B/C3/CXCL6/ARRB2/SAA1/CCL18/PROK2/HSPA1A/CXCL13/S100A14/TULP3/DEFB1/MARCO/FPR1/AGTR1/WNT11/WNT16/ADRA2A/CCL13/EDN2/PF4V1/ADRB1/PMCH | 35 |
| BP | GO:0070371 | ERK1 and ERK2 cascade | 34/1200 | 330/18723 | 0.004302 | 0.050109 | 0.041645 | IL26/SLAMF1/MFHAS1/P2RY1/RPS6KA6/GPNMB/MYC/TREM2/LYN/PTPRC/PTK2B/GPR183/TIAM1/IL1B/SCIMP/F2RL1/EGF/CCL5/FGFR4/FGF19/ARRB2/CCL18/NDRG2/FGFR2/SEMA7A/DUSP4/BMP4/HTR2B/NLRP6/DLG1/MARCO/TNFSF11/CCL13/KLF4 | 34 |
| MF | GO:0005125 | cytokine activity | 34/1229 | 235/18368 | 1.73E-05 | 0.001767 | 0.001594 | IL26/IL24/IL21/CXCL1/IL1B/LTB/CXCL3/IL17F/WNT2B/WNT3/CXCL2/TGFB3/C10orf99/CCL5/SLURP1/IL17A/FAM3B/CXCL6/CCL18/CMTM2/BMP4/SCG2/CXCL13/CD40LG/GDF6/TNFSF11/WNT11/TNFSF8/WNT16/TIMP1/CCL13/PF4V1/BMP5/CRLF1 | 34 |
| BP | GO:0002573 | myeloid leukocyte differentiation | 33/1200 | 208/18723 | 1.24E-06 | 0.00012 | 9.95E-05 | HCLS1/IRF4/RASSF2/BATF/CTNNBIP1/MYC/LILRB3/TREM2/LYN/TLR2/GPR183/OCSTAMP/LILRB1/TCIRG1/MMP9/PIK3R1/F2RL1/FBN1/PPARG/IL17A/TESC/TSPAN2/TYROBP/LTF/BMP4/PIK3CD/MT1G/PIR/MITF/TNFSF11/PLA2G3/IL31RA/CEBPE | 33 |
| BP | GO:0050866 | negative regulation of cell activation | 33/1200 | 210/18723 | 1.54E-06 | 0.000144 | 0.000119 | SAMSN1/LILRB2/CD86/BANK1/GPNMB/CD200/CTLA4/TREM2/CST7/LYN/PTPRC/BTK/LILRB1/FGR/NCKAP1L/LAX1/CR1/CD80/TYROBP/LRFN5/MILR1/MNDA/PLA2G2D/IL2RA/GCLC/BMP4/MERTK/DLG1/IL13RA2/NRARP/IL31RA/SOX11/F2 | 33 |
| BP | GO:0019932 | second-messenger-mediated signaling | 33/1200 | 312/18723 | 0.003248 | 0.043345 | 0.036024 | LAT2/CHP2/PLCG2/MAPT/PDE3B/NPY2R/PLEK/TREM2/P2RX5/CXCR4/PTPRC/PTK2B/NPPC/BTK/P2RX4/SELP/P2RX7/TRAT1/SAMD14/BHLHA15/HTR2B/NFATC2/DEFB1/CCR3/FPR1/SPINK1/TNFSF11/ADA/AGTR1/PCLO/CXCR1/EDN2/NMUR1 | 33 |
| BP | GO:0060249 | anatomical structure homeostasis | 33/1200 | 314/18723 | 0.003587 | 0.046027 | 0.038252 | CD38/KRT1/STK39/P2RY1/JCHAIN/USH1G/SLC2A1/WNK3/PECAM1/ADRB2/PTK2B/SLC39A8/AKR1B1/P2RX4/CXADR/P2RX1/TCIRG1/P2RX7/ALDH1A1/MC4R/CLDN3/GJA1/IL17A/RDH12/OCLN/LYZ/LTF/ADAM8/VSIG1/TNFSF11/AZGP1/TFF1/CNGB1 | 33 |
| BP | GO:0001906 | cell killing | 32/1200 | 188/18723 | 3.61E-07 | 4.26E-05 | 3.54E-05 | SLAMF7/CD226/VAV1/ARL8B/STAP1/CR1L/IL21/PTPRC/PRF1/LILRB1/CFH/P2RX7/F2RL1/NCKAP1L/IL7R/TREM1/C3/CXCL6/ARRB2/TYROBP/LYZ/DCD/LTF/GZMB/NLRP6/BCL2L1/AZGP1/CD1E/CCL13/GNLY/LCE3B/F2 | 32 |
| BP | GO:0071674 | mononuclear cell migration | 32/1200 | 196/18723 | 9.41E-07 | 9.80E-05 | 8.14E-05 | MYO1G/WNK1/SLAMF1/STK39/ITGA4/AIF1/FUT7/CD200/PECAM1/CXCR4/LYN/PTK2B/GPR183/C10orf99/ADTRP/CCL5/PIK3CG/SAA1/CCL18/PLA2G7/C3AR1/ADAM8/CXCL13/MIA3/S100A14/PIK3CD/TNFSF11/RET/CCL13/CXCR1/BMP5/ECM1 | 32 |
| BP | GO:0071222 | cellular response to lipopolysaccharide | 32/1200 | 209/18723 | 3.90E-06 | 0.000305 | 0.000253 | LILRB2/LY96/NUGGC/XBP1/PLCG2/CD86/CD180/STAP1/IL24/LYN/TLR2/CXCL1/IL1B/CD14/SCIMP/LILRB1/CXCL3/LY86/CXCL2/CD80/CCL5/CXCL6/LTF/CXCL13/STAR/ANKRD1/IRAK2/TNIP3/PDE4B/LILRA2/PF4V1/CEBPE | 32 |
| BP | GO:0002685 | regulation of leukocyte migration | 32/1200 | 210/18723 | 4.33E-06 | 0.000328 | 0.000273 | STAP1/WNK1/SLAMF1/STK39/ITGA4/AIF1/FUT7/RHOH/CD200/TREM2/LYN/CHST2/PTK2B/P2RX4/SELP/GPR18/F2RL1/NCKAP1L/C10orf99/ADTRP/CCL5/PLA2G7/PTN/C3AR1/ADAM8/CXCL13/MIA3/S100A14/ADA/EDN2/BMP5/ECM1 | 32 |
| BP | GO:0002703 | regulation of leukocyte mediated immunity | 32/1200 | 226/18723 | 2.02E-05 | 0.001108 | 0.000921 | CD226/CR2/PLCG2/VAV1/STAP1/SLAMF1/CR1L/SUSD4/IL21/FUT7/TREM2/LYN/PTPRC/BTK/IL1B/SCIMP/LILRB1/FGR/F2RL1/NCKAP1L/CR1/IL7R/C3/CXCL6/ARRB2/TYROBP/FZD5/CD46/PLA2G3/IL13RA2/AZGP1/CD1E | 32 |
| BP | GO:0010466 | negative regulation of peptidase activity | 32/1200 | 262/18723 | 0.000343 | 0.008496 | 0.007061 | SERPINI1/CST7/DPEP1/BIRC3/HERPUD1/COL4A3/RARRES1/CRYAB/MMP9/CR1/SERPINB5/A2ML1/C3/SERPINA11/ARRB2/DNAJB6/LTF/SERPINB13/SERPINB11/KNG1/SPINK1/SERPINB7/SPOCK1/SERPINI2/TFPI2/TIMP1/KLF4/SMR3B/SPINK7/ECM1/SPINK9/SIAH2 | 32 |
| BP | GO:0044403 | biological process involved in symbiotic interaction | 32/1200 | 290/18723 | 0.001919 | 0.029454 | 0.024479 | CR2/SMARCB1/CD86/ARL8B/SLAMF1/TRIM35/CXCR4/CXADR/FCN1/F2RL1/CR1/TREM1/MID2/CTSL/CD80/CCL5/CLEC4M/HSPA1B/CXCL6/IFI27/FCN3/DCD/LTF/HSPA1A/NLRP6/ACE2/CD46/BCL2L1/AGTR1/SIGLEC1/F2/CLDN9 | 32 |
| BP | GO:0070372 | regulation of ERK1 and ERK2 cascade | 32/1200 | 309/18723 | 0.005092 | 0.056103 | 0.046627 | IL26/SLAMF1/MFHAS1/P2RY1/RPS6KA6/GPNMB/TREM2/LYN/PTPRC/PTK2B/GPR183/TIAM1/IL1B/SCIMP/F2RL1/CCL5/FGFR4/FGF19/ARRB2/CCL18/NDRG2/FGFR2/SEMA7A/DUSP4/BMP4/HTR2B/NLRP6/DLG1/MARCO/TNFSF11/CCL13/KLF4 | 32 |
| BP | GO:0097530 | granulocyte migration | 31/1200 | 148/18723 | 3.75E-09 | 1.11E-06 | 9.20E-07 | VAV1/SLAMF1/FUT7/PREX1/RHOH/PECAM1/DPEP1/CXCL1/CXADR/CXCL3/VAV3/CXCL2/NCKAP1L/TREM1/CCL5/PIK3CG/IL17A/CXCL6/SAA1/CCL18/C3AR1/ADAM8/SCG2/CXCL13/S100A14/PIK3CD/CCL13/PDE4B/CXCR1/EDN2/PF4V1 | 31 |
| BP | GO:0002221 | pattern recognition receptor signaling pathway | 31/1200 | 172/18723 | 1.47E-07 | 1.95E-05 | 1.62E-05 | CTSS/LY96/IRF4/PLCG2/TLR10/MFHAS1/CYBA/TREM2/LYN/FCRL3/LGR4/TLR2/BTK/CD14/BIRC3/SCIMP/TLR1/FFAR2/FCN1/F2RL1/HSPA1B/RAB11FIP2/ARRB2/CLEC4E/LTF/HSPA1A/NLRP6/S100A14/IRAK2/TNIP3/LILRA2 | 31 |
| BP | GO:0050870 | positive regulation of T cell activation | 31/1200 | 216/18723 | 2.07E-05 | 0.001122 | 0.000933 | LILRB2/XBP1/CD86/VAV1/SLAMF1/RASAL3/AIF1/IL21/RHOH/CLECL1/LYN/PRKCQ/PTPRC/ICOS/IL1B/LILRB1/PCK1/NCKAP1L/CR1/IL7R/CD80/CCL5/EGR3/LCK/VNN1/IL2RA/ADAM8/CD40LG/CD46/TNFSF11/ADA | 31 |
| BP | GO:0010951 | negative regulation of endopeptidase activity | 31/1200 | 252/18723 | 0.000372 | 0.00883 | 0.007338 | SERPINI1/CST7/DPEP1/BIRC3/HERPUD1/COL4A3/RARRES1/CRYAB/MMP9/CR1/SERPINB5/A2ML1/C3/SERPINA11/ARRB2/DNAJB6/LTF/SERPINB13/SERPINB11/KNG1/SPINK1/SERPINB7/SPOCK1/SERPINI2/TFPI2/TIMP1/KLF4/SMR3B/SPINK7/SPINK9/SIAH2 | 31 |
| BP | GO:0051924 | regulation of calcium ion transport | 31/1200 | 255/18723 | 0.000457 | 0.010148 | 0.008434 | CD19/LILRB2/JSRP1/PLCG2/CYBA/HSPA2/WNK3/P2RX5/CXCR4/LYN/FCRL3/PTK2B/P2RX4/LILRB1/P2RX1/P2RX7/LILRA5/EGF/GJA1/CCL5/PIK3CG/ARRB2/STAC2/RGS4/SPINK1/ADRA2A/PDE4B/CACNG6/LILRA2/F2/SLN | 31 |
| BP | GO:0001894 | tissue homeostasis | 31/1200 | 268/18723 | 0.001055 | 0.018616 | 0.015471 | CD38/KRT1/STK39/JCHAIN/USH1G/SLC2A1/WNK3/PECAM1/ADRB2/PTK2B/SLC39A8/AKR1B1/P2RX4/CXADR/TCIRG1/P2RX7/ALDH1A1/MC4R/CLDN3/GJA1/IL17A/RDH12/OCLN/LYZ/LTF/ADAM8/VSIG1/TNFSF11/AZGP1/TFF1/CNGB1 | 31 |
| CC | GO:0101002 | ficolin-1-rich granule | 31/1263 | 185/19550 | 9.28E-07 | 0.000129 | 0.000116 | CTSS/GMFG/DSP/LILRB2/TNFAIP6/SIGLEC5/KRT1/PRDX4/ARL8A/CLEC4D/BIN2/ITGAX/JUP/PKP1/CALML5/HK3/TCIRG1/MMP9/FCN1/ENPP4/NCKAP1L/GNS/CR1/HSPA1B/MNDA/ADAM8/PGM2/HSPA1A/FPR1/MGAM/YPEL5 | 31 |
| MF | GO:0017171 | serine hydrolase activity | 31/1229 | 195/18368 | 5.93E-06 | 0.000907 | 0.000819 | CTSS/TLL2/MMP3/SEC11C/GZMA/AADAC/MMP10/MMP7/OVCH2/TMPRSS13/RHBDD1/F12/MMP1/MMP9/KLK11/HTRA4/PCSK2/TMPRSS11D/GZMH/TYSND1/PRSS57/LTF/GZMB/ADAM8/KLK10/KLK7/MMP12/PRSS22/MMP13/F2/TMPRSS11B | 31 |
| BP | GO:0002695 | negative regulation of leukocyte activation | 30/1200 | 187/18723 | 2.95E-06 | 0.000241 | 0.0002 | SAMSN1/LILRB2/CD86/BANK1/GPNMB/CD200/CTLA4/CST7/LYN/PTPRC/BTK/LILRB1/FGR/NCKAP1L/LAX1/CR1/CD80/TYROBP/LRFN5/MILR1/MNDA/PLA2G2D/IL2RA/BMP4/MERTK/DLG1/IL13RA2/NRARP/IL31RA/SOX11 | 30 |
| BP | GO:0007596 | blood coagulation | 30/1200 | 217/18723 | 5.71E-05 | 0.002357 | 0.001959 | PLCG2/VAV1/KRT1/P2RY1/PLEK/F5/LYN/PRKCQ/F12/MPL/GP1BA/SELP/P2RX1/VAV3/F2RL1/ENPP4/ADTRP/PIK3CG/LCK/SAA1/CD40LG/MERTK/SLC7A11/KNG1/CYP4F11/TSPAN8/TFPI2/ADRA2A/PF4V1/F2 | 30 |
| BP | GO:0007599 | hemostasis | 30/1200 | 222/18723 | 8.72E-05 | 0.003048 | 0.002533 | PLCG2/VAV1/KRT1/P2RY1/PLEK/F5/LYN/PRKCQ/F12/MPL/GP1BA/SELP/P2RX1/VAV3/F2RL1/ENPP4/ADTRP/PIK3CG/LCK/SAA1/CD40LG/MERTK/SLC7A11/KNG1/CYP4F11/TSPAN8/TFPI2/ADRA2A/PF4V1/F2 | 30 |
| BP | GO:0050817 | coagulation | 30/1200 | 222/18723 | 8.72E-05 | 0.003048 | 0.002533 | PLCG2/VAV1/KRT1/P2RY1/PLEK/F5/LYN/PRKCQ/F12/MPL/GP1BA/SELP/P2RX1/VAV3/F2RL1/ENPP4/ADTRP/PIK3CG/LCK/SAA1/CD40LG/MERTK/SLC7A11/KNG1/CYP4F11/TSPAN8/TFPI2/ADRA2A/PF4V1/F2 | 30 |
| BP | GO:0043547 | positive regulation of GTPase activity | 30/1200 | 255/18723 | 0.00096 | 0.017468 | 0.014518 | VAV1/GPR65/PREX1/GRHL3/PTK2B/RGS7/TBC1D9/TIAM1/NGEF/PLXNB1/F2RL1/ARHGEF26/CCL5/ARAP2/RAB11FIP2/CCL18/DOCK9/SIPA1/NTF3/SIPA1L2/CXCL13/RGS4/EVI5L/WNT11/CCL13/ADRB1/TGM2/RGS1/ODAM/FZD10 | 30 |
| BP | GO:0050730 | regulation of peptidyl-tyrosine phosphorylation | 30/1200 | 264/18723 | 0.001664 | 0.02665 | 0.022149 | HCLS1/CCK/SAMSN1/PLCG2/BANK1/STAP1/ENPP2/IL24/IL21/FLT3/TREM2/PECAM1/HES5/LYN/GHR/PTPRC/PTK2B/EFNA5/LILRA5/EGF/CD80/CCL5/IL22RA2/ARRB2/CLEC6A/NTF3/SPINK1/IL31RA/ADRA2A/CRLF1 | 30 |
| BP | GO:0021700 | developmental maturation | 30/1200 | 280/18723 | 0.004032 | 0.049118 | 0.040821 | BFSP2/IL21/B4GALT6/HES5/PTK2B/NPPC/AKR1B1/BTK/ZDHHC2/UNC13B/PLXNB1/NRCAM/PPARG/C3/NEFL/FZD5/SOX18/LTF/BHLHA15/ROPN1B/ROPN1/C1QA/DEFB1/FERMT1/SPINK1/PLA2G3/LHX6/PAEP/RET/CNGB1 | 30 |
| CC | GO:0005882 | intermediate filament | 30/1263 | 217/19550 | 6.63E-05 | 0.003081 | 0.002772 | DSP/KRT3/IFFO1/KRT16/KRT1/KRT8/BFSP2/KRT18/JUP/KRT33A/PKP1/KRT2/KRT75/KRT13/CLIP1/KRT6B/KRT27/NEFM/KRT85/NEFL/IFFO2/KRT10/KRT31/LMNB1/KRT34/PPL/KRT76/KRT24/KRT25/KRT26 | 30 |
| CC | GO:0045111 | intermediate filament cytoskeleton | 30/1263 | 258/19550 | 0.001316 | 0.031916 | 0.028719 | DSP/KRT3/IFFO1/KRT16/KRT1/KRT8/BFSP2/KRT18/JUP/KRT33A/PKP1/KRT2/KRT75/KRT13/CLIP1/KRT6B/KRT27/NEFM/KRT85/NEFL/IFFO2/KRT10/KRT31/LMNB1/KRT34/PPL/KRT76/KRT24/KRT25/KRT26 | 30 |
| MF | GO:0008201 | heparin binding | 30/1229 | 166/18368 | 5.44E-07 | 0.00025 | 0.000225 | GPNMB/MMP7/SELL/PTPRC/PTPRF/SELP/CFH/ADAMTS3/FBN1/FGFR4/CXCL6/SAA1/MPO/FGFR2/PRSS57/FGFBP1/PLA2G2D/LTF/PTN/BMP4/THBS2/CXCL13/PRELP/KNG1/TGFBR3/PF4V1/F2/LRTM1/LAMC2/RSPO4 | 30 |
| MF | GO:0004252 | serine-type endopeptidase activity | 30/1229 | 174/18368 | 1.52E-06 | 0.00035 | 0.000315 | CTSS/TLL2/MMP3/SEC11C/GZMA/MMP10/MMP7/OVCH2/TMPRSS13/RHBDD1/F12/MMP1/MMP9/KLK11/HTRA4/PCSK2/TMPRSS11D/GZMH/TYSND1/PRSS57/LTF/GZMB/ADAM8/KLK10/KLK7/MMP12/PRSS22/MMP13/F2/TMPRSS11B | 30 |
| MF | GO:0008236 | serine-type peptidase activity | 30/1229 | 191/18368 | 1.06E-05 | 0.001221 | 0.001102 | CTSS/TLL2/MMP3/SEC11C/GZMA/MMP10/MMP7/OVCH2/TMPRSS13/RHBDD1/F12/MMP1/MMP9/KLK11/HTRA4/PCSK2/TMPRSS11D/GZMH/TYSND1/PRSS57/LTF/GZMB/ADAM8/KLK10/KLK7/MMP12/PRSS22/MMP13/F2/TMPRSS11B | 30 |
| BP | GO:0031348 | negative regulation of defense response | 29/1200 | 258/18723 | 0.002319 | 0.033477 | 0.027822 | CCK/TNFAIP6/KRT1/MFHAS1/SUSD4/CD200/TREM2/CST7/LYN/PTPRC/LILRB1/FGR/TARBP2/CR1/IL22RA2/PPARG/ARRB2/SAA1/LRFN5/GPR31/IL2RA/SHARPIN/NLRP6/NR1D2/MMP12/PLK2/ADA/SIGLEC10/KLF4 | 29 |
| CC | GO:0070820 | tertiary granule | 29/1263 | 164/19550 | 6.60E-07 | 0.000123 | 0.00011 | CTSS/DSP/LILRB2/TNFAIP6/CD53/SIGLEC5/CYBA/ARL8A/CLEC4D/ITGAX/PLD1/CYBB/CXCL1/PKP1/TCIRG1/MMP9/ENPP4/NCKAP1L/CR1/LAIR1/LYZ/CLEC12A/LTF/ADAM8/SVIP/FPR1/MGAM/YPEL5/CRISP3 | 29 |
| BP | GO:0002833 | positive regulation of response to biotic stimulus | 28/1200 | 168/18723 | 2.93E-06 | 0.000241 | 0.0002 | CD226/LY96/PLCG2/VAV1/CD180/NLRC4/ZBP1/CYBA/IL21/LYN/AIM2/SCIMP/IL17F/LY86/FFAR2/FCN1/F2RL1/PAK3/CCL5/IL17A/TYROBP/MNDA/CLEC6A/ADAM8/KLK7/PGC/MMP12/LILRA2 | 28 |
| BP | GO:0009913 | epidermal cell differentiation | 28/1200 | 202/18723 | 9.50E-05 | 0.003255 | 0.002705 | DSP/KRT16/JAG1/IRF6/HES5/TP63/AQP3/ACER1/KRT2/OVOL3/ESRP1/GRHL2/EXPH5/ALOX15B/SFRP4/LIPK/SHARPIN/KRT10/BMP4/ERCC3/WNT16/PPL/LIPM/KLF4/LCE3B/SPRR1A/TGM3/TCHH | 28 |
| BP | GO:0045637 | regulation of myeloid cell differentiation | 28/1200 | 210/18723 | 0.000186 | 0.005461 | 0.004538 | HCLS1/RASSF2/JAG1/CTNNBIP1/MYC/MEIS1/LILRB3/SCIN/TREM2/LYN/PTK2B/OCSTAMP/MPL/LILRB1/PIK3R1/FBN1/NCKAP1L/PITHD1/HSPA1B/IL17A/TESC/TYROBP/LTF/HSPA1A/ACVR1B/MITF/TNFSF11/PLA2G3 | 28 |
| BP | GO:0050920 | regulation of chemotaxis | 28/1200 | 223/18723 | 0.000504 | 0.010969 | 0.009116 | STAP1/WNK1/SLAMF1/STK39/AIF1/TREM2/CXCR4/LYN/PTK2B/GPR183/NOVA2/TIAM1/P2RX4/ROBO2/GPR18/WNT3/F2RL1/NCKAP1L/CCL5/PLA2G7/SEMA7A/PTN/C3AR1/NTF3/SCG2/CXCL13/S100A14/EDN2 | 28 |
| BP | GO:1901617 | organic hydroxy compound biosynthetic process | 28/1200 | 237/18723 | 0.001307 | 0.021909 | 0.018208 | PLCG2/CYB5R3/P2RY1/PLEK/OSBPL3/PTK2B/AKR1B1/PMVK/DCT/PCK1/ACER1/TYRP1/CYP7A1/FGFR4/FGF19/OSBPL6/ALOX15B/OSBPL1A/PPIP5K1/SLC27A2/STAR/SLC7A11/CYP39A1/PLTP/HMGCS1/BMP5/TYR/CES1 | 28 |
| BP | GO:1990266 | neutrophil migration | 27/1200 | 122/18723 | 1.09E-08 | 2.64E-06 | 2.19E-06 | VAV1/FUT7/PREX1/RHOH/PECAM1/DPEP1/CXCL1/CXADR/CXCL3/VAV3/CXCL2/NCKAP1L/TREM1/CCL5/PIK3CG/CXCL6/SAA1/CCL18/C3AR1/ADAM8/CXCL13/PIK3CD/CCL13/PDE4B/CXCR1/EDN2/PF4V1 | 27 |
| BP | GO:1902107 | positive regulation of leukocyte differentiation | 27/1200 | 157/18723 | 2.36E-06 | 0.000206 | 0.000171 | HCLS1/LILRB2/XBP1/CD86/CTNNBIP1/RHOH/TREM2/PTPRC/BTK/OCSTAMP/PCK1/NCKAP1L/CR1/IL7R/CD80/EGR3/IL17A/TESC/TYROBP/VNN1/IL2RA/ADAM8/CD46/TNFSF11/PLA2G3/ADA/TOX | 27 |
| BP | GO:1903708 | positive regulation of hemopoiesis | 27/1200 | 157/18723 | 2.36E-06 | 0.000206 | 0.000171 | HCLS1/LILRB2/XBP1/CD86/CTNNBIP1/RHOH/TREM2/PTPRC/BTK/OCSTAMP/PCK1/NCKAP1L/CR1/IL7R/CD80/EGR3/IL17A/TESC/TYROBP/VNN1/IL2RA/ADAM8/CD46/TNFSF11/PLA2G3/ADA/TOX | 27 |
| BP | GO:0002700 | regulation of production of molecular mediator of immune response | 27/1200 | 164/18723 | 5.54E-06 | 0.000387 | 0.000322 | MZB1/CD226/XBP1/PLCG2/CD86/SLAMF1/IL21/FCRL3/PTPRC/DNAJB9/BTK/IL1B/SCIMP/LILRB1/IL17F/FFAR2/TGFB3/F2RL1/CR1/IL17A/SEMA7A/FZD5/CD40LG/SLC7A5/KLK7/PGC/IL13RA2 | 27 |
| BP | GO:0071466 | cellular response to xenobiotic stimulus | 27/1200 | 177/18723 | 2.32E-05 | 0.001233 | 0.001025 | GSTA4/FBP1/AADAC/GSTA5/MYC/CXCR4/AIM2/DPEP1/IL1B/CYP2W1/ACSM1/CYP2C18/CYP2J2/AHRR/CYP3A7/CYP3A5/RORC/CYP4F12/STAR/GSTA1/ANKRD1/PDE4B/SULT2A1/GSTA2/UGT2B15/FMO2/UGT2B28 | 27 |
| BP | GO:0050731 | positive regulation of peptidyl-tyrosine phosphorylation | 27/1200 | 193/18723 | 0.000107 | 0.003542 | 0.002944 | HCLS1/CCK/PLCG2/BANK1/STAP1/ENPP2/IL24/IL21/FLT3/TREM2/PECAM1/HES5/LYN/GHR/PTPRC/PTK2B/EFNA5/LILRA5/EGF/CD80/CCL5/ARRB2/CLEC6A/NTF3/IL31RA/ADRA2A/CRLF1 | 27 |
| BP | GO:0045216 | cell-cell junction organization | 27/1200 | 200/18723 | 0.000196 | 0.005714 | 0.004749 | DSP/PKP3/CLDN10/PECAM1/PERP/JUP/POF1B/PKP1/IL1B/CXADR/PARD3/TGFB3/CLDN3/F2RL1/GJA1/IL17A/GRHL2/MARVELD2/OCLN/FZD5/PARD6A/CDH1/ACE2/DLG1/CDH18/WNT11/CLDN9 | 27 |
| BP | GO:0019722 | calcium-mediated signaling | 27/1200 | 202/18723 | 0.000231 | 0.00632 | 0.005253 | LAT2/CHP2/PLCG2/MAPT/PLEK/TREM2/P2RX5/CXCR4/PTPRC/PTK2B/BTK/P2RX4/SELP/P2RX7/TRAT1/SAMD14/BHLHA15/HTR2B/NFATC2/DEFB1/CCR3/TNFSF11/ADA/AGTR1/CXCR1/EDN2/NMUR1 | 27 |
| BP | GO:0006643 | membrane lipid metabolic process | 27/1200 | 203/18723 | 0.00025 | 0.0067 | 0.005568 | ST8SIA4/SPTSSB/PIGK/ENPP2/PGAP1/FUT7/ST3GAL1/B4GALT6/DEGS1/NEU4/ELOVL4/P2RX1/ST6GALNAC4/ACER1/GBGT1/PGAP2/ST3GAL2/CWH43/FUCA1/PSAPL1/B3GNT5/SGPP2/ELOVL2/PIGA/ALDH3B2/ELOVL6/PNPLA1 | 27 |
| BP | GO:0050679 | positive regulation of epithelial cell proliferation | 27/1200 | 207/18723 | 0.000344 | 0.008496 | 0.007061 | XBP1/MYDGF/CYBA/ITGA4/MYC/TBX1/DLX5/TP63/EGF/EGR3/FGFR2/FGFBP1/PTN/BMP4/HTR2B/SCG2/PIK3CD/CCR3/MMP12/NRARP/HAS2/AGTR1/SOX11/BMP5/ODAM/ECM1/STAT5A | 27 |
| BP | GO:0046777 | protein autophosphorylation | 27/1200 | 227/18723 | 0.00143 | 0.023519 | 0.019546 | STK17B/RASSF2/WNK1/STK39/GPNMB/STK33/ERN1/FLT3/WNK3/PIM2/LYN/PTPRC/PTK2B/BTK/MAP4K1/EPHB3/ENG/FGR/GRK5/ITK/SLK/FGFR4/FGFR2/MEX3B/ACVR1B/MAP3K9/NTRK2 | 27 |
| BP | GO:0071695 | anatomical structure maturation | 27/1200 | 229/18723 | 0.001627 | 0.026268 | 0.021831 | BFSP2/IL21/B4GALT6/HES5/PTK2B/NPPC/AKR1B1/BTK/PLXNB1/NRCAM/PPARG/C3/FZD5/SOX18/LTF/BHLHA15/ROPN1B/ROPN1/C1QA/DEFB1/FERMT1/SPINK1/PLA2G3/LHX6/PAEP/RET/CNGB1 | 27 |
| BP | GO:0045055 | regulated exocytosis | 27/1200 | 230/18723 | 0.001734 | 0.02741 | 0.02278 | LAT2/CADPS2/ARL8B/P2RY1/RAB11B/PLEK/SCIN/LYN/BTK/PRKCB/P2RX1/UNC13B/FGR/F2RL1/SCAMP5/NCKAP1L/STXBP5/PIK3CG/RAB11FIP2/MILR1/CPLX3/PIK3CD/PLA2G3/IL13RA2/PCLO/ADRA2A/SYN1 | 27 |
| BP | GO:2000116 | regulation of cysteine-type endopeptidase activity | 27/1200 | 235/18723 | 0.002363 | 0.034021 | 0.028274 | CCK/MAPT/NLRC4/MYC/SOX2/CST7/AIM2/PERP/DPEP1/BIRC3/HERPUD1/TP63/COL4A3/P2RX1/CRYAB/MMP9/CASP10/PPARG/LCK/ARRB2/DNAJB6/LTF/NLRP6/KLF4/FOXL2/CIDEB/SIAH2 | 27 |
| BP | GO:0002224 | toll-like receptor signaling pathway | 26/1200 | 121/18723 | 3.87E-08 | 7.34E-06 | 6.10E-06 | CTSS/LY96/IRF4/PLCG2/TLR10/MFHAS1/CYBA/TREM2/LYN/FCRL3/LGR4/TLR2/BTK/CD14/BIRC3/SCIMP/TLR1/F2RL1/RAB11FIP2/ARRB2/LTF/NLRP6/S100A14/IRAK2/TNIP3/LILRA2 | 26 |
| BP | GO:0071621 | granulocyte chemotaxis | 26/1200 | 125/18723 | 7.77E-08 | 1.25E-05 | 1.04E-05 | VAV1/SLAMF1/PREX1/DPEP1/CXCL1/CXADR/CXCL3/VAV3/CXCL2/NCKAP1L/TREM1/CCL5/PIK3CG/CXCL6/SAA1/CCL18/C3AR1/SCG2/CXCL13/S100A14/PIK3CD/CCL13/PDE4B/CXCR1/EDN2/PF4V1 | 26 |
| BP | GO:0042098 | T cell proliferation | 26/1200 | 199/18723 | 0.000427 | 0.009746 | 0.008099 | LILRB2/CD86/SLAMF1/RASAL3/GPNMB/AIF1/IL21/CTLA4/CLECL1/PRKCQ/PTPRC/IL1B/LILRB1/NCKAP1L/CR1/CD80/CCL5/PIK3CG/BTN3A1/PLA2G2D/IL2RA/BMP4/CD40LG/CD46/DLG1/TNFSF8 | 26 |
| BP | GO:0043491 | protein kinase B signaling | 26/1200 | 211/18723 | 0.001039 | 0.018474 | 0.015354 | HCLS1/TPTE2/BANK1/MYDGF/IL26/MST1R/MFHAS1/PLEKHA1/IL1B/ENG/P2RX4/EGF/ADTRP/CCL5/PIK3CG/ARRB2/FAM110C/ADAM8/PIK3CD/MERTK/DLG1/TNFSF11/RET/KLF4/SFRP5/NTRK2 | 26 |
| BP | GO:0045088 | regulation of innate immune response | 26/1200 | 218/18723 | 0.001668 | 0.02665 | 0.022149 | CD226/PLCG2/VAV1/NLRC4/ZBP1/SUSD4/IL21/TREM2/LYN/AIM2/BIRC3/LILRB1/FGR/FFAR2/FCN1/PAK3/CR1/CCL5/PPARG/ARRB2/TYROBP/MNDA/CLEC6A/ADAM8/MMP12/LILRA2 | 26 |
| BP | GO:0050853 | B cell receptor signaling pathway | 25/1200 | 131/18723 | 7.70E-07 | 8.34E-05 | 6.93E-05 | CD19/LAT2/CD38/KLHL6/PLCG2/BANK1/CD79A/STAP1/PLEKHA1/CTLA4/LYN/FCRL3/IGLL1/PTPRC/BTK/PRKCB/VAV3/MS4A1/LPXN/ITK/NCKAP1L/LCK/MNDA/NFATC2/PIK3CD | 25 |
| BP | GO:0030183 | B cell differentiation | 25/1200 | 141/18723 | 3.14E-06 | 0.000253 | 0.00021 | CD19/CR2/POU2AF1/XBP1/PLCG2/IKZF3/CD79A/ITGA4/IL21/FLT3/ST3GAL1/FCRL3/PTPRC/PTK2B/GPR183/DNAJB9/BTK/TCIRG1/PIK3R1/MS4A1/NCKAP1L/CR1/CD40LG/ADA/KLF6 | 25 |
| BP | GO:0070665 | positive regulation of leukocyte proliferation | 25/1200 | 150/18723 | 9.77E-06 | 0.000611 | 0.000508 | LILRB2/CD38/CD86/SLAMF1/RASAL3/AIF1/IL21/CLECL1/LYN/FCRL3/PRKCQ/PTPRC/GPR183/OCSTAMP/IL1B/MPL/VAV3/NCKAP1L/CD80/CCL5/IL2RA/CD40LG/NFATC2/CD46/ADA | 25 |
| BP | GO:0002819 | regulation of adaptive immune response | 25/1200 | 183/18723 | 0.000276 | 0.007229 | 0.006008 | SAMSN1/CD226/CR2/SLAMF1/CR1L/SUSD4/FUT7/SKAP1/TREM2/PRKCQ/PTPRC/BTK/IL1B/LILRB1/NCKAP1L/CR1/IL7R/CD80/C3/FZD5/CLEC6A/CD46/ADA/AZGP1/CD1E | 25 |
| BP | GO:0071706 | tumor necrosis factor superfamily cytokine production | 25/1200 | 186/18723 | 0.000355 | 0.008557 | 0.007111 | LY96/PLCG2/CD86/SLAMF1/CYBA/GPNMB/UBE2J1/TREM2/CYBB/PTPRC/TLR2/CD14/LILRB1/IL17F/TLR1/GPR18/PIK3R1/LILRA5/IL17A/ARRB2/TYROBP/FZD5/LTF/ADAM8/LILRA2 | 25 |
| BP | GO:1903555 | regulation of tumor necrosis factor superfamily cytokine production | 25/1200 | 186/18723 | 0.000355 | 0.008557 | 0.007111 | LY96/PLCG2/CD86/SLAMF1/CYBA/GPNMB/UBE2J1/TREM2/CYBB/PTPRC/TLR2/CD14/LILRB1/IL17F/TLR1/GPR18/PIK3R1/LILRA5/IL17A/ARRB2/TYROBP/FZD5/LTF/ADAM8/LILRA2 | 25 |
| BP | GO:0002285 | lymphocyte activation involved in immune response | 25/1200 | 194/18723 | 0.000671 | 0.013242 | 0.011005 | CD19/POU2AF1/IRF4/XBP1/PLCG2/CD86/CD180/BATF/IL21/ST3GAL1/PTPRC/PTK2B/GPR183/LILRB1/PCK1/F2RL1/NCKAP1L/CR1/CD80/GAPT/RORC/CD40LG/CD46/EOMES/ADA | 25 |
| BP | GO:0060348 | bone development | 25/1200 | 205/18723 | 0.001487 | 0.024302 | 0.020197 | SULF2/MEIS1/GHR/PTPRC/DLX5/NPPC/ENG/LILRB1/ZNF385A/TGFB3/PLXNB1/ANXA6/FBN1/GJA1/TYROBP/SFRP4/FGFR2/LTF/BMP4/TULP3/TNFSF11/HAS2/MMP13/NEUROG1/TGM2 | 25 |
| CC | GO:0031225 | anchored component of membrane | 25/1263 | 170/19550 | 9.65E-05 | 0.003845 | 0.00346 | VNN2/RAB3C/RAB11B/RAB3B/LY6K/DPEP1/EFNA5/CD14/GP1BA/LYPD6B/CD52/TEX101/ENPP6/SEMA7A/VNN1/LYPD6/SVIP/CPLX3/CNTN3/EFNA3/LYPD3/GAD2/SYN1/MDGA2/EEPD1 | 25 |
| MF | GO:0140375 | immune receptor activity | 25/1229 | 144/18368 | 9.78E-06 | 0.001221 | 0.001102 | LILRB2/CR2/FLT3/LILRB3/CXCR4/GHR/MPL/LILRB1/LILRA5/LILRA6/CR1/IL7R/LILRA1/IL22RA2/IL2RA/C3AR1/IL2RG/IL12RB2/CCR3/FPR1/IL13RA2/IL31RA/CXCR1/LILRA2/CRLF1 | 25 |
| MF | GO:0061135 | endopeptidase regulator activity | 25/1229 | 194/18368 | 0.001231 | 0.038978 | 0.035174 | SERPINI1/APH1B/NLRC4/CST7/DPEP1/BIRC3/COL4A3/RARRES1/SERPINB5/A2ML1/C3/SERPINA11/LTF/SERPINB13/SERPINB11/KNG1/SPINK1/SERPINB7/SPOCK1/SERPINI2/TFPI2/TIMP1/SMR3B/SPINK7/SPINK9 | 25 |
| BP | GO:0042100 | B cell proliferation | 24/1200 | 99/18723 | 1.07E-08 | 2.64E-06 | 2.19E-06 | CD19/MZB1/CR2/CD38/CD180/IKZF3/CD79A/IL21/CTLA4/LYN/FCRL3/PTPRC/GPR183/BTK/VAV3/MS4A1/NCKAP1L/IL7R/GAPT/TYROBP/MNDA/CD40LG/NFATC2/ADA | 24 |
| BP | GO:0035966 | response to topologically incorrect protein | 24/1200 | 159/18723 | 7.67E-05 | 0.002857 | 0.002374 | XBP1/ERLEC1/EDEM1/HSPA2/ERN1/HSPA13/RHBDD1/SDF2L1/F12/CTH/DNAJB9/HERPUD1/UGGT1/PIK3R1/DNAJB1/HSPA1B/CREB3L2/DNAJC3/AGR2/CHAC1/BHLHA15/HSPA1A/HSPA4L/HSPA1L | 24 |
| BP | GO:0042129 | regulation of T cell proliferation | 24/1200 | 171/18723 | 0.000241 | 0.006531 | 0.005428 | LILRB2/CD86/SLAMF1/RASAL3/GPNMB/AIF1/IL21/CTLA4/CLECL1/PRKCQ/PTPRC/IL1B/LILRB1/NCKAP1L/CR1/CD80/CCL5/PLA2G2D/IL2RA/BMP4/CD40LG/CD46/DLG1/TNFSF8 | 24 |
| BP | GO:0001659 | temperature homeostasis | 24/1200 | 174/18723 | 0.000314 | 0.007939 | 0.006598 | IRF4/NPR3/CXCR4/UCP1/ADRB2/LGR4/NOVA2/IL1B/ALDH1A1/GJA1/FABP4/ABHD6/GATM/UCP2/SCD/TNFSF11/PLAC8/ELOVL6/G0S2/TSHR/EDN2/ESRRG/ADRB1/SLN | 24 |
| BP | GO:0045619 | regulation of lymphocyte differentiation | 24/1200 | 174/18723 | 0.000314 | 0.007939 | 0.006598 | LILRB2/IRF4/XBP1/CD86/IKZF3/RHOH/CTLA4/FCRL3/PTPRC/BTK/PCK1/NCKAP1L/CR1/IL7R/CD80/EGR3/VNN1/IL2RA/ADAM8/BMP4/CD46/NRARP/ADA/TOX | 24 |
| BP | GO:0048017 | inositol lipid-mediated signaling | 24/1200 | 182/18723 | 0.000611 | 0.012524 | 0.010408 | HCLS1/TPTE2/MYDGF/PIK3C2G/NPR3/PLEKHA1/FLT3/TREM2/PLD1/SELP/FGR/PIK3R1/PLCD3/PLXNB1/F2RL1/EGF/CCL5/PIK3CG/HTR2B/PIK3CD/WNT16/KLF4/F2/NTRK2 | 24 |
| BP | GO:0050864 | regulation of B cell activation | 24/1200 | 198/18723 | 0.001985 | 0.029952 | 0.024893 | CD19/MZB1/SAMSN1/CD38/XBP1/BANK1/IKZF3/SH3KBP1/IL21/CTLA4/LYN/FCRL3/IGLL1/PTPRC/GPR183/BTK/VAV3/THEMIS2/NCKAP1L/CR1/TYROBP/MNDA/NFATC2/ADA | 24 |
| BP | GO:0030593 | neutrophil chemotaxis | 23/1200 | 103/18723 | 1.11E-07 | 1.68E-05 | 1.40E-05 | VAV1/PREX1/DPEP1/CXCL1/CXADR/CXCL3/VAV3/CXCL2/NCKAP1L/TREM1/CCL5/PIK3CG/CXCL6/SAA1/CCL18/C3AR1/CXCL13/PIK3CD/CCL13/PDE4B/CXCR1/EDN2/PF4V1 | 23 |
| BP | GO:0050671 | positive regulation of lymphocyte proliferation | 23/1200 | 137/18723 | 1.94E-05 | 0.001073 | 0.000892 | LILRB2/CD38/CD86/SLAMF1/RASAL3/AIF1/IL21/CLECL1/FCRL3/PRKCQ/PTPRC/GPR183/IL1B/MPL/VAV3/NCKAP1L/CD80/CCL5/IL2RA/CD40LG/NFATC2/CD46/ADA | 23 |
| BP | GO:0032946 | positive regulation of mononuclear cell proliferation | 23/1200 | 138/18723 | 2.19E-05 | 0.001174 | 0.000976 | LILRB2/CD38/CD86/SLAMF1/RASAL3/AIF1/IL21/CLECL1/FCRL3/PRKCQ/PTPRC/GPR183/IL1B/MPL/VAV3/NCKAP1L/CD80/CCL5/IL2RA/CD40LG/NFATC2/CD46/ADA | 23 |
| BP | GO:0046631 | alpha-beta T cell activation | 23/1200 | 156/18723 | 0.000154 | 0.004728 | 0.00393 | IRF4/CD86/BATF/RASAL3/IL21/FUT7/PRKCQ/PTPRC/GPR183/PSMB11/LILRB1/TCIRG1/GPR18/ITK/NCKAP1L/CTSL/CD80/PLA2G2D/RORC/EOMES/ADA/TNFSF8/TOX | 23 |
| BP | GO:0051250 | negative regulation of lymphocyte activation | 23/1200 | 157/18723 | 0.00017 | 0.005122 | 0.004257 | SAMSN1/LILRB2/CD86/BANK1/GPNMB/CTLA4/LYN/BTK/LILRB1/FGR/NCKAP1L/LAX1/CR1/CD80/TYROBP/MNDA/PLA2G2D/IL2RA/BMP4/MERTK/DLG1/NRARP/SOX11 | 23 |
| BP | GO:0002822 | regulation of adaptive immune response based on somatic recombination of immune receptors built from immunoglobulin superfamily domains | 23/1200 | 168/18723 | 0.000462 | 0.010176 | 0.008457 | CD226/CR2/SLAMF1/CR1L/SUSD4/FUT7/TREM2/PRKCQ/PTPRC/BTK/IL1B/LILRB1/NCKAP1L/CR1/IL7R/CD80/C3/FZD5/CLEC6A/CD46/ADA/AZGP1/CD1E | 23 |
| BP | GO:0048469 | cell maturation | 23/1200 | 171/18723 | 0.000594 | 0.012239 | 0.010172 | BFSP2/IL21/B4GALT6/HES5/PTK2B/NPPC/AKR1B1/BTK/NRCAM/PPARG/C3/FZD5/SOX18/BHLHA15/ROPN1B/ROPN1/C1QA/DEFB1/SPINK1/PLA2G3/LHX6/PAEP/RET | 23 |
| BP | GO:0009266 | response to temperature stimulus | 23/1200 | 178/18723 | 0.00104 | 0.018474 | 0.015354 | MAPT/HSPA2/CXCR4/LYN/UCP1/ADRB2/CD14/DNAJB1/HSPA1B/ARRB2/DNAJB6/UCP2/DNAJC3/GCLC/HTR2B/HSPA1A/GRIK2/TP53INP1/TRPA1/PLAC8/ADRB1/SST/DNAJA4 | 23 |
| BP | GO:0048015 | phosphatidylinositol-mediated signaling | 23/1200 | 178/18723 | 0.00104 | 0.018474 | 0.015354 | HCLS1/TPTE2/MYDGF/PIK3C2G/NPR3/PLEKHA1/FLT3/TREM2/SELP/FGR/PIK3R1/PLCD3/PLXNB1/F2RL1/EGF/CCL5/PIK3CG/HTR2B/PIK3CD/WNT16/KLF4/F2/NTRK2 | 23 |
| BP | GO:0022408 | negative regulation of cell-cell adhesion | 23/1200 | 196/18723 | 0.003674 | 0.046871 | 0.038954 | LILRB2/CD86/WNK1/AKNA/GPNMB/JAG1/CTLA4/PODXL/LILRB1/NCKAP1L/LAX1/ADTRP/CR1/CD80/PLA2G2D/IL2RA/BMP4/CDH1/MIA3/EPCAM/DLG1/NRARP/KLF4 | 23 |
| MF | GO:0031406 | carboxylic acid binding | 23/1229 | 176/18368 | 0.00157 | 0.042817 | 0.038639 | ST8SIA4/SIGLEC5/APOC1/ALOX5AP/SIGLEC12/CRABP2/UCP1/CYP2W1/SELP/ACOXL/PCK1/FCN1/FABP4/PPARG/GPR31/SHMT1/GCLC/GSTA1/CYP4F11/SIGLEC10/GLUD2/GAD2/GAD1 | 23 |
| MF | GO:0004866 | endopeptidase inhibitor activity | 23/1229 | 180/18368 | 0.002113 | 0.052429 | 0.047313 | SERPINI1/CST7/DPEP1/BIRC3/COL4A3/RARRES1/SERPINB5/A2ML1/C3/SERPINA11/LTF/SERPINB13/SERPINB11/KNG1/SPINK1/SERPINB7/SPOCK1/SERPINI2/TFPI2/TIMP1/SMR3B/SPINK7/SPINK9 | 23 |
| BP | GO:0072676 | lymphocyte migration | 22/1200 | 117/18723 | 4.49E-06 | 0.000336 | 0.000279 | MYO1G/WNK1/STK39/ITGA4/AIF1/FUT7/CD200/PTK2B/GPR183/C10orf99/ADTRP/CCL5/PIK3CG/SAA1/CCL18/ADAM8/CXCL13/MIA3/PIK3CD/RET/CCL13/ECM1 | 22 |
| BP | GO:0046467 | membrane lipid biosynthetic process | 22/1200 | 142/18723 | 0.000102 | 0.003427 | 0.002848 | ST8SIA4/SPTSSB/PIGK/PGAP1/ST3GAL1/B4GALT6/DEGS1/ELOVL4/P2RX1/ST6GALNAC4/ACER1/GBGT1/PGAP2/ST3GAL2/CWH43/B3GNT5/SGPP2/ELOVL2/PIGA/ALDH3B2/ELOVL6/PNPLA1 | 22 |
| BP | GO:1990845 | adaptive thermogenesis | 22/1200 | 157/18723 | 0.000439 | 0.009929 | 0.008252 | IRF4/NPR3/CXCR4/UCP1/ADRB2/LGR4/NOVA2/ALDH1A1/MC4R/GJA1/FABP4/ABHD6/GATM/UCP2/SCD/PLAC8/ELOVL6/G0S2/TSHR/ESRRG/ADRB1/SLN | 22 |
| BP | GO:0032635 | interleukin-6 production | 22/1200 | 165/18723 | 0.000871 | 0.016579 | 0.013779 | LILRB2/POU2AF1/XBP1/PLCG2/BANK1/SLAMF1/CYBA/AIF1/CD200/TREM2/TLR2/IL1B/SCIMP/IL17F/TLR1/LILRA5/F2RL1/NCKAP1L/IL17A/ARRB2/TYROBP/LILRA2 | 22 |
| BP | GO:0032675 | regulation of interleukin-6 production | 22/1200 | 165/18723 | 0.000871 | 0.016579 | 0.013779 | LILRB2/POU2AF1/XBP1/PLCG2/BANK1/SLAMF1/CYBA/AIF1/CD200/TREM2/TLR2/IL1B/SCIMP/IL17F/TLR1/LILRA5/F2RL1/NCKAP1L/IL17A/ARRB2/TYROBP/LILRA2 | 22 |
| BP | GO:1903034 | regulation of response to wounding | 22/1200 | 167/18723 | 0.001024 | 0.018474 | 0.015354 | XBP1/KRT1/CXCR4/F12/PTPRF/GP1BA/KREMEN1/CLDN3/KANK1/F2RL1/ENPP4/GJA1/ADTRP/OCLN/PTN/FERMT1/KNG1/TSPAN8/ADRA2A/SIGLEC10/KLF4/F2 | 22 |
| BP | GO:0002706 | regulation of lymphocyte mediated immunity | 22/1200 | 168/18723 | 0.001108 | 0.019364 | 0.016093 | CD226/CR2/VAV1/SLAMF1/CR1L/SUSD4/IL21/FUT7/TREM2/PTPRC/BTK/IL1B/LILRB1/NCKAP1L/CR1/IL7R/C3/ARRB2/FZD5/CD46/AZGP1/CD1E | 22 |
| BP | GO:0006937 | regulation of muscle contraction | 22/1200 | 169/18723 | 0.001199 | 0.020476 | 0.017017 | DSP/DSC2/TNNT3/NPY2R/JUP/ADRB2/P2RX4/P2RX1/ANXA6/KCNJ2/PIK3CG/TNNT1/CHRNA3/PPP1R12B/PROK2/ACE2/DLG1/ADA/ADRA2A/PDE4B/EDN2/RNF207 | 22 |
| BP | GO:0050728 | negative regulation of inflammatory response | 22/1200 | 176/18723 | 0.00203 | 0.030201 | 0.0251 | TNFAIP6/KRT1/MFHAS1/CD200/TREM2/CST7/LYN/PTPRC/FGR/IL22RA2/PPARG/SAA1/LRFN5/GPR31/IL2RA/SHARPIN/NLRP6/NR1D2/PLK2/ADA/SIGLEC10/KLF4 | 22 |
| BP | GO:0032640 | tumor necrosis factor production | 22/1200 | 181/18723 | 0.002887 | 0.039116 | 0.032509 | LY96/PLCG2/SLAMF1/CYBA/GPNMB/UBE2J1/TREM2/CYBB/PTPRC/TLR2/CD14/LILRB1/TLR1/GPR18/PIK3R1/LILRA5/IL17A/ARRB2/TYROBP/FZD5/LTF/LILRA2 | 22 |
| BP | GO:0032680 | regulation of tumor necrosis factor production | 22/1200 | 181/18723 | 0.002887 | 0.039116 | 0.032509 | LY96/PLCG2/SLAMF1/CYBA/GPNMB/UBE2J1/TREM2/CYBB/PTPRC/TLR2/CD14/LILRB1/TLR1/GPR18/PIK3R1/LILRA5/IL17A/ARRB2/TYROBP/FZD5/LTF/LILRA2 | 22 |
| BP | GO:0051896 | regulation of protein kinase B signaling | 22/1200 | 185/18723 | 0.003775 | 0.047655 | 0.039605 | HCLS1/TPTE2/BANK1/MYDGF/IL26/MST1R/MFHAS1/PLEKHA1/ENG/P2RX4/EGF/PIK3CG/ARRB2/FAM110C/ADAM8/PIK3CD/DLG1/TNFSF11/RET/KLF4/SFRP5/NTRK2 | 22 |
| BP | GO:0032963 | collagen metabolic process | 21/1200 | 104/18723 | 2.25E-06 | 0.000203 | 0.000168 | CTSS/MMP3/MMP10/MMP7/NPPC/ENG/TNS2/MMP1/MMP9/ADAMTS3/TGFB3/MMP27/CTSL/CYP7A1/ARRB2/BMP4/TRAM2/MMP12/SERPINB7/MMP13/F2 | 21 |
| BP | GO:0002702 | positive regulation of production of molecular mediator of immune response | 21/1200 | 117/18723 | 1.55E-05 | 0.000898 | 0.000746 | MZB1/CD226/XBP1/PLCG2/CD86/SLAMF1/IL21/PTPRC/DNAJB9/IL1B/SCIMP/LILRB1/IL17F/FFAR2/F2RL1/IL17A/SEMA7A/FZD5/SLC7A5/KLK7/PGC | 21 |
| BP | GO:0001909 | leukocyte mediated cytotoxicity | 21/1200 | 124/18723 | 3.85E-05 | 0.001746 | 0.001451 | SLAMF7/CD226/VAV1/ARL8B/STAP1/IL21/PTPRC/PRF1/LILRB1/F2RL1/NCKAP1L/IL7R/TREM1/CXCL6/ARRB2/TYROBP/GZMB/NLRP6/AZGP1/CD1E/F2 | 21 |
| BP | GO:0006986 | response to unfolded protein | 21/1200 | 137/18723 | 0.000169 | 0.005122 | 0.004257 | XBP1/ERLEC1/EDEM1/HSPA2/ERN1/HSPA13/RHBDD1/CTH/DNAJB9/HERPUD1/PIK3R1/DNAJB1/HSPA1B/CREB3L2/DNAJC3/AGR2/CHAC1/BHLHA15/HSPA1A/HSPA4L/HSPA1L | 21 |
| BP | GO:0030216 | keratinocyte differentiation | 21/1200 | 139/18723 | 0.000208 | 0.005895 | 0.004899 | DSP/KRT16/JAG1/IRF6/TP63/AQP3/ACER1/KRT2/GRHL2/EXPH5/ALOX15B/LIPK/SHARPIN/KRT10/WNT16/PPL/LIPM/LCE3B/SPRR1A/TGM3/TCHH | 21 |
| BP | GO:0050729 | positive regulation of inflammatory response | 21/1200 | 142/18723 | 0.00028 | 0.007277 | 0.006048 | PLCG2/STAP1/ZBP1/TLR10/IL21/ALOX5AP/TREM2/TLR2/BTK/IL1B/FFAR2/LILRA5/FABP4/PIK3CG/C3/PLA2G7/ADAM8/TNFSF11/PLA2G3/AGTR1/TGM2 | 21 |
| BP | GO:0014065 | phosphatidylinositol 3-kinase signaling | 21/1200 | 144/18723 | 0.000341 | 0.008492 | 0.007058 | HCLS1/TPTE2/MYDGF/PIK3C2G/PLEKHA1/FLT3/TREM2/SELP/FGR/PIK3R1/PLXNB1/F2RL1/EGF/CCL5/PIK3CG/HTR2B/PIK3CD/WNT16/KLF4/F2/NTRK2 | 21 |
| BP | GO:0106106 | cold-induced thermogenesis | 21/1200 | 144/18723 | 0.000341 | 0.008492 | 0.007058 | IRF4/NPR3/CXCR4/UCP1/ADRB2/LGR4/NOVA2/ALDH1A1/GJA1/FABP4/ABHD6/GATM/UCP2/SCD/PLAC8/ELOVL6/G0S2/TSHR/ESRRG/ADRB1/SLN | 21 |
| BP | GO:0120161 | regulation of cold-induced thermogenesis | 21/1200 | 144/18723 | 0.000341 | 0.008492 | 0.007058 | IRF4/NPR3/CXCR4/UCP1/ADRB2/LGR4/NOVA2/ALDH1A1/GJA1/FABP4/ABHD6/GATM/UCP2/SCD/PLAC8/ELOVL6/G0S2/TSHR/ESRRG/ADRB1/SLN | 21 |
| BP | GO:0045580 | regulation of T cell differentiation | 21/1200 | 146/18723 | 0.000412 | 0.009475 | 0.007874 | LILRB2/IRF4/XBP1/CD86/RHOH/CTLA4/PTPRC/PCK1/NCKAP1L/CR1/IL7R/CD80/EGR3/VNN1/IL2RA/ADAM8/BMP4/CD46/NRARP/ADA/TOX | 21 |
| BP | GO:0031214 | biomineral tissue development | 21/1200 | 169/18723 | 0.002724 | 0.037195 | 0.030912 | SRGN/TUFT1/GPNMB/TBX1/ADRB2/PTK2B/LGR4/MGP/TCIRG1/P2RX7/FGR/TGFB3/FGFR2/LTF/PTN/BMP4/STATH/WNT11/MMP13/ODAM/ECM1 | 21 |
| BP | GO:0110148 | biomineralization | 21/1200 | 171/18723 | 0.003139 | 0.041996 | 0.034903 | SRGN/TUFT1/GPNMB/TBX1/ADRB2/PTK2B/LGR4/MGP/TCIRG1/P2RX7/FGR/TGFB3/FGFR2/LTF/PTN/BMP4/STATH/WNT11/MMP13/ODAM/ECM1 | 21 |
| BP | GO:0052126 | movement in host environment | 21/1200 | 175/18723 | 0.004128 | 0.049664 | 0.041275 | CR2/CD86/ARL8B/SLAMF1/TRIM35/CXCR4/CXADR/FCN1/CR1/MID2/CTSL/CD80/CLEC4M/HSPA1B/FCN3/HSPA1A/ACE2/CD46/AGTR1/SIGLEC1/CLDN9 | 21 |
| BP | GO:0043405 | regulation of MAP kinase activity | 21/1200 | 177/18723 | 0.004713 | 0.053042 | 0.044082 | MST1R/ERN1/FLT3/LYN/GHR/PTK2B/TIAM1/IL1B/EGF/LAX1/PPARG/PIK3CG/FZD5/ADAM8/NTF3/BMP4/HTR2B/AJUBA/TNFSF11/ADRA2A/FZD10 | 21 |
| BP | GO:0006941 | striated muscle contraction | 21/1200 | 179/18723 | 0.005365 | 0.057224 | 0.047558 | DSP/JSRP1/DSC2/TNNT3/RCSD1/CXCR4/JUP/P2RX4/KCNJ8/PPP1R13L/KCNA5/GJA1/KCNJ2/PIK3CG/TNNT1/STAC2/ACE2/DLG1/PDE4B/MYL1/RNF207 | 21 |
| BP | GO:0002526 | acute inflammatory response | 20/1200 | 112/18723 | 2.66E-05 | 0.001329 | 0.001105 | CTNNBIP1/FUT7/ALOX5AP/F12/BTK/IL1B/FFAR2/SAA2/TREM1/PIK3CG/C3/SAA1/PLA2G2D/VNN1/ADAM8/NLRP6/TNFSF11/MBL2/IL31RA/F2 | 20 |
| BP | GO:0030168 | platelet activation | 20/1200 | 123/18723 | 0.000105 | 0.003502 | 0.00291 | PLCG2/VAV1/P2RY1/PLEK/LYN/PRKCQ/MPL/GP1BA/SELP/P2RX1/VAV3/PIK3CG/LCK/SAA1/CD40LG/MERTK/SLC7A11/ADRA2A/PF4V1/F2 | 20 |
| BP | GO:0002687 | positive regulation of leukocyte migration | 20/1200 | 135/18723 | 0.000379 | 0.008897 | 0.007394 | WNK1/SLAMF1/STK39/ITGA4/AIF1/TREM2/PTK2B/P2RX4/SELP/F2RL1/NCKAP1L/CCL5/PLA2G7/PTN/C3AR1/ADAM8/CXCL13/MIA3/S100A14/EDN2 | 20 |
| BP | GO:0050921 | positive regulation of chemotaxis | 20/1200 | 141/18723 | 0.00067 | 0.013242 | 0.011005 | WNK1/SLAMF1/STK39/AIF1/TREM2/CXCR4/PTK2B/TIAM1/P2RX4/F2RL1/NCKAP1L/CCL5/PLA2G7/PTN/C3AR1/NTF3/SCG2/CXCL13/S100A14/EDN2 | 20 |
| BP | GO:0007043 | cell-cell junction assembly | 20/1200 | 146/18723 | 0.001045 | 0.018504 | 0.015379 | PKP3/CLDN10/PECAM1/JUP/POF1B/PKP1/IL1B/PARD3/CLDN3/GJA1/IL17A/GRHL2/MARVELD2/OCLN/FZD5/ACE2/DLG1/CDH18/WNT11/CLDN9 | 20 |
| BP | GO:0045834 | positive regulation of lipid metabolic process | 20/1200 | 149/18723 | 0.001347 | 0.022427 | 0.018639 | CD19/AADAC/APOC1/FLT3/AGAP2/LYN/PTK2B/IL1B/VAV3/FGR/PPARG/CYP7A1/ABHD6/HTR2B/STAR/PLA2G3/AGTR1/CES1/F2/CGA | 20 |
| BP | GO:0120254 | olefinic compound metabolic process | 20/1200 | 153/18723 | 0.001862 | 0.028922 | 0.024037 | BCO1/AKR1B1/ALDH1A1/ABHD6/RDH12/ALOX15B/CYP2C18/CYP2J2/CYP3A7/CYP3A5/CYP4F12/ADH7/STAR/AKR1B10/ELOVL2/GSTA1/CYP4F11/PLA2G10/PNLIP/BMP5 | 20 |
| CC | GO:0072562 | blood microparticle | 20/1263 | 146/19550 | 0.001153 | 0.031831 | 0.028642 | KRT1/JCHAIN/HSPA2/SLC2A1/TMPRSS13/ZBTB38/ENG/FCN2/CFH/ACSM1/HSPA1B/C3/FCN3/BCHE/HSPA1A/HSPA1L/KNG1/C1QB/OAZ3/F2 | 20 |
| MF | GO:0019199 | transmembrane receptor protein kinase activity | 20/1229 | 143/18368 | 0.001355 | 0.040129 | 0.036213 | MST1R/FLT3/LYN/PTK2B/BTK/EPHB3/ENG/FGR/MATK/ITK/FGFR4/LCK/FGFR2/ACVR1B/WEE1/EFNA3/MERTK/RET/TGFBR3/NTRK2 | 20 |
| BP | GO:0031341 | regulation of cell killing | 19/1200 | 99/18723 | 1.46E-05 | 0.000852 | 0.000708 | CD226/VAV1/STAP1/CR1L/IL21/PTPRC/PRF1/LILRB1/CFH/P2RX7/F2RL1/NCKAP1L/IL7R/CXCL6/ARRB2/TYROBP/BCL2L1/AZGP1/CD1E | 19 |
| BP | GO:0042116 | macrophage activation | 19/1200 | 106/18723 | 3.97E-05 | 0.001772 | 0.001473 | PLCG2/MAPT/STAP1/MFHAS1/AIF1/CD200/TREM2/CST7/PTPRC/TLR2/TLR1/SPACA3/TYROBP/LRFN5/C1QA/PLA2G3/PLA2G10/IL31RA/EDN2 | 19 |
| BP | GO:0006805 | xenobiotic metabolic process | 19/1200 | 111/18723 | 7.63E-05 | 0.002857 | 0.002374 | GSTA4/AADAC/GSTA5/CYP2W1/ACSM1/CYP2C18/CYP2J2/AHRR/CYP3A7/CYP3A5/RORC/CYP4F12/STAR/GSTA1/SULT2A1/GSTA2/UGT2B15/FMO2/UGT2B28 | 19 |
| BP | GO:0071675 | regulation of mononuclear cell migration | 19/1200 | 115/18723 | 0.000124 | 0.00403 | 0.003349 | WNK1/SLAMF1/STK39/ITGA4/AIF1/CD200/LYN/PTK2B/C10orf99/ADTRP/CCL5/PLA2G7/C3AR1/ADAM8/CXCL13/MIA3/S100A14/BMP5/ECM1 | 19 |
| BP | GO:0033559 | unsaturated fatty acid metabolic process | 19/1200 | 116/18723 | 0.00014 | 0.004401 | 0.003657 | CES2/DEGS1/AKR1B1/IL1B/ELOVL4/CYP4F8/ABHD6/ALOX15B/CYP2C18/CYP2J2/CYP4F12/ELOVL2/GSTA1/SCD/CYP4F11/PLA2G3/PLA2G10/ELOVL6/EDN2 | 19 |
| BP | GO:0035296 | regulation of tube diameter | 19/1200 | 141/18723 | 0.001676 | 0.02665 | 0.022149 | CD38/NPR3/P2RY1/SLC6A4/ADRB2/P2RX1/F2RL1/KCNA5/GJA1/GCLC/HTR2B/ACE2/UTS2/KNG1/HTR7/AGTR1/ADRA2A/EDN2/ADRB1 | 19 |
| BP | GO:0097746 | blood vessel diameter maintenance | 19/1200 | 141/18723 | 0.001676 | 0.02665 | 0.022149 | CD38/NPR3/P2RY1/SLC6A4/ADRB2/P2RX1/F2RL1/KCNA5/GJA1/GCLC/HTR2B/ACE2/UTS2/KNG1/HTR7/AGTR1/ADRA2A/EDN2/ADRB1 | 19 |
| BP | GO:0035150 | regulation of tube size | 19/1200 | 142/18723 | 0.001821 | 0.028452 | 0.023646 | CD38/NPR3/P2RY1/SLC6A4/ADRB2/P2RX1/F2RL1/KCNA5/GJA1/GCLC/HTR2B/ACE2/UTS2/KNG1/HTR7/AGTR1/ADRA2A/EDN2/ADRB1 | 19 |
| BP | GO:0046718 | viral entry into host cell | 19/1200 | 144/18723 | 0.002144 | 0.031551 | 0.026222 | CR2/CD86/SLAMF1/CXCR4/CXADR/FCN1/CR1/MID2/CTSL/CD80/CLEC4M/HSPA1B/FCN3/HSPA1A/ACE2/CD46/AGTR1/SIGLEC1/CLDN9 | 19 |
| BP | GO:0044409 | entry into host | 19/1200 | 151/18723 | 0.003679 | 0.046871 | 0.038954 | CR2/CD86/SLAMF1/CXCR4/CXADR/FCN1/CR1/MID2/CTSL/CD80/CLEC4M/HSPA1B/FCN3/HSPA1A/ACE2/CD46/AGTR1/SIGLEC1/CLDN9 | 19 |
| BP | GO:0006665 | sphingolipid metabolic process | 19/1200 | 155/18723 | 0.004907 | 0.054989 | 0.045701 | ST8SIA4/SPTSSB/ENPP2/FUT7/ST3GAL1/B4GALT6/DEGS1/NEU4/ELOVL4/P2RX1/ST6GALNAC4/ACER1/ST3GAL2/PSAPL1/SGPP2/ELOVL2/ALDH3B2/ELOVL6/PNPLA1 | 19 |
| CC | GO:0043296 | apical junction complex | 19/1263 | 145/19550 | 0.002543 | 0.052934 | 0.047631 | AMOTL1/CLDN10/WNK3/JUP/POF1B/CXADR/PARD3/MAGI1/CLDN3/NHS/MARVELD2/OCLN/FZD5/PARD6A/CDH1/EPCAM/DLG1/SHROOM1/CLDN9 | 19 |
| MF | GO:0004222 | metalloendopeptidase activity | 19/1229 | 111/18368 | 0.000134 | 0.007245 | 0.006538 | TLL2/MMP3/MMP10/MMP7/ADAMTS9/ADAM28/MMP1/MMP9/ADAMDEC1/ADAMTS6/ADAMTS3/MMP27/ADAM23/ADAM8/KLK7/MMP12/MMP13/CLCA2/ADAMTS20 | 19 |
| BP | GO:0045621 | positive regulation of lymphocyte differentiation | 18/1200 | 104/18723 | 0.000101 | 0.003427 | 0.002848 | LILRB2/XBP1/CD86/RHOH/PTPRC/BTK/PCK1/NCKAP1L/CR1/IL7R/CD80/EGR3/VNN1/IL2RA/ADAM8/CD46/ADA/TOX | 18 |
| BP | GO:0071887 | leukocyte apoptotic process | 18/1200 | 106/18723 | 0.00013 | 0.004176 | 0.00347 | HCLS1/CD3G/ST3GAL1/LYN/PRKCQ/BTK/LILRB1/IL7R/CTSL/CCL5/IL2RA/ADAM8/BMP4/IDO1/PIK3CD/MERTK/SLC7A11/ADA | 18 |
| BP | GO:0046632 | alpha-beta T cell differentiation | 18/1200 | 112/18723 | 0.000265 | 0.006999 | 0.005817 | IRF4/CD86/BATF/IL21/FUT7/GPR183/PSMB11/GPR18/ITK/NCKAP1L/CTSL/CD80/PLA2G2D/RORC/EOMES/ADA/TNFSF8/TOX | 18 |
| BP | GO:0035967 | cellular response to topologically incorrect protein | 18/1200 | 116/18723 | 0.000412 | 0.009475 | 0.007874 | XBP1/ERLEC1/HSPA2/ERN1/HSPA13/RHBDD1/SDF2L1/CTH/DNAJB9/HERPUD1/UGGT1/PIK3R1/HSPA1B/CREB3L2/AGR2/BHLHA15/HSPA1A/HSPA1L | 18 |
| BP | GO:0030282 | bone mineralization | 18/1200 | 119/18723 | 0.000565 | 0.01205 | 0.010015 | SRGN/TUFT1/GPNMB/ADRB2/PTK2B/LGR4/MGP/P2RX7/FGR/TGFB3/FGFR2/LTF/PTN/BMP4/STATH/WNT11/MMP13/ECM1 | 18 |
| BP | GO:0002761 | regulation of myeloid leukocyte differentiation | 18/1200 | 120/18723 | 0.000625 | 0.012775 | 0.010617 | HCLS1/RASSF2/CTNNBIP1/MYC/LILRB3/TREM2/LYN/OCSTAMP/LILRB1/PIK3R1/FBN1/IL17A/TESC/TYROBP/LTF/MITF/TNFSF11/PLA2G3 | 18 |
| BP | GO:0019730 | antimicrobial humoral response | 18/1200 | 122/18723 | 0.000764 | 0.014967 | 0.012439 | RNASE6/JCHAIN/CXCL1/CXCL3/IL17F/CXCL2/IL17A/CXCL6/LYZ/LTF/CXCL13/KLK7/DEFB1/PGC/CCL13/PF4V1/GNLY/F2 | 18 |
| BP | GO:0045089 | positive regulation of innate immune response | 18/1200 | 131/18723 | 0.001757 | 0.02769 | 0.023013 | CD226/PLCG2/VAV1/NLRC4/ZBP1/IL21/LYN/AIM2/FFAR2/FCN1/PAK3/CCL5/TYROBP/MNDA/CLEC6A/ADAM8/MMP12/LILRA2 | 18 |
| BP | GO:1903038 | negative regulation of leukocyte cell-cell adhesion | 18/1200 | 141/18723 | 0.003962 | 0.048691 | 0.040466 | LILRB2/CD86/WNK1/GPNMB/CTLA4/LILRB1/NCKAP1L/LAX1/ADTRP/CR1/CD80/PLA2G2D/IL2RA/BMP4/MIA3/DLG1/NRARP/KLF4 | 18 |
| BP | GO:0072006 | nephron development | 18/1200 | 142/18723 | 0.004273 | 0.049942 | 0.041506 | COL4A4/SULF2/JAG1/CTNNBIP1/MYC/NID1/PECAM1/HES5/PODXL/LGR4/COL4A3/WNT2B/BMP4/IRX2/DLG1/SERPINB7/RET/WNT11 | 18 |
| BP | GO:0070098 | chemokine-mediated signaling pathway | 17/1200 | 88/18723 | 3.72E-05 | 0.001702 | 0.001415 | WNK1/STK39/TREM2/CXCR4/PTK2B/CXCL1/MPL/CXCL3/CXCL2/CCL5/CXCL6/CCL18/CXCL13/CCR3/CCL13/CXCR1/PF4V1 | 17 |
| BP | GO:1990868 | response to chemokine | 17/1200 | 97/18723 | 0.000133 | 0.004197 | 0.003488 | WNK1/STK39/TREM2/CXCR4/PTK2B/CXCL1/MPL/CXCL3/CXCL2/CCL5/CXCL6/CCL18/CXCL13/CCR3/CCL13/CXCR1/PF4V1 | 17 |
| BP | GO:1990869 | cellular response to chemokine | 17/1200 | 97/18723 | 0.000133 | 0.004197 | 0.003488 | WNK1/STK39/TREM2/CXCR4/PTK2B/CXCL1/MPL/CXCL3/CXCL2/CCL5/CXCL6/CCL18/CXCL13/CCR3/CCL13/CXCR1/PF4V1 | 17 |
| BP | GO:0002444 | myeloid leukocyte mediated immunity | 17/1200 | 99/18723 | 0.000172 | 0.005156 | 0.004285 | LAT2/STAP1/LYN/BTK/FGR/F2RL1/TREM1/PIK3CG/C3/CXCL6/TYROBP/MILR1/NLRP6/PIK3CD/PLA2G3/IL13RA2/F2 | 17 |
| BP | GO:0045639 | positive regulation of myeloid cell differentiation | 17/1200 | 103/18723 | 0.000281 | 0.007277 | 0.006048 | HCLS1/JAG1/CTNNBIP1/SCIN/TREM2/OCSTAMP/MPL/NCKAP1L/PITHD1/HSPA1B/IL17A/TESC/TYROBP/HSPA1A/ACVR1B/TNFSF11/PLA2G3 | 17 |
| BP | GO:0062207 | regulation of pattern recognition receptor signaling pathway | 17/1200 | 105/18723 | 0.000355 | 0.008557 | 0.007111 | IRF4/MFHAS1/CYBA/TREM2/LYN/FCRL3/LGR4/TLR2/BIRC3/TLR1/F2RL1/HSPA1B/ARRB2/LTF/HSPA1A/NLRP6/LILRA2 | 17 |
| BP | GO:1903557 | positive regulation of tumor necrosis factor superfamily cytokine production | 17/1200 | 107/18723 | 0.000445 | 0.009985 | 0.008298 | LY96/PLCG2/CD86/CYBA/CYBB/PTPRC/TLR2/CD14/IL17F/TLR1/PIK3R1/LILRA5/IL17A/TYROBP/FZD5/ADAM8/LILRA2 | 17 |
| BP | GO:0043406 | positive regulation of MAP kinase activity | 17/1200 | 112/18723 | 0.000763 | 0.014967 | 0.012439 | MST1R/ERN1/FLT3/GHR/PTK2B/TIAM1/IL1B/EGF/PIK3CG/FZD5/ADAM8/NTF3/HTR2B/AJUBA/TNFSF11/ADRA2A/FZD10 | 17 |
| BP | GO:0002688 | regulation of leukocyte chemotaxis | 17/1200 | 122/18723 | 0.001997 | 0.030054 | 0.024978 | STAP1/WNK1/SLAMF1/STK39/AIF1/LYN/PTK2B/GPR18/F2RL1/NCKAP1L/CCL5/PLA2G7/PTN/C3AR1/CXCL13/S100A14/EDN2 | 17 |
| BP | GO:0006690 | icosanoid metabolic process | 17/1200 | 123/18723 | 0.002182 | 0.031938 | 0.026543 | CES2/ALOX5AP/DPEP1/TLR2/AKR1B1/IL1B/CYP4F8/ABHD6/ALOX15B/CYP2C18/CYP2J2/CYP4F12/GSTA1/CYP4F11/PLA2G3/PLA2G10/EDN2 | 17 |
| BP | GO:0050852 | T cell receptor signaling pathway | 17/1200 | 123/18723 | 0.002182 | 0.031938 | 0.026543 | CD226/PLCG2/WNK1/SKAP1/CTLA4/CD247/PTPRC/KCNN4/TRAT1/THEMIS2/ITK/LCK/BTN3A1/PIK3CD/ADA/PDE4B/BTN1A1 | 17 |
| BP | GO:0070304 | positive regulation of stress-activated protein kinase signaling cascade | 17/1200 | 128/18723 | 0.003338 | 0.044429 | 0.036925 | RASSF2/IL26/SLAMF1/STK39/MFHAS1/LYN/TNFRSF19/PTK2B/IL1B/SCIMP/EDA2R/F2RL1/FGF19/BMP4/GDF6/TNFSF11/WNT16 | 17 |
| BP | GO:0007498 | mesoderm development | 17/1200 | 129/18723 | 0.003621 | 0.046351 | 0.038522 | IKZF3/MESP1/RPS6KA6/TBX1/BTK/ITGA8/TP63/WNT3/GJA1/TCF15/TXNRD1/FGFR2/BMP4/EOMES/ETS2/WNT11/KLF4 | 17 |
| BP | GO:0006956 | complement activation | 17/1200 | 130/18723 | 0.003924 | 0.048473 | 0.040286 | CR2/KRT1/CR1L/SUSD4/TREM2/IGLL1/IL1B/FCN2/CFH/FCN1/CR1/C3/FCN3/CD46/C1QA/MBL2/C1QB | 17 |
| BP | GO:0002705 | positive regulation of leukocyte mediated immunity | 17/1200 | 134/18723 | 0.00535 | 0.057182 | 0.047523 | CD226/PLCG2/VAV1/STAP1/SLAMF1/IL21/TREM2/PTPRC/BTK/IL1B/SCIMP/F2RL1/C3/TYROBP/FZD5/AZGP1/CD1E | 17 |
| BP | GO:0061041 | regulation of wound healing | 17/1200 | 134/18723 | 0.00535 | 0.057182 | 0.047523 | XBP1/KRT1/CXCR4/F12/GP1BA/CLDN3/KANK1/F2RL1/ENPP4/GJA1/ADTRP/OCLN/FERMT1/KNG1/TSPAN8/ADRA2A/F2 | 17 |
| CC | GO:1904813 | ficolin-1-rich granule lumen | 17/1263 | 124/19550 | 0.002591 | 0.052934 | 0.047631 | CTSS/GMFG/TNFAIP6/KRT1/PRDX4/BIN2/JUP/CALML5/HK3/MMP9/FCN1/GNS/HSPA1B/MNDA/PGM2/HSPA1A/YPEL5 | 17 |
| BP | GO:0030888 | regulation of B cell proliferation | 16/1200 | 64/18723 | 1.92E-06 | 0.000176 | 0.000146 | MZB1/CD38/IKZF3/IL21/CTLA4/LYN/FCRL3/PTPRC/GPR183/BTK/VAV3/NCKAP1L/TYROBP/MNDA/NFATC2/ADA | 16 |
| BP | GO:0033627 | cell adhesion mediated by integrin | 16/1200 | 72/18723 | 9.94E-06 | 0.000614 | 0.00051 | WNK1/ITGA4/PDE3B/SKAP1/LYN/PODXL/LPXN/FBN1/NCKAP1L/CCL5/PIK3CG/TESC/CXCL13/FERMT1/RET/ADA | 16 |
| BP | GO:0061844 | antimicrobial humoral immune response mediated by antimicrobial peptide | 16/1200 | 79/18723 | 3.41E-05 | 0.001595 | 0.001326 | RNASE6/CXCL1/CXCL3/IL17F/CXCL2/IL17A/CXCL6/LTF/CXCL13/KLK7/DEFB1/PGC/CCL13/PF4V1/GNLY/F2 | 16 |
| BP | GO:2000106 | regulation of leukocyte apoptotic process | 16/1200 | 81/18723 | 4.70E-05 | 0.002047 | 0.001701 | HCLS1/CD3G/ST3GAL1/LYN/PRKCQ/BTK/LILRB1/IL7R/CCL5/ADAM8/BMP4/IDO1/PIK3CD/MERTK/SLC7A11/ADA | 16 |
| BP | GO:0050672 | negative regulation of lymphocyte proliferation | 16/1200 | 83/18723 | 6.41E-05 | 0.002561 | 0.002128 | LILRB2/CD86/GPNMB/CTLA4/LYN/BTK/LILRB1/CR1/CD80/TYROBP/MNDA/PLA2G2D/IL2RA/BMP4/DLG1/SOX11 | 16 |
| BP | GO:0032945 | negative regulation of mononuclear cell proliferation | 16/1200 | 84/18723 | 7.46E-05 | 0.002849 | 0.002368 | LILRB2/CD86/GPNMB/CTLA4/LYN/BTK/LILRB1/CR1/CD80/TYROBP/MNDA/PLA2G2D/IL2RA/BMP4/DLG1/SOX11 | 16 |
| BP | GO:0001776 | leukocyte homeostasis | 16/1200 | 87/18723 | 0.000115 | 0.003782 | 0.003143 | TNFRSF17/FLT3/LYN/GPR183/GPR174/MPL/TCIRG1/NCKAP1L/CXCL6/GAPT/IL2RA/PIK3CD/MERTK/SLC7A11/ADA/PDE4B | 16 |
| BP | GO:0070664 | negative regulation of leukocyte proliferation | 16/1200 | 90/18723 | 0.000174 | 0.005205 | 0.004326 | LILRB2/CD86/GPNMB/CTLA4/LYN/BTK/LILRB1/CR1/CD80/TYROBP/MNDA/PLA2G2D/IL2RA/BMP4/DLG1/SOX11 | 16 |
| BP | GO:0045582 | positive regulation of T cell differentiation | 16/1200 | 91/18723 | 0.000199 | 0.005752 | 0.004781 | LILRB2/XBP1/CD86/RHOH/PTPRC/PCK1/NCKAP1L/CR1/IL7R/CD80/EGR3/VNN1/IL2RA/ADAM8/CD46/ADA | 16 |
| BP | GO:0032755 | positive regulation of interleukin-6 production | 16/1200 | 93/18723 | 0.000258 | 0.006858 | 0.0057 | LILRB2/POU2AF1/XBP1/PLCG2/CYBA/AIF1/TLR2/IL1B/SCIMP/IL17F/TLR1/LILRA5/F2RL1/IL17A/TYROBP/LILRA2 | 16 |
| BP | GO:0034620 | cellular response to unfolded protein | 16/1200 | 96/18723 | 0.000375 | 0.008849 | 0.007354 | XBP1/ERLEC1/HSPA2/ERN1/HSPA13/RHBDD1/CTH/DNAJB9/HERPUD1/PIK3R1/HSPA1B/CREB3L2/AGR2/BHLHA15/HSPA1A/HSPA1L | 16 |
| BP | GO:0120162 | positive regulation of cold-induced thermogenesis | 16/1200 | 97/18723 | 0.000423 | 0.009677 | 0.008042 | IRF4/CXCR4/UCP1/ADRB2/GJA1/FABP4/GATM/UCP2/SCD/PLAC8/ELOVL6/G0S2/TSHR/ESRRG/ADRB1/SLN | 16 |
| BP | GO:0042102 | positive regulation of T cell proliferation | 16/1200 | 101/18723 | 0.00067 | 0.013242 | 0.011005 | LILRB2/CD86/SLAMF1/RASAL3/AIF1/IL21/CLECL1/PRKCQ/PTPRC/IL1B/NCKAP1L/CD80/CCL5/IL2RA/CD40LG/CD46 | 16 |
| BP | GO:0007200 | phospholipase C-activating G protein-coupled receptor signaling pathway | 16/1200 | 104/18723 | 0.000929 | 0.017006 | 0.014133 | NPR3/P2RY1/P2RY10/GPR65/GPR174/GPR18/F2RL1/LPAR6/C3AR1/HTR2B/FPR1/AGTR1/ADRA2A/NMUR1/TGM2/F2 | 16 |
| BP | GO:0006720 | isoprenoid metabolic process | 16/1200 | 116/18723 | 0.002963 | 0.039847 | 0.033117 | ALDH3A2/BCO1/CRABP2/AKR1B1/CYP2W1/PMVK/ALDH1A1/RDH12/CYP2C18/CYP3A7/CYP3A5/ADH7/STAR/AKR1B10/PNLIP/HMGCS1 | 16 |
| BP | GO:0001704 | formation of primary germ layer | 16/1200 | 121/18723 | 0.004526 | 0.051638 | 0.042916 | MESP1/ITGA4/SOX2/ITGA8/MMP9/WNT3/COL4A2/GJA1/TXNRD1/FGFR2/DUSP4/BMP4/EOMES/ETS2/WNT11/KLF4 | 16 |
| BP | GO:0002920 | regulation of humoral immune response | 15/1200 | 45/18723 | 6.44E-08 | 1.07E-05 | 8.89E-06 | CR2/CR1L/SUSD4/TREM2/PTPRC/IL1B/IL17F/CFH/CR1/IL17A/C3/CXCL13/CD46/KLK7/PGC | 15 |
| BP | GO:0034113 | heterotypic cell-cell adhesion | 15/1200 | 61/18723 | 4.99E-06 | 0.000368 | 0.000306 | DSP/LILRB2/WNK1/ITGA4/DSC2/SKAP1/CD200/ITGAX/PERP/JUP/PTPRC/IL1B/CXADR/LCK/KLF4 | 15 |
| BP | GO:0034121 | regulation of toll-like receptor signaling pathway | 15/1200 | 75/18723 | 6.93E-05 | 0.002745 | 0.002282 | IRF4/MFHAS1/CYBA/TREM2/LYN/FCRL3/LGR4/TLR2/BIRC3/TLR1/F2RL1/ARRB2/LTF/NLRP6/LILRA2 | 15 |
| BP | GO:0120193 | tight junction organization | 15/1200 | 80/18723 | 0.000149 | 0.004634 | 0.003851 | CLDN10/PECAM1/POF1B/PARD3/TGFB3/CLDN3/GJA1/IL17A/GRHL2/MARVELD2/OCLN/FZD5/DLG1/WNT11/CLDN9 | 15 |
| BP | GO:0002312 | B cell activation involved in immune response | 15/1200 | 82/18723 | 0.000199 | 0.005752 | 0.004781 | CD19/POU2AF1/XBP1/PLCG2/CD180/BATF/IL21/ST3GAL1/PTPRC/PTK2B/GPR183/CR1/GAPT/CD40LG/ADA | 15 |
| BP | GO:0050829 | defense response to Gram-negative bacterium | 15/1200 | 88/18723 | 0.000441 | 0.009929 | 0.008252 | RNASE6/TREM2/SELP/FCN2/IL17F/F2RL1/TREM1/IL17A/CXCL6/LYZ/LTF/DEFB1/PRB3/LCE3B/F2 | 15 |
| BP | GO:0046849 | bone remodeling | 15/1200 | 90/18723 | 0.000565 | 0.01205 | 0.010015 | CD38/RASSF2/ADRB2/PTK2B/LGR4/TCIRG1/P2RX7/MC4R/TGFB3/GJA1/PTN/ADAM8/MITF/TNFSF11/WNT16 | 15 |
| BP | GO:0002275 | myeloid cell activation involved in immune response | 15/1200 | 91/18723 | 0.000637 | 0.012912 | 0.010731 | LAT2/PLCG2/SLAMF1/TREM2/LYN/BTK/FGR/F2RL1/PIK3CG/TYROBP/MILR1/PIK3CD/PLA2G3/IL13RA2/LILRA2 | 15 |
| BP | GO:0050764 | regulation of phagocytosis | 15/1200 | 95/18723 | 0.001009 | 0.018293 | 0.015203 | PLCG2/STAP1/CYBA/TREM2/PTPRC/TLR2/IL1B/FGR/F2RL1/NCKAP1L/SPACA3/C3/IL2RG/MERTK/TGM2 | 15 |
| BP | GO:0006721 | terpenoid metabolic process | 15/1200 | 97/18723 | 0.001255 | 0.021234 | 0.017647 | ALDH3A2/BCO1/CRABP2/AKR1B1/CYP2W1/ALDH1A1/RDH12/CYP2C18/CYP3A7/CYP3A5/ADH7/STAR/AKR1B10/PNLIP/HMGCS1 | 15 |
| BP | GO:0050830 | defense response to Gram-positive bacterium | 15/1200 | 101/18723 | 0.001901 | 0.029264 | 0.024321 | RNASE6/TLR2/FCN2/IL17F/FGR/GBP6/C10orf99/IL7R/IL17A/LYZ/NLRP6/DEFB1/MBL2/TNFSF8/LCE3B | 15 |
| BP | GO:0034308 | primary alcohol metabolic process | 15/1200 | 102/18723 | 0.002099 | 0.030976 | 0.025743 | ALDH3A2/BCO1/TPK1/AKR1B1/ALDH1A1/RDH12/CYP2C18/CYP3A7/CYP3A5/ADH7/AKR1B10/ALDH3B2/SULT2A1/PNLIP/BMP5 | 15 |
| BP | GO:0030148 | sphingolipid biosynthetic process | 15/1200 | 103/18723 | 0.002315 | 0.033477 | 0.027822 | ST8SIA4/SPTSSB/ST3GAL1/B4GALT6/DEGS1/ELOVL4/P2RX1/ST6GALNAC4/ACER1/ST3GAL2/SGPP2/ELOVL2/ALDH3B2/ELOVL6/PNPLA1 | 15 |
| BP | GO:0032526 | response to retinoic acid | 15/1200 | 107/18723 | 0.003369 | 0.044631 | 0.037092 | CD38/SLC6A4/TBX1/LYN/PTK2B/AQP3/PCK1/WNT3/GJA1/TESC/FGFR2/RET/WNT11/KLF4/FZD10 | 15 |
| BP | GO:0003014 | renal system process | 15/1200 | 110/18723 | 0.004395 | 0.050975 | 0.042365 | NPR3/CYBA/SULF2/JCHAIN/RHPN2/AKR1B1/AQP3/F2RL1/GJA1/CHRNA3/BMP4/CYP4F12/HAS2/AGTR1/MLLT6 | 15 |
| BP | GO:0006939 | smooth muscle contraction | 15/1200 | 110/18723 | 0.004395 | 0.050975 | 0.042365 | CD38/SULF2/NPY2R/ADRB2/P2RX1/CHRNA3/PROK2/HTR2B/DLG1/HTR7/ADA/ADRA2A/EDN2/NMUR1/NEUROG1 | 15 |
| BP | GO:0001938 | positive regulation of endothelial cell proliferation | 15/1200 | 111/18723 | 0.004789 | 0.053779 | 0.044695 | MYDGF/CYBA/ITGA4/EGF/EGR3/FGFBP1/BMP4/HTR2B/SCG2/PIK3CD/CCR3/NRARP/AGTR1/ECM1/STAT5A | 15 |
| BP | GO:0001676 | long-chain fatty acid metabolic process | 15/1200 | 112/18723 | 0.005211 | 0.056257 | 0.046754 | ACOT2/SLC27A6/ADTRP/ACSM1/ABHD6/ALOX15B/CYP2C18/CYP2J2/SLC27A2/CYP4F12/ELOVL2/GSTA1/CYP4F11/PLA2G10/ELOVL6 | 15 |
| BP | GO:0032609 | interferon-gamma production | 15/1200 | 112/18723 | 0.005211 | 0.056257 | 0.046754 | CD226/SLAMF1/IL21/IL1B/CD14/LILRB1/F2RL1/CR1/HMHB1/BTN3A1/FZD5/NLRP6/IL12RB2/SLC7A5/PDE4B | 15 |
| BP | GO:0032649 | regulation of interferon-gamma production | 15/1200 | 112/18723 | 0.005211 | 0.056257 | 0.046754 | CD226/SLAMF1/IL21/IL1B/CD14/LILRB1/F2RL1/CR1/HMHB1/BTN3A1/FZD5/NLRP6/IL12RB2/SLC7A5/PDE4B | 15 |
| BP | GO:0048640 | negative regulation of developmental growth | 15/1200 | 112/18723 | 0.005211 | 0.056257 | 0.046754 | TLL2/MAP2/MEIS1/SLC6A4/ADRB2/FSTL4/WNT3/GJA1/ARHGAP4/SEMA7A/BMP4/RGS4/PLAC8/ADRB1/CGA | 15 |
| CC | GO:0005604 | basement membrane | 15/1263 | 96/19550 | 0.001222 | 0.031831 | 0.028642 | LAMB4/COL4A4/NID1/COL4A3/COL4A2/FBN1/COL4A1/LAMC3/PTN/THBS2/COL15A1/DLG1/COL4A6/TIMP1/LAMC2 | 15 |
| BP | GO:0033628 | regulation of cell adhesion mediated by integrin | 14/1200 | 48/18723 | 1.12E-06 | 0.000115 | 9.55E-05 | WNK1/PDE3B/SKAP1/LYN/PODXL/LPXN/NCKAP1L/CCL5/PIK3CG/TESC/CXCL13/FERMT1/RET/ADA | 14 |
| BP | GO:0006968 | cellular defense response | 14/1200 | 54/18723 | 5.27E-06 | 0.000378 | 0.000314 | LILRB2/LY96/PTK2B/PRF1/FCMR/TCIRG1/TRAT1/ITK/TYROBP/NCF2/MNDA/CCR3/GNLY/DCDC2 | 14 |
| BP | GO:0072678 | T cell migration | 14/1200 | 66/18723 | 6.10E-05 | 0.002475 | 0.002057 | MYO1G/WNK1/STK39/ITGA4/AIF1/CD200/GPR183/C10orf99/CCL5/PIK3CG/ADAM8/CXCL13/PIK3CD/ECM1 | 14 |
| BP | GO:0031640 | killing of cells of other organism | 14/1200 | 68/18723 | 8.62E-05 | 0.003048 | 0.002533 | PRF1/P2RX7/F2RL1/TREM1/CXCL6/LYZ/DCD/LTF/NLRP6/BCL2L1/CCL13/GNLY/LCE3B/F2 | 14 |
| BP | GO:0070830 | bicellular tight junction assembly | 14/1200 | 70/18723 | 0.00012 | 0.003906 | 0.003246 | CLDN10/PECAM1/POF1B/PARD3/CLDN3/GJA1/IL17A/GRHL2/MARVELD2/OCLN/FZD5/DLG1/WNT11/CLDN9 | 14 |
| BP | GO:0120192 | tight junction assembly | 14/1200 | 74/18723 | 0.000223 | 0.006156 | 0.005117 | CLDN10/PECAM1/POF1B/PARD3/CLDN3/GJA1/IL17A/GRHL2/MARVELD2/OCLN/FZD5/DLG1/WNT11/CLDN9 | 14 |
| BP | GO:0043297 | apical junction assembly | 14/1200 | 78/18723 | 0.000393 | 0.009162 | 0.007615 | CLDN10/PECAM1/POF1B/PARD3/CLDN3/GJA1/IL17A/GRHL2/MARVELD2/OCLN/FZD5/DLG1/WNT11/CLDN9 | 14 |
| BP | GO:0001910 | regulation of leukocyte mediated cytotoxicity | 14/1200 | 82/18723 | 0.000665 | 0.013242 | 0.011005 | CD226/VAV1/STAP1/IL21/PTPRC/LILRB1/F2RL1/NCKAP1L/IL7R/CXCL6/ARRB2/TYROBP/AZGP1/CD1E | 14 |
| BP | GO:0016101 | diterpenoid metabolic process | 14/1200 | 87/18723 | 0.001215 | 0.02068 | 0.017187 | ALDH3A2/BCO1/CRABP2/AKR1B1/CYP2W1/ALDH1A1/RDH12/CYP2C18/CYP3A7/CYP3A5/ADH7/STAR/AKR1B10/PNLIP | 14 |
| BP | GO:0034109 | homotypic cell-cell adhesion | 14/1200 | 90/18723 | 0.001698 | 0.026925 | 0.022377 | DSP/DSC2/PLEK/LYN/JUP/PRKCQ/MPL/CXADR/GP1BA/CCL5/PIK3CG/SLC7A11/TNFSF11/MEGF10 | 14 |
| BP | GO:0002690 | positive regulation of leukocyte chemotaxis | 14/1200 | 94/18723 | 0.002582 | 0.035808 | 0.029759 | WNK1/SLAMF1/STK39/AIF1/PTK2B/F2RL1/NCKAP1L/CCL5/PLA2G7/PTN/C3AR1/CXCL13/S100A14/EDN2 | 14 |
| BP | GO:0030316 | osteoclast differentiation | 14/1200 | 94/18723 | 0.002582 | 0.035808 | 0.029759 | RASSF2/LILRB3/TREM2/GPR183/OCSTAMP/LILRB1/TCIRG1/PIK3R1/FBN1/IL17A/TYROBP/LTF/MITF/TNFSF11 | 14 |
| BP | GO:0002367 | cytokine production involved in immune response | 14/1200 | 98/18723 | 0.003813 | 0.047655 | 0.039605 | CD226/PLCG2/SLAMF1/BTK/IL1B/SCIMP/LILRB1/FFAR2/TGFB3/F2RL1/TREM1/SEMA7A/FZD5/SLC7A5 | 14 |
| BP | GO:0006664 | glycolipid metabolic process | 14/1200 | 100/18723 | 0.004588 | 0.05196 | 0.043183 | ST8SIA4/PIGK/PGAP1/ST3GAL1/B4GALT6/NEU4/ST6GALNAC4/GBGT1/PGAP2/ST3GAL2/CWH43/FUCA1/B3GNT5/PIGA | 14 |
| BP | GO:1903509 | liposaccharide metabolic process | 14/1200 | 101/18723 | 0.00502 | 0.056103 | 0.046627 | ST8SIA4/PIGK/PGAP1/ST3GAL1/B4GALT6/NEU4/ST6GALNAC4/GBGT1/PGAP2/ST3GAL2/CWH43/FUCA1/B3GNT5/PIGA | 14 |
| BP | GO:0035710 | CD4-positive, alpha-beta T cell activation | 14/1200 | 102/18723 | 0.005485 | 0.057578 | 0.047852 | IRF4/CD86/BATF/IL21/FUT7/PRKCQ/GPR183/TCIRG1/NCKAP1L/CTSL/CD80/PLA2G2D/RORC/TOX | 14 |
| CC | GO:0001533 | cornified envelope | 14/1263 | 45/19550 | 5.15E-07 | 0.000123 | 0.00011 | DSP/KRT1/DSC2/PKP3/DSC3/JUP/PKP1/KRT2/DSG3/RPTN/KRT10/PPL/SPRR1A/TCHH | 14 |
| CC | GO:0101003 | ficolin-1-rich granule membrane | 14/1263 | 61/19550 | 2.62E-05 | 0.001331 | 0.001197 | DSP/LILRB2/SIGLEC5/ARL8A/CLEC4D/ITGAX/PKP1/TCIRG1/ENPP4/NCKAP1L/CR1/ADAM8/FPR1/MGAM | 14 |
| BP | GO:0045104 | intermediate filament cytoskeleton organization | 13/1200 | 51/18723 | 1.39E-05 | 0.000839 | 0.000697 | DSP/KRT3/KRT16/BFSP2/KRT18/PKP1/KRT2/NEFM/DNAJB6/NEFL/PPL/KRT25/TCHH | 13 |
| BP | GO:0043277 | apoptotic cell clearance | 13/1200 | 52/18723 | 1.75E-05 | 0.000977 | 0.000812 | TXNDC5/RHOH/TREM2/FCN2/FCN1/C3/FCN3/TYROBP/MERTK/MARCO/MEGF10/TGM2/XKR9 | 13 |
| BP | GO:0045103 | intermediate filament-based process | 13/1200 | 52/18723 | 1.75E-05 | 0.000977 | 0.000812 | DSP/KRT3/KRT16/BFSP2/KRT18/PKP1/KRT2/NEFM/DNAJB6/NEFL/PPL/KRT25/TCHH | 13 |
| BP | GO:0031663 | lipopolysaccharide-mediated signaling pathway | 13/1200 | 60/18723 | 8.81E-05 | 0.003059 | 0.002542 | LY96/PLCG2/CD180/LYN/TLR2/IL1B/CD14/SCIMP/LY86/CCL5/LTF/IRAK2/LILRA2 | 13 |
| BP | GO:2000401 | regulation of lymphocyte migration | 13/1200 | 61/18723 | 0.000105 | 0.003502 | 0.00291 | WNK1/STK39/ITGA4/AIF1/CD200/PTK2B/C10orf99/ADTRP/CCL5/ADAM8/CXCL13/MIA3/ECM1 | 13 |
| BP | GO:0048247 | lymphocyte chemotaxis | 13/1200 | 64/18723 | 0.000176 | 0.005235 | 0.004351 | WNK1/STK39/PTK2B/GPR183/C10orf99/CCL5/PIK3CG/SAA1/CCL18/ADAM8/CXCL13/PIK3CD/CCL13 | 13 |
| BP | GO:0032729 | positive regulation of interferon-gamma production | 13/1200 | 72/18723 | 0.000591 | 0.012225 | 0.01016 | CD226/SLAMF1/IL21/IL1B/CD14/LILRB1/F2RL1/HMHB1/BTN3A1/FZD5/IL12RB2/SLC7A5/PDE4B | 13 |
| BP | GO:0033077 | T cell differentiation in thymus | 13/1200 | 75/18723 | 0.000884 | 0.016595 | 0.013792 | CD3D/CD3G/PTPRC/IL1B/PSMB11/IL7R/EGR3/FZD5/VNN1/ADAM8/BMP4/ADA/TOX | 13 |
| BP | GO:0014068 | positive regulation of phosphatidylinositol 3-kinase signaling | 13/1200 | 79/18723 | 0.001453 | 0.023828 | 0.019803 | HCLS1/MYDGF/FLT3/TREM2/SELP/FGR/PLXNB1/F2RL1/EGF/CCL5/WNT16/F2/NTRK2 | 13 |
| BP | GO:0048678 | response to axon injury | 13/1200 | 83/18723 | 0.002299 | 0.033367 | 0.027731 | PLCG2/CHL1/AIF1/TREM2/LYN/P2RX4/PTPRF/KREMEN1/TYROBP/NEFL/PTN/FLRT3/KLF4 | 13 |
| CC | GO:0070821 | tertiary granule membrane | 13/1263 | 73/19550 | 0.000731 | 0.021475 | 0.019324 | LILRB2/CD53/SIGLEC5/CYBA/CLEC4D/ITGAX/PLD1/CYBB/LAIR1/CLEC12A/ADAM8/SVIP/MGAM | 13 |
| MF | GO:0001618 | virus receptor activity | 13/1229 | 76/18368 | 0.001485 | 0.042594 | 0.038437 | CR2/CD86/SLAMF1/CXCR4/CXADR/CR1/CD80/CLEC4M/HSPA1B/HSPA1A/ACE2/CD46/CLDN9 | 13 |
| MF | GO:0140272 | exogenous protein binding | 13/1229 | 77/18368 | 0.001679 | 0.042817 | 0.038639 | CR2/CD86/SLAMF1/CXCR4/CXADR/CR1/CD80/CLEC4M/HSPA1B/HSPA1A/ACE2/CD46/CLDN9 | 13 |
| BP | GO:0034142 | toll-like receptor 4 signaling pathway | 12/1200 | 43/18723 | 1.09E-05 | 0.000664 | 0.000552 | LY96/MFHAS1/TREM2/LYN/CD14/SCIMP/F2RL1/RAB11FIP2/LTF/S100A14/TNIP3/LILRA2 | 12 |
| BP | GO:0002218 | activation of innate immune response | 12/1200 | 52/18723 | 8.56E-05 | 0.003048 | 0.002533 | PLCG2/NLRC4/ZBP1/LYN/AIM2/FFAR2/FCN1/PAK3/TYROBP/MNDA/CLEC6A/LILRA2 | 12 |
| BP | GO:0009620 | response to fungus | 12/1200 | 60/18723 | 0.000361 | 0.008598 | 0.007146 | PLCG2/CLEC4D/BTK/SCIMP/C10orf99/IL17A/CLEC4E/MPO/DCD/LTF/CLEC6A/GNLY | 12 |
| BP | GO:0002704 | negative regulation of leukocyte mediated immunity | 12/1200 | 63/18723 | 0.000576 | 0.01205 | 0.010015 | CR2/SLAMF1/CR1L/SUSD4/PTPRC/LILRB1/NCKAP1L/CR1/IL7R/ARRB2/CD46/IL13RA2 | 12 |
| BP | GO:0050854 | regulation of antigen receptor-mediated signaling pathway | 12/1200 | 63/18723 | 0.000576 | 0.01205 | 0.010015 | CD19/CD226/STAP1/LYN/FCRL3/PTPRC/PRKCB/KCNN4/TRAT1/LPXN/LCK/ADA | 12 |
| BP | GO:0045670 | regulation of osteoclast differentiation | 12/1200 | 64/18723 | 0.000669 | 0.013242 | 0.011005 | RASSF2/LILRB3/TREM2/OCSTAMP/LILRB1/PIK3R1/FBN1/IL17A/TYROBP/LTF/MITF/TNFSF11 | 12 |
| BP | GO:0050766 | positive regulation of phagocytosis | 12/1200 | 66/18723 | 0.000891 | 0.016595 | 0.013792 | PLCG2/STAP1/CYBA/TREM2/PTPRC/IL1B/F2RL1/NCKAP1L/SPACA3/C3/IL2RG/MERTK | 12 |
| BP | GO:0071300 | cellular response to retinoic acid | 12/1200 | 66/18723 | 0.000891 | 0.016595 | 0.013792 | SLC6A4/TBX1/LYN/PTK2B/PCK1/WNT3/TESC/FGFR2/RET/WNT11/KLF4/FZD10 | 12 |
| BP | GO:0009247 | glycolipid biosynthetic process | 12/1200 | 68/18723 | 0.001171 | 0.020062 | 0.016674 | ST8SIA4/PIGK/PGAP1/ST3GAL1/B4GALT6/ST6GALNAC4/GBGT1/PGAP2/ST3GAL2/CWH43/B3GNT5/PIGA | 12 |
| BP | GO:0070227 | lymphocyte apoptotic process | 12/1200 | 72/18723 | 0.001952 | 0.029675 | 0.024662 | CD3G/ST3GAL1/LYN/PRKCQ/BTK/IL7R/CCL5/IL2RA/ADAM8/BMP4/IDO1/ADA | 12 |
| BP | GO:0032732 | positive regulation of interleukin-1 production | 12/1200 | 73/18723 | 0.002202 | 0.032138 | 0.02671 | NLRC4/AIM2/P2RX7/LILRA5/F2RL1/PANX2/IL17A/TYROBP/SAA1/FZD5/MNDA/LILRA2 | 12 |
| BP | GO:0001523 | retinoid metabolic process | 12/1200 | 81/18723 | 0.005299 | 0.056864 | 0.047259 | BCO1/CRABP2/AKR1B1/CYP2W1/ALDH1A1/RDH12/CYP2C18/CYP3A7/CYP3A5/ADH7/AKR1B10/PNLIP | 12 |
| BP | GO:0071260 | cellular response to mechanical stimulus | 12/1200 | 81/18723 | 0.005299 | 0.056864 | 0.047259 | CYBA/MAP3K1/MMP7/SLC2A1/IL1B/ENG/GJA1/KCNJ2/SLC38A2/GCLC/ANKRD1/WNT11 | 12 |
| CC | GO:0044304 | main axon | 12/1263 | 68/19550 | 0.001255 | 0.031831 | 0.028642 | CCK/ERMN/MAPT/DLG2/MAP2/TIAM1/ROBO2/KCNA2/PARD3/NRCAM/DLG1/SPOCK1 | 12 |
| BP | GO:0030574 | collagen catabolic process | 11/1200 | 42/18723 | 4.84E-05 | 0.002074 | 0.001724 | CTSS/MMP3/MMP10/MMP7/MMP1/MMP9/ADAMTS3/MMP27/CTSL/MMP12/MMP13 | 11 |
| BP | GO:0002673 | regulation of acute inflammatory response | 11/1200 | 48/18723 | 0.000179 | 0.005291 | 0.004397 | FUT7/ALOX5AP/F12/BTK/IL1B/FFAR2/PIK3CG/C3/PLA2G2D/ADAM8/TNFSF11 | 11 |
| BP | GO:0008206 | bile acid metabolic process | 11/1200 | 49/18723 | 0.000218 | 0.006093 | 0.005064 | OSBPL3/CYP7A1/FGFR4/FGF19/OSBPL6/OSBPL1A/SLC27A2/STAR/CYP39A1/SULT2A1/CES1 | 11 |
| BP | GO:0002707 | negative regulation of lymphocyte mediated immunity | 11/1200 | 53/18723 | 0.000451 | 0.010055 | 0.008357 | CR2/SLAMF1/CR1L/SUSD4/PTPRC/LILRB1/NCKAP1L/CR1/IL7R/ARRB2/CD46 | 11 |
| BP | GO:0070228 | regulation of lymphocyte apoptotic process | 11/1200 | 54/18723 | 0.000533 | 0.011566 | 0.009612 | CD3G/ST3GAL1/LYN/PRKCQ/BTK/IL7R/CCL5/ADAM8/BMP4/IDO1/ADA | 11 |
| BP | GO:1905517 | macrophage migration | 11/1200 | 55/18723 | 0.000629 | 0.012797 | 0.010635 | STAP1/SLAMF1/TRIM55/CD200/TREM2/PTK2B/P2RX4/CCL5/SAA1/C3AR1/EDN2 | 11 |
| BP | GO:0002820 | negative regulation of adaptive immune response | 11/1200 | 59/18723 | 0.001163 | 0.019993 | 0.016616 | SAMSN1/CR2/SLAMF1/CR1L/SUSD4/PTPRC/LILRB1/NCKAP1L/CR1/IL7R/CD46 | 11 |
| BP | GO:0032835 | glomerulus development | 11/1200 | 62/18723 | 0.001772 | 0.027765 | 0.023075 | COL4A4/SULF2/JAG1/NID1/PECAM1/PODXL/LGR4/COL4A3/BMP4/SERPINB7/RET | 11 |
| BP | GO:0045576 | mast cell activation | 11/1200 | 62/18723 | 0.001772 | 0.027765 | 0.023075 | CD226/LAT2/RHOH/LYN/BTK/FGR/PIK3CG/MILR1/PIK3CD/PLA2G3/IL13RA2 | 11 |
| BP | GO:0022617 | extracellular matrix disassembly | 11/1200 | 63/18723 | 0.002025 | 0.030201 | 0.0251 | CTSS/MMP3/MMP10/MMP7/MMP1/MMP9/FGFR4/ADAM8/KLK7/MMP12/MMP13 | 11 |
| BP | GO:0002720 | positive regulation of cytokine production involved in immune response | 11/1200 | 65/18723 | 0.002619 | 0.03613 | 0.030027 | CD226/PLCG2/SLAMF1/IL1B/SCIMP/LILRB1/FFAR2/F2RL1/SEMA7A/FZD5/SLC7A5 | 11 |
| BP | GO:0071677 | positive regulation of mononuclear cell migration | 11/1200 | 65/18723 | 0.002619 | 0.03613 | 0.030027 | WNK1/SLAMF1/STK39/ITGA4/AIF1/PTK2B/CCL5/PLA2G7/ADAM8/CXCL13/S100A14 | 11 |
| BP | GO:0042130 | negative regulation of T cell proliferation | 11/1200 | 67/18723 | 0.003346 | 0.044429 | 0.036925 | LILRB2/CD86/GPNMB/CTLA4/LILRB1/CR1/CD80/PLA2G2D/IL2RA/BMP4/DLG1 | 11 |
| BP | GO:0050805 | negative regulation of synaptic transmission | 11/1200 | 69/18723 | 0.004226 | 0.049664 | 0.041275 | LILRB2/CD38/MAPT/SLC6A4/NPY2R/PTK2B/IL1B/PCDH17/ABHD6/BCHE/PLK2 | 11 |
| BP | GO:1903036 | positive regulation of response to wounding | 11/1200 | 72/18723 | 0.005882 | 0.060085 | 0.049936 | XBP1/CXCR4/F12/CLDN3/KANK1/ENPP4/OCLN/PTN/FERMT1/ADRA2A/F2 | 11 |
| CC | GO:0001772 | immunological synapse | 11/1263 | 44/19550 | 8.29E-05 | 0.003557 | 0.003201 | CD53/GZMA/RHOH/SKAP1/PRKCQ/CD37/SCIMP/VAV3/LCK/GZMB/DLG1 | 11 |
| MF | GO:0051787 | misfolded protein binding | 11/1229 | 29/18368 | 1.30E-06 | 0.00035 | 0.000315 | EDEM1/HSPA2/HSPA13/DNAJC10/SDF2L1/F12/DNAJB9/HSPA1B/DNAJC3/HSPA1A/HSPA1L | 11 |
| MF | GO:0098632 | cell-cell adhesion mediator activity | 11/1229 | 49/18368 | 0.000316 | 0.013196 | 0.011909 | DSP/IGSF9/DSC2/PKP3/TRIM29/KRT18/CD200/EMB/JUP/CXADR/EPCAM | 11 |
| MF | GO:0098631 | cell adhesion mediator activity | 11/1229 | 59/18368 | 0.001644 | 0.042817 | 0.038639 | DSP/IGSF9/DSC2/PKP3/TRIM29/KRT18/CD200/EMB/JUP/CXADR/EPCAM | 11 |
| BP | GO:0002335 | mature B cell differentiation | 10/1200 | 33/18723 | 2.68E-05 | 0.001329 | 0.001105 | CD19/POU2AF1/XBP1/PLCG2/IL21/ST3GAL1/PTK2B/GPR183/CR1/ADA | 10 |
| BP | GO:0006699 | bile acid biosynthetic process | 10/1200 | 36/18723 | 6.16E-05 | 0.00248 | 0.002061 | OSBPL3/CYP7A1/FGFR4/FGF19/OSBPL6/OSBPL1A/SLC27A2/STAR/CYP39A1/CES1 | 10 |
| BP | GO:0045730 | respiratory burst | 10/1200 | 37/18723 | 7.96E-05 | 0.002878 | 0.002392 | CYBA/NCF4/JCHAIN/TREM2/CYBB/CD52/PIK3CG/NCF2/MPO/PIK3CD | 10 |
| BP | GO:0032941 | secretion by tissue | 10/1200 | 38/18723 | 0.000102 | 0.003427 | 0.002848 | WNK1/NPR3/STK39/CYBA/KCNN4/AGR2/NLRP6/STATH/ADA/NEUROG1 | 10 |
| BP | GO:2000404 | regulation of T cell migration | 10/1200 | 42/18723 | 0.000251 | 0.0067 | 0.005568 | WNK1/STK39/ITGA4/AIF1/CD200/C10orf99/CCL5/ADAM8/CXCL13/ECM1 | 10 |
| BP | GO:0001774 | microglial cell activation | 10/1200 | 47/18723 | 0.00066 | 0.013242 | 0.011005 | MAPT/STAP1/AIF1/TREM2/CST7/PTPRC/TLR2/TLR1/TYROBP/C1QA | 10 |
| BP | GO:0097028 | dendritic cell differentiation | 10/1200 | 47/18723 | 0.00066 | 0.013242 | 0.011005 | LILRB2/IRF4/BATF/TMEM176B/FLT3/TREM2/LYN/LILRB1/F2RL1/TMEM176A | 10 |
| BP | GO:0050832 | defense response to fungus | 10/1200 | 49/18723 | 0.000932 | 0.017006 | 0.014133 | PLCG2/CLEC4D/C10orf99/IL17A/CLEC4E/MPO/DCD/LTF/CLEC6A/GNLY | 10 |
| BP | GO:0038093 | Fc receptor signaling pathway | 10/1200 | 50/18723 | 0.001099 | 0.019261 | 0.016008 | MYO1G/PLCG2/VAV1/MAP3K1/LYN/PRKCQ/PTPRC/BTK/VAV3/FGR | 10 |
| BP | GO:0042572 | retinol metabolic process | 10/1200 | 50/18723 | 0.001099 | 0.019261 | 0.016008 | BCO1/AKR1B1/ALDH1A1/RDH12/CYP2C18/CYP3A7/CYP3A5/ADH7/AKR1B10/PNLIP | 10 |
| BP | GO:0032964 | collagen biosynthetic process | 10/1200 | 51/18723 | 0.001289 | 0.021675 | 0.018014 | NPPC/ENG/ADAMTS3/TGFB3/CYP7A1/ARRB2/BMP4/TRAM2/SERPINB7/F2 | 10 |
| BP | GO:0002823 | negative regulation of adaptive immune response based on somatic recombination of immune receptors built from immunoglobulin superfamily domains | 10/1200 | 54/18723 | 0.002029 | 0.030201 | 0.0251 | CR2/SLAMF1/CR1L/SUSD4/PTPRC/LILRB1/NCKAP1L/CR1/IL7R/CD46 | 10 |
| BP | GO:0002886 | regulation of myeloid leukocyte mediated immunity | 10/1200 | 56/18723 | 0.002688 | 0.0368 | 0.030584 | STAP1/LYN/BTK/FGR/F2RL1/C3/CXCL6/TYROBP/PLA2G3/IL13RA2 | 10 |
| BP | GO:0031424 | keratinization | 10/1200 | 58/18723 | 0.003508 | 0.045566 | 0.037869 | KRT16/KRT2/LIPK/SHARPIN/PPL/LIPM/LCE3B/SPRR1A/TGM3/TCHH | 10 |
| BP | GO:0090303 | positive regulation of wound healing | 10/1200 | 59/18723 | 0.003987 | 0.048691 | 0.040466 | XBP1/CXCR4/F12/CLDN3/KANK1/ENPP4/OCLN/FERMT1/ADRA2A/F2 | 10 |
| BP | GO:0002712 | regulation of B cell mediated immunity | 10/1200 | 60/18723 | 0.004516 | 0.051638 | 0.042916 | CD226/CR2/CR1L/SUSD4/TREM2/PTPRC/BTK/CR1/C3/CD46 | 10 |
| BP | GO:0002889 | regulation of immunoglobulin mediated immune response | 10/1200 | 60/18723 | 0.004516 | 0.051638 | 0.042916 | CD226/CR2/CR1L/SUSD4/TREM2/PTPRC/BTK/CR1/C3/CD46 | 10 |
| BP | GO:0019748 | secondary metabolic process | 10/1200 | 61/18723 | 0.005098 | 0.056103 | 0.046627 | AKR1B1/CYP2W1/DCT/TYRP1/CYP3A5/STAR/AKR1B10/SLC7A11/FMO2/TYR | 10 |
| BP | GO:0043030 | regulation of macrophage activation | 10/1200 | 61/18723 | 0.005098 | 0.056103 | 0.046627 | STAP1/MFHAS1/CD200/TREM2/CST7/PTPRC/SPACA3/LRFN5/PLA2G10/IL31RA | 10 |
| BP | GO:0032613 | interleukin-10 production | 10/1200 | 62/18723 | 0.005738 | 0.059299 | 0.049282 | IRF4/PLCG2/TREM2/TLR2/LILRB1/LILRA5/F2RL1/TYROBP/CD40LG/CD46 | 10 |
| BP | GO:0032615 | interleukin-12 production | 10/1200 | 62/18723 | 0.005738 | 0.059299 | 0.049282 | PLCG2/SLAMF1/TLR2/LTB/SCIMP/LILRB1/LILRA5/IL17A/ARRB2/CD40LG | 10 |
| BP | GO:0032623 | interleukin-2 production | 10/1200 | 62/18723 | 0.005738 | 0.059299 | 0.049282 | IRF4/PLCG2/CD86/PRKCQ/PTPRC/IL1B/IL17F/CR1/CD80/PDE4B | 10 |
| BP | GO:0032653 | regulation of interleukin-10 production | 10/1200 | 62/18723 | 0.005738 | 0.059299 | 0.049282 | IRF4/PLCG2/TREM2/TLR2/LILRB1/LILRA5/F2RL1/TYROBP/CD40LG/CD46 | 10 |
| BP | GO:0032655 | regulation of interleukin-12 production | 10/1200 | 62/18723 | 0.005738 | 0.059299 | 0.049282 | PLCG2/SLAMF1/TLR2/LTB/SCIMP/LILRB1/LILRA5/IL17A/ARRB2/CD40LG | 10 |
| BP | GO:0032663 | regulation of interleukin-2 production | 10/1200 | 62/18723 | 0.005738 | 0.059299 | 0.049282 | IRF4/PLCG2/CD86/PRKCQ/PTPRC/IL1B/IL17F/CR1/CD80/PDE4B | 10 |
| BP | GO:0032731 | positive regulation of interleukin-1 beta production | 10/1200 | 62/18723 | 0.005738 | 0.059299 | 0.049282 | NLRC4/AIM2/P2RX7/LILRA5/F2RL1/IL17A/TYROBP/FZD5/MNDA/LILRA2 | 10 |
| BP | GO:0032757 | positive regulation of interleukin-8 production | 10/1200 | 62/18723 | 0.005738 | 0.059299 | 0.049282 | TLR2/IL1B/CD14/TLR1/FFAR2/FCN1/F2RL1/HSPA1B/HSPA1A/LILRA2 | 10 |
| CC | GO:0030057 | desmosome | 10/1263 | 25/19550 | 1.63E-06 | 0.000182 | 0.000164 | DSP/DSC2/PKP3/DSC3/PERP/JUP/POF1B/PKP1/DSG3/PPL | 10 |
| MF | GO:0043394 | proteoglycan binding | 10/1229 | 36/18368 | 8.85E-05 | 0.005416 | 0.004887 | CTSS/GPNMB/NID1/PTPRC/PTPRF/FCN2/CFH/CTSL/PLA2G2D/PTN | 10 |
| MF | GO:0016712 | oxidoreductase activity, acting on paired donors, with incorporation or reduction of molecular oxygen, reduced flavin or flavoprotein as one donor, and incorporation of one atom of oxygen | 10/1229 | 40/18368 | 0.000231 | 0.010657 | 0.009617 | CYP2W1/CYP4F8/CYP7A1/CYP2C18/CYP2J2/CYP3A7/CYP3A5/CYP4F12/CYP4F11/CYP39A1 | 10 |
| MF | GO:0008009 | chemokine activity | 10/1229 | 49/18368 | 0.001293 | 0.039565 | 0.035705 | CXCL1/CXCL3/CXCL2/C10orf99/CCL5/CXCL6/CCL18/CXCL13/CCL13/PF4V1 | 10 |
| BP | GO:0030449 | regulation of complement activation | 9/1200 | 21/18723 | 2.58E-06 | 0.000221 | 0.000183 | CR2/CR1L/SUSD4/TREM2/IL1B/CFH/CR1/C3/CD46 | 9 |
| BP | GO:0002922 | positive regulation of humoral immune response | 9/1200 | 24/18723 | 9.61E-06 | 0.000608 | 0.000505 | TREM2/PTPRC/IL1B/IL17F/CR1/IL17A/C3/KLK7/PGC | 9 |
| BP | GO:0045109 | intermediate filament organization | 9/1200 | 25/18723 | 1.42E-05 | 0.000839 | 0.000697 | DSP/BFSP2/PKP1/KRT2/NEFM/DNAJB6/NEFL/KRT25/TCHH | 9 |
| BP | GO:0002313 | mature B cell differentiation involved in immune response | 9/1200 | 28/18723 | 4.02E-05 | 0.00178 | 0.001479 | POU2AF1/XBP1/PLCG2/IL21/ST3GAL1/PTK2B/GPR183/CR1/ADA | 9 |
| BP | GO:0035590 | purinergic nucleotide receptor signaling pathway | 9/1200 | 34/18723 | 0.000215 | 0.006054 | 0.005031 | P2RY1/GPR34/P2RY10/P2RX5/P2RX4/P2RY13/P2RX1/P2RX7/ADA | 9 |
| BP | GO:0051482 | positive regulation of cytosolic calcium ion concentration involved in phospholipase C-activating G protein-coupled signaling pathway | 9/1200 | 34/18723 | 0.000215 | 0.006054 | 0.005031 | P2RY10/GPR65/GPR174/GPR18/F2RL1/LPAR6/C3AR1/AGTR1/TGM2 | 9 |
| BP | GO:0034122 | negative regulation of toll-like receptor signaling pathway | 9/1200 | 40/18723 | 0.000793 | 0.015436 | 0.012829 | IRF4/MFHAS1/TREM2/LYN/LGR4/F2RL1/ARRB2/NLRP6/LILRA2 | 9 |
| BP | GO:0150077 | regulation of neuroinflammatory response | 9/1200 | 40/18723 | 0.000793 | 0.015436 | 0.012829 | MMP3/PLCG2/STAP1/CD200/TREM2/CST7/PTPRC/IL1B/MMP9 | 9 |
| BP | GO:0030890 | positive regulation of B cell proliferation | 9/1200 | 42/18723 | 0.001153 | 0.019878 | 0.016521 | CD38/IL21/FCRL3/PTPRC/GPR183/VAV3/NCKAP1L/NFATC2/ADA | 9 |
| BP | GO:0002861 | regulation of inflammatory response to antigenic stimulus | 9/1200 | 43/18723 | 0.001376 | 0.022787 | 0.018938 | FUT7/TREM2/LYN/BTK/FGR/C3/PLA2G2D/NLRP6/PLK2 | 9 |
| BP | GO:0150076 | neuroinflammatory response | 9/1200 | 44/18723 | 0.001633 | 0.026287 | 0.021847 | MMP3/PLCG2/STAP1/CD200/TREM2/CST7/PTPRC/IL1B/MMP9 | 9 |
| BP | GO:2000107 | negative regulation of leukocyte apoptotic process | 9/1200 | 46/18723 | 0.002262 | 0.03292 | 0.027359 | HCLS1/ST3GAL1/PRKCQ/LILRB1/IL7R/CCL5/BMP4/MERTK/ADA | 9 |
| BP | GO:0043303 | mast cell degranulation | 9/1200 | 48/18723 | 0.00307 | 0.041178 | 0.034222 | LAT2/LYN/BTK/FGR/PIK3CG/MILR1/PIK3CD/PLA2G3/IL13RA2 | 9 |
| BP | GO:0002279 | mast cell activation involved in immune response | 9/1200 | 49/18723 | 0.003551 | 0.045842 | 0.038099 | LAT2/LYN/BTK/FGR/PIK3CG/MILR1/PIK3CD/PLA2G3/IL13RA2 | 9 |
| BP | GO:0002448 | mast cell mediated immunity | 9/1200 | 50/18723 | 0.004089 | 0.049479 | 0.041121 | LAT2/LYN/BTK/FGR/PIK3CG/MILR1/PIK3CD/PLA2G3/IL13RA2 | 9 |
| BP | GO:0043268 | positive regulation of potassium ion transport | 9/1200 | 50/18723 | 0.004089 | 0.049479 | 0.041121 | WNK1/STK39/WNK3/TREM2/KCNN4/KCNJ2/DLG1/ADRA2A/RNF207 | 9 |
| BP | GO:0070231 | T cell apoptotic process | 9/1200 | 50/18723 | 0.004089 | 0.049479 | 0.041121 | ST3GAL1/PRKCQ/IL7R/CCL5/IL2RA/ADAM8/BMP4/IDO1/ADA | 9 |
| BP | GO:0006636 | unsaturated fatty acid biosynthetic process | 9/1200 | 51/18723 | 0.004689 | 0.052883 | 0.04395 | DEGS1/IL1B/ELOVL4/ALOX15B/ELOVL2/SCD/PLA2G3/ELOVL6/EDN2 | 9 |
| MF | GO:0001530 | lipopolysaccharide binding | 9/1229 | 33/18368 | 0.000232 | 0.010657 | 0.009617 | LY96/TREM2/TLR2/CD14/SELP/P2RX7/BPIFC/LTF/F2 | 9 |
| MF | GO:0005504 | fatty acid binding | 9/1229 | 39/18368 | 0.000887 | 0.02909 | 0.026251 | APOC1/ALOX5AP/UCP1/ACOXL/FABP4/PPARG/GPR31/GSTA1/CYP4F11 | 9 |
| BP | GO:0070269 | pyroptosis | 8/1200 | 21/18723 | 2.67E-05 | 0.001329 | 0.001105 | NLRC4/ZBP1/GZMA/TREM2/AIM2/GSDMC/GZMB/NLRP6 | 8 |
| BP | GO:0002220 | innate immune response activating cell surface receptor signaling pathway | 8/1200 | 22/18723 | 3.96E-05 | 0.001772 | 0.001473 | PLCG2/LYN/FFAR2/FCN1/PAK3/TYROBP/CLEC6A/LILRA2 | 8 |
| BP | GO:0002758 | innate immune response-activating signal transduction | 8/1200 | 23/18723 | 5.72E-05 | 0.002357 | 0.001959 | PLCG2/LYN/FFAR2/FCN1/PAK3/TYROBP/CLEC6A/LILRA2 | 8 |
| BP | GO:0050857 | positive regulation of antigen receptor-mediated signaling pathway | 8/1200 | 26/18723 | 0.000153 | 0.004728 | 0.00393 | CD226/STAP1/PTPRC/PRKCB/KCNN4/TRAT1/LCK/ADA | 8 |
| BP | GO:0002675 | positive regulation of acute inflammatory response | 8/1200 | 28/18723 | 0.000272 | 0.007159 | 0.005949 | ALOX5AP/BTK/IL1B/FFAR2/PIK3CG/C3/ADAM8/TNFSF11 | 8 |
| BP | GO:0003382 | epithelial cell morphogenesis | 8/1200 | 33/18723 | 0.000913 | 0.016788 | 0.013952 | RAB25/POF1B/CLDN3/ARHGEF26/GRHL2/VSIG1/COL15A1/TNMD | 8 |
| BP | GO:0032743 | positive regulation of interleukin-2 production | 8/1200 | 34/18723 | 0.001128 | 0.019519 | 0.016222 | IRF4/PLCG2/CD86/PRKCQ/PTPRC/IL1B/CD80/PDE4B | 8 |
| BP | GO:0050869 | negative regulation of B cell activation | 8/1200 | 34/18723 | 0.001128 | 0.019519 | 0.016222 | SAMSN1/BANK1/CTLA4/LYN/BTK/CR1/TYROBP/MNDA | 8 |
| BP | GO:0070232 | regulation of T cell apoptotic process | 8/1200 | 34/18723 | 0.001128 | 0.019519 | 0.016222 | ST3GAL1/PRKCQ/IL7R/CCL5/ADAM8/BMP4/IDO1/ADA | 8 |
| BP | GO:0018149 | peptide cross-linking | 8/1200 | 35/18723 | 0.001381 | 0.022787 | 0.018938 | DSP/KRT1/TGM7/KRT2/KRT10/TGM2/SPRR1A/TGM3 | 8 |
| BP | GO:2000403 | positive regulation of lymphocyte migration | 8/1200 | 35/18723 | 0.001381 | 0.022787 | 0.018938 | WNK1/STK39/ITGA4/AIF1/PTK2B/CCL5/ADAM8/CXCL13 | 8 |
| BP | GO:0090218 | positive regulation of lipid kinase activity | 8/1200 | 37/18723 | 0.002022 | 0.030201 | 0.0251 | CD19/FLT3/AGAP2/LYN/PTK2B/VAV3/FGR/F2 | 8 |
| BP | GO:0051084 | 'de novo' posttranslational protein folding | 8/1200 | 39/18723 | 0.002876 | 0.039116 | 0.032509 | HSPA2/HSPA13/SDF2L1/UGGT1/DNAJB1/HSPA1B/HSPA1A/HSPA1L | 8 |
| BP | GO:0032733 | positive regulation of interleukin-10 production | 8/1200 | 41/18723 | 0.003987 | 0.048691 | 0.040466 | IRF4/PLCG2/TREM2/TLR2/LILRA5/F2RL1/CD40LG/CD46 | 8 |
| BP | GO:0048286 | lung alveolus development | 8/1200 | 41/18723 | 0.003987 | 0.048691 | 0.040466 | TGFB3/HOPX/FGFR2/BMP4/SLC7A11/MMP12/ADA/EDN2 | 8 |
| BP | GO:1905521 | regulation of macrophage migration | 8/1200 | 41/18723 | 0.003987 | 0.048691 | 0.040466 | STAP1/SLAMF1/CD200/TREM2/PTK2B/P2RX4/CCL5/C3AR1 | 8 |
| BP | GO:0032965 | regulation of collagen biosynthetic process | 8/1200 | 42/18723 | 0.004654 | 0.052603 | 0.043718 | NPPC/ENG/TGFB3/CYP7A1/ARRB2/BMP4/SERPINB7/F2 | 8 |
| BP | GO:0006458 | 'de novo' protein folding | 8/1200 | 43/18723 | 0.005404 | 0.057408 | 0.047711 | HSPA2/HSPA13/SDF2L1/UGGT1/DNAJB1/HSPA1B/HSPA1A/HSPA1L | 8 |
| BP | GO:0009268 | response to pH | 8/1200 | 43/18723 | 0.005404 | 0.057408 | 0.047711 | CTSS/RAB11B/GPR65/PCK1/ACER1/GJA1/GPR31/SST | 8 |
| MF | GO:0001614 | purinergic nucleotide receptor activity | 8/1229 | 21/18368 | 3.64E-05 | 0.003033 | 0.002737 | P2RY1/GPR34/P2RY10/P2RX5/P2RX4/P2RY13/P2RX1/P2RX7 | 8 |
| MF | GO:0016502 | nucleotide receptor activity | 8/1229 | 21/18368 | 3.64E-05 | 0.003033 | 0.002737 | P2RY1/GPR34/P2RY10/P2RX5/P2RX4/P2RY13/P2RX1/P2RX7 | 8 |
| MF | GO:0038187 | pattern recognition receptor activity | 8/1229 | 26/18368 | 0.000207 | 0.010542 | 0.009513 | LY96/CLEC4D/TLR2/CD14/FCN1/CLEC4E/CLEC6A/MARCO | 8 |
| MF | GO:0051861 | glycolipid binding | 8/1229 | 28/18368 | 0.000365 | 0.014558 | 0.013137 | PIGK/HSPA2/SELL/TREM2/LYN/DPEP1/SELP/CLEC4E | 8 |
| BP | GO:0033631 | cell-cell adhesion mediated by integrin | 7/1200 | 16/18723 | 2.99E-05 | 0.001417 | 0.001178 | WNK1/ITGA4/SKAP1/PODXL/CCL5/CXCL13/ADA | 7 |
| BP | GO:0002923 | regulation of humoral immune response mediated by circulating immunoglobulin | 7/1200 | 18/18723 | 7.42E-05 | 0.002849 | 0.002368 | CR2/CR1L/SUSD4/TREM2/PTPRC/CR1/CD46 | 7 |
| BP | GO:0034134 | toll-like receptor 2 signaling pathway | 7/1200 | 18/18723 | 7.42E-05 | 0.002849 | 0.002368 | MFHAS1/CYBA/TREM2/LYN/TLR2/TLR1/F2RL1 | 7 |
| BP | GO:0046629 | gamma-delta T cell activation | 7/1200 | 20/18723 | 0.000161 | 0.004926 | 0.004094 | PTPRC/CXADR/LILRB1/GPR18/ITK/NCKAP1L/EGR3 | 7 |
| BP | GO:0033630 | positive regulation of cell adhesion mediated by integrin | 7/1200 | 21/18723 | 0.000229 | 0.006294 | 0.005231 | SKAP1/PODXL/NCKAP1L/CCL5/CXCL13/FERMT1/RET | 7 |
| BP | GO:0033081 | regulation of T cell differentiation in thymus | 7/1200 | 23/18723 | 0.000431 | 0.009775 | 0.008123 | IL7R/EGR3/VNN1/ADAM8/BMP4/ADA/TOX | 7 |
| BP | GO:0038095 | Fc-epsilon receptor signaling pathway | 7/1200 | 24/18723 | 0.000574 | 0.01205 | 0.010015 | PLCG2/VAV1/MAP3K1/LYN/PRKCQ/BTK/VAV3 | 7 |
| BP | GO:0070841 | inclusion body assembly | 7/1200 | 24/18723 | 0.000574 | 0.01205 | 0.010015 | MAPT/HSPA2/DNAJB1/HSPA1B/DNAJB6/HSPA1A/DNAJA4 | 7 |
| BP | GO:0036037 | CD8-positive, alpha-beta T cell activation | 7/1200 | 27/18723 | 0.001245 | 0.021125 | 0.017557 | PSMB11/LILRB1/GPR18/NCKAP1L/EOMES/TNFSF8/TOX | 7 |
| BP | GO:0010464 | regulation of mesenchymal cell proliferation | 7/1200 | 28/18723 | 0.001569 | 0.025487 | 0.021182 | CTNNBIP1/MYC/TBX1/FGFR2/PTN/BMP4/WNT11 | 7 |
| BP | GO:0010818 | T cell chemotaxis | 7/1200 | 28/18723 | 0.001569 | 0.025487 | 0.021182 | WNK1/STK39/GPR183/CCL5/PIK3CG/CXCL13/PIK3CD | 7 |
| BP | GO:0030970 | retrograde protein transport, ER to cytosol | 7/1200 | 29/18723 | 0.001955 | 0.029675 | 0.024662 | ERLEC1/EDEM1/UBE2J1/SEL1L/RHBDD1/HERPUD1/SVIP | 7 |
| BP | GO:1903513 | endoplasmic reticulum to cytosol transport | 7/1200 | 29/18723 | 0.001955 | 0.029675 | 0.024662 | ERLEC1/EDEM1/UBE2J1/SEL1L/RHBDD1/HERPUD1/SVIP | 7 |
| BP | GO:2000406 | positive regulation of T cell migration | 7/1200 | 29/18723 | 0.001955 | 0.029675 | 0.024662 | WNK1/STK39/ITGA4/AIF1/CCL5/ADAM8/CXCL13 | 7 |
| BP | GO:0034368 | protein-lipid complex remodeling | 7/1200 | 30/18723 | 0.002411 | 0.034523 | 0.028692 | APOC1/PLA2G7/MPO/PLA2G3/PLA2G10/AGTR1/PLTP | 7 |
| BP | GO:0034369 | plasma lipoprotein particle remodeling | 7/1200 | 30/18723 | 0.002411 | 0.034523 | 0.028692 | APOC1/PLA2G7/MPO/PLA2G3/PLA2G10/AGTR1/PLTP | 7 |
| BP | GO:0045066 | regulatory T cell differentiation | 7/1200 | 31/18723 | 0.002945 | 0.039702 | 0.032996 | LILRB2/FUT7/CTLA4/CR1/PLA2G2D/CD46/TOX | 7 |
| BP | GO:0019835 | cytolysis | 7/1200 | 32/18723 | 0.003564 | 0.045842 | 0.038099 | GZMA/PRF1/CR1/GZMH/LYZ/GZMB/F2 | 7 |
| BP | GO:0034367 | protein-containing complex remodeling | 7/1200 | 32/18723 | 0.003564 | 0.045842 | 0.038099 | APOC1/PLA2G7/MPO/PLA2G3/PLA2G10/AGTR1/PLTP | 7 |
| BP | GO:0050850 | positive regulation of calcium-mediated signaling | 7/1200 | 32/18723 | 0.003564 | 0.045842 | 0.038099 | CHP2/PLCG2/TREM2/P2RX5/P2RX4/TRAT1/ADA | 7 |
| BP | GO:0002431 | Fc receptor mediated stimulatory signaling pathway | 7/1200 | 33/18723 | 0.004278 | 0.049942 | 0.041506 | MYO1G/CD226/VAV1/LYN/PTPRC/VAV3/FGR | 7 |
| BP | GO:0043552 | positive regulation of phosphatidylinositol 3-kinase activity | 7/1200 | 33/18723 | 0.004278 | 0.049942 | 0.041506 | CD19/FLT3/AGAP2/LYN/PTK2B/VAV3/FGR | 7 |
| BP | GO:0000038 | very long-chain fatty acid metabolic process | 7/1200 | 34/18723 | 0.005094 | 0.056103 | 0.046627 | ACOT2/ELOVL4/SLC27A6/SLC27A2/CYP4F12/ELOVL2/ELOVL6 | 7 |
| BP | GO:0031128 | developmental induction | 7/1200 | 34/18723 | 0.005094 | 0.056103 | 0.046627 | MESP1/ROBO2/WNT2B/WNT3/FZD5/BMP4/WNT11 | 7 |
| BP | GO:0051085 | chaperone cofactor-dependent protein refolding | 7/1200 | 34/18723 | 0.005094 | 0.056103 | 0.046627 | HSPA2/HSPA13/SDF2L1/DNAJB1/HSPA1B/HSPA1A/HSPA1L | 7 |
| BP | GO:0098751 | bone cell development | 7/1200 | 34/18723 | 0.005094 | 0.056103 | 0.046627 | MEIS1/LILRB1/ZNF385A/FBN1/TYROBP/LTF/TNFSF11 | 7 |
| MF | GO:0032396 | inhibitory MHC class I receptor activity | 7/1229 | 12/18368 | 3.47E-06 | 0.000637 | 0.000575 | LILRB2/LILRB3/LILRB1/LILRA5/LILRA6/LILRA1/LILRA2 | 7 |
| MF | GO:0032393 | MHC class I receptor activity | 7/1229 | 17/18368 | 6.32E-05 | 0.004465 | 0.00403 | LILRB2/LILRB3/LILRB1/LILRA5/LILRA6/LILRA1/LILRA2 | 7 |
| BP | GO:0033632 | regulation of cell-cell adhesion mediated by integrin | 6/1200 | 11/18723 | 2.39E-05 | 0.001245 | 0.001035 | WNK1/SKAP1/PODXL/CCL5/CXCL13/ADA | 6 |
| BP | GO:0090084 | negative regulation of inclusion body assembly | 6/1200 | 11/18723 | 2.39E-05 | 0.001245 | 0.001035 | HSPA2/DNAJB1/HSPA1B/DNAJB6/HSPA1A/DNAJA4 | 6 |
| BP | GO:0034135 | regulation of toll-like receptor 2 signaling pathway | 6/1200 | 13/18723 | 7.94E-05 | 0.002878 | 0.002392 | MFHAS1/CYBA/TREM2/LYN/TLR1/F2RL1 | 6 |
| BP | GO:0060100 | positive regulation of phagocytosis, engulfment | 6/1200 | 13/18723 | 7.94E-05 | 0.002878 | 0.002392 | PLCG2/STAP1/TREM2/F2RL1/NCKAP1L/C3 | 6 |
| BP | GO:1905155 | positive regulation of membrane invagination | 6/1200 | 13/18723 | 7.94E-05 | 0.002878 | 0.002392 | PLCG2/STAP1/TREM2/F2RL1/NCKAP1L/C3 | 6 |
| BP | GO:0043374 | CD8-positive, alpha-beta T cell differentiation | 6/1200 | 14/18723 | 0.000131 | 0.004197 | 0.003488 | PSMB11/GPR18/NCKAP1L/EOMES/TNFSF8/TOX | 6 |
| BP | GO:0060099 | regulation of phagocytosis, engulfment | 6/1200 | 15/18723 | 0.000207 | 0.005895 | 0.004899 | PLCG2/STAP1/TREM2/F2RL1/NCKAP1L/C3 | 6 |
| BP | GO:1905153 | regulation of membrane invagination | 6/1200 | 16/18723 | 0.000314 | 0.007939 | 0.006598 | PLCG2/STAP1/TREM2/F2RL1/NCKAP1L/C3 | 6 |
| BP | GO:0090083 | regulation of inclusion body assembly | 6/1200 | 17/18723 | 0.000459 | 0.010148 | 0.008434 | HSPA2/DNAJB1/HSPA1B/DNAJB6/HSPA1A/DNAJA4 | 6 |
| BP | GO:0002523 | leukocyte migration involved in inflammatory response | 6/1200 | 19/18723 | 0.0009 | 0.016595 | 0.013792 | SLAMF1/TRIM55/FUT7/FFAR2/PTN/ADAM8 | 6 |
| BP | GO:0070233 | negative regulation of T cell apoptotic process | 6/1200 | 19/18723 | 0.0009 | 0.016595 | 0.013792 | ST3GAL1/PRKCQ/IL7R/CCL5/BMP4/ADA | 6 |
| BP | GO:0150146 | cell junction disassembly | 6/1200 | 21/18723 | 0.001611 | 0.026094 | 0.021686 | TREM2/PIK3R1/TGFB3/C3/C1QA/C1QB | 6 |
| BP | GO:0060713 | labyrinthine layer morphogenesis | 6/1200 | 22/18723 | 0.002097 | 0.030976 | 0.025743 | GJB5/GRHL2/DNAJB6/FGFR2/FZD5/BMP5 | 6 |
| BP | GO:0042026 | protein refolding | 6/1200 | 23/18723 | 0.002685 | 0.0368 | 0.030584 | HSPA2/HSPA13/HSPA1B/HSPA1A/HSPA1L/DNAJA4 | 6 |
| BP | GO:0002438 | acute inflammatory response to antigenic stimulus | 6/1200 | 24/18723 | 0.003388 | 0.044657 | 0.037114 | FUT7/BTK/C3/PLA2G2D/NLRP6/IL31RA | 6 |
| BP | GO:0050855 | regulation of B cell receptor signaling pathway | 6/1200 | 24/18723 | 0.003388 | 0.044657 | 0.037114 | CD19/STAP1/LYN/FCRL3/PRKCB/LPXN | 6 |
| BP | GO:0003094 | glomerular filtration | 6/1200 | 25/18723 | 0.00422 | 0.049664 | 0.041275 | CYBA/SULF2/JCHAIN/RHPN2/F2RL1/GJA1 | 6 |
| BP | GO:0010447 | response to acidic pH | 6/1200 | 25/18723 | 0.00422 | 0.049664 | 0.041275 | CTSS/RAB11B/GPR65/PCK1/GPR31/SST | 6 |
| BP | GO:0031664 | regulation of lipopolysaccharide-mediated signaling pathway | 6/1200 | 25/18723 | 0.00422 | 0.049664 | 0.041275 | LY96/CD180/SCIMP/LY86/LTF/LILRA2 | 6 |
| BP | GO:0032753 | positive regulation of interleukin-4 production | 6/1200 | 25/18723 | 0.00422 | 0.049664 | 0.041275 | IRF4/CD86/CLECL1/PRKCQ/CD40LG/SLC7A5 | 6 |
| BP | GO:0033622 | integrin activation | 6/1200 | 25/18723 | 0.00422 | 0.049664 | 0.041275 | MZB1/PLEK/SKAP1/SELP/CXCL13/FERMT1 | 6 |
| BP | GO:0036315 | cellular response to sterol | 6/1200 | 25/18723 | 0.00422 | 0.049664 | 0.041275 | GRAMD1C/GRAMD1B/CYP7A1/RORC/HMGCS1/CES1 | 6 |
| BP | GO:0060706 | cell differentiation involved in embryonic placenta development | 6/1200 | 25/18723 | 0.00422 | 0.049664 | 0.041275 | KRT8/GJB5/GRHL2/DNAJB6/FZD5/EOMES | 6 |
| BP | GO:0062009 | secondary palate development | 6/1200 | 25/18723 | 0.00422 | 0.049664 | 0.041275 | TBX1/TGFB3/DLG1/WNT11/TGFBR3/SOX11 | 6 |
| BP | GO:0098581 | detection of external biotic stimulus | 6/1200 | 25/18723 | 0.00422 | 0.049664 | 0.041275 | LY96/NLRC4/TREM2/TLR2/TLR1/CLEC6A | 6 |
| BP | GO:0001573 | ganglioside metabolic process | 6/1200 | 26/18723 | 0.005193 | 0.056257 | 0.046754 | ST8SIA4/ST3GAL1/B4GALT6/NEU4/ST6GALNAC4/ST3GAL2 | 6 |
| BP | GO:0002407 | dendritic cell chemotaxis | 6/1200 | 26/18723 | 0.005193 | 0.056257 | 0.046754 | SLAMF1/CXCR4/GPR183/CCL5/PIK3CG/CXCR1 | 6 |
| BP | GO:0042537 | benzene-containing compound metabolic process | 6/1200 | 26/18723 | 0.005193 | 0.056257 | 0.046754 | AADAT/KYNU/ACSM1/TDO2/IDO1/STAR | 6 |
| BP | GO:0060669 | embryonic placenta morphogenesis | 6/1200 | 26/18723 | 0.005193 | 0.056257 | 0.046754 | GJB5/GRHL2/DNAJB6/FGFR2/FZD5/BMP5 | 6 |
| BP | GO:0097205 | renal filtration | 6/1200 | 26/18723 | 0.005193 | 0.056257 | 0.046754 | CYBA/SULF2/JCHAIN/RHPN2/F2RL1/GJA1 | 6 |
| CC | GO:0005639 | integral component of nuclear inner membrane | 6/1263 | 18/19550 | 0.000679 | 0.02105 | 0.018941 | TMEM43/SPAG4/P2RX5/P2RX4/P2RX1/P2RX7 | 6 |
| CC | GO:0031229 | intrinsic component of nuclear inner membrane | 6/1263 | 18/19550 | 0.000679 | 0.02105 | 0.018941 | TMEM43/SPAG4/P2RX5/P2RX4/P2RX1/P2RX7 | 6 |
| CC | GO:0097038 | perinuclear endoplasmic reticulum | 6/1263 | 21/19550 | 0.001679 | 0.03904 | 0.035129 | CYBA/OSBPL3/CYBB/PIK3R1/OSBPL6/CREB3L2 | 6 |
| MF | GO:0030280 | structural constituent of skin epidermis | 6/1229 | 15/18368 | 0.000262 | 0.011466 | 0.010348 | KRT1/PKP1/KRT2/KRT10/PNPLA1/SPRR1A | 6 |
| MF | GO:0045236 | CXCR chemokine receptor binding | 6/1229 | 18/18368 | 0.000818 | 0.027795 | 0.025083 | CXCL1/CXCL3/CXCL2/CXCL6/CXCL13/PF4V1 | 6 |
| BP | GO:0033089 | positive regulation of T cell differentiation in thymus | 5/1200 | 10/18723 | 0.000206 | 0.005895 | 0.004899 | IL7R/EGR3/VNN1/ADAM8/ADA | 5 |
| BP | GO:0001867 | complement activation, lectin pathway | 5/1200 | 11/18723 | 0.000358 | 0.008557 | 0.007111 | KRT1/FCN2/FCN1/FCN3/MBL2 | 5 |
| BP | GO:0002924 | negative regulation of humoral immune response mediated by circulating immunoglobulin | 5/1200 | 11/18723 | 0.000358 | 0.008557 | 0.007111 | CR2/CR1L/SUSD4/CR1/CD46 | 5 |
| BP | GO:0032308 | positive regulation of prostaglandin secretion | 5/1200 | 11/18723 | 0.000358 | 0.008557 | 0.007111 | IL1B/P2RX4/TNFSF11/PLA2G3/PLA2G10 | 5 |
| BP | GO:0070944 | neutrophil-mediated killing of bacterium | 5/1200 | 11/18723 | 0.000358 | 0.008557 | 0.007111 | F2RL1/TREM1/CXCL6/NLRP6/F2 | 5 |
| BP | GO:0032306 | regulation of prostaglandin secretion | 5/1200 | 12/18723 | 0.000581 | 0.01205 | 0.010015 | IL1B/P2RX4/TNFSF11/PLA2G3/PLA2G10 | 5 |
| BP | GO:0045916 | negative regulation of complement activation | 5/1200 | 12/18723 | 0.000581 | 0.01205 | 0.010015 | CR2/CR1L/SUSD4/CR1/CD46 | 5 |
| BP | GO:0070857 | regulation of bile acid biosynthetic process | 5/1200 | 12/18723 | 0.000581 | 0.01205 | 0.010015 | CYP7A1/FGFR4/FGF19/STAR/CES1 | 5 |
| BP | GO:0070943 | neutrophil-mediated killing of symbiont cell | 5/1200 | 12/18723 | 0.000581 | 0.01205 | 0.010015 | F2RL1/TREM1/CXCL6/NLRP6/F2 | 5 |
| BP | GO:2001204 | regulation of osteoclast development | 5/1200 | 12/18723 | 0.000581 | 0.01205 | 0.010015 | LILRB1/FBN1/TYROBP/LTF/TNFSF11 | 5 |
| BP | GO:0036005 | response to macrophage colony-stimulating factor | 5/1200 | 13/18723 | 0.000894 | 0.016595 | 0.013792 | STAP1/MST1R/TREM2/DOK1/TLR2 | 5 |
| BP | GO:0036006 | cellular response to macrophage colony-stimulating factor stimulus | 5/1200 | 13/18723 | 0.000894 | 0.016595 | 0.013792 | STAP1/MST1R/TREM2/DOK1/TLR2 | 5 |
| BP | GO:0051709 | regulation of killing of cells of other organism | 5/1200 | 13/18723 | 0.000894 | 0.016595 | 0.013792 | PRF1/P2RX7/F2RL1/CXCL6/BCL2L1 | 5 |
| BP | GO:0072672 | neutrophil extravasation | 5/1200 | 13/18723 | 0.000894 | 0.016595 | 0.013792 | FUT7/PECAM1/PIK3CG/ADAM8/PIK3CD | 5 |
| BP | GO:0070942 | neutrophil mediated cytotoxicity | 5/1200 | 14/18723 | 0.001318 | 0.022012 | 0.018294 | F2RL1/TREM1/CXCL6/NLRP6/F2 | 5 |
| BP | GO:0023035 | CD40 signaling pathway | 5/1200 | 15/18723 | 0.001873 | 0.028922 | 0.024037 | CD86/SLAMF1/TREM2/SHARPIN/CD40LG | 5 |
| BP | GO:0032310 | prostaglandin secretion | 5/1200 | 15/18723 | 0.001873 | 0.028922 | 0.024037 | IL1B/P2RX4/TNFSF11/PLA2G3/PLA2G10 | 5 |
| BP | GO:1904251 | regulation of bile acid metabolic process | 5/1200 | 15/18723 | 0.001873 | 0.028922 | 0.024037 | CYP7A1/FGFR4/FGF19/STAR/CES1 | 5 |
| BP | GO:0001574 | ganglioside biosynthetic process | 5/1200 | 16/18723 | 0.002582 | 0.035808 | 0.029759 | ST8SIA4/ST3GAL1/B4GALT6/ST6GALNAC4/ST3GAL2 | 5 |
| BP | GO:0002713 | negative regulation of B cell mediated immunity | 5/1200 | 16/18723 | 0.002582 | 0.035808 | 0.029759 | CR2/CR1L/SUSD4/CR1/CD46 | 5 |
| BP | GO:0002890 | negative regulation of immunoglobulin mediated immune response | 5/1200 | 16/18723 | 0.002582 | 0.035808 | 0.029759 | CR2/CR1L/SUSD4/CR1/CD46 | 5 |
| BP | GO:0002921 | negative regulation of humoral immune response | 5/1200 | 16/18723 | 0.002582 | 0.035808 | 0.029759 | CR2/CR1L/SUSD4/CR1/CD46 | 5 |
| BP | GO:0034374 | low-density lipoprotein particle remodeling | 5/1200 | 16/18723 | 0.002582 | 0.035808 | 0.029759 | PLA2G7/MPO/PLA2G3/PLA2G10/AGTR1 | 5 |
| BP | GO:0050862 | positive regulation of T cell receptor signaling pathway | 5/1200 | 16/18723 | 0.002582 | 0.035808 | 0.029759 | CD226/KCNN4/TRAT1/LCK/ADA | 5 |
| BP | GO:0001780 | neutrophil homeostasis | 5/1200 | 17/18723 | 0.003466 | 0.045132 | 0.037509 | MPL/PIK3CD/MERTK/SLC7A11/PDE4B | 5 |
| BP | GO:0032604 | granulocyte macrophage colony-stimulating factor production | 5/1200 | 17/18723 | 0.003466 | 0.045132 | 0.037509 | IL1B/IL17F/CD80/PAEP/LILRA2 | 5 |
| BP | GO:0032645 | regulation of granulocyte macrophage colony-stimulating factor production | 5/1200 | 17/18723 | 0.003466 | 0.045132 | 0.037509 | IL1B/IL17F/CD80/PAEP/LILRA2 | 5 |
| BP | GO:0033623 | regulation of integrin activation | 5/1200 | 17/18723 | 0.003466 | 0.045132 | 0.037509 | PLEK/SKAP1/SELP/CXCL13/FERMT1 | 5 |
| BP | GO:0036035 | osteoclast development | 5/1200 | 17/18723 | 0.003466 | 0.045132 | 0.037509 | LILRB1/FBN1/TYROBP/LTF/TNFSF11 | 5 |
| BP | GO:0002223 | stimulatory C-type lectin receptor signaling pathway | 5/1200 | 18/18723 | 0.004549 | 0.051638 | 0.042916 | PLCG2/LYN/PAK3/TYROBP/CLEC6A | 5 |
| BP | GO:0006957 | complement activation, alternative pathway | 5/1200 | 18/18723 | 0.004549 | 0.051638 | 0.042916 | CR2/SUSD4/CFH/CR1/C3 | 5 |
| BP | GO:0015732 | prostaglandin transport | 5/1200 | 18/18723 | 0.004549 | 0.051638 | 0.042916 | IL1B/P2RX4/TNFSF11/PLA2G3/PLA2G10 | 5 |
| BP | GO:0030889 | negative regulation of B cell proliferation | 5/1200 | 18/18723 | 0.004549 | 0.051638 | 0.042916 | CTLA4/LYN/BTK/TYROBP/MNDA | 5 |
| BP | GO:0032305 | positive regulation of icosanoid secretion | 5/1200 | 18/18723 | 0.004549 | 0.051638 | 0.042916 | IL1B/P2RX4/TNFSF11/PLA2G3/PLA2G10 | 5 |
| BP | GO:1990840 | response to lectin | 5/1200 | 18/18723 | 0.004549 | 0.051638 | 0.042916 | PLCG2/LYN/PAK3/TYROBP/CLEC6A | 5 |
| BP | GO:1990858 | cellular response to lectin | 5/1200 | 18/18723 | 0.004549 | 0.051638 | 0.042916 | PLCG2/LYN/PAK3/TYROBP/CLEC6A | 5 |
| BP | GO:0002888 | positive regulation of myeloid leukocyte mediated immunity | 5/1200 | 19/18723 | 0.005853 | 0.059905 | 0.049786 | STAP1/BTK/F2RL1/C3/TYROBP | 5 |
| BP | GO:0043031 | negative regulation of macrophage activation | 5/1200 | 19/18723 | 0.005853 | 0.059905 | 0.049786 | CD200/CST7/PTPRC/LRFN5/IL31RA | 5 |
| BP | GO:0051546 | keratinocyte migration | 5/1200 | 19/18723 | 0.005853 | 0.059905 | 0.049786 | KRT16/MMP9/KRT2/FERMT1/HAS2 | 5 |
| BP | GO:0140131 | positive regulation of lymphocyte chemotaxis | 5/1200 | 19/18723 | 0.005853 | 0.059905 | 0.049786 | WNK1/STK39/PTK2B/CCL5/CXCL13 | 5 |
| BP | GO:1903975 | regulation of glial cell migration | 5/1200 | 19/18723 | 0.005853 | 0.059905 | 0.049786 | STAP1/TREM2/GPR183/TIAM1/P2RX4 | 5 |
| CC | GO:0098993 | anchored component of synaptic vesicle membrane | 5/1263 | 12/19550 | 0.000603 | 0.021015 | 0.01891 | RAB3C/RAB11B/RAB3B/CPLX3/SYN1 | 5 |
| MF | GO:0086080 | protein binding involved in heterotypic cell-cell adhesion | 5/1229 | 11/18368 | 0.000437 | 0.016716 | 0.015085 | DSP/DSC2/CD200/JUP/CXADR | 5 |
| MF | GO:0036041 | long-chain fatty acid binding | 5/1229 | 14/18368 | 0.001599 | 0.042817 | 0.038639 | ALOX5AP/UCP1/FABP4/PPARG/GPR31 | 5 |
| BP | GO:0001915 | negative regulation of T cell mediated cytotoxicity | 4/1200 | 10/18723 | 0.002579 | 0.035808 | 0.029759 | PTPRC/LILRB1/NCKAP1L/IL7R | 4 |
| BP | GO:0002934 | desmosome organization | 4/1200 | 10/18723 | 0.002579 | 0.035808 | 0.029759 | DSP/PKP3/PERP/JUP | 4 |
| BP | GO:0003129 | heart induction | 4/1200 | 10/18723 | 0.002579 | 0.035808 | 0.029759 | MESP1/ROBO2/BMP4/WNT11 | 4 |
| BP | GO:0002759 | regulation of antimicrobial humoral response | 4/1200 | 11/18723 | 0.003849 | 0.047655 | 0.039605 | IL17F/IL17A/KLK7/PGC | 4 |
| BP | GO:0032836 | glomerular basement membrane development | 4/1200 | 11/18723 | 0.003849 | 0.047655 | 0.039605 | COL4A4/SULF2/NID1/COL4A3 | 4 |
| BP | GO:0033625 | positive regulation of integrin activation | 4/1200 | 11/18723 | 0.003849 | 0.047655 | 0.039605 | PLEK/SKAP1/CXCL13/FERMT1 | 4 |
| BP | GO:0046643 | regulation of gamma-delta T cell activation | 4/1200 | 11/18723 | 0.003849 | 0.047655 | 0.039605 | PTPRC/LILRB1/NCKAP1L/EGR3 | 4 |
| BP | GO:0070189 | kynurenine metabolic process | 4/1200 | 11/18723 | 0.003849 | 0.047655 | 0.039605 | AADAT/KYNU/TDO2/IDO1 | 4 |
| BP | GO:0071492 | cellular response to UV-A | 4/1200 | 11/18723 | 0.003849 | 0.047655 | 0.039605 | MMP3/MMP1/MMP9/TIMP1 | 4 |
| BP | GO:0098883 | synapse pruning | 4/1200 | 11/18723 | 0.003849 | 0.047655 | 0.039605 | TREM2/C3/C1QA/C1QB | 4 |
| BP | GO:1900426 | positive regulation of defense response to bacterium | 4/1200 | 11/18723 | 0.003849 | 0.047655 | 0.039605 | CYBA/F2RL1/KLK7/PGC | 4 |
| BP | GO:1903238 | positive regulation of leukocyte tethering or rolling | 4/1200 | 11/18723 | 0.003849 | 0.047655 | 0.039605 | ITGA4/FUT7/CHST2/SELP | 4 |
| BP | GO:2000826 | regulation of heart morphogenesis | 4/1200 | 11/18723 | 0.003849 | 0.047655 | 0.039605 | MESP1/ROBO2/BMP4/WNT11 | 4 |
| BP | GO:0001821 | histamine secretion | 4/1200 | 12/18723 | 0.005483 | 0.057578 | 0.047852 | LYN/BTK/PLA2G3/ADA | 4 |
| BP | GO:0002024 | diet induced thermogenesis | 4/1200 | 12/18723 | 0.005483 | 0.057578 | 0.047852 | UCP1/ADRB2/MC4R/ADRB1 | 4 |
| BP | GO:0032490 | detection of molecule of bacterial origin | 4/1200 | 12/18723 | 0.005483 | 0.057578 | 0.047852 | LY96/TREM2/TLR2/TLR1 | 4 |
| BP | GO:0038063 | collagen-activated tyrosine kinase receptor signaling pathway | 4/1200 | 12/18723 | 0.005483 | 0.057578 | 0.047852 | COL4A3/COL4A2/COL4A1/COL4A6 | 4 |
| BP | GO:0098911 | regulation of ventricular cardiac muscle cell action potential | 4/1200 | 12/18723 | 0.005483 | 0.057578 | 0.047852 | DSP/DSC2/JUP/DLG1 | 4 |
| CC | GO:0034663 | endoplasmic reticulum chaperone complex | 4/1263 | 10/19550 | 0.002656 | 0.052934 | 0.047631 | MZB1/DNAJC10/SDF2L1/PDIA6 | 4 |
