# Supplementary material for: Bioinformatics investigation of adaptive immune‐related genes in peri‐implantitis and periodontitis: Characteristics and diagnostic values
Source: Immun Inflamm Dis. 2024 May 23;12(5):e1272. doi: 10.1002/iid3.1272 (PMC11112631; doi:10.1002/iid3.1272)
Supplement: Supplementary file 6 — Supporting information. [file IID3-12-e1272-s007.docx]

**Supplementary Table 6. KEGG enrichment analysis of** **differential expression genes in P vs HP groups**

| **ID** | **Description** | **GeneRatio** | **BgRatio** | **pvalue** | **p.adjust** | **qvalue** | **geneID** | **Count** | **GeneRatio** |
| --- | --- | --- | --- | --- | --- | --- | --- | --- | --- |
| hsa04080 | Neuroactive ligand-receptor interaction | 46/606 | 362/8159 | 0.000202 | 0.004945 | 0.004242 | PNOC/CCK/P2RY1/GZMA/P2RY10/NPY2R/P2RX5/GHR/ADRB2/P2RX4/PARD3/P2RY13/P2RX1/P2RX7/MC4R/LYPD6B/F2RL1/SLURP1/THRB/C3/CHRNA3/LPAR6/GLP2R/GRM8/C3AR1/HTR2B/GRIK2/LYPD6/GABRR2/CHRM4/UTS2/FPR1/KNG1/HTR7/PTGIR/AGTR1/ADRA2A/TSHR/EDN2/NPBWR1/NMUR1/ADRB1/F2/SST/PMCH/CGA | 46 | 0.0759 |
| hsa04151 | PI3K-Akt signaling pathway | 41/606 | 354/8159 | 0.002735 | 0.040253 | 0.034535 | CD19/LAMB4/COL4A4/GNG7/GNGT2/ITGA4/PPP2R3A/MYC/CCND2/FLT3/GHR/PPP2R2C/TLR2/EFNA5/ITGA8/MAGI1/COL4A3/PCK1/PIK3R1/COL4A2/EGF/IL7R/PIK3CG/COL4A1/FGFR4/FGF19/LAMC3/FGFR2/CREB3L2/LPAR6/IL2RA/NTF3/THBS2/SGK2/IL2RG/EFNA3/PIK3CD/COL4A6/BCL2L1/NTRK2/LAMC2 | 41 | 0.0677 |
| hsa04060 | Cytokine-cytokine receptor interaction | 39/606 | 295/8159 | 0.000274 | 0.006234 | 0.005348 | TNFRSF17/IL26/IL24/IL21/CXCR4/TNFRSF19/GHR/CXCL1/IL1B/MPL/LTB/CXCL3/IL17F/EDA2R/CXCL2/TGFB3/IL7R/CCL5/IL17A/CXCL6/CCL18/IL2RA/BMP4/TNFRSF9/CXCL13/ACVR1B/CD40LG/GDF6/IL2RG/IL12RB2/CCR3/TNFSF11/IL13RA2/TNFSF8/IL31RA/CCL13/CXCR1/PF4V1/BMP5 | 39 | 0.0644 |
| hsa05417 | Lipid and atherosclerosis | 33/606 | 215/8159 | 4.64E-05 | 0.002056 | 0.001764 | MMP3/LY96/XBP1/VAV1/CYBA/NCF4/HSPA2/ERN1/CYBB/LYN/TLR2/CXCL1/CALML5/IL1B/CD14/SELP/CXCL3/VAV3/MMP1/MMP9/PIK3R1/CXCL2/CCL5/PPARG/HSPA1B/NCF2/CYP2J2/HSPA1A/CD40LG/NFATC2/PIK3CD/HSPA1L/BCL2L1 | 33 | 0.0545 |
| hsa04062 | Chemokine signaling pathway | 30/606 | 192/8159 | 7.30E-05 | 0.00233 | 0.001999 | PLCG2/VAV1/GNG7/GNGT2/PREX1/CXCR4/LYN/PTK2B/CXCL1/TIAM1/CXCL3/PARD3/PRKCB/VAV3/FGR/PIK3R1/CXCL2/GRK5/ITK/CCL5/PIK3CG/CXCL6/ARRB2/CCL18/CXCL13/PIK3CD/CCR3/CCL13/CXCR1/PF4V1 | 30 | 0.0495 |
| hsa04141 | Protein processing in endoplasmic reticulum | 26/606 | 171/8159 | 0.00034 | 0.006772 | 0.00581 | SSR3/SSR4/XBP1/ERLEC1/EDEM1/HSPA2/ERN1/UBE2J1/SEL1L/TXNDC5/DNAJC10/PDIA4/SEC24D/HERPUD1/UGGT1/CRYAB/RPN1/DNAJB1/PDIA6/HSPA1B/DNAJC3/HSPA1A/SVIP/HSPA4L/HSPA1L/DNAJC5B | 26 | 0.0429 |
| hsa04514 | Cell adhesion molecules | 23/606 | 157/8159 | 0.001236 | 0.020753 | 0.017805 | CD226/ICAM2/CD86/ITGA4/CLDN10/SELL/CTLA4/PECAM1/PTPRC/ICOS/ITGA8/PTPRF/SELP/CLDN3/NRCAM/CD80/OCLN/CDH1/CD40LG/CD8B/SIGLEC1/CLDN9/SLITRK4 | 23 | 0.038 |
| hsa04670 | Leukocyte transendothelial migration | 22/606 | 114/8159 | 2.68E-05 | 0.002056 | 0.001764 | PLCG2/VAV1/CYBA/NCF4/ITGA4/CLDN10/RHOH/PECAM1/CYBB/CXCR4/PTK2B/PRKCB/VAV3/MMP9/PIK3R1/CLDN3/ITK/OCLN/NCF2/SIPA1/PIK3CD/CLDN9 | 22 | 0.0363 |
| hsa04380 | Osteoclast differentiation | 22/606 | 128/8159 | 0.000164 | 0.004767 | 0.00409 | LILRB2/PLCG2/CYBA/NCF4/LILRB3/TREM2/BTK/IL1B/LILRB1/PIK3R1/LILRA5/LILRA6/LILRA1/PPARG/LCK/TYROBP/NCF2/NFATC2/PIK3CD/MITF/TNFSF11/LILRA2 | 22 | 0.0363 |
| hsa04390 | Hippo signaling pathway | 22/606 | 157/8159 | 0.002776 | 0.040253 | 0.034535 | DLG2/MYC/CCND2/SOX2/PPP2R2C/BIRC3/PARD3/WNT2B/WNT3/TGFB3/FZD5/RASSF6/BMP4/PARD6A/CDH1/GDF6/DLG1/AJUBA/WNT11/WNT16/BMP5/FZD10 | 22 | 0.0363 |
| hsa04915 | Estrogen signaling pathway | 21/606 | 138/8159 | 0.001218 | 0.020753 | 0.017805 | KRT16/HSPA2/KRT18/KRT33A/CALML5/MMP9/PIK3R1/KRT13/HSPA1B/KRT27/CREB3L2/KRT10/HSPA1A/PIK3CD/HSPA1L/KRT31/KRT34/KRT24/TFF1/KRT25/KRT26 | 21 | 0.0347 |
| hsa04662 | B cell receptor signaling pathway | 20/606 | 82/8159 | 1.49E-06 | 0.00028 | 0.000241 | CD19/LILRB2/CR2/PLCG2/VAV1/CD79A/LILRB3/LYN/BTK/LILRB1/PRKCB/VAV3/PIK3R1/LILRA5/LILRA6/LILRA1/NFKBIE/NFATC2/PIK3CD/LILRA2 | 20 | 0.033 |
| hsa05150 | Staphylococcus aureus infection | 20/606 | 96/8159 | 1.94E-05 | 0.002056 | 0.001764 | KRT16/KRT18/KRT33A/SELP/CFH/KRT13/C3/KRT27/C3AR1/KRT10/C1QA/DEFB1/KRT31/FPR1/MBL2/KRT34/C1QB/KRT24/KRT25/KRT26 | 20 | 0.033 |
| hsa05146 | Amoebiasis | 20/606 | 102/8159 | 4.88E-05 | 0.002056 | 0.001764 | LAMB4/COL4A4/TLR2/CXCL1/IL1B/CD14/CXCL3/PRKCB/COL4A3/PIK3R1/CXCL2/TGFB3/COL4A2/COL4A1/LAMC3/SERPINB13/PIK3CD/COL4A6/CD1E/LAMC2 | 20 | 0.033 |
| hsa04064 | NF-kappa B signaling pathway | 20/606 | 104/8159 | 6.51E-05 | 0.002308 | 0.001981 | LY96/PLCG2/LYN/BCL2A1/PRKCQ/BTK/CXCL1/IL1B/CD14/BIRC3/LTB/CXCL3/PRKCB/EDA2R/CXCL2/LCK/CD40LG/BCL2L1/TNFSF11/CCL13 | 20 | 0.033 |
| hsa05323 | Rheumatoid arthritis | 19/606 | 93/8159 | 4.15E-05 | 0.002056 | 0.001764 | MMP3/CD86/CTLA4/TLR2/CXCL1/IL1B/LTB/CXCL3/MMP1/TCIRG1/CXCL2/TGFB3/CTSL/CD80/CCL5/IL17A/CXCL6/ATP6V0D2/TNFSF11 | 19 | 0.0314 |
| hsa04660 | T cell receptor signaling pathway | 19/606 | 104/8159 | 0.0002 | 0.004945 | 0.004242 | CD3D/VAV1/CD3G/CTLA4/CD247/PRKCQ/PTPRC/ICOS/VAV3/PIK3R1/ITK/PAK3/LCK/NFKBIE/CD40LG/CD8B/NFATC2/PIK3CD/DLG1 | 19 | 0.0314 |
| hsa04640 | Hematopoietic cell lineage | 18/606 | 99/8159 | 0.000311 | 0.006624 | 0.005683 | CD19/CR2/CD3D/CD38/CR1L/ITGA4/CD3G/FLT3/CD37/IL1B/CD14/GP1BA/MS4A1/CR1/IL7R/IL2RA/CD8B/CD1E | 18 | 0.0297 |
| hsa04610 | Complement and coagulation cascades | 15/606 | 85/8159 | 0.001328 | 0.021179 | 0.018171 | CR2/CR1L/ITGAX/F5/F12/CFH/CR1/C3/C3AR1/CD46/C1QA/KNG1/MBL2/C1QB/F2 | 15 | 0.0248 |
| hsa05134 | Legionellosis | 14/606 | 57/8159 | 5.16E-05 | 0.002056 | 0.001764 | NLRC4/CR1L/HSPA2/TLR2/CXCL1/IL1B/CD14/CXCL3/CXCL2/CR1/HSPA1B/C3/HSPA1A/HSPA1L | 14 | 0.0231 |
| hsa05340 | Primary immunodeficiency | 13/606 | 38/8159 | 1.76E-06 | 0.00028 | 0.000241 | CD19/CD3D/CD79A/IGLL1/PTPRC/BTK/ICOS/IL7R/LCK/CD40LG/CD8B/IL2RG/ADA | 13 | 0.0215 |
| hsa03320 | PPAR signaling pathway | 13/606 | 75/8159 | 0.003211 | 0.044533 | 0.038207 | UCP1/MMP1/SLC27A6/PCK1/DBI/FABP4/PPARG/CYP7A1/SLC27A2/GK2/SCD/PLTP/HMGCS1 | 13 | 0.0215 |
| hsa05144 | Malaria | 11/606 | 50/8159 | 0.000895 | 0.016796 | 0.01441 | CR1L/KLRB1/PECAM1/TLR2/IL1B/SELP/TGFB3/GYPC/CR1/THBS2/CD40LG | 11 | 0.0182 |
