# Supplementary material for: Bioinformatics investigation of adaptive immune‐related genes in peri‐implantitis and periodontitis: Characteristics and diagnostic values
Source: Immun Inflamm Dis. 2024 May 23;12(5):e1272. doi: 10.1002/iid3.1272 (PMC11112631; doi:10.1002/iid3.1272)
Supplement: Supplementary file 7 — Supporting information. [file IID3-12-e1272-s005.docx]

**Supplementary Table 7. 84 immune-related genes in the protein-protein interaction network diagram**

| **Attribute** | **Tag** |
| --- | --- |
| UP | TNFRSF17, FCRLA, FCRL2, STAP1, CD19, CD180, MMP12, RGS1, CD38, CTSS, SAMSN1, SELL, P2RY10, CD53, IL17F, ST8SIA4, SCG2, PLEK, PNOC, IL26, CD200, MNDA, FCRL5, COL4A4, MS4A1, CYBB, CR2, RCSD1, GLIPR1, STAT5A, CD37, CLECL1, ITGA4, AIM2, IL17A, TAGAP, P2RX5, NKG7, SKAP1, PTGIR, GPR18, PDE4B, ADAM28, GNG7, TLR9, MS4A6A, MARCO, ADAM8, SELP, FCRL1, CD3D, SH3KBP1, BIRC3, GNLY, C3AR1, CD3G, ATM, FCER1G, CD52, SIGLEC1, RARA, AKNA, LTC4S, SIGLEC10, CCL5, ITK, GZMM |
| DOWN | FCER1A, ZCCHC2, RYR1, GATM, IVNS1ABP, AQP3, F12, RTKN2, RAB27B, OSBPL1A, DUSP14, ANKRD22, MYC, CSRP2, KLF5, GSTA4, NEFL |
