# Supplementary material for: Bioinformatics investigation of adaptive immune‐related genes in peri‐implantitis and periodontitis: Characteristics and diagnostic values
Source: Immun Inflamm Dis. 2024 May 23;12(5):e1272. doi: 10.1002/iid3.1272 (PMC11112631; doi:10.1002/iid3.1272)
Supplement: Supplementary file 8 — Supporting information. [file IID3-12-e1272-s006.docx]

**Supplementary Table 8. Correlation between Hub gene and 14 kinds of adaptive immune cells**

| **Immune cells types** | ***CD19*** | | ***CD38*** | | **SELL** | | **IL17A** | | **CD53** | | ***PLEK*** | | ***CYBB*** | |
| --- | --- | --- | --- | --- | --- | --- | --- | --- | --- | --- | --- | --- | --- | --- |
|  | **r** | ***P*** | **r** | ***P*** | **r** | ***P*** | **r** | ***P*** | **r** | ***P*** | **r** | ***P*** | **r** | ***P*** |
| Activated B cell | 0.787 | <0.001 | 0.852 | <0.001 | 0.671 | 0.006 | 0.517 | 0.049 | 0.672 | 0.006 | 0.466 | 0.080 | 0.547 | 0.035 |
| Activated CD4^+^ T cell | -0.244 | 0.382 | 0.229 | 0.411 | 0.483 | 0.068 | 0.117 | 0.679 | 0.613 | 0.015 | 0.579 | 0.024 | 0.595 | 0.019 |
| Activated CD8^+^ T cell | 0.574 | 0.025 | 0.490 | 0.064 | 0.681 | 0.005 | 0.611 | 0.015 | 0.671 | 0.006 | 0.676 | 0.006 | 0.627 | 0.012 |
| Central memory CD4^+^ T cell | 0.402 | 0.137 | 0.694 | 0.004 | 0.625 | 0.013 | 0.262 | 0.346 | 0.618 | 0.014 | 0.387 | 0.154 | 0.554 | 0.032 |
| Central memory CD8^+^ T cell | 0.166 | 0.555 | 0.449 | 0.093 | 0.731 | 0.002 | 0.290 | 0.295 | 0.729 | 0.002 | 0.467 | 0.079 | 0.532 | 0.041 |
| Effector memory CD4^+^ T cell | -0.390 | 0.150 | 0.231 | 0.408 | 0.276 | 0.319 | -0.134 | 0.633 | 0.501 | 0.057 | 0.375 | 0.168 | 0.538 | 0.039 |
| Effector memory CD8^+^ T cell | -0.310 | 0.260 | 0.323 | 0.241 | 0.695 | 0.004 | 0.298 | 0.280 | 0.803 | <0.001 | 0.657 | 0.008 | 0.716 | 0.003 |
| Immature B cell | 0.600 | 0.018 | 0.876 | <0.001 | 0.793 | <0.001 | 0.562 | 0.029 | 0.855 | <0.001 | 0.613 | 0.015 | 0.742 | 0.002 |
| Memory B cell | 0.326 | 0.236 | 0.086 | 0.760 | 0.116 | 0.679 | -0.216 | 0.440 | 0.083 | 0.768 | 0.192 | 0.494 | 0.121 | 0.668 |
| Regulatory T cell | 0.490 | 0.064 | 0.666 | 0.007 | 0.842 | <0.001 | 0.502 | 0.057 | 0.871 | <0.001 | 0.811 | <0.001 | 0.829 | <0.001 |
| T follicular helper cell | 0.487 | 0.065 | 0.419 | 0.120 | 0.613 | 0.015 | 0.439 | 0.102 | 0.619 | 0.014 | 0.565 | 0.028 | 0.510 | 0.052 |
| Type 1 T helper cell | 0.758 | 0.001 | 0.616 | 0.014 | 0.684 | <0.001 | 0.582 | 0.023 | 0.622 | 0.013 | 0.508 | 0.053 | 0.522 | 0.046 |
| Type 17 T helper cell | 0.483 | 0.068 | -0.204 | 0.466 | 0.144 | 0.608 | 0.660 | 0.007 | -0.077 | 0.784 | -0.003 | 0.991 | -0.098 | 0.729 |
| Type 2 T helper cell | -0.519 | 0.047 | -0.242 | 0.384 | 0.275 | 0.322 | 0.044 | 0.875 | 0.301 | 0.276 | 0.467 | 0.079 | 0.299 | 0.279 |
