# Supplementary material for: Bioinformatics investigation of adaptive immune‐related genes in peri‐implantitis and periodontitis: Characteristics and diagnostic values
Source: Immun Inflamm Dis. 2024 May 23;12(5):e1272. doi: 10.1002/iid3.1272 (PMC11112631; doi:10.1002/iid3.1272)
Supplement: Supplementary file 9 — Supporting information. [file IID3-12-e1272-s008.docx]

**Supplementary Table 9. 20 immune factors related to activated CD4^+^ T cell**

| **Types** | **genes** | **r** | ***P*** |
| --- | --- | --- | --- |
| chemokine | CCL2 | 0.579 | 0.024 |
|  | CCL25 | -0.528 | 0.043 |
|  | CCL5 | 0.637 | 0.011 |
|  | CXCL1 | 0.682 | 0.005 |
|  | CXCL13 | 0.647 | 0.009 |
|  | XCL1 | 0.764 | 0.001 |
| Immuno-inhibitor | CD274 | 0.599 | 0.018 |
|  | CD96 | 0.644 | 0.01 |
|  | HAVCR2 | 0.629 | 0.012 |
|  | IL10RB | 0.651 | 0.009 |
|  | PDCD1LG2 | 0.664 | 0.007 |
|  | TGFBR1 | 0.596 | 0.019 |
| Immuno-stimulator | CD80 | 0.562 | 0.029 |
|  | CD86 | 0.536 | 0.039 |
|  | LTA | -0.636 | 0.011 |
|  | TNFRSF18 | -0.564 | 0.029 |
| MHC | B2M | 0.845 | <0.001 |
|  | TAPBP | 0.537 | 0.039 |
| receptor | CCR10 | -0.625 | 0.013 |
|  | CXCR5 | -0.522 | 0.041 |
